# Discovery of GLPG2737, a Potent Type 2 Corrector of CFTR for the Treatment of Cystic Fibrosis in Combination with a Potentiator and a Type 1 Co-corrector

**DOI:** 10.1021/acs.jmedchem.3c01790

**Published:** 2024-03-25

**Authors:** Mathieu Pizzonero, Rhalid Akkari, Xavier Bock, Romain Gosmini, Elsa De Lemos, Béranger Duthion, Gregory Newsome, Thi-Thu-Trang Mai, Virginie Roques, Hélène Jary, Jean-Michel Lefrancois, Laetitia Cherel, Vanessa Quenehen, Marielle Babel, Nuria Merayo, Natacha Bienvenu, Oscar Mammoliti, Ghjuvanni Coti, Adeline Palisse, Marlon Cowart, Anurupa Shrestha, Stephen Greszler, Steven Van Der Plas, Koen Jansen, Pieter Claes, Mia Jans, Maarten Gees, Monica Borgonovi, Gert De Wilde, Katja Conrath

**Affiliations:** 1 Galapagos SASU, 102 Avenue Gaston Roussel, 93230 Romainville, France; 2 Galapagos NV, Generaal De Wittelaan L11, A3, 2800 Mechelen, Belgium; 3 AbbVie, Inc., 1 North Waukegan Road, North Chicago, Illinois 60064-1802, United States

## Abstract

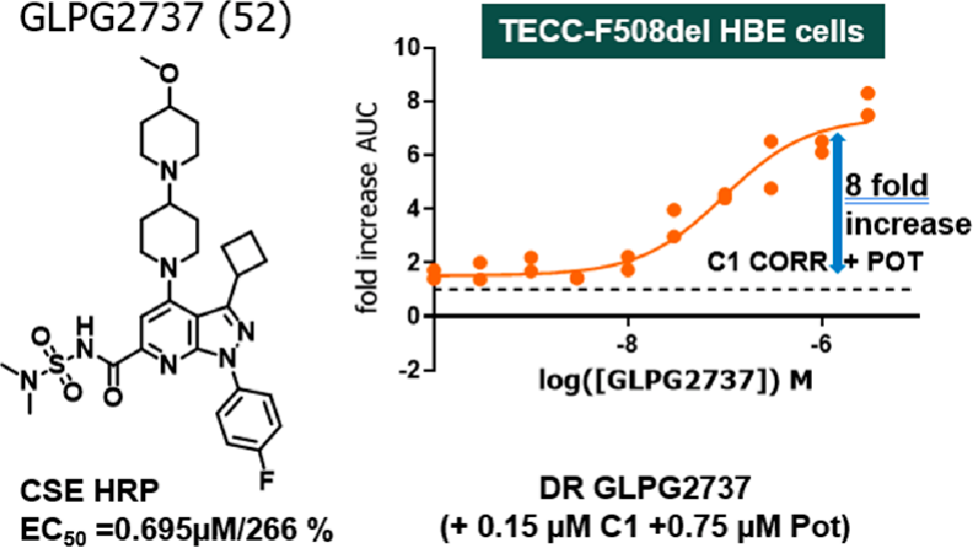

Cystic fibrosis (CF) is caused by mutations in the CF transmembrane conductance regulator (CFTR) protein. This epithelial anion channel regulates the active transport of chloride and bicarbonate ions across membranes. Mutations result in reduced surface expression of CFTR channels with impaired functionality. Correctors are small molecules that support the trafficking of CFTR to increase its membrane expression. Such correctors can have different mechanisms of action. Combinations may result in a further improved therapeutic benefit. We describe the identification and optimization of a new pyrazolol3,4-bl pyridine-6-carboxylic acid series with high potency and efficacy in rescuing CFTR from the cell surface. Investigations showed that carboxylic acid group replacement with acylsulfonamides and acylsulfonylureas improved ADMET and PK properties, leading to the discovery of the structurally novel co-corrector GLPG2737. The addition of GLPG2737 to the combination of the potentiator GLPG1837 and C1 corrector **4** led to an 8-fold increase in the F508del CFTR activity.

## Introduction

Cystic fibrosis (CF) is the most common life-limiting autosomal recessive disorder in Caucasian populations and is currently estimated to affect over 100,000 people worldwide.^1^ CF is caused by mutations in the cystic fibrosis transmembrane conductance regulator (CFTR) protein. CFTR is an epithelial anion channel that primarily regulates the active transport of chloride and bicarbonate ions across the membrane. In patients with CF, the CFTR channel is either dysfunctional or produced in insufficient quantity or a combination of both, which leads to a buildup of thick mucus in the respiratory and digestive epithelial membranes. Although CF is a systemic disease, the primary cause of death in patients with CF is lung disease.^2^ This is caused by the accumulated mucus trapping bacteria in the airways, leading to infection, lung damage, and eventually respiratory failure. Over the past decade, a number of small-molecule disease-modifying therapies, known as CFTR modulators, have been developed and approved for the treatment of CF.^3^ These therapies directly target the functional consequences of the CFTR mutations. There are more than 2000 known mutations of the CFTR gene,^4^ of which more than 400 have documented clinical significance.^5^ CFTR mutations are categorized into six broad classes. Approximately 10% of patients with CF have class III mutations, which are amenable to pharmacological intervention, the most common of which is G551D. This mutation results in CFTR proteins that are expressed at the membrane, but the channel has a substantially reduced ability to open and function correctly. Modulators that increase the ion conductance of the CFTR protein are known as potentiators. Ivacaftor is the only potentiator approved as monotherapy in patients with the G551D mutation.^6,7^ The most common class II mutation–and the most common CFTR mutation overall–is ΔF508, with approximately 80% of patients with CF in Europe having at least one such allele.^2^ Class II mutations prevent the proper folding and assembly of the CFTR protein, which results in very few copies reaching the membrane, many of which are dysfunctional. Modulators such as lumacaftor (VX-809) and tezacaftor (VX-661) that function by supporting membrane trafficking of CFTR proteins with class II mutations to increase the quantity expressed on the membrane are known as correctors.^3^ The dual potentiator/corrector combinations ivacaftor/lumacaftor and ivacaftor/tezacaftor are approved for the treatment of patients homozygous for ΔF508, and homozygous or heterozygous for ΔF508, respectively. Nonetheless, the clinical improvements in lung function observed in patients treated with these therapies are moderate.^3^ As a result, there is a need for further development of “co-correctors” or C2 correctors.

Correctors are classified as type 1 (C1 correctors) or type 2 correctors (C2 correctors) according to how complementary their mechanisms of action are. C1 correctors, such as VX-809 (lumacaftor), stabilize the first membrane-spanning domain (MSD1) of the CFTR protein, which is the part that interacts with the cell membrane. C2 correctors are molecules which have additive or synergistic functional effects by any complementary mechanism of action.^8^ Elexacaftor, a co-corrector, has recently been approved in combination with ivacaftor/tezacaftor as a triple therapy (Trikafta) for patients with at least one ΔF508 mutation. Notably, ivacaftor/tezacaftor/elexacaftor triple therapy demonstrated a clinically and statistically significant improvement in lung function compared with ivacaftor/tezacaftor alone.^9^

Herein, we report the discovery process of the structurally novel co-corrector GLPG2737, a small molecule that is mechanistically different from elexacaftor and provides an additive functional effect when combined with a potentiator/corrector.^9,10^ This article details the structure–activity relationship (SAR), the optimization of the *in vitro* pharmacological and *in vivo* pharmacokinetic (PK) parameters, and the chemical synthesis that led to the identification of GLPG2737.

## Results and Discussion

### GLPG2737 Profile

**Figure 1:**
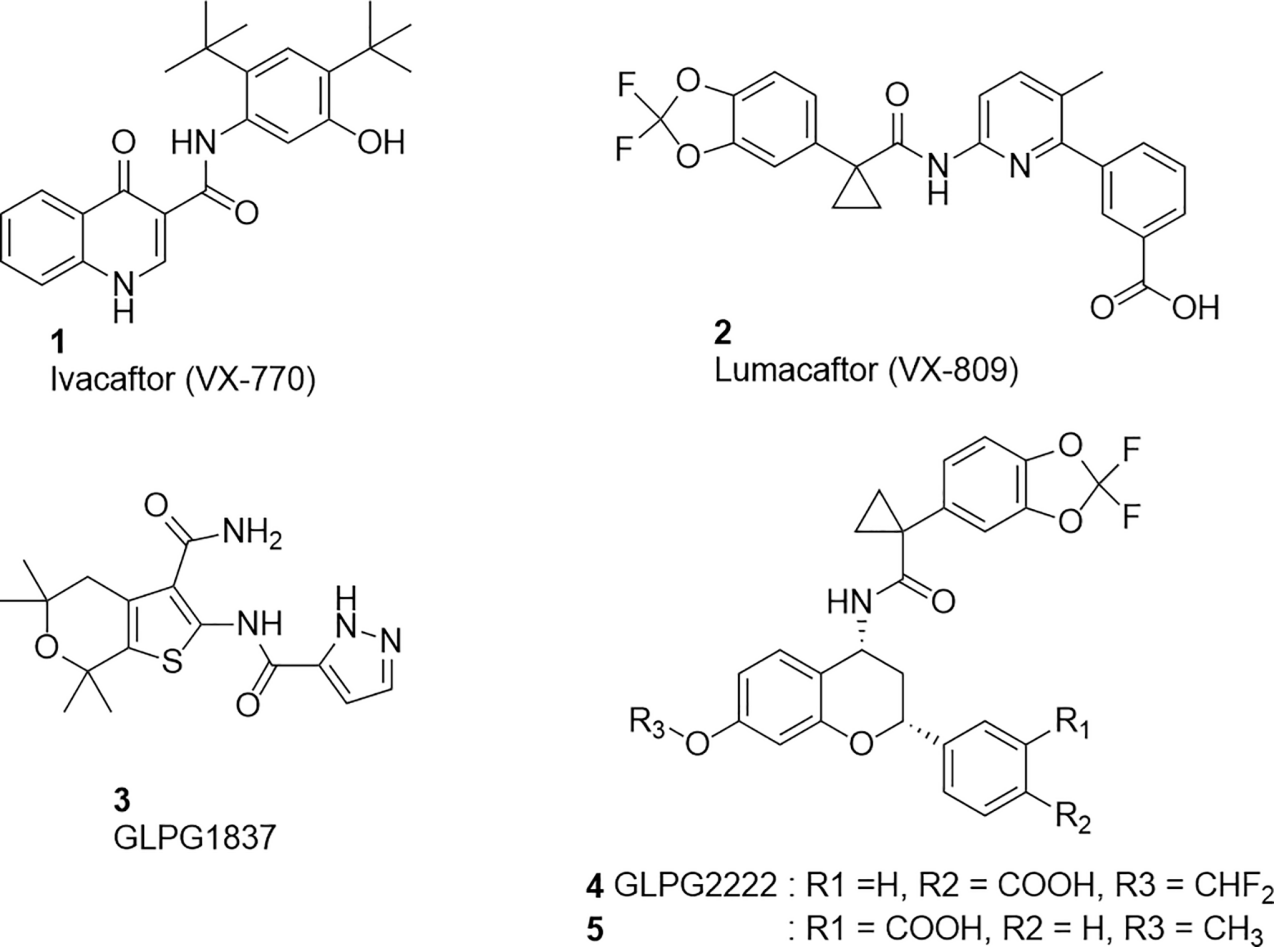
Structures of existing potentiators (**1** and **3**) and correctors (**2, 4**, and **5**).

The structurally novel co-corrector GLPG2737 was designed to provide a crucial building block for triple combination therapy in CF. To ensure a suitable efficacy profile, GLPG2737 was required to increase ΔF508 CFTR protein levels of at least 50% in multiple patient samples compared with wild type protein levels when combined with an existing potentiator (GLPG1837 or ivacaftor/VX-770) and a C1 corrector (lumacaftor/VX809, GLPG2222 or **5**) (Figure 1).^11−13^

Examples of existing potentiators and correctors are shown in Figure 1.

### Hit Identification

A high throughput screening (HTS) campaign was undertaken for a diverse range of compounds (105,000 unique structures in total) using a lung epithelial cell line stably expressing the ΔF508 CFTR protein and that had been incubated with the screened compound for 24 h.^10^ This was carried out in the presence or absence of a 3 μM concentration of C1 corrector, lumacaftor (Figure 1), to identify a series of compounds with the ability to rescue trafficking of the ΔF508 CFTR protein to the plasma membrane and thus increase its cell surface expression.

Following the HTS campaign, a pyrazolo-pyridine compound (**6**; Table 1) was identified with an interesting profile combining moderate potency and efficacy and no metabolic stability liability. Opportunistic expansion identified a commercially available close analogue of this Hit, **7** (Table 1), devoid of the methyl group at the *para* position of the phenyl ring in N1.

**Table 1:**
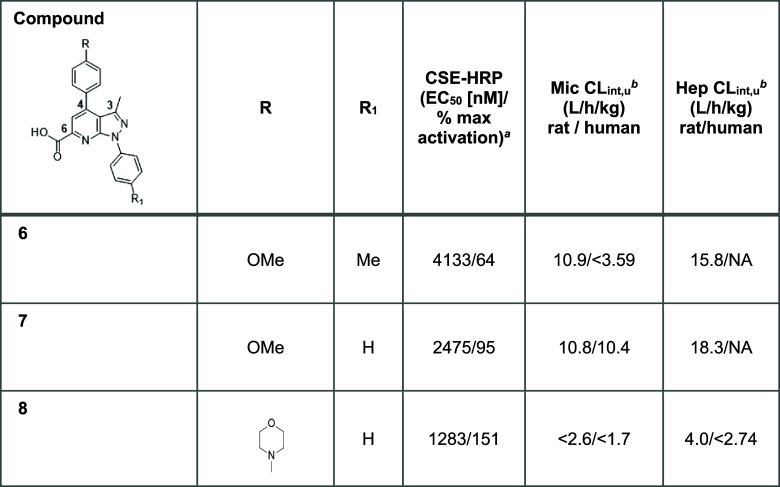
Initial Hits from the HTS Campaign and Expansion at N1 and C4^c^

a Assay conditions are described in the Experimental Section using lumacaftor or **5** (3 μM) as the positive control. All values are the geometric mean calculated from at least two runs.

b Calculated from measured fumic (unbound fraction in microsomes).

c CL_int_, intrinsic clearance; CSE-HRP, cell surface expression-horse radish peroxidase; mic, microsome; hep, hepatocyte.

**7** showed both improved potency and efficacy compared with **6**, but it was found to have high unbound clearance in rat and human microsomes and rat hepatocytes. Interestingly, the ester analogue of **7** (data not shown) was found to be inactive, illustrating the key role of the carboxylic acid moiety in maintaining activity. Replacement of the methoxy group with the electron donating morpholine on the C4 phenyl group (**8**; Table 1) resulted in improved efficacy and potency and reduced *in vitro* clearance, offering a positive balance for initiating SAR investigation and multiparametric optimization. This analogue was therefore used as a reference for SAR exploration going forward.

### SAR Optimization

During the first stages of SAR optimization, the 4-phenyl-1*H*-pyrazolo[3,4-*b*]-pyridine-6-carboxylic acid core was maintained, while the SAR exploration focused on the exit vectors N1, C3, and C6.

The N1 SAR exploration is detailed above in Table 2. Fluoro substitution at the *meta* or *para* positions of a phenyl group at the N1 position (**9** and **10**, respectively) improved potency over **8**, but tended to lower efficacy. Conversely, substitution of a cyclopropyl moiety at this position (**11**) resulted in a loss of potency, while the larger cyclohexyl ring (**12**) enabled retention of potency at the expense of a decrease in efficacy that accompanied the increase in lipophilicity. Finally, the introduction of more polar nonaromatic heterocycles (**13** and **14**) substantially reduced potency. Therefore, analogue **8** with a phenyl group at the N1 position was taken forward for further development.

**Table 2:**
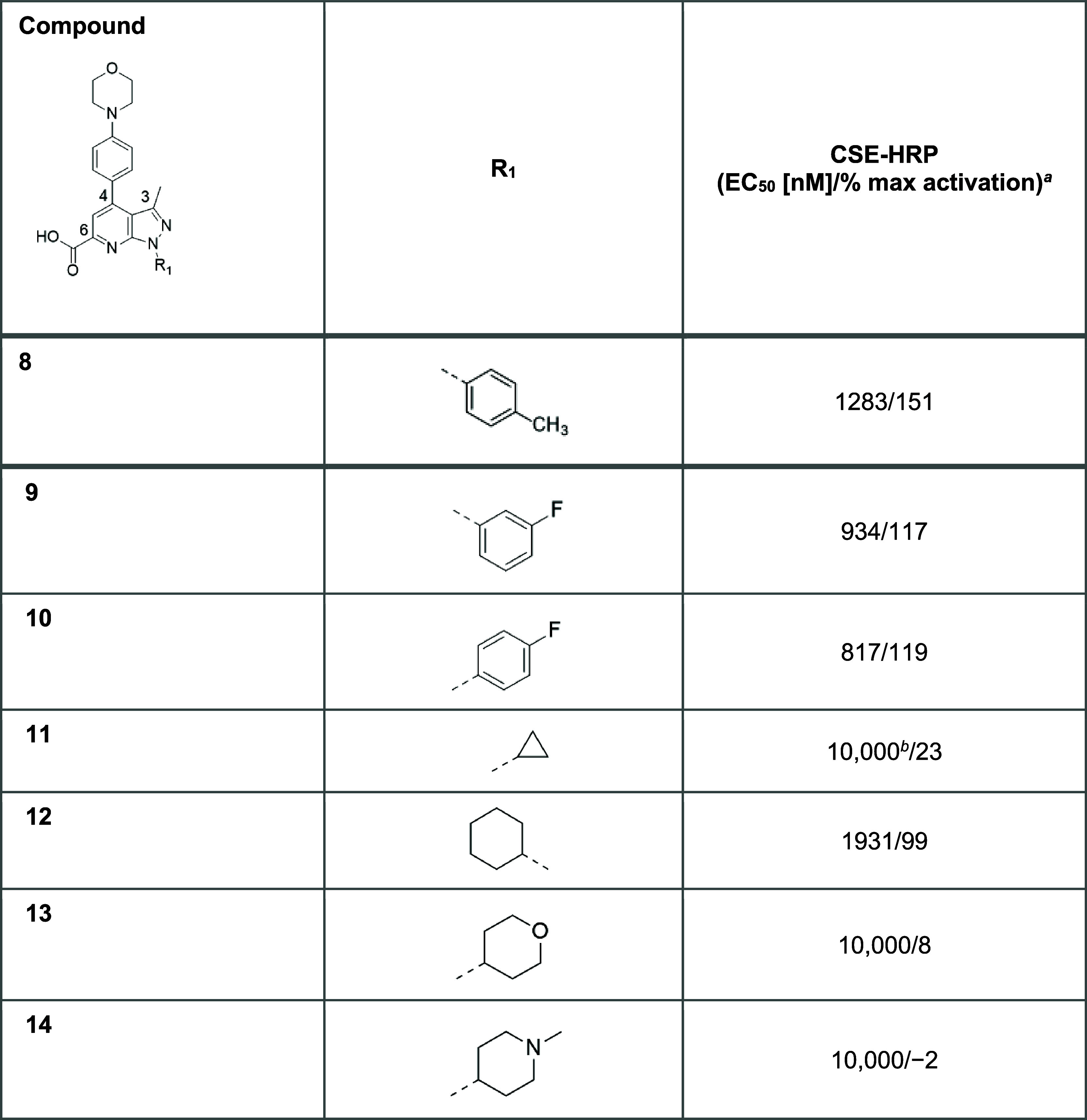
SAR Exploration at N1^c^

a Assay conditions are described in the Experimental Section using Lumacaftor or 5 (3 μM) as the positive control. All values are the geometric mean calculated from at least two runs, except when mentioned.

b Only one measure was done.

c CSE-HRP, cell surface expression-horse radish peroxidase.

Next, C3 SAR exploration was undertaken, the results of which are detailed in Table 3.

**Table 3:**
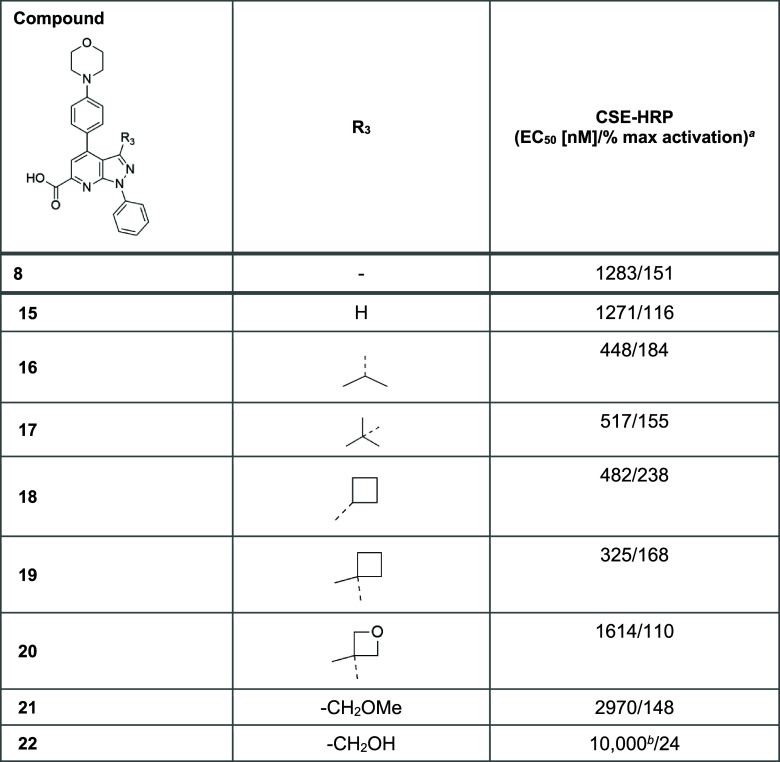
SAR Exploration at C3^c^

a Assay conditions are described in the Experimental Section using lumacaftor or **5** (3 μM) as the positive control. All values are the geometric mean calculated from at least two runs, except when mentioned.

b Only one measure was done.

c CSE-HRP, cell surface expression-horse radish peroxidase.

The introduction of aliphatic groups such as *i*-propyl, *t*-butyl, and cyclobutyl (**16**, **17**, and **18**, respectively) resulted in meaningful improvements in both potency and efficacy compared with the unsubstituted analogue (**15**). In contrast, the introduction of more polar ether moieties, both cyclic (**20**) and acyclic (**21**), retained reasonable efficacy; however, this was at the expense of a loss of potency. Substitution with a hydrogen bond donor primary alcohol (**22**) abolished activity, revealing the importance of a lipophilic moiety at C3. Although aliphatic groups and in particular the cyclobutyl moiety were found to be the most favorable substituents at the C3 position, SAR exploration was pursued on the C4 and C6 positions by retaining the methyl group in C3 to simplify molecular matched pair analysis.

The results of the subsequent exploration at C4 are detailed in Table 4. Replacing the phenyl linker with a pyridine ring had only a minor impact on both the potency and the efficacy of the molecule (**23**). For the series with the original phenyl linker, opening the morpholine ring at position C4 to afford a secondary aniline (**24**) ensured retention of potency, and substitution of the nitrogen with a methyl group to yield the less polar tertiary aniline (**25**) enabled a noticeable improvement in efficacy. The acyclic tertiary aniline (**26**) demonstrated significant gains in both efficacy and potency of more than 200%.

**Table 4:**
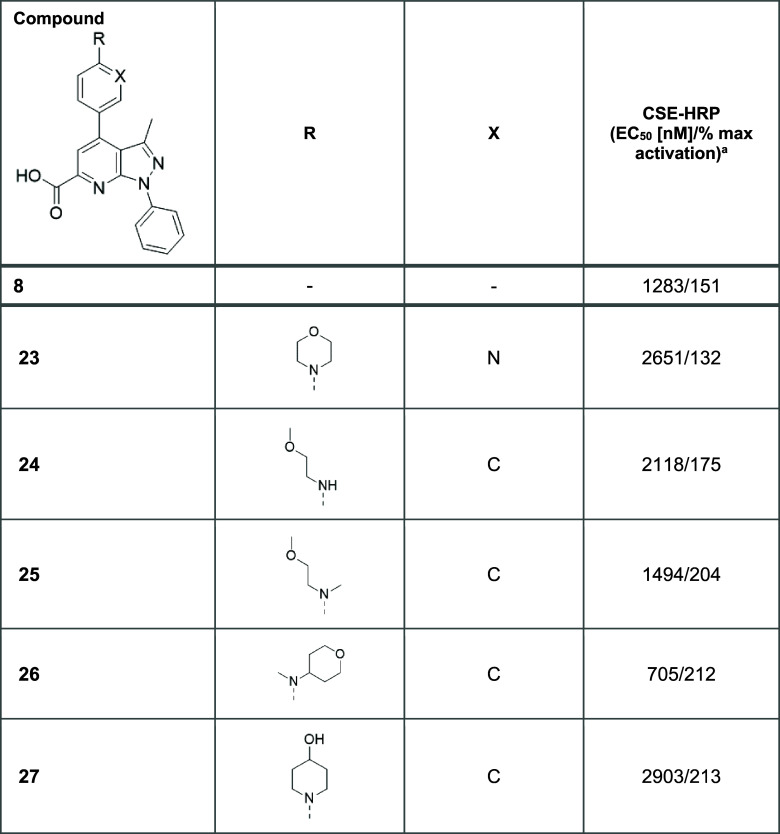
SAR Exploration at C4^b^

a Assay conditions are described in the Experimental Section using lumacaftor or **5** (3 μM) as the positive control. All values are the geometric mean calculated from at least two runs.

b CSE-HRP, cell surface expression-horse radish peroxidase.

The final analogues in this series investigated the effect of introducing more polar substituents at C4, including hydroxyl (**27**) and cyano (**28**) moieties via the cyclic aniline, while retaining the phenyl linker between the pyrazolo-pyridine and C4 substituent. Although the introduction of a hydrogen bond donor (hydroxyl group) on the piperidine ring facilitated an improvement in efficacy, this was accompanied by a slight decrease in potency. Overall, the cyano-substituted piperidine ring offered the best combination of improved efficacy and potency out of the analogues examined during C4 SAR investigation.

The subsequent SAR exploration examined the impact on potency and efficacy of substituting the carboxylic acid moiety for alternative substituents of varying polarity at C6. The results of this campaign are detailed in Table 5.

**Table 5:**
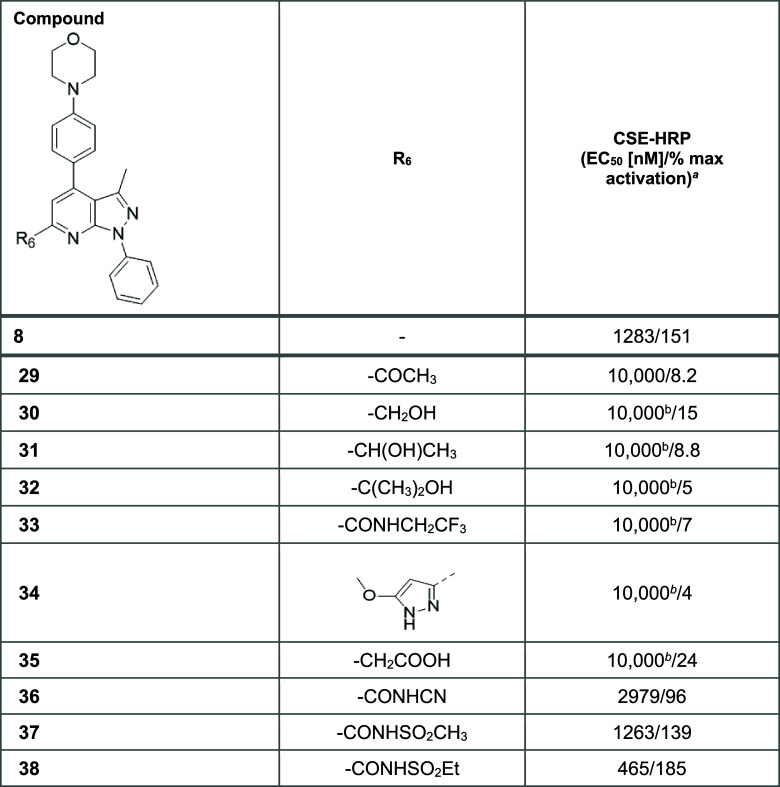
SAR Exploration at C6^c^

a Assay conditions are described in the Experimental Section using lumacaftor or **5** (3 μM) as the positive control. All values are the geometric mean calculated from at least two runs, except when mentioned.

b Only one measure was done.

c CSE-HRP, cell surface expression-horse radish peroxidase.

Maintaining a highly polar carboxylic acid functionality at the C6 position was found to be critical for maintaining activity. Most analogues, including those with a methyl ketone (**29**), methyl hydroxyl (**30**), ethyl hydroxyl (**31**), propan-2-ol (**32**), trifluoro-ethylamide (**33**), and 5-methoxy-pyrazole (**34**) substituent, resulted in substantially compromised activity. Further, homologation of the carboxylic acid (**35**) was detrimental for both efficacy and potency, highlighting the importance of correctly positioning the negative charge and the orientation of the associated dipole for retention of activity.

Analogues with an acidic proton at the C6 position offered bioisosteric alternatives to the carboxylic acid functional group,^14^ for example, an acidic cyanamide (**36**) and acylsulfonamide moieties (**37** and **38**) were shown to have good activity, particularly in comparison with the other analogues.^14^ Of these, the most interesting bioisosteres to the carboxylic acid were the acylsulfonamides, which either maintained (**37**) or improved (**38**) efficacy and potency.

This exploration determined that amides were generally not tolerated at the C6 position and that elongated analogues resulted in lack of activity. In addition, the heterocyclic bioisostere (**34**) did not retain potency. Thus, the acylsulfonamide was selected moving forward in the SAR campaign.

**Figure 2:**
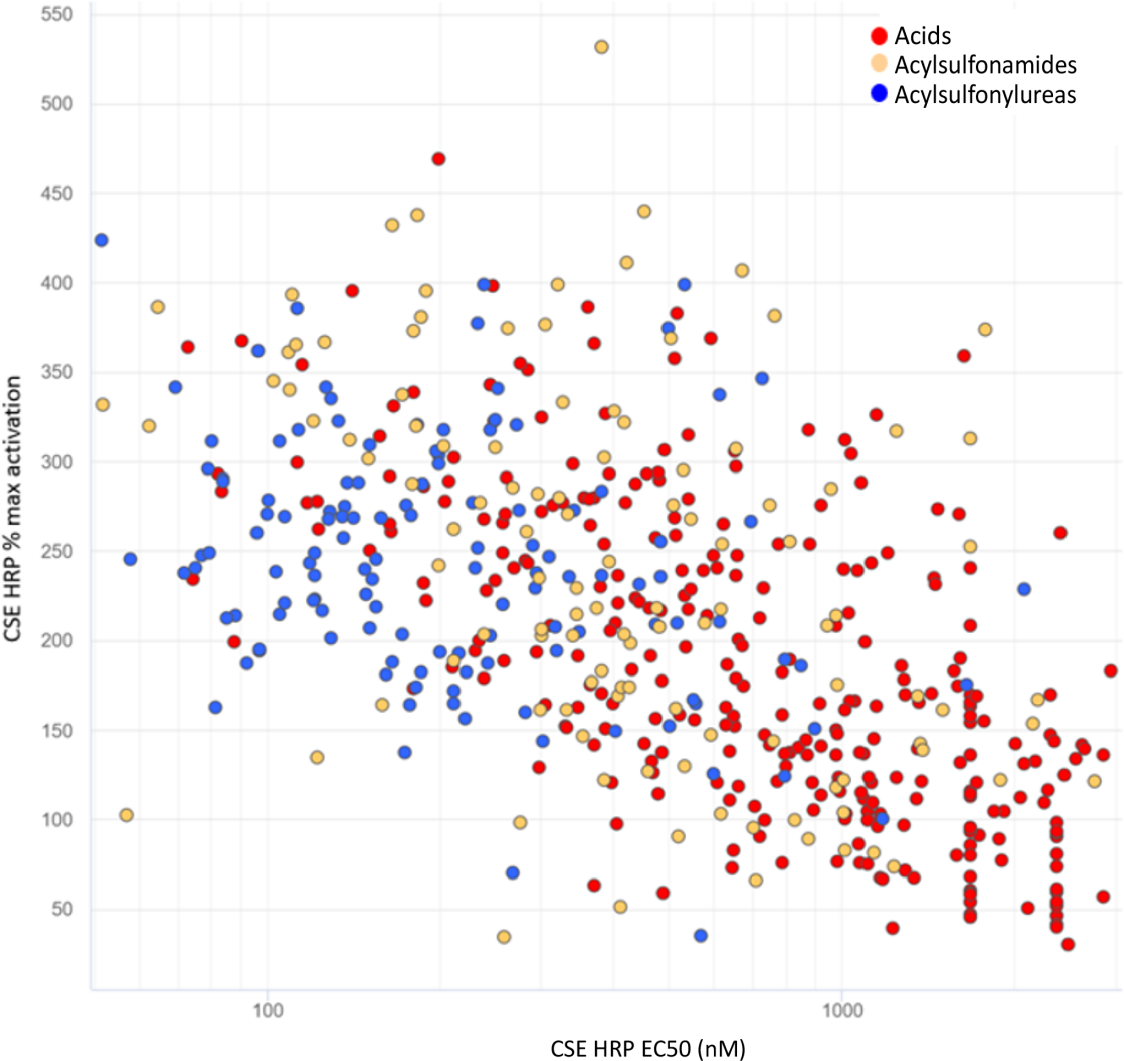
Plot of CSE HRP potency (*X*-axis, EC_50_ [nM]) and CSE HRP efficacy (*Y*-axis, % max activation) for acid (red), acylsulfonylurea (blue), and acylsulfonamide (yellow) analogues.

The extensive SAR effort during the project showed that an increase in potency was generally accompanied by an increase in efficacy (Figure 2). However, a lack of good correlation between potency and efficacy was observed for the carboxylic acids and the sulfonamides. *It is worth mentioning that a similar observation was noticed in the case of acylsulfonylureas derivatives, which will be presented later in the discussion.* Thus, medicinal chemistry efforts shifted toward molecular matched pair optimizations to understand the effect of these structural modifications better. This was done using the newly identified acylsulfonamide group and undertaking molecular matched pair analysis with the parent carboxylic acids (Table 6). Here, the more favorable phenyl and cyclobutyl substituents at the N1 and C3 positions, respectively, were maintained. For further SAR understanding, the analogues were also evaluated for their suitability as co-correctors in the presence of an established corrector (**5**; Figure 1). Carboxylic acids and acylsulfonamides behaved similarly in ΔF508 cell surface expression without an additional corrector; however, in the presence of a corrector, acylsulfonamides showed slightly greater efficacy than carboxylic acids for three molecular matched pairs and showed higher potency for all matched pairs. The 4-cyano-piperidine pyridine moiety was thus confirmed to be one of the most favorable C4 substitutions (**44** and **45**).

**Table 6:**
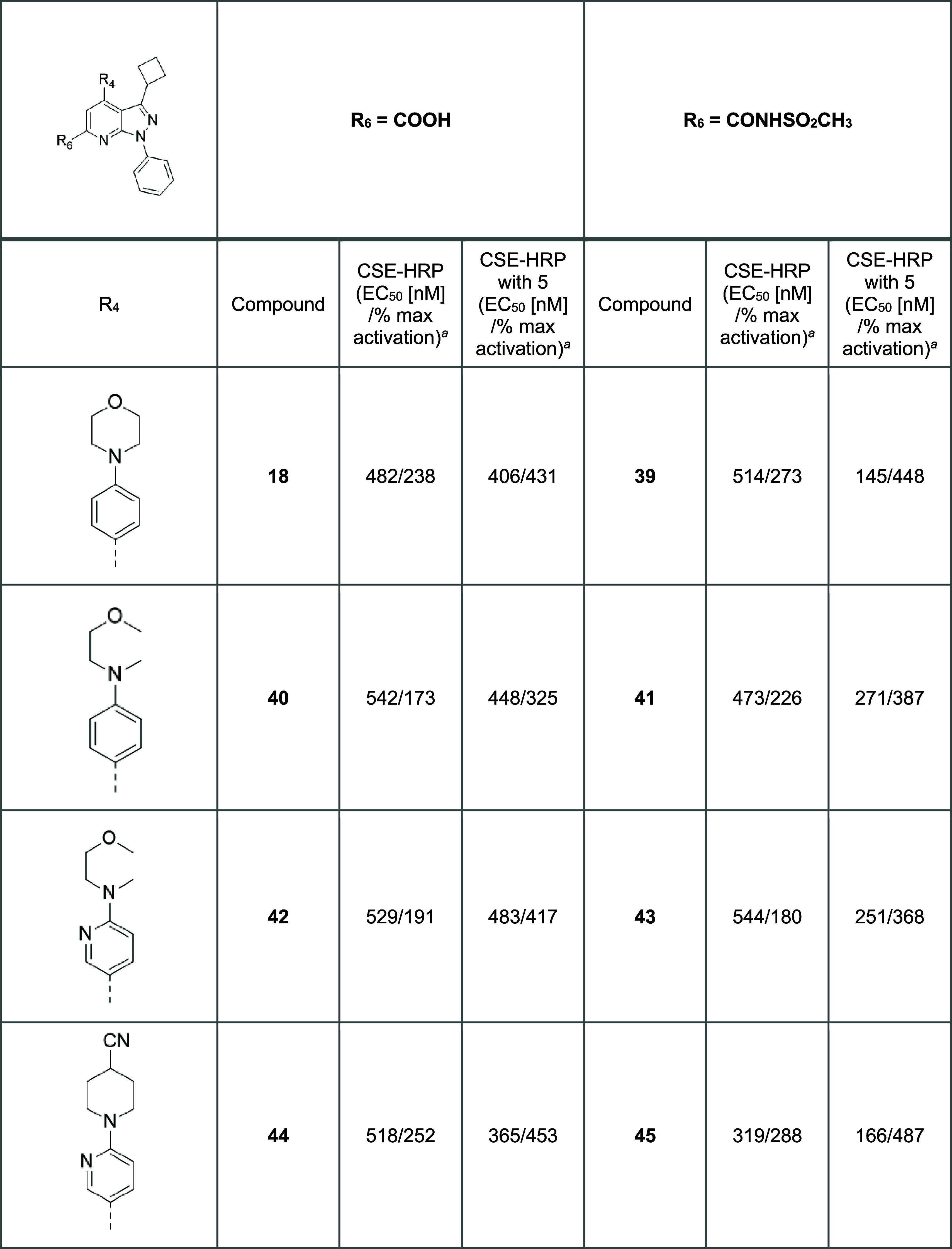
Molecular Matched Pair Analysis of Acylsulfonamides with the Parent Carboxylic Acids^b^

a Assay conditions are described in the Experimental Section using lumacaftor or **5** (3 μM) as the positive control. All values are the geometric mean calculated from at least two runs.

b CSE-HRP, cell surface expression-horse radish peroxidase.

### Pharmacokinetic Analysis

Carboxylic acid **18** and acylsulfonamide **45** were evaluated in rat PK studies and showed low plasma clearance (0.2 and 0.0246 L/h/g, respectively) following iv dosing, partly due to their very high plasma protein binding (PPB) (Table 7). The high PPB measured in rats and humans for these two compounds limited their distribution (Vss = 0.2 L/kg), as generally observed for acidic molecules.^15^**45** showed high oral bioavailability in rats following 5 mg/kg dosing.

**Table 7:** ADME and Rat Pharmacokinetic Parameters^a^

	CL	Vss	PPB% (rat/human)	F (%)
**18**	0.2	0.474	100/99	−
**45**	0.0246	0.195	99.97/99.65	63.4

a Dose 1 mg/kg iv. CL: clearance (L/h/kg). Vss (L/kg). Dose 5 mg/kg po.

Although the compounds containing a carboxylic acid or an acylsulfonamide showed comparable PK profiles, we considered the moderate but consistent potency and efficacy gains obtained for acylsulfonamide derivatives over carboxylic acids in the presence of a C1 corrector significant enough to engage further SAR exploration while retaining the acylsulfonamide group. Representative compound **45** was profiled in CYP induction and inhibition assays to examine potential drug–drug interactions that may cause safety concerns and limit real-world use. No CYP-mediated PK drug–drug interactions that could limit possible combinations with future partners for triple therapy were observed. On all cytochrome P450 subtypes except 2C9, **45** did not show an IC_50_ below 50 μM. In the case of 2C9, the IC_50_ was 0.187 μM and was not considered a liability considering the metabolic profile of the putative potentiator/corrector combination candidates. **45** exhibited low CYP3A4 induction compared with the positive control rifampicin and was devoid of mechanism-based inactivation of the CYP3A4 enzyme.

Starting from this compound, the next investigations consisted of maintaining potency and efficacy while increasing the volume of distribution to favor the drug distribution to the targeted lung tissues (Table 8). Although the high PPB is not a limitation *per se*, we aimed at identifying compounds with slightly reduced PPB, in a range allowing a reproducible and accurate measurement favoring human dose prediction calculations.^16^

**Table 8:**
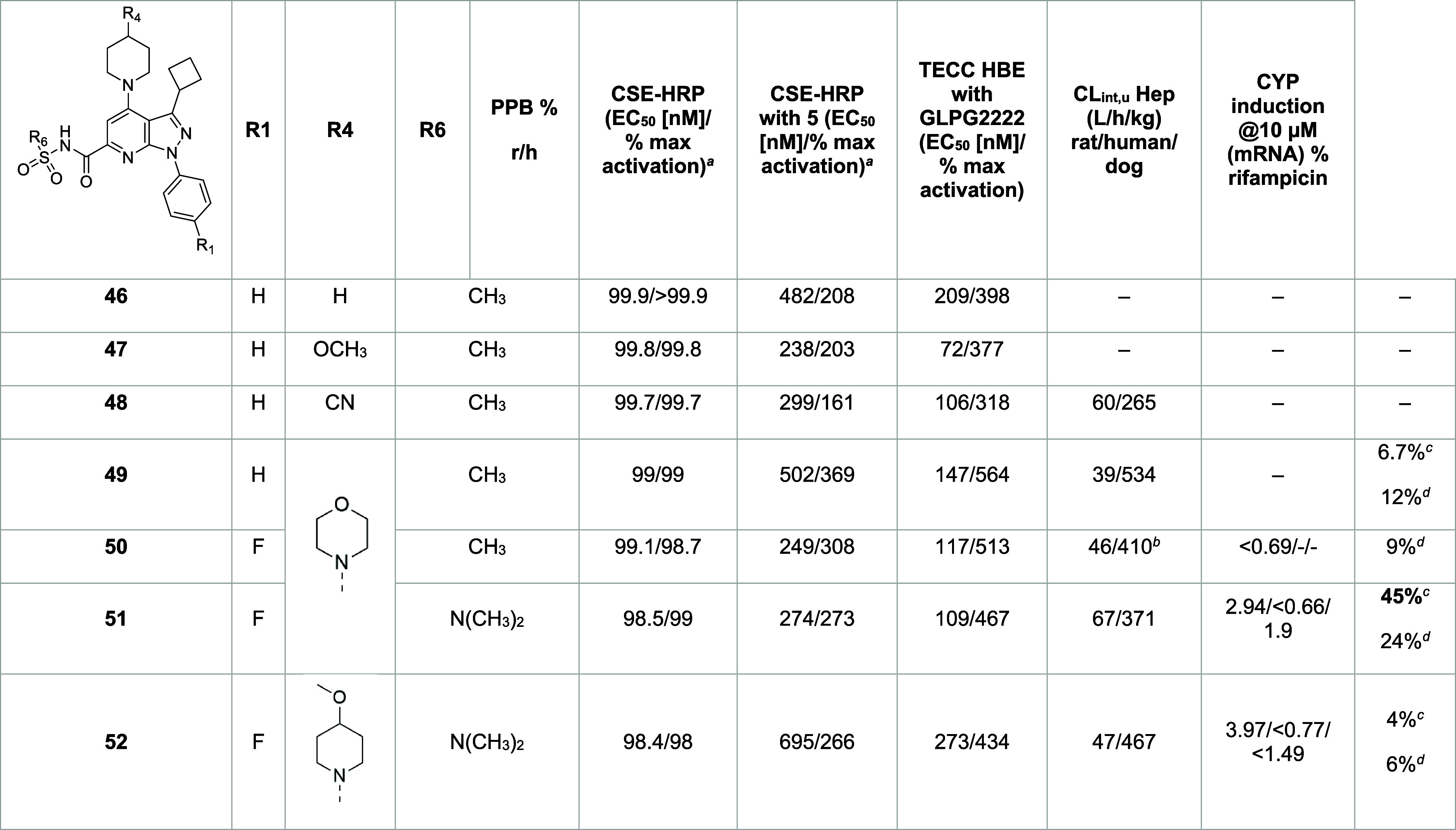
SAR Exploration from Hit 46 at Three Sites^e^

a Assay conditions are described in the Experimental Section using **5** (3 μM) as the positive control. All values are the geometric mean calculated from at least two runs, except when mentioned.

b In contrast to the other TECC experiments, after the 24 h incubation with a range of different concentrations, a fixed 1 μM C2 corrector concentration was added during the electrophysiological recording.

c Data generated by AbbVie team.

d Data generated by Galapagos.

e CSE-HRP, cell surface expression-horse radish peroxidase; HBE, human bronchial epithelial; TECC, transepithelial clamp circuit.

Acidic molecules such as carboxylic acid and acylsulfonamide derivatives tend to be generally highly bound to plasma albumin and possess low membrane affinity, which restricts their tissue distribution.^15^ Basic molecules bind to α1 acid glycoprotein and albumin in plasma and owing to their positive charge at physiological pH, they tend to partition into phospholipid membranes, owing to the interactions with anionic phospholipid head groups. This translates generally into moderate to high volumes of distribution for basic drugs (>3 L/kg).^15^ These considerations led us to investigate modifications at the C4 position of the ring to drastically impact the physicochemical properties while retaining the biological activity.^17^

Removal of the aromatic moiety with the direct linkage of a nonsubstituted piperidine (**46**) reduced the lipophilicity, the number of aromatic counts, and the molecular weight. This modification was tolerated in terms of potency but had no impact on the PPB, probably due to the limited change in the p*K*_a_ of the molecule (acidic p*K*_a_ = 5.16 vs 4.82 for **46** and **45**, respectively, Table 9). Similarly, although the introduction of methoxy or cyano substituents on the piperidine moiety (**47** and **48**, respectively) had a marked effect on physicochemical properties while retaining the potency, the very high PPB remained unchanged compared with the initial **45** derivative, likely due to the modest change in the acid p*K*_a_. Gratifyingly, the introduction of a basic morpholine moiety (**49**) led to a PPB within the acceptable range (99%), with the retention of high potency and efficacy in the absence and presence of a corrector. The increased expression of ΔF508 CFTR at the epithelial cell surface compared with the initial Hit also translated into an increased functional rescue when using the transepithelial clamp circuit (TECC) assay, in terms of both potency (**EC**_**50**_ = 39 nM) and efficacy (534%). **49** was devoid of any CYP induction liability but showed a low volume of distribution in rat and dog. The close analogue with a fluoro atom in *para* of the pending phenyl ring in the N1 position (**50**) retained most of the target profile characteristics, confirming the lower PPB and good biological activity. Despite the introduction of the basic morpholine moiety (basic p*K*_a_ = 7.01, Table 9), the impact on the volume of distribution remained low (Vss = 0.4 L/kg for **49** and Vss = 0.47 L/kg for **50**, Table S1). Modifications to reduce the acidic character of the acylsulfonamide were then investigated. To this end, the acylsulfonamide group was replaced by a less acidic acylsulfonylurea moiety (**51**), as it was reported to be 1 log unit less acidic in studies analyzing the structure–property relationship of carboxylic acid isosteres (p*K*_a_ = 5.86 vs p*K*_a_ = 4.94 in the case of the phenylpropionic acid derivatives).^18^ We calculated the acidic and basic p*K*_a_ values for several analogues using Simul Plus (Table 9).

**Table 9:** Most Acidic and Basic p*K*_a_ Values^a^

	XlogP3	TPSA	acidic p*K*_a_^a^	most basic p*K*_a_^a^
**45**	4.02	142	4.82	3.93
**46**	3.76	106	5.16	3.13
**47**	3.32	115	5.13	3.21
**48**	3.13	129	5.00	2.94
**49**	3.13	118	5.1	7.04
**50**	3.23	118	4.9	7.01
**51**	3.14	121	4.57	6.96
**52**	3.92	121	4.78	7.96

a Calculated with Simul Plus.

However, we observed almost no difference between the calculated p*K*_a_ of the two acid bioisosters, with even a slightly more acidic p*K*_a_ for the acylsulfonamyl derivative (**51** vs **50**). This structural change was well tolerated and almost neutral in terms of potency, in ΔF508 cell surface expression, in functional TECC assays, in physicochemical properties, and in PPB. Surprisingly, it resulted in a marked increase in the volume of distribution in rat and dog, to 0.81 and 0.9 L/kg, respectively (Table 10).

**Table 10:** Rat and Dog PK Data^a^

	CL_p_ (L/h/kg) rat/dog	CL_u_ (L/h/kg) rat/dog	*t*_1/2 iv_ (h) rat/dog	Vss (L/kg) rat/dog	PPB (%) rat/dog/human
**51**	0.19/0.16	13.2/12.1	3.6/4.9	0.81/0.9	98.5/99.0/98.7
**52**	0.32/0.21	20.0/10.7	3.45/5.4	1.38/1.37	98.4/97.5/98.0

a CL_p_, plasma clearance; CL_u_, unbound clearance; *t*_1/2 iv_, half-life following iv dosing; Vss, volume of distribution at steady state; PPB, plasma protein binding.

**51** showed a low plasma clearance in rat and dog after iv administration (Table 10). However, **51** demonstrated CYP mRNA induction, precluding further progression (Table S2). Replacement of the morpholine in **51** by a more basic 4-methoxy-piperidine substituent was calculated to increase the p*K*_a_ by 1 log unit (Table 9; 51 p*K*_a_ = 6.96 vs **52** p*K*_a_ = 7.96). This modification only slightly reduced the potency on the ΔF508 cell surface expression assay, while it delivered one of the most potent and efficacious analogues, **52**, in the TECC assay. In addition, **52** was devoid of any CYP mRNA induction liability. **52** showed similar low total plasma clearance, moderate unbound plasma clearance, and a desired increase and large volume of distribution in rat and dog (Vss = 1.38 in rat and Vss = 1.37 in dog, Table 10).

In the course of our investigations, we observed that the coaddition of correctors from the pyrazolopyridine series to various reported potentiators can inhibit ΔF508 and wild type CFTR gating.^10^ The inhibition of WT CFTR gating translated into an increased need of potentiator level when rescuing ΔF508 CFTR. Although the triple combination therapy might result in a remarkable gain of ΔF508 CFTR function, we considered that the dose levels of each component needed for optimal ΔF508 rescue should be as low as possible to avoid any potential safety limitations. Therefore, a maximum of 5-fold more potentiator (compared to the level of potentiator required for a single corrector **5**) was set as the acceptable corrector-induced shift in the potentiator gating activity. Advanced compounds of the series were evaluated using a single concentration in a functional ΔF508 TECC assay to evaluate the impact on the potency of the potentiator GLPG1837 (Table 11).

**Table 11:** Potency of Potentiator GLPG1837 (**1**) in the Presence of C2 Correctors (1 μM) + C1 Corrector GLPG2222 (**4**) (0.15 μM) in a TECC Assay

C2 corrector	pEC_50_	EC_50_ (nM)	fold
–	7.9 ± 0.18 (*n* = 6)	13	1
**45**	6.8 (*n* = 1)	154	11.8
**49**	6.9 ± 0.10 (*n* = 5)	119	9.2
**51**	7.2 ± 0.09 (*n* = 3)	62^a^	4.8
**52** (GLPG2737)	7.2 ± 0.08 (*n* = 5)	71^a^	5.5

a The differences in the reported values are due to the rounding of data on the logarithmic scale.

**Figure 3:**
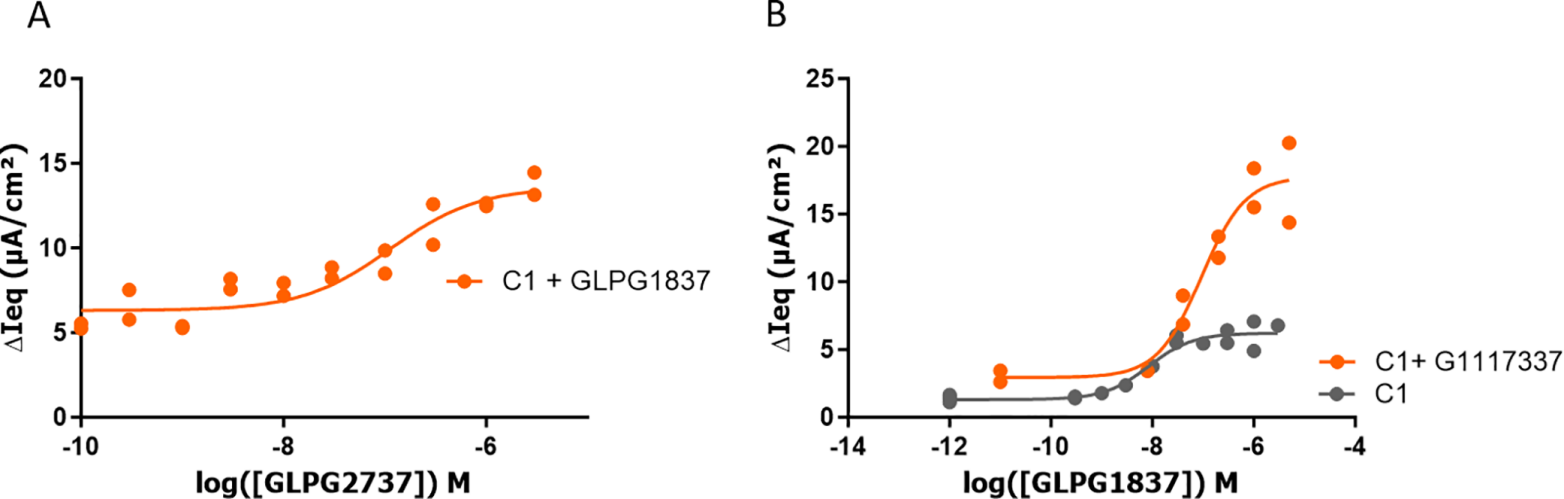
TECC current measurement assay in primary CF derived HBE cells (current induced after FSK stimulation after 24 h of incubation with correctors and potentiators). (A) Dose response of GLPG2737 in combination with 1 μM potentiator GLPG1837 and 0.15 μM corrector **4**. (B) Comparison of a dose response for GLPG1837 in combination with 1 μM compound **52** + 0.15 μM C1 corrector **4** or only corrector **4**.

The potentiator shift induced by the corrector was calculated as the ratio of the potentiator potency observed in the triple combination to the potency measured with corrector **5** alone. As shown in Table 10, the two acylsulfonylurea derivatives (**51** and **52**) showed a moderate and acceptable shift in potentiator potency, while the acylsulfonamide analogues **45** and **49** shifted the potentiator activity by 1 log unit. Altogether, the subtle change from the acylsulfonamide to the acylsulfonylurea group brought an improved volume of distribution combined with an acceptable potency shift. Although the acylsulfonylurea moiety is not very common within the chemical structures of the pharmacopeia, beclabuvir^19^ being the only marketed drug possessing this group, we considered **52** as an interesting candidate, namely GLPG2737, for further evaluation. As shown in Figure 3A, GLPG2737 was a potent corrector of ΔF508 CFTR in a TECC assay measuring the current induced after FSK stimulation after 24 h of incubation with correctors and potentiators. In a triple combination with 1 μM of the potentiator GLPG1837 and 0.15 μM C1 corrector **4** we observed a potency of 46 nM (see also Table 8). The potentiator shift for GLPG1837 in a triple combination with 1 μM GLPG2737 and 0.15 μM C1 corrector is shown in Figure 3B and was around 5.5-fold (see also Table 11).

GLPG2737 showed high permeability both in MDCKII-MDRI and Caco-2 cells, with low efflux allowing an elevated absorbed fraction FaxFg and a dose proportional increase in exposure in rat from a dose of 5 to 300 mg/kg, despite a low aqueous thermodynamic solubility (Table 12). Experimental determination of **52** acidic and basic p*K*_a_ using both potentiometric and UV methods (4.96 and 4.74, respectively) confirmed the calculated value for the acylsulfonamyl group (4.78) and revealed a slight underestimation of the piperidine basicity by the predictive model.

**Table 12:** GLPG2737 (**52**) ADME Data^a^

MDCK-MDR1: PA2B (cm × 10^–6^ s^–1^)/ER	5.45/4.35 (*n* = 2)
Caco-2: PA2B (cm × 10^–6^ s^–1^)/ER	24.5/1.27 (*n* = 1)
thermodynamic solubility	
FASSGF	10.9 μg/mL
pH 7.4	9.9 μg/mL
measured p*K*_a_: potentiometric method/UV method^b^	
acylsulfonamyl p*K*_a_	4.96/4.74
piperidine p*K*_a_	8.42/8.51
CYP inhibition in HLM	IC_50_ (μM)
CYPlA2 phenacetin	>100
CYP2C19 S-mephenytoin	>33
CYP2C9 diclofenac	1.4
CYP2D6 dextromethorphan	>100
CYP3A4 midazolam	>100
CYP3A4 testosterone	>100

a MDCK-MDR1, permeability assay using Madin-Darby canine kidney cells transfected with MDR1; PA2B, apparent permeability from A side to B side; ER, efflux ratio; Caco-2, permeability assay in human epithelial cell line (colorectal adenocarcinoma cells); FASSGF, fasted stimulated gastric fluid; CYP, cytochrome P450; HLM, human liver microsomes.

b p*K*_a_ values were measured at Charles River.

GLPG2737 was selective for CFTR, as evaluated in a panel of 154 kinase assays and in a panel of 68 receptors, ion channel transporters, and enzyme assays from Eurofins. GLPG2737 was found to only inhibit binding from cognate radioligands to (h)H1 and (h)Sigma with respective IC_50_s determined to be 8 and 0.36 μM. GLPG2737 was not mutagenic, clastogenic, or aneugenic as assessed in an Ames II and *in vitro* micronucleus test. No significant inhibition was obtained in the hERG manual patch clamp test.

GLPG2737 was evaluated in preclinical DMPK (Table S3), safety pharmacology, and toxicology studies in rats and dogs, which supported its selection as a candidate for clinical development.

The safety, tolerability, and PK of single ascending oral doses and multiple ascending oral doses of GLPG2737 were evaluated in a first-in-human phase 1 study (NCT03410979) in healthy male participants. Clinical outcomes supported the progression of GLPG2737 in the phase 2a study PELICAN (NCT03474042). GLPG2737 was well tolerated and demonstrated improved efficacy versus placebo in patients with CF homozygous for ΔF508 who were receiving ivacaftor/lumacaftor.^20^ In a phase 1b study (NCT03540524), GLPG2737 was evaluated in a triple combination with potentiator GLPG2451,^20,21^ and GLPG2222 in patients with CF homozygous for ΔF508; however, the plasma levels of GLPG2451 were not high enough to overcome the effect of GLPG2737 on CFTR gating, resulting in limited benefit for patients. Given the inhibitory activity of GLPG2737 toward wild type CFTR, GLPG2737 could have potential for use in diseases other than CF. Indeed, inhibition of the CFTR channel might reduce cyst growth and kidney enlargement in patients with ADPKD.^22^

### Chemical Synthesis

**Scheme 1:**
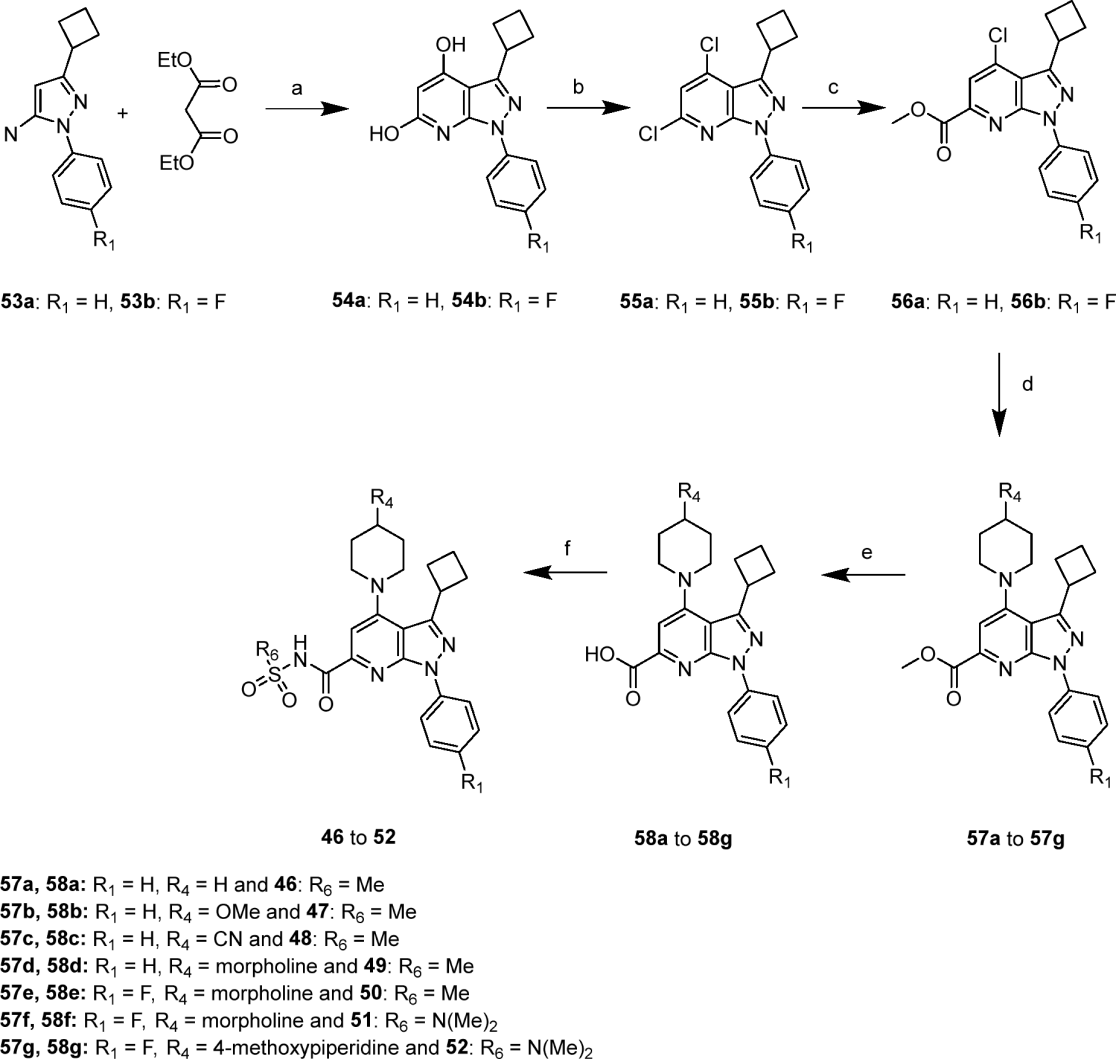
a) 130–170 °C. 3–40 h, (54a: 67%, 54b: 95%); b) Phenyl dichlorophosphate. 170 °C. 15–21 h, (55a: 75%, 55b: 85%); c) CO(g). Pd(dppf)Cl_2_.DCM. sodium acetate. 1,4-dioxane. MeOH. 40–60 °C. 2–40 h, (56a: 58%, 56b: 63%); d) Amine. DIPEA or NEt_3_. MeCN or DMSO. 50–130 °C, (46–94%); e) NaOH or LiOH. H_2_O. MeOH and/or THF or dioxane. rt to 70 °C, (79–100%); f) EDC.HCl. corresponding nucleophile DMAP. DCM or THF or MeCN. rt. 20 h (30–80%) or CDI. DMF corresponding nucleophile. DBU. Rt (**49**: 100%, **52**: 67%).

Here, we detail the preparation and the synthetic route for **52**/GLPG2737, which can also be applied to analogues **46** to **51**. Syntheses and experimental protocols for other derivatives are described in the Supporting Information. GLPG2737 was prepared following a general 6-step synthetic route that is depicted in Scheme 1. The synthesis involved the condensation of 5-aminopyrazole derivatives **53a/53b** with diethyl malonate at 130–170 °C to give the pyrazolo[3.4-b] pyridine-4.6-diol analogues **54a/54b** that were further chlorinated at a high temperature with phenyl dichlorophosphate to afford **55a/55b**. The next carbonylation reaction selectively took place at the C6 position of the scaffold, directing the SNAr reaction with various piperidine analogues at the C4 position. Saponification then coupling of the sulfonamide or sulfonylurea groups afforded GLPG2737 (**52**), as well as the analogues **46**–**51** listed in Scheme 1.

## Conclusion

An HTS campaign using a lung epithelial cell line stably expressing ΔF508 CFTR allowed the identification of a new pyrazolo-pyridine Hit series able to rescue the trafficking of ΔF508 CFTR to the plasma membrane. Optimizing various exit vectors using a CSE assay and progressing through matched pair analysis enabled us to gain an understanding of the SAR components. Combinations of the best moieties led to significant increases in potency and efficacy, which were further enhanced in combination with a corrector. The presence of an acidic moiety was found to be critical for the *in vitro* potency but drastically limited the *in vivo* distribution of the compounds. Further identification of less acidic acylsulfonylureas combined with introduction of basic moieties in the 4-position of the pyrazolopyridine ring contributed to a major improvement in terms of both *in vitro* potency in a functional TECC assay and acceptable PK parameters, leading to the identification of GLPG2737.

## Experimental Section

### General Chemistry Methods

All reagents were of commercial grade and were used as received without further purification, unless otherwise stated. Commercially available anhydrous solvents were used for reactions conducted under an inert atmosphere. Reagent-grade solvents were used in all other cases unless otherwise specified. Flash column chromatography was performed on silica gel 60 (thickness: 35–70 μm). Thin-layer chromatography was carried out using precoated silica gel 60F-254 plates (thickness: 0.25 mm). Celpure P65, a commercial product (61790–53–2), was used as a filtration aid. ^1^H NMR spectra were recorded on a 400 MHz Avance spectrometer (SEI probe) or a 300 MHz DPX Bruker spectrometer (QNP probe). Chemical shifts (δ) for ^1^H NMR spectra are reported in ppm relative to tetramethylsilane (δ 0.00) or the appropriate residual solvent peak (i.e., CHCl_3_ [δ 7.27], as internal reference). Multiplicities are given as singlet (s), doublet (d), triplet (t), quartet (q), quintuplet (quin), multiplet (m), and broad (br). Electrospray MS spectra were obtained using a Waters Acquity UPLC with a Waters Acquity photodiode array detector and a single quad detector mass spectrometer. Columns used were: UPLC Ethylene-Bridged Hybrid (BEH) C18 1.7 μm, 2.1 × 5 mm VanGuard precolumn with Acquity UPLC BEH C18 1.7 μm, 2.1 × 30 mm Column or Acquity UPLC BEH C18 1.7 μm, 2.1 × 50 mm Column. All the methods used MeCN/H_2_O gradients. MeCN and H_2_O contained either 0.1% formic acid or 0.05% NH_3_. The autopurification system from Waters was used for LC-MS purification. LC-MS columns used were Waters XBridge Prep C18 5 μm, ODB 30 mm inner diameter (ID) × 100 mm length (L) (preparative column) and Waters XBridge C18 5 μm, 4.6 mm ID × 100 mm L (analytical column). All the methods used MeCN/H_2_O gradients. MeCN and H_2_O contained either 0.1% formic acid or 0.1% diethylamine. All final compounds reported were analyzed using these analytical methods, and purities were >95% unless otherwise indicated.

### Illustrative Synthesis of GLPG2737: 3-Cyclobutyl-*N*-(*N*,*N*-dimethylsulfamoyl)-1-(4-fluorophenyl)-4-(4-methoxy-[1,4′-bipiperidin]-1′-yl)-1*H*-pyrazolo[3,4-*b*]pyridine-6-carboxamide (**52**)

#### Step a: 3-Cyclobutyl-1-(4-fluorophenyl)-1*H*-pyrazolo[3,4-*b*]pyridine-4,6-diol (**54b**)

A mixture of **53b** (5 g, 18.6 mmol) and diethyl malonate ([105–53–3], 8.5 mL, 55.8 mmol) was heated at 100 °C for 30 min and then at 170 °C for 3 h. The reaction mixture was cooled down to room temperature and dissolved in DCM (60 mL). The resultant solution was poured into a stirred solution of *n*-heptane (700 mL). The precipitate was collected by filtration, washed with *n*-heptane, and dried at 40 °C under reduced pressure to give the title compound 54b (5.29 g, 95% yield). LC-MS: *m*/*z* = 300.3 (M + H)^+^. ^1^H NMR (400 MHz, DMSO) δ 11.35 (s, 1H), 8.31–8.11 (m, 2H), 7.42–7.25 (m, 2H), 5.87 (s, 1H), 3.97–3.84 (m, 1H), 2.46–2.26 (m, 4H), 2.06–1.96 (m, 1H), 1.93–1.80 (m, 1H).

#### Step b: 4,6-Dichloro-3-cyclobutyl-1-(4-fluorophenyl)-1*H*-pyrazolo[3,4-b]pyridine (**55b**)

A three-neck round-bottom flask equipped with a Dean–Stark apparatus was charged with phenyl dichlorophosphate ([770–12–7], 854 g, 4.05 mol). 3-cyclobutyl-1-(4-fluorophenyl)-1*H*-pyrazolo[3,4-*b*]pyridine-4,6-diol **54b** (404 g, 1.35 mol) was added in portions over a period of 5 min. The temperature was increased from room temperature to 170 °C over a period of 1 h, and the stirring at 170 °C was continued for 21 h. The reaction mixture was cooled down to 50 °C and added slowly to a stirred aqueous 4 M NaOH (5 L), keeping the temperature below 20 °C. The suspension was stirred for 1 h at 10–15 °C, and then cold water (3 L) was added. The precipitate was collected by filtration, washed with water, and dried at 40 °C under reduced pressure to give the title compound **55b** (385 g, 85% yield). LC-MS: *m*/*z* = 337.3 (M + H)^+^. ^1^H NMR (400 MHz, CDCl_3_) δ 8.24–8.15 (m, 2H), 7.26–7.19 (m, 2H), 7.18 (s, 1H), 4.23–4.09 (m, 1H), 2.64–2.43 (m, 4H), 2.23–2.08 (m, 1H), 2.08–1.95 (m, 1H).

#### Step c: Methyl 4-Chloro-3-cyclobutyl-1-(4-fluorophenyl)-1*H*-pyrazolo[3,4-*b*]pyridine-6-carboxylate (**56b**)

A pressurized vessel was charged with 4,6-dichloro-3-cyclobutyl-1-(4-fluorophenyl)-1*H*-pyrazolo[3,4-*b*]pyridine **55b** (5 g, 14.9 mmol), Pd(dppf)Cl_2_·DCM ([95464-05-4], 218 mg, 0.3 mmol), and sodium acetate (1.8 g, 22.3 mmol) in 1,4-dioxane/methanol (1:1, 25 mL). The system was loaded with CO (4 bar) and heated at 40 °C for 2 h. The vessel was cooled to room temperature, and the conversion was monitored by LC-MS. The reaction vessel was charged again with CO (4 bar) and heated at 40 °C. The sequence was repeated until full conversion was observed on LC-MS. The crude mixture was concentrated under reduced pressure and purified by flash column chromatography eluting with a mixture of *n*-heptane/DCM (90/10 to 30/70) to give the title compound **56b** (3.38 g, 63% yield). LC-MS: *m*/*z* = 360.2 (M + H)^+^. ^1^H NMR (400 MHz, DMSO) δ 8.31–8.17 (m, 2H), 7.94 (s, 1H), 7.53–7.40 (m, 2H), 4.25–4.10 (m, 1H), 3.95 (s, 3H), 2.49–2.41 (m, 4H), 2.19–2.04 (m, 1H), 2.01–1.87 (m, 1H).

#### Step d: Methyl 3-Cyclobutyl-1-(4-fluorophenyl)-4-(4-methoxy-[1,4′-bipiperidin]-1′-yl)-1*H*-pyrazolo[3,4-*b*]pyridine-6-carboxylate (**57f**)

To a solution of **56b** (1 g, 2.78 mmol) in DMSO (10 mL) was added 4-methoxy-1,4′-bipiperidine (1.5 g, 5.56 mmol) and triethylamine (1.55 g, 11.12 mmol). The reaction mixture was stirred at 100 °C for 24 h and then cooled down to ambient temperature diluted with water (100 mL). The suspension was stirred for 20 h, filtered and was washed with water. The precipitate was dried at 40 °C under reduced pressure to afford the title compound **57f** (670 mg, 46%) LCMS: *m*/*z* = 522.4 (M + H)^+^. ^1^H NMR (400 MHz, DMSO-*d*_6_) δ 8.34–8.25 (m, 2H), 7.42 (t, J = 8.8 Hz, 2H), 7.29 (s, 1H), 4.04–3.93 (m, 1H), 3.91 (s, 3H), 3.60 (d, J = 12.1 Hz, 2H), 3.24 (s, 3H), 3.21–3.13 (m, 1H), 2.92 (t, J = 11.9 Hz, 2H), 2.87–2.77 (m, 2H), 2.46–2.36 (m, 4H), 2.35–2.22 (m, 3H), 2.14–1.81 (m, 6H), 1.78–1.61 (m, 2H), 1.49–1.34 (m, 2H).

#### Step e: 3-Cyclobutyl-1-(4-fluorophenyl)-4-(4-methoxy-[1,4′-bipiperidin]-1′-yl)-1H-pyrazolo[3,4-*b*]pyridine-6-carboxylic acid (**58g**)

Methyl 3-cyclobutyl-1-(4-fluorophenyl)-4-(4-methoxy-[1,4′-bipiperidin]-1′-yl)-1H-pyrazolo[3,4-*b*]pyridine-6-carboxylate **57f** (12.7 g, 22.58 mmol, 1 equiv) and lithium hydroxide monohydrate ([1310–66–3] 1.9 g, 45.16 mmol, 2 equiv) in a mixture of 1,4-dioxane/H_2_O (200 mL [2:1]) were heated at 50 °C for 1 h. The reaction mixture was cooled down to room temperature, and the volatiles were removed *in vacuo*. The residue was diluted with H_2_O and acidified to pH 5 with aqueous 1 M HCl. The precipitate was filtered, washed with water, and dried at 40 °C under reduced pressure to afford the title product **58f** (9.7 g, 79% yield). LC-MS: *m*/*z* = 508.0 (M + H)^+^. ^1^H NMR (400 MHz, CDCl_3_) δ 12.33–12.23 (m, 1H), 7.94 (m, 2H), 7.15 (m, 2H), 3.83 (p, J = 8.2 Hz, 1H), 3.74 (d, J = 12.6 Hz, 2H), 3.59 (s, 1H), 3.34–3.22 (m, 6H), 3.13 (q, J = 11.5 Hz, 2H), 2.97 (t, J = 12.3 Hz, 2H), 2.60–2.30 (m, 9H), 2.15–1.95 (m, 6H).

#### Step f: 3-Cyclobutyl-*N*-(*N*,*N*-dimethylsulfamoyl)-1-(4-fluorophenyl)-4-(4-methoxy-[1,4′-bipiperidin]-1′-yl)-1H-pyrazolo[3,4-*b*]pyridine-6-carboxamide (**52**) or GLPG2737

A round-bottom flask was charged with 3-cyclobutyl-1-(4-fluorophenyl)-4-(4-methoxy-[1,4′-bipiperidin]-1′-yl)-1*H*-pyrazolo[3,4-*b*]pyridine-6-carboxylic acid **58f** (7.1 g, 13.94 mmol, 1 equiv) and dry DMF (100 mL). 1,1′-Carbonyldiimidazole (5.42 g, 33.46 mmol, 2.4 equiv) was added in one portion and the mixture was stirred at room temperature for 30 min. *N*,*N*-Dimethylsulfamide (3.46 g, 27.88 mmol, 2.0 equiv) was added, followed by 1,8-diazabicyclo[5.4.0]undec-7-ene (5 mL, 33.46 mmol, 2.4 equiv). The mixture was stirred for 1 h and then poured onto H_2_O (500 mL). The solution was acidified with 1 M citric acid until a persistent precipitate appeared. The precipitate was filtered, washed with water, and dried *in vacuo* at 40 °C. The crude material was purified by flash column chromatography eluting with DCM/MeOH to afford the title product **52** (5.71 g, 67% yield). LC-MS: *m*/*z* = 614.5 (M + H)^+^. ^1^H NMR (400 MHz, CDCl_3_) δ 8.07–7.98 (m, 2H), 7.46 (s, 1H), 7.32–7.21 (m, 2H), 4.00 (p, J = 8.4 Hz, 1H), 3.73 (d, J = 12.2 Hz, 2H), 3.39 (s, 3H), 3.34–3.24 (m, 1H), 3.06 (s, 6H), 3.02–2.87 (m, 4H), 2.62 (dq, J = 11.5, 9.0 Hz, 2H), 2.55–2.35 (m, 5H), 2.24–2.03 (m, 5H), 1.97 (s, 2H), 1.91–1.77 (m, 2H), 1.75–1.60 (m, 2H).

### Cell Culture

A CFBE41o– cell line stably expressing ΔF508 CFTR harboring an HRP-tag in the fourth extracellular loop was obtained from Professor Gergely Lukacs (Department of Physiology, McGill University, Montreal, QC, Canada).^25^ Cells were grown in Eagle’s minimal essential medium (Life Technologies) supplemented with 10% FBS, 1% l-glutamine (Life Technologies), 10 mM HEPES (Life Technologies), 200 μg/mL Geneticin (Life Technologies), and 3 μg/mL puromycin (Sigma) in culture flasks coated with 0.01% bovine serum albumin (BSA) (Sigma), 30 μg/mL Purecol (Nutacon), and 0.001% human fibronectin (Sigma).

Bronchial epithelial cells isolated from the lungs of patients with CF homozygous for the ΔF508 CFTR mutation were obtained from McGill University (Montreal, QC, Canada) or the University of North Carolina (Chapel Hill, NC, United States). Cells were isolated from lungs obtained from donors undergoing planned transplantation. These primary cells were cultured directly on type IV collagen-coated polycarbonate Transwell supports with a diameter of 6.5 mm and pore size of 0.4 μm (Costar, #3397) for 18–25 days in an air–liquid interface, essentially as previously described for TECC.^23^

### Cell Surface Expression Horseradish Peroxidase Assay (CSE-HRP Assay)

CFBE41o– TetON cells expressing HRP tagged ΔF508-CFTR were seeded in white 384-well plates (Greiner) at a density of 2000 cells per well. Medium containing 500 ng/mL doxycycline was used to induce expression of ΔF508-CFTR-HRP. After 3 days, cells were treated with corrector or potentiator compounds and transferred to an incubator at 33 °C. On day 4, cells were washed five times with PBS containing Ca^2+^ and Mg^2+^ using a Bio-Tek plate washer and incubated with a chemiluminescent HRP substrate (SuperSignal West Pico Chemiluminescent Substrate, Thermo Scientific) for 15 min. Chemiluminescence was measured using an Envision plate reader (PerkinElmer). Dose response data was fitted using a 4 parameter hill function of the form Response = neg control + (pos control – neg control)/(1 + 10^(log **EC_50_**-concentration)/HillSlope)^ to determine **EC_50_** values. Percentage efficacy was calculated using the following formula: (max response – neg control)/(C1 response – neg control).

### Transepithelial Clamp Circuit (TECC)

Twenty-four hours prior to the electrophysiological recording, corrector and/or potentiator compounds were added on both the apical and basolateral sides. TECC recordings were performed by using the TECC instrument developed and sold by EP Design (Bertem, Belgium). During the recording, the epithelial cells were bathed in a NaCl-Ringer solution (120 mM NaCl, 20 mM HEPES, 1.2 mM CaCl_2_, 1.2 mM MgCl_2_, 0.8 mM KH_2_PO_4_, 0.8 mM K_2_HPO_4_, 5 mM glucose, pH 7.4) on both the basolateral (640 μL) and the apical (160 μL) sides and kept at 37 °C. Corrector and potentiator compounds were readded at the same concentration used during the 24 h pretreatment. Apical amiloride was used to inhibit the endogenous ENaC currents (100 μM), while forskolin (10 μM) was applied on both the apical and basolateral sides to stimulate CFTR. Measurements were done during a 20 min time frame with recordings every 2 min. The transepithelial potential and transepithelial resistance were measured in an open circuit and transformed to Ieq using Ohm’s law. The maximal increase in Ieq (ΔIeq, the difference in current before and after forskolin or potentiator treatment) was used as a measure for the increased CFTR activity. **EC**_**50**_ values were generated by measuring the impact of different concentrations of compounds on ΔIeq in primary cells. For this purpose, each transwell was treated with a different compound concentration. CFTRInh-172 was added apically at 10 μM to assess the specificity of the response. Dose response data was fitted using a 3-parameter hill function of the form Response = Bottom + (Top – Bottom)/(1 + (**EC**_**50**_/concentration)) to determine **EC**_**50**_ values. Percentage efficacy was calculated using the following formula: (max response – neg control)/(C1 response – neg control).

### Liver Microsomal Stability (LMS) Assay

The microsomal stability assay was performed by incubation of test compound at 1 μM, 0.2% DMSO in phosphate buffer with microsomes (0.5 mg/mL) from mouse, rat, dog, or human (Xeno-Tech, Kansas City, KS, USA), and cofactors with final concentrations of 0.6 U/mL glucose-6- phosphate dehydrogenase, 3.3 mM MgCl_2_, 3.3 mM glucose-6-phosphate, and 1.3 mM nicotinamide adenine dinucleotide phosphate (NADP)+. Before addition of the microsomes (time zero) and after 30 min of incubation at 37 °C with shaking, the reaction was stopped, and proteins were precipitated with an excess of acetonitrile containing an internal standard. The samples were mixed, centrifuged, and filtered, and the supernatant was analyzed by liquid chromatography–mass spectrometry/mass spectrometry (LC–MS/MS). The instrument responses (peak areas/IS peak areas) were referenced to the zero-time point samples (considered as 100%) to determine the percentage of compound remaining. *In vitro* unbound intrinsic clearance (CL_int,u_) was calculated from the half-life using the following equations:
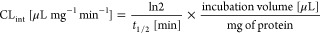

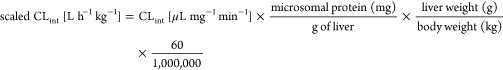
To take into account nonspecific binding, scaled CL_int_ values were corrected with the fraction unbound in microsomes (fu, mic).
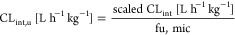


### Fu, mic (Fraction Unbound in Microsomes)

Equilibrium dialysis is a technique used to measure microsomal binding. Briefly, the assay was performed in a 96-well Teflon dialysis unit (Dialysis Device), where each well consists of two chambers separated by a dialysis membrane (membrane strips, MW cutoff 12–14 kDa, HTDialysis). Inactivated liver microsomes (pooled human liver microsomes, Xenotech, protein concentration of 0.5 mg/mL) spiked with a compound (1 μM final concentration, 0.5% DMSO) were added to one chamber and buffer solution (50 mM PBS Buffer) was added to the other side of the well. Each compound was analyzed in duplicate for 4 h at 37 °C. At the end of incubation, both chambers were sampled and analyzed by LC-MS/MS. The unbound microsomal fraction (fu, mic) was calculated as the concentration in buffer divided by the total concentration in the microsomal side. Positive controls included in this assay are terfenadine and verapamil.

### Hepatocyte Stability Assay

The hepatocyte stability assay was performed by incubation of test compound at 1 μM, 0.03% DMSO in modified Krebs–Henseleit buffer with suspension of pooled cryopreserved hepatocytes (BioIVT, Hicksville, NY, USA) from mouse, rat, dog, or human (BioIVT, Hicksville, NY, USA) at 0.5 million viable hepatocytes/mL. Before adding the hepatocytes (time zero) and after 10, 20, 45, 90, 120, and 180 min of incubation (*n* = 2) at 37 °C while gently shaking, the reaction was stopped and proteins were precipitated with an excess of acetonitrile containing an internal standard. The samples were mixed, centrifuged, and filtered, and the supernatant was analyzed by liquid chromatography–mass spectrometry/mass spectrometry (LC–MS/MS). The instrument responses (peak areas/IS peak areas) were referenced to the zero-time point samples (considered as 100%) in order to determine the percentage of compound remaining. *In vitro* unbound intrinsic clearance (CL_int,u_) was calculated from the half-life using the following equations:
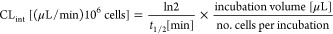

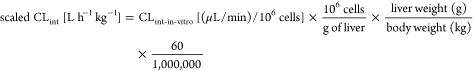
To take into account nonspecific binding, scaled CL_int_ values were corrected with the fraction unbound in microsomes (fu, mic), which was adapted to take into account the environment of the hepatocytes (fu, inc = 1/((1+(10∧((log10(((1-fu, mic)/fu, mic))-0.06)/1.52)))))^24^) according to the equation below
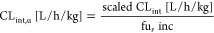


### Plasma Protein Binding

Plasma protein binding was determined by equilibrium dialysis using the Pierce Red Device plate with inserts (ThermoScientific). Test compound at 5 μM (0.5% DMSO) spiked in freshly thawed human, rat, mouse, or dog plasma (Bioreclamation INC, Westbury, NY, USA) was dialyzed against phosphate-buffered saline (PBS, pH 7.4) at 37 °C under shaking for 4 h. Aliquots were taken from each side of the well, and matrix matched. Proteins were precipitated with an excess of acetonitrile, samples were mixed and centrifuged, and the supernatant was analyzed by liquid chromatography–mass spectrometry/mass spectrometry (LC–MS/MS). Addition of compound peak areas in the buffer chamber and the plasma chamber was considered to be 100% compound. The percentage bound to plasma proteins was derived from these results with the formula = *x* – % bound 100 peak area of plasma peak area of buffer peak area of plasma.

### PK Rat

These studies were performed with naïve male Sprague–Dawley rats (Janvier France, 6–8 weeks old). Rats were dosed iv via a bolus in the tail vein with a dose level of 0.1 mg/kg or orally as a single esophageal gavage with a dose level of 5 mg/kg. For the iv route, the compound was formulated in PEG200/water (60/40; v/v). For the oral route, the compound was formulated in MC 0.5% (2/98) as a homogeneous suspension. Before the oral dosing, the animals were deprived of food for at least 12 h before compound administration until 3 h after administration. All animals had free access to the tap water. Blood samples were collected under light anesthesia and placed into tubes containing Li-heparin as an anticoagulant. Blood samples were collected up to 24 h after dosing (*n* = 3 rats per route). After centrifugation, the resulting plasma samples were assayed by LC-MS/MS with a nongood laboratory practice (non-GLP)-validated method. PK parameters were calculated by noncompartmental analysis using WinNonlin software (Certara, Princeton, NJ, USA).

### CYP3A4 Induction

Cryopreserved human hepatocytes from a single donor were seeded at 0.1 × 10^6^ cells/well. The next day, cells were dosed with test compound in assay medium (final test compound concentration 10 μM; final DMSO concentration 0.1%). Positive control inducer, rifampicin, for CYP3A4, was incubated alongside the test compound. Negative control wells were included where the test compound is replaced by vehicle solvent (0.1% DMSO in assay medium). Each test or control compound was dosed in triplicate at a single concentration (10 μM). The cells were exposed to the solutions for 72 h with fresh solution added every 24 h. For mRNA assessment, all media was removed from each of the wells, and the cells were washed once. The cells were lysed, and total RNA was then isolated from the hepatocyte lysates. Reverse transcription was performed, and quantitative PCR analysis was performed on the resulting cDNA, using gene-specific primer probe sets for CYP3A4 target cDNA and endogenous control. Samples were analyzed using an ABI 7900 HT real time PCR system. For mRNA assessment, relative fold mRNA expression was determined based on the threshold cycle (CT) data of target gene relative to endogenous control for each reaction and normalized to negative control using the 2-ΔΔCT method. Data were expressed as fold activation relative to the vehicle control and, as a percent, to the 10 μM rifampicin using the following formula: % of positive control = ((fold increase of the cpd relative to vehicle control – 1)/(fold increase of the rifampicin at 10 μM – 1)) × 100.

### MDCK-MDR1 Assay

MDCKII-MDR1 cells were seeded on Millicell-24 cell culture insert plate assemblies at a final concentration of 0.12 × 106 cells/well. Cells were cultured in a CO_2_ incubator for 3–4 days prior to experiment start with media replacement 24 h post seeding. On the day of the experiment, cells were preincubated for 45 min with Dulbecco’s Phosphate Buffer saline (D-PBS, pH7.4), containing 1% of DMSO. Compounds were prepared in D-PBS, pH 7.4 and added to either the apical or basolateral chambers of the Millicell cell culture insert plates assembly at a final concentration of 10 μM with a final DMSO concentration of 1%. Lucifer Yellow was added to all donor buffer solutions in order to assess integrity of the cell monolayers by monitoring Lucifer Yellow permeation. After a 1 h incubation at 37 °C while being shaken, aliquots were taken from both apical (A) and basolateral (B) chambers and added to acetonitrile:water solution (2:1) containing analytical internal standard. Samples were also taken at the beginning of the experiment from donor solutions to obtain the initial (*C*_0_) concentration. After brief mixing and centrifugation, the supernatant was analyzed by LC-MS/MS. The apparent permeability coefficient (*P*_app_) was calculated according to the following equation: *P*_app_ = (d*Q*/d*T*)(1/*C*_0_)(1/*A*), where d*Q*/d*T* = permeability rate; *C*_0_ = initial concentration in donor compartment; *A* = surface area of the cell monolayer (0.7 cm^2^). “Concentration” is the ratio between the compound and internal standard peak areas. The *P*_app_ value has a dimension of a rate (×10^–6^ cm/s). The efflux ratio is calculated as *P*_app_ from B to A divided by *P*_app_ from A to B. Passive permeability (×10^–6^ cm/s) is calculated by the formula: *P*_app_ from A to B × efflux ratio + 1/2.

### Intestinal Permeability on Caco-2 Cells

Caco-2 cells were obtained from the European Collection of Cell Cultures (ECACC) and used after a 21-day cell culture in 24-well Transwell plates. Test compounds and the references (vinblastine and propranolol) were prepared in protein-free Hanks’ balanced salt solution containing 25 mM HEPES (pH 7.4) at a concentration of 10 μM and added to either the apical or basolateral chambers of a Transwell plate assembly. Before the experiment, the integrity of the monolayer was checked by measuring the transepithelial resistance. Lucifer yellow (LY) was added to the donor buffer in all wells to assess the integrity of the cell layers by monitoring LY permeation. As LY cannot freely permeate lipophilic barriers, a high degree of LY transport indicates poor integrity of the cell layer. After 1 h of incubation at 37 °C, aliquots were taken from both apical (A) and basolateral (B) chambers and added to acetonitrile containing analytical internal standard (carbamazepine) in a 96-well plate. Concentrations of the compound in the samples were measured by LC–MS/MS. Apparent permeability (P_app_) coefficients were calculated from the relationship: *P*_app_ = d*Q*/d*t* × 1/*A* × *C*_0_, where *P*_app_ is the apparent permeability coefficient (cm s^–1^), d*Q*/d*t* is the amount of drug permeated per unit of time, *A* is the effective surface area of the artificial membrane exposed to the medium, and *C*_0_ is the initial drug concentration in the donor compartment. The efflux ratios, as an indication of active efflux from the apical cell surface, were calculated using the ratio of *P*_app_(B → A)/*P*_app_(A → B).

### Thermodynamic Solubility

Dry matter of compound was dissolved at 1 mg/mL in buffers at different pH (Fasted simulated gastric fluid and pH 7.4) in glass vials. After 24 h of stirring at room temperature, a sample is taken, centrifuged for 10 min at 10,000 rpm, and filtered. The samples were diluted in duplicate in DMSO (F100 and F10). Then, a final dilution (F100) in 80/20 water/acetonitrile containing the internal standard was used for the LCMS-MS analysis.

A standard curve was made ranging from a 200,000–75 ng/mL stock in DMSO, freshly prepared from dry matter. The standard curve and quality controls were diluted in an F100 in 80/20 water/acetonitrile (with internal standard) and analyzed on LC/MS-MS. The peak areas of the standard curve are plotted in a graph, and a linear or polynomial of the second order equation is used to calculate the unknown concentrations of the test compound.

### CYP Reversible Inhibition Assay

Test compounds were diluted in methanol and then added to mixture containing 50 mM potassium phosphate buffer, pH 7.4, human liver microsomes (BD Gentest) and probe substrate. After prewarming for 5 min at 37 °C, the reaction was started by adding cofactor mix (7.65 mg/mL glucose-6-phosphate, 1.7 mg/mL NADP, 6 U/mL of glucose-6-phosphate dehydrogenase), resulting in seven final concentrations of test compounds in the range 0.14–100 μM (2% methanol). Isoform specific conditions regarding probe substrate and microsomal protein concentrations are available in Table 2. Final concentrations of cofactor mix components were as follows: 1.56 mg/mL glucose-6-phosphate, 0.34 mg/mL NADP, and 1.2 U/mL glucose-6-phosphate dehydrogenase. After incubation at 37 °C, the reaction was terminated with acetonitrile:methanol (2:1) solution with internal standard. Samples were centrifuged, and the supernatant fractions were analyzed by LC-MS/MS. The instrument responses (test compounds and internal standard peak areas) were referenced to those for solvent controls (assumed as 100%) in order to determine the percentage reduction in probe metabolism. Percent of control activity vs concentration plots were generated and fitted using GraphPad Prism software to generate IC_50_.

### PK Dog

These studies were performed with non-naïve male Beagle dogs (age 13.8 months old, origin: Marshall US, North Rose, NY 14516, USA; age 15.8 months old, origin: Harlan; age 17.7 months old, origin: Harlan). Dogs (*n* = 3) were dosed iv via a 10 min infusion via a catheter with a dose level of 1 mg/kg. After a washout of 3 days, they were dosed orally as a single gavage with a dose level of 5 or 1 mg/kg in PEG400/MC 0.5% or MC 0.5%. Before administration by the po route, animals were fasted for a period of at least 12 h before treatment, and food was given just after the 3 h of blood sampling. All animals had free access to tap water. Blood samples were taken without an anesthetic from a jugular or cephalic vein into tubes containing lithium heparin as an anticoagulant. Blood samples were taken up to 24 h after the start of the infusion. After centrifugation, the resulting plasma samples were assayed by LC-MS/MS with a nongood laboratory practice (non-GLP)-validated method. PK parameters were calculated by noncompartmental analysis using WinNonlin software (Certara, Princeton, NJ, USA).

## Supporting Information Available

Supplemental PK tables; general methods for compound synthesis/analysis, general methods for synthesis of intermediates, and experimental procedure for the synthesis of intermediates and compounds **8**–**52**; HPLC traces of compounds **8**–**52** (PDF)
Formula strings (CSV)

## Abbreviations Used

BID: bis in die or twice a day
CDI: carbonyldiimidazole
CF: cystic fibrosis
CFTR: cystic fibrosis transmembrane conductance regulator
CL: clearance
CL_u_: unbound clearance
Cl_int_: intrinsic clearance
CLogP: calculated log partition coefficient (octanol/water)
CO: carbon monoxide
CSE: cell surface expression
CYP: cytochrome P450
DIPEA: diisopropylethylamine
EDC: 1-ethyl-3-(3-dimethylaminopropyl)carbodiimide
F: oral bioavailability
Fa: the fraction of the administered dose that is absorbed into the systemic circulation
Fg: the fraction of the absorbed dose that escapes first-pass metabolism in the liver and reaches the systemic circulation unchanged
FASSGF: Fasted State Simulated Gastric Fluid
FSK: forskolin
HEPES: buffer made from 2-[4-(2-hydroxyethyl)piperazin-1-yl]ethanesulfonic acid
HBE: human bronchial epithelial
HEP: hepatocyte
HLM: human liver microsomes
HRP: horse radish peroxidase
LMS: liver microsomes stability
MC: methyl cellulose
MDCK: Madin-Darby canine kidney
MeCN: acetonitrile
NADP: nicotinamide adenine dinucleotide phosphate
PD: pharmacodynamics
PEG: polyethylene glycol
PO: per os (oral dosing)
p*K*_a_: acid dissociation constant
QD: quaque die once a day
TECC: transepithelial clamp circuit
T1/2: eff, effective half-life (MRT*Vd,ss/CL)
tPSA: total polar surface area
Vd,ss: volume of distribution at steady state
WT: wild type
