# Supplementary material for: Discovery of GLPG2737, a Potent Type 2 Corrector of CFTR for the Treatment of Cystic Fibrosis in Combination with a Potentiator and a Type 1 Co-corrector
Source: J Med Chem. 2024 Mar 25;67(7):5216–32. doi: 10.1021/acs.jmedchem.3c01790 (PMC11017246; doi:10.1021/acs.jmedchem.3c01790)
Supplement: Supplementary file 1 — jm3c01790_si_001.pdf [file jm3c01790_si_001.pdf]

Discovery of GLPG2737, a potent type 2 corrector of CFTR for the treatment of cystic fibrosis in combination with a potentiator and a type 1 co-corrector

Mathieu Pizzonero,† Rhalid Akkari,† Xavier Bock,† Romain Gosmini,*† Elsa De Lemos,† Béranger Duthion,† Gregory Newsome,† Thi-Thu-Trang Mai,† Virginie Roques,† Hélène Jary,† Jean-Michel Lefrancois,† Laetitia Cherel,† Vanessa Quenehen,† Marielle Babel,† Nuria Merayo,† Natacha Bienvenu,† Oscar Mammoliti,‡ Ghjuvanni Coti,‡ Adeline Palisse,‡ Marlon Cowart,§ Anurupa Shresta,§ Stephen Greszler,§ Steven Van Der Plas,‡ Koen Jansen,‡ Pieter Claes,‡ Mia Jans,‡ Maarten Gees,‡ Monica Borgonovi,‡ Gert De Wilde,‡ and Katja Conrath‡

†Galapagos SASU, 102 Avenue Gaston Roussel, 93230 Romainville, France

‡Galapagos NV, Generaal De Wittelaan L11, A3, 2800 Mechelen, Belgium

§AbbVie, Inc., 60064-1802, 1 North Waukegan Road, North Chicago, Illinois, USA

***Corresponding author:** Romain Gosmini
Novalix, 102 Avenue Gaston Roussel, 93230 Romainville, France
Tel: 33617169449
Email: rgosmini@novalix.com

Table of contents

I. Supplemental PK tables

a. Table S1. Rat and dog PK data for 49 and 50 (S3)

b. Table S2. ADME and PK data of 51 (S3)

c. Table S3. ADME and PK data of 52 or GLPG2737 (S4-S5)

II. General Methods for Compound Synthesis/Analysis

a. General (S6)

b. General Methods for synthesis of intermediates (S7-S15)

c. Experimental procedure for the synthesis of intermediates and compounds 8-52 (S16-S62)

III. HPLC traces of compounds 8-52 (S63-S112)

IV. Formula strings (S113-S114)

I. Supplemental PK tables

Table S1. Rat and dog PK data for 49 and 50

	Clp (L/h/kg)<br>rat / dog	Cl.u (L/h/kg)<br>rat / dog	$t_{1/2\ iv}$ (h)<br>rat / dog	Vss (L/kg)<br>rat / dog	PPB (%)<br>rat / dog
**49**	0.06 / 0.02	4.2 / 0.5	4.7 / 6.6	0.4 / 0.2	99 / 98.5
**50**	0.11 / -	12.3 / -	3.31 / -	0.47 / -	99.1 / -

Table S2. ADME and PK data of 51

	XlogP3	TPSA	PPB %<br>rat<br>dog<br>human	CLint,u Hep (L/h/kg)<br>rat<br>dog<br>human	CYP induction @10 µM (mRNA) % rifampicin	Clp (L/h/kg)<br>rat / dog	Cl.u (L/h/kg)<br>rat / dog	$t_{1/2\ iv}$ (h)<br>rat / dog	Vss (L/kg)<br>rat / dog
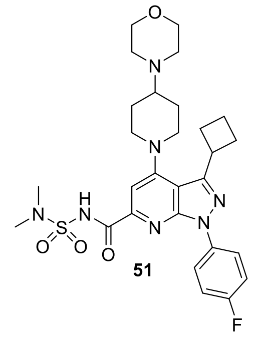<br>51	3.14	121	98.5<br>99<br>98.	2.94<br>1,9<br><0.66	**45%**[c]<br>24%[d]	0.19 /<br>0.16	13.2 /<br>12.1	3.6 /<br>4.9	0.81 /<br>0.9

[a]*Assay conditions are described in the experimental methods section using 5 (3 µM) as the positive control. All values are the geometric mean calculated from at least two runs, except when mentioned.* [b]*In contrast to the other TECC experiments, after the 24h incubation with a range of different concentrations, a fixed 1 µM C2 corrector concentration was added during the electrophysiological recording.* [c]*Data generated by AbbVie team.* [d]*Data generated by Galapagos*

Table S3. ADME and PK data of 52 or GLPG2**737**

**MDCK: PA2B (cm ×10^{-6} s^{-1}) / ER**	5.45 / 4.35 (n = 2)
**Caco-2: PA2B (cm ×10^{-6} s^{-1}) / ER**	24.5 / 1.27 (n = 1)
**Thermodynamic solubility**	
**FASSGF**	10.9 µg/mL
**pH 7.4**	9.9 µg/mL
**PPB (%)**	
**Rat/ dog/ human**	98.4 / 97.5 / 98
**Measured pKa: potentiometric method / UV method[a]**	
**Acylsulfonamyl pKa**	4.96 / 4.74
**Piperidine pKa**	8.42 / 8.51
**CYP inhibition in HLM**	IC_{50} (µM)
**CYPlA2 phenacetin**	>100
**CYP2C19 S-mephenytoin**	>33
**CYP2C9 diclofenac**	1.4
**CYP2D6 dextromethorphan**	>100
**CYP3A4 midazolam**	>100
**CYP3A4 testosterone**	>100
**Clintr,unbound (L/h/kg)**	

Rat/dog/ monkey/human	4.94/<1.92/20.8/4.00
**Hepatocyte stability (1mM)**<br>Mouse/ rat/dog/monkey/human<br>Predicted hepCl (L/h/kg)<br>(%LBF)	 <br>1.9/ 1.53/<0.35/1.73/<0.28<br>24/30/14/65/22
**Doses (mg/kg)**<br>Mouse/rat/dog (iv)<br>Mouse/rat/dog (po)	 1/ 1/ 0.5<br>5/ 30/ 5
**AUC $_{(0\text{-}24h)}$ (mg.h/mL)(1mg/kg, iv)**<br>Mouse/rat/dog	 3,061/ 2,395
**Clp (L/h/kg)**<br>Mouse/rat/dog	 0.28/ 0.32 / 0.21
**Cl.u (L/h/kg)**<br>Mouse/rat/dog	 66.7/ 20 / 10.7
**$t_{1/2\ iv}$ (h)**<br>Mouse/rat/dog	 3.22/ 3.45/ 5.4
**Vss (L/kg)**<br>Mouse/rat/dog	 1.27/1.38 / 1.37
**F (%)**<br>Mouse/rat/dog	 71.3/ 69.1/ 59.5

[a]*pKa were measured at Charles River.*

II. General Methods for Compound Synthesis/Analysis

a. General.

All reagents were of commercial grade and were used as received without further purification, unless otherwise stated. Commercially available anhydrous solvents were used for reactions conducted under inert atmosphere. Reagent grade solvents were used in all other cases, unless otherwise specified. Column chromatography was performed on silica gel 60 (35-70 µm). Thin layer chromatography was carried out using pre-coated silica gel F-254 plates (thickness 0.25 mm). Purification with preparatory HPLC was performed with Waters FractionLynx system coupled to 2996 PDA detector and a Waters Mass detector QDA. For basic method, column used: Waters XBridge prep (C18, 10 µm OBD, 19 x 100 mm), for acidic method, column used: Waters XSelect CSH (C18, 5 µm OBD, 19 x 100 mm). Flow rate: 20 mL/min. ^{1}H NMR spectra were recorded on a Bruker DPX 400 NMR spectrometer (400 MHz). Chemical shifts (δ) for ^{1}H NMR spectra are reported in parts per million (ppm) relative to tetramethyl silane (δ 0.00) or the appropriate residual solvent peak, i.e. $CHCl_3$ (δ 7.27), as internal reference. Multiplicities are given as singlet (s), doublet (d), triplet (t), quartet (q), quintuplet (quin), multiplet (m) and broad (br). Ultraviolet and electrospray MS spectra were obtained on a Waters platform LC/MS spectrometer or with Waters Acquity H-Class UPLC coupled to a Waters mass detector QDA. Purities were determined by LCMS analysis (UV traces determined with an Acquity PDA detector) using two methods. Method A: column used: Waters Acquity UPLC CSH C18 1.7µm, 2.1mm ID x 50mm L; MeCN/H_2O gradients (H_2O contains 0.1% formic acid); Method B: column used: Waters Acquity UPLC BEH C18 1.7µm, 2.1mm ID x 50 mmL; MeCN/H_2O gradients (H_2O contains 13.4 mM NH_3). All reported final compounds were analysed with one of these analytical methods and had purities ≥95% at the exception of 4 examples for which 1H NMR spectra are provided Microwave heating was performed with a Biotage Initiator.

b. General Methods for synthesis of intermediates (S7-S11)

General Method A1 for the synthesis of Arylidenepyruvic acids 59

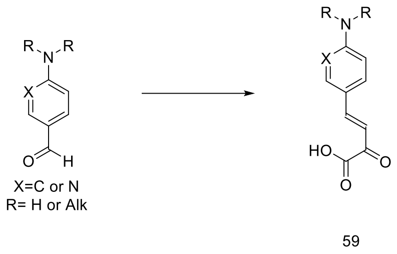

Scheme S1

A solution of potassium hydroxide (from1.5 to 2 eq) in water is added dropwise at 0 °C to a solution of aldehyde (1eq) and pyruvic acid (CAS 127-17-3, from1 eq to 1.5 eq) in methanol.

The reaction is warmed up to RT and then heated at 40 °C for 1 h to several days. Then the reaction mixture undergoes one of the following processes:

Either the formed precipitate is collected by filtration, suspended in an aqueous acidic solution, collected by filtration again, and dried in vacuo to give the titled compound.

Or alternatively, methanol is removed in vacuo, and the resulting suspension is filtered. The solid is taken up in water and either ethyl acetate or dichloromethane and acidified to pH = 3-5 with either acetic acid or an aqueous solution of 2 M HCl. The two phases are separated, and the aqueous phase is extracted with either ethyl acetate or dichloromethane. The combined organic phases are washed with water and brine, dried over $MgSO_4$, filtered and concentrated in vacuo to afford the titled compound which is used as such or further purified by precipitation.

General Method A2 for the synthesis of Arylidenepyruvate ethyl esters 60.

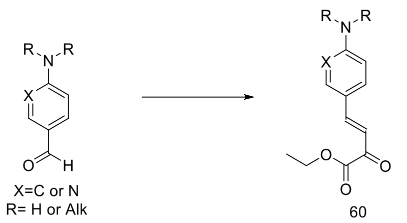

Scheme S2

Triflic acid (from 1.35 eq to 2.5 eq) is added dropwise to a solution of aldehyde (1 eq), triethyl orthoformate (from 1.1 eq to 1.3 eq) and ethyl pyruvate (from 1.5 to 3.5 eq) in chloroform. The solution is refluxed for 30 minutes to 24 h. The reaction mixture is cooled down to RT, diluted with dichloromethane, basified with a saturated aqueous solution of Na_2CO_3 or $NaHCO_3$. The two phases are separated, and the aqueous phase is extracted with dichloromethane. The combined organic phases are dried over sodium sulfate, filtered and concentrated under reduced pressure. This crude mixture is purified either by flash chromatography on silica gel or by precipitation to afford the titled compound.

General Method B1 for the synthesis of 5-amino-3-methyl-1-susbstituted pyrazoles 53.

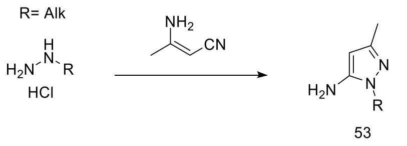

Scheme S3

3-Aminocrotononitrile (CAS: 1118-61-2, 1.1 eq), the hydrazine hydrochloride (1.0 eq) and few drops of 1 N HCl solution are heated in EtOH at reflux until the reaction is finished. The reaction mixture is cooled down to RT and then is diluted with a saturated solution of sodium hydrogencarbonate. The aqueous phase is extracted with dichloromethane. The combined organic phases are filtered through a phase separator and concentrated under vacuum to afford the aminopyrazole which is used as such.

General Procedure B2 for the synthesis of 5-amino-3-substituted-1-phenyl pyrazoles 53.

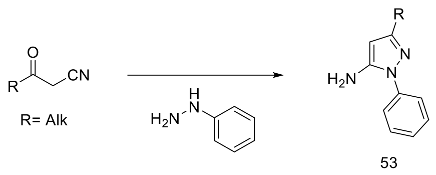

Scheme S4

To the hydrazine either as a free base or as a hydrochloride or a trifluoroacetic acid mono or di-salt (1 eq) and the cyanoketone (from 1.0 to 1.5 eq) in ethanol or toluene at RT. Then the reaction mixture is heated at reflux until the reaction is finished. The reaction mixture is concentrated in vacuo to afford a crude mixture which is used as such or purified by flash chromatography on silica gel to afford the aminopyrazole as its free base.

General Method C1 for the synthesis of Pyrazolo[3,4-b]pyridine-6-carboxylic acids PPCA through cyclocondensation of Arylidenepyruvic acids 59 with 5-amino-3,1-susbstituted pyrazoles 53.

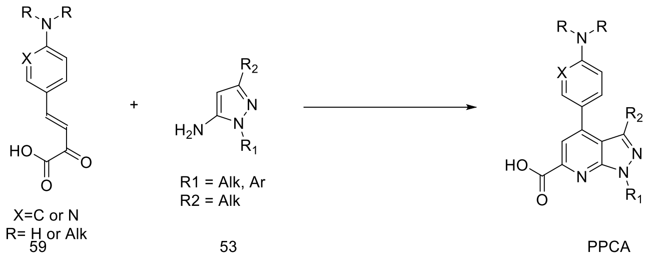

Scheme S5

The Arylidenepyruvic acid **59** (1 to 1.5 eq) and the 5-aminopyrazole **53** (1 to 1.5 eq) in acetic acid are stirred under air at temperatures ranging from RT to reflux for 1 h to several days. Alternatively, the Arylidenepyruvic acid **59** (1 to 1.5 eq) and the 5-aminopyrazole **53** (1 to 1.5

eq) in DMF or acetic acid are heated under microwave irradiation at 150 °C for 20 minutes to 2 h followed by stirring under air in an opened flask at temperatures ranging from RT to 90 °C for 1 h to several days with or without dilution of the reaction mixture with a large amount of ethanol or methanol. Then the reaction mixture is filtered, and the solid is washed with solvents, and dried in vacuo to afford the titled compound which is used as such or purified by precipitation, by preparative HPLC or by flash chromatography on silica gel. Alternatively, the reaction mixture is concentrated in vacuo to afford a crude mixture which is used as such or further purified either by precipitation, by preparative HPLC or by flash chromatography on silica gel.

General Method C2 for the synthesis of Pyrazolo[3,4-b]pyridine-6-carboxylate ethyl esters 61 through cyclocondensation of Arylidenepyruvate esters 60 with 5-amino-3,1-susbstituted pyrazoles 53.

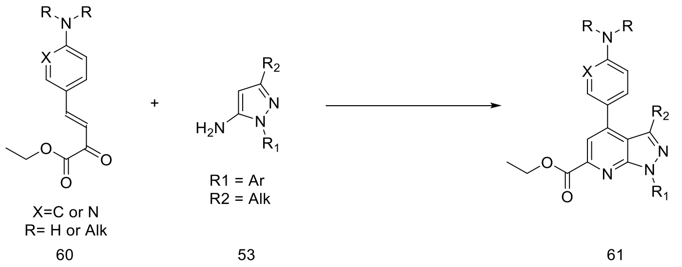

Scheme S6

The Arylidenepyruvate ethyl esters **60** (1 to 1.5 eq) and the aminopyrazole **53** (1 to 1.5 eq) in acetic acid are stirred under air at temperatures ranging from RT to reflux for 1 h to several days. Alternatively, the reaction mixture is heated under microwave irradiation at 150 °C for 20 minutes to 2 h followed either by stirring under air in an opened flask at temperatures ranging from RT to 90 °C for 1 h to several days or by removal of the solvent in vacuo, dilution of the residue in ethanol and stirring at reflux for 1 h to several days. Then volatiles are removed in vacuo to afford

the titled compound which is used as such or alternatively worked up by dilution with an organic solvent, washed successively with a basic aqueous solution and brine, dried over sodium sulfate, filtered and concentrated in vacuo and used as such or further purified either by precipitation, by preparative HPLC or by flash chromatography on silica gel.

General Method D for Buchwald-Hartwig cross coupling on 62.

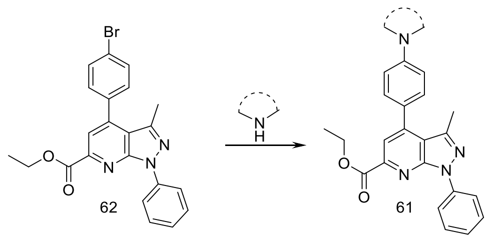

Scheme S7

To the amine (from 1.3 to 2 eq), **62** (1 eq) and sodium tert-butoxide (CAS: 865-48-5, from 1.3 to 3 eq) is added degassed anhydrous toluene. The reaction mixture is purged with argon, XPhos Pd G1 (CAS 1028206-56-5, 0.1 eq) is added, and the mixture is purged again with argon. The reaction mixture is stirred at a temperature ranging from 90 °C to 110 °C for 1 h to 24 h. The reaction mixture is cooled down and filtered on a pad of diatomaceous earth. Solids are washed with organic solvents, and the combined filtrates are concentrated in vacuo. The resulting residue is purified by flash chromatography on silica gel to afford the titled compound **61**.

General Method E for saponification of esters 61.

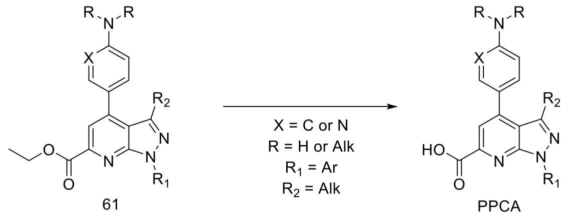

Scheme S8

To the intermediate ester (1 eq) in THF or methanol or in a mixture of THF/methanol or DCM/methanol at RT is added either 1 or 2 N aqueous sodium hydroxide (from 1 to 5 eq). The reaction mixture is stirred at a temperature ranging from RT to 70 °C for 1 hour to several days (up to 8 days). The volatiles are removed under reduced pressure, and the resulting mixture is acidified with either 1 or 2 N aqueous HCl or acetic acid. If a filterable suspension is obtained, the precipitate is collected by filtration, washed with water and dried in vacuo to afford the titled compound which is used as such or further purified either by preparative HPLC or flash chromatography on silica gel. In other cases, the mixture is partitioned between water and either dichloromethane or ethyl acetate. The two phases are separated, and the aqueous phase is extracted either with dichloromethane,methanol/dichloromethane or ethyl acetate. The combined organic phases are washed with brine, dried over $MgSO_4$, filtered and concentrated in vacuo to afford the titled compound which is used as such or further purified either by preparative HPLC or flash chromatography on silica gel.

General Method F for the synthesis of acylsulfonamides and acylsulfonylureas from Pyrazolo[3,4-b]pyridine-6-carboxylic acids PPCA.

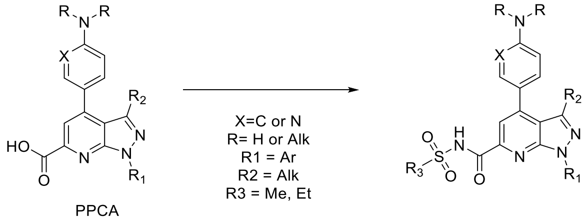

Scheme S9

EDC•HCl (1.0 to 4.0 eq) is added at RT to a stirring solution of carboxylic acid, sulfonamide or sulfamate (from 1.0 to 4.0 eq) and 4-(dimethylamino) pyridine (from 0.1 to 2.0 eq) in

dichloromethane or/and THF or/and acetone or/and acetonitrile. The reaction mixture is stirred at RT until full conversion is observed. The reaction mixture can be worked up or the solvent can be evaporated and the residue is purified by precipitation, by flash column chromatography or by preparative HPLC to yield the titled acylsulfonamide or acylsulfamide. To work up the reaction mixture, the solvent can be evaporated, and the residue is then partitioned between dichloromethane and water. The organic phase is successively washed with a saturated aqueous solution of NaHCO3, aqueous 0.5 N HCl and brine. The organic phase is separated, dried over sodium sulfate, filtered and concentrated in vacuo. Alternatively, the reaction mixture may be concentrated without an extractive workup. The residue is purified either by precipitation, by flash column chromatography or by preparative HPLC to yield the titled acylsulfonamide or acylsulfamide.

General Method G for the synthesis of aldehydes by SNAr.

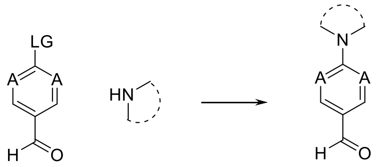

Scheme S10

A solution of the aldehyde (1 eq), the amine (1.3 to 2 eq) and DIPEA (2 eq) is prepared in acetonitrile. This mixture is heated under thermal conditions or under microwave irradiations at a temperature ranging from 85 °C to 150 °C. The reaction is worked up by diluting the reaction mixture with ethyl acetate or DCM and washing the organic phase with water and brine. In all cases, the organic phase is concentrated under reduced pressure, and the crude residue is used as such or purified either by flash column chromatography or precipitation to give the titled compound.

General Method H for the synthesis of ester 57 by SNAr.

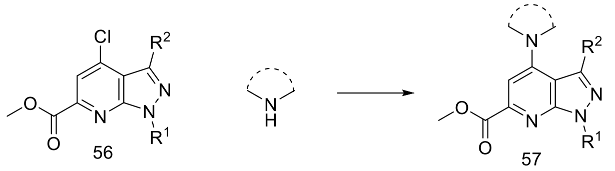

Scheme S11

A mixture of the chloride intermediate **56** (1.0 eq.), the amine as free base or hydrochloride salt (from 1 to 10 eq.) and DIPEA (from 1 to 15 eq.) in anhydrous acetonitrile and DMSO or *N*-methylpyrrolidinone in a sealed tube or a round bottom flask is heated at a temperature ranging from 50 to 130 °C for 1 h to several days (up to 8 days). The reaction mixture is cooled to RT, and volatiles are removed in vacuo. Alternatively, the reaction mixture can be worked up extractively. The resulting residue is either purified by precipitation or by flash chromatography on silica gel to afford the titled compound or alternatively partitioned between either dichloromethane or ethyl acetate and water. The two phases are then separated, and the aqueous phase is extracted with either ethyl acetate or dichloromethane. The combined organic phases are washed with brine, dried over $MgSO_4$, filtered, and concentrated in vacuo and the resulting crude mixture is either used as such or purified by flash chromatography on silica gel to afford the titled compound **57**.

General Method I for the synthesis of carboxylic acids 58 by saponification of ester 57.

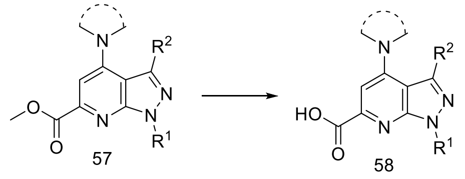

Scheme S12

To the ester intermediate **57** (1 eq.) in THF or a mixture of THF / methanol 1 / 1 at room temperature is added either aqueous 1 N or 2 N sodium hydroxide 1 (from 2 to 4 eq). The reaction mixture is stirred at room temperature until complete conversion is observed. Sodium hydroxide can be added to allow the full conversion of the starting ester. The volatiles are removed under reduced pressure, and the resulting mixture is acidified with either aqueous 1 N or 2 N HCl. If a filterable suspension is obtained, the precipitate is collected by filtration, washed with water, and

dried in vacuo to afford the titled compound **58** which is used as such or further purified either by preparative HPLC or flash chromatography on silica gel.

General Method J for the synthesis of acylsulfonamides and acylsulfonylureas from carboxylic acids 58.

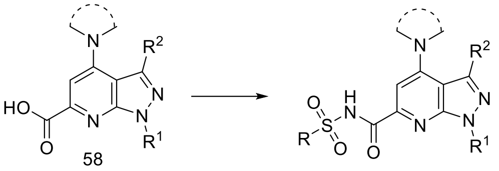

Scheme S13

EDC•HCl (CAS 25952-538, 1.2 to 1.4 eq.) is added at room temperature to a stirring solution of carboxylic acid **58** (1 eq.), sulfonamide or sulfamate (from 1.2 to 1.4 eq.) and 4-(dimethylamino) pyridine (CAS 1122-58-3, 0.2 eq.) in dichloromethane. The reaction mixture is stirred at room temperature until full conversion is observed. The reaction mixture can be worked up or the solvent can be evaporated, and the crude sample is purified by precipitation, by flash column chromatography or by preparative HPLC to yield the titled compound. To work up the reaction mixture, the solvent can be evaporated, and the crude sample is then partitioned between dichloromethane and water. The organic phase is successively washed with a saturated aqueous solution of $NaHCO_3$, aqueous 0.5 N HCl and brine. The organic phase is separated, dried over sodium sulfate, filtered and concentrated in vacuo. The residue is purified either by precipitation, by flash column chromatography or by preparative HPLC to yield the titled compound.

b. Experimental procedures for the synthesis of intermediates and compounds 8-52

6 was purchased from Enamine (CAS 941028-36-0) and **7** was purchased from Vitas-M Laboratory (CAS 926282-55-5). Other compounds were synthesized using the synthetic routes depicted below.

Synthesis of 1-substituted-3-methyl-4-(4-morpholinophenyl)-1H-pyrazolo[3,4-b]pyridine-6-carboxylic acids (**8,9,10,12)**

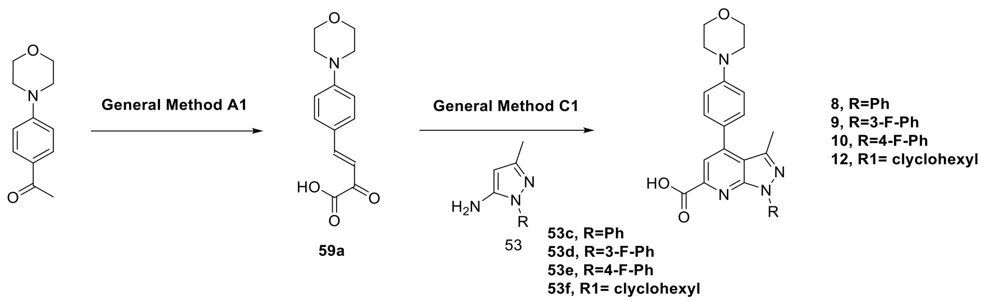

Scheme S14

(E)-4-(4-morpholinophenyl)-2-oxo-but-3-enoic acid (59a).

4-morpholinobenzaldehyde (40 g, 209 mmol, 1 eq) was treated according to general procedure A1 to afford the desired product **59a** (38 g, 70% yield). LCMS: m/z = 262.1 (M+H)+. 1H NMR (400 MHz, DMSO-d6) δ 13.96 (s, 1H), 7.74 – 7.59 (m, 3H), 7.05 (d, J = 16.1 Hz, 1H), 7.02 – 6.94 (m, 2H), 3.82 – 3.63 (m, 4H), 3.29 (t, J = 4.9 Hz, 4H).

3-methyl-4-[4-(morpholin-4-yl)phenyl]-1-phenyl-1H-pyrazolo[3,4-b]pyridine-6-carboxylic acid (8).

59a (20 g, 76.6 mmol, 1.05 eq) and 3-methyl-1-phenyl-1H-pyrazol-5-amine **53c** (12.6 g, 72.9 mmol, 1 eq) in acetic acid were treated according to general procedure C1 to afford the desired compound **8** (18 g, 60% yield). LCMS: m/z = 415.0 (M+H)+. 1H NMR (400 MHz, DMSO-d6) δ 8.37 – 8.30 (m, 2H), 7.77 (s, 1H), 7.63 – 7.50 (m, 4H), 7.40 – 7.31 (m, 1H), 7.17 – 7.09 (m, 2H), 3.78 (dd, J = 6.1, 3.6 Hz, 4H), 3.27 – 3.23 (m, 4H), 2.38 (s, 3H).

1-(3-fluorophenyl)-3-methyl-4-(4-morpholinophenyl)-1H-pyrazolo[3,4-b]pyridine-6-carboxylic acid (9).

59a (200 mg, 0.765 mmol, 1.0 eq) and 1-(3-fluorophenyl)-3-methyl-1H-pyrazol-5-amine **53d** (CAS: 105438-45-7; 146 mg, 0.765 mmol, 1.0 eq) in DMF were treated according to the general procedure C1 to afford the desired compound **9** LCMS: m/z = 433.0 (M+H)+. 1H NMR (400 MHz, DMSO-d6) δ 13.56 (s, 1H), 8.34 (dt, J = 11.2, 2.3 Hz, 1H), 8.27 (dd, J = 8.0, 2.0 Hz, 1H), 7.80 (s, 1H), 7.63 (td, J = 8.3, 6.7 Hz, 1H), 7.55 (d, J = 8.5 Hz, 2H), 7.23 – 7.10 (m, 3H), 3.78 (t, J = 4.8 Hz, 4H), 3.30 – 3.23 (m, 4H), 2.39 (s, 3H).

1-(4-fluorophenyl)-3-methyl-4-(4-morpholinophenyl)-1H-pyrazolo[3,4-b]pyridine-6-carboxylic acid (10).

59a (200 mg, 0.765 mmol, 1.0 eq) and 1-(4-fluorophenyl)-3-methyl-1H-pyrazol-5-amine **53e** (146 mg, 0.765 mmol, 1.0 eq) in DMF were treated according to the general procedure C1 to afford the desired compound **10**. LCMS: m/z = 433.0 $(M+H)^+$. ^{1}H NMR (400 MHz, DMSO-d_6) δ 13.56 (s, 1H), 8.39 – 8.31 (m, 2H), 7.78 (s, 1H), 7.59 – 7.52 (m, 2H), 7.51 – 7.40 (m, 2H), 7.14 (d, J = 8.7 Hz, 2H), 3.78 (dd, J = 6.0, 3.6 Hz, 4H), 3.26 (t, J = 4.9 Hz, 4H), 2.38 (s, 3H).

1-cyclohexyl-3-methyl-4-(4-morpholinophenyl)-1H-pyrazolo[3,4-b]pyridine-6-carboxylic acid (12).

59a (200 mg, 0.765 mmol, 1.0 eq) and 1-cyclohexyl-3-methyl-1H-pyrazol-5-amine **53f** (CAS : 56547-82-1, 137 mg, 0.765 mmol, 1.0 eq) in DMF were treated according to the general procedure C1 to afford the desired compound **12**. LCMS: m/z = 421.0 (M+H)+. 1H NMR (400 MHz, DMSO-d6) δ 13.32 (s, 1H), 7.64 (s, 1H), 7.53 – 7.45 (m, 2H), 7.15 – 7.06 (m, 2H), 4.90 (tt, J = 10.4, 5.2

Hz, 1H), 3.77 (t, J = 4.8 Hz, 4H), 3.24 (dd, J = 5.9, 3.8 Hz, 4H), 2.31 (s, 3H), 2.03 – 1.82 (m, 6H), 1.74 (d, J = 13.0 Hz, 1H), 1.60 – 1.44 (m, 2H), 1.29 (q, J = 13.0 Hz, 1H).

Synthesis of 1-substituted-3-methyl-4-(4-morpholinophenyl)-1H-pyrazolo[3,4-b]pyridine-6-carboxylic acids (**11,13,14**)

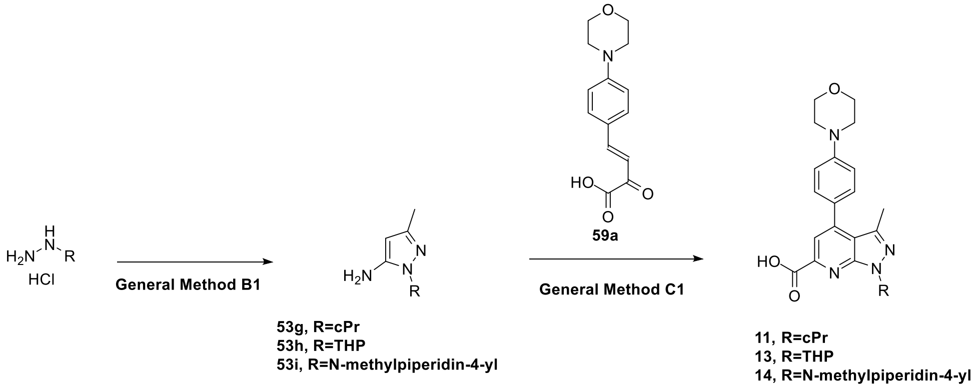

Scheme S15

1-cyclopropyl-3-methyl-1H-pyrazol-5-amine 53g.

Cyclopropylhydrazine hydrochloride (602 mg, 5.53 mmol, 1.0 eq) was treated according to general procedure B1 to afford the desired compound **53g**. LCMS: m/z = 138.1 (M+H)+.

1-cyclopropyl-3-methyl-4-(4-morpholinophenyl)-1H-pyrazolo[3,4-b]pyridine-6-carboxylic acid (11).

59a (200 mg, 0.765 mmol, 1.0 eq) and **53g** (105 mg, 0.765 mmol, 1.0 eq) in DMF were treated according to the general procedure C1 to afford the desired compound **11**. LCMS: m/z = 379.0 (M+H)+. 1H NMR (400 MHz, DMSO-d6) δ 7.65 (s, 1H), 7.48 (d, J = 8.4 Hz, 2H), 7.10 (d, J = 8.6 Hz, 2H), 4.01 (tt, J = 7.4, 3.9 Hz, 1H), 3.77 (t, J = 4.8 Hz, 4H), 3.24 (t, J = 4.9 Hz, 4H), 2.26 (s, 3H), 1.25 – 1.06 (m, 4H).

3-methyl-1-(tetrahydro-2H-pyran-4-yl)-1H-pyrazol-5-amine (53h).

(Tetrahydro-2H-pyran-4-yl)hydrazine dihydrochloride (1.04 g, 5.50 mmol, 1.0 eq) was treated according to general procedure B1 to afford the desired compound **53h**. LCMS: m/z = 182.2 (M+H)+.

3-methyl-4-(4-morpholinophenyl)-1-(tetrahydro-2H-pyran-4-yl)-1H-pyrazolo[3,4-b]pyridine-6-carboxylic acid (13).

59a (200 mg, 0.765 mmol, 1.0 eq) and **53h** (139 mg, 0.765 mmol, 1.0 eq) in DMF were treated according to the general procedure C1 to afford the desired compound **13**. LCMS: m/z = 423.0 (M+H)+. 1H NMR (400 MHz, DMSO-d6) δ 7.60 (s, 1H), 7.51 – 7.43 (m, 2H), 7.14 – 7.06 (m, 2H), 5.13 (tt, J = 11.6, 4.2 Hz, 1H), 4.02 (dd, J = 10.7, 4.2 Hz, 2H), 3.80 – 3.75 (m, 4H), 3.59 (td, J = 12.0, 1.9 Hz, 2H), 3.25 – 3.21 (m, 4H), 2.29 (s, 3H), 2.20 (qd, J = 12.4, 4.6 Hz, 2H), 1.91 – 1.83 (m, 2H).

3-methyl-1-(1-methylpiperidin-4-yl)-1H-pyrazol-5-amine (53i).

4-hydrazineyl-1-methylpiperidine hydrochloride (918 mg, 5.54 mmol, 1.0 eq) was treated according to general procedure B1 to afford the desired compound **53i**. LCMS: m/z = 195.2 (M+H)+.

3-methyl-1-(1-methylpiperidin-4-yl)-4-(4-morpholinophenyl)-1H-pyrazolo[3,4-b]pyridine-6-carboxylic acid (14).

59a (200 mg, 0.765 mmol, 1.0 eq) and **53i** (149 mg, 0.765 mmol, 1.0 eq) in DMF were treated according to the general procedure C1 to afford the desired compound **14**. LCMS: m/z = 436.0

(M+H)+. 1H NMR (400 MHz, DMSO-d6) δ 7.63 (s, 1H), 7.52 – 7.45 (m, 2H), 7.14 – 7.07 (m, 2H), 4.90 (s, 1H), 3.77 (dd, J = 5.9, 3.7 Hz, 4H), 3.24 (dd, J = 5.9, 3.8 Hz, 4H), 3.00 (d, J = 7.0 Hz, 2H), 2.35 – 2.16 (m, 10H), 1.96 – 1.84 (m, 2H).

Synthesis of 3-methyl-4-(6-morpholinopyridin-3-yl)-1-phenyl-1H-pyrazolo[3,4-b]pyridine-6-carboxylic acid **23**

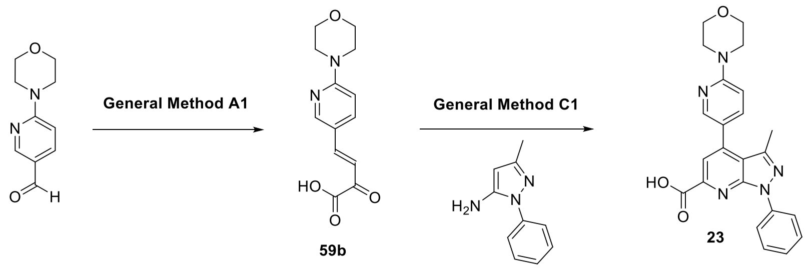

Scheme S16

(E)-4-(6-morpholino-3-pyridyl)-2-oxo-but-3-enoic acid (59b).

6-morpholinonicotinaldehyde (520 mg, 2.7 mmol, 1 eq) was treated according to general procedure A1 to afford the desired product **59b** (300 mg, 42% yield). LCMS: m/z = 263.1 (M+H)+.

3-methyl-4-(6-morpholinopyridin-3-yl)-1-phenyl-1H-pyrazolo[3,4-b]pyridine-6-carboxylic acid (23).

59b (131 mg, 0.5 mmol, 1.0 eq) and 3-methyl-1-phenyl-1H-pyrazol-5-amine **53c** (87 mg, 0.5 mmol, 1 eq) in acetic acid were treated according to general procedure C1 to afford the desired compound **23**. LCMS: m/z = 416.0 (M+H)+. 1H NMR (400 MHz, DMSO-d6) δ 8.43 (d, J = 2.5 Hz, 1H), 8.36 – 8.29 (m, 2H), 7.97 (dd, J = 8.9, 2.5 Hz, 1H), 7.82 (s, 1H), 7.64 – 7.56 (m, 2H), 7.41 – 7.32 (m, 1H), 7.09 (d, J = 9.0 Hz, 1H), 3.76 (dd, J = 5.8, 3.9 Hz, 4H), 3.64 – 3.60 (m, 4H), 2.42 (s, 3H).

Synthesis of **24, 25, 26, 28**

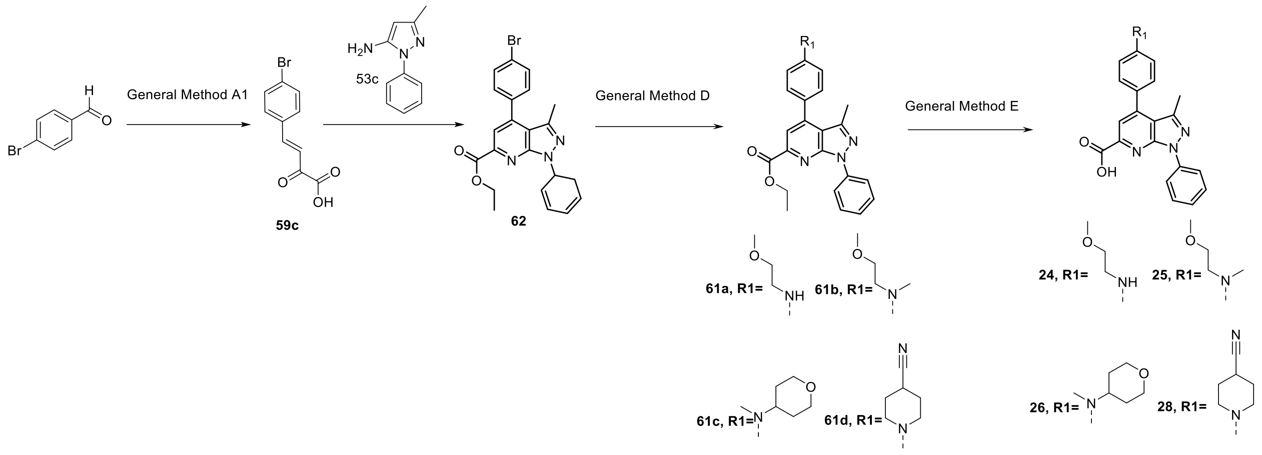

Scheme S17

(E)-4-(4-bromo-phenyl)-2-oxo-but-3-enoic acid (59c).

4-bromobenzaldehyde (15 g, 81.1 mmol, 1 eq) was treated according to general procedure A to afford the desired product **59c** (16.6 g, 80% yield). LCMS: m/z = 255.1(^{79}Br) / 257.1 (^{81}Br) (M+H)+. 1H NMR (400 MHz, DMSO-d6) δ 7.81 – 7.76 (m, 2H), 7.73 (d, J = 16.4 Hz, 1H), 7.69 – 7.63 (m, 2H), 7.33 (d, J = 16.4 Hz, 1H).

Ethyl 4-(4-bromophenyl)-3-methyl-1-phenyl-pyrazolo[3,4-b]pyridine-6-carboxylate (62).

59c (5 g, 19.60 mmol, 1 eq) and 5-amino-3-methyl-1-phenylpyrazole **53c** (3.39 g, 19.60 mmol, 1 eq) in acetic acid (17.8 ml) are stirred under air at reflux for 30 min. The reaction mixture was cooled down to RT and filtered. The yellow solid was washed with acetic acid and dried in vacuo. Then to the resulting solid in suspension in Ethanol (104 ml) at RT was added concentrated sulfuric acid (0.374 ml, 9% W/V). The reaction mixture was refluxed for 80h then left under stirring at RT under air for 3 days. The resulting suspension was filtered. The solid was washed with ethanol and then dried in vacuo. The crude material was purified by flash chromatography on silica gel (eluent

system: heptane/DCM) to give the titled compound **62** (2.25 g, 51% yield) LCMS: m/z = 436.3 (^{79}Br) / 438.3 (^{81}Br) $(M+H)^+$. ^{1}H NMR (300 MHz, Chloroform-d) δ 8.45 –8.33 (m, 2H), 7.91 (s, 1H), 7.77 – 7.69 (m, 2H), 7.64 – 7.53(m, 2H), 7.48 – 7.40 (m, 2H), 7.39 – 7.31 (m, 1H), 4.55 (q, J = 7.1 Hz, 2H), 2.39 (s, 3H), 1.50 (t, J = 7.1 Hz, 3H).

Ethyl 4-(4-((2-methoxyethyl)amino)phenyl)-3-methyl-1-phenyl-1H-pyrazolo[3,4-b]pyridine-6-carboxylate (61a).

61 (100 mg, 0.229 mmol, 1 eq) was treated with 2-methoxyethylamine according to general procedure D to afford the desired product **61a** (16 mg, 16% yield). LCMS: m/z = 431.5 (M+H)+.

4-(4-((2-methoxyethyl)amino)phenyl)-3-methyl-1-phenyl-1H-pyrazolo[3,4-b]pyridine-6-carboxylic acid (24).

61a (16 mg, 0.037 mmol, 1.0 eq) in DCM/Methanol was treated according to general procedure E to afford the desired compound **24** (12 mg, 80% yield). LCMS: m/z = 403.5 (M+H)+. 1H NMR (300 MHz, Chloroform-d) δ 8.09 – 7.98 (m, 3H), 7.57 (t, J = 7.9 Hz, 2H), 7.44 – 7.33 (m, 3H), 6.82 – 6.73 (m, 2H), 3.68 (dd, J = 5.7, 4.6 Hz, 2H), 3.44 (s, 3H), 3.39 (t, J = 5.2 Hz, 2H), 2.48 (s, 3H).

Ethyl 4-(4-((2-methoxyethyl)(methyl)amino)phenyl)-3-methyl-1-phenyl-1H-pyrazolo[3,4-b]pyridine-6-carboxylate (61b).

61 (300 mg, 0.688 mmol, 1 eq) was treated with 2-methoxy-N-methylethan-1-amine according to general procedure D to afford the desired product **61b** (41 mg, 13% yield). LCMS: m/z = 445.5 (M+H)+.

4-(4-((2-methoxyethyl)(methyl)amino)phenyl)-3-methyl-1-phenyl-1H-pyrazolo[3,4-b]pyridine-6-carboxylic acid (25).

61b (41 mg, 0.092 mmol, 1.0 eq) in DCM/Methanol was treated according to general procedure E to afford the desired compound **25** (22 mg, 59% yield). LCMS: m/z = 417.5 (M+H)+. 1H NMR (400 MHz, Chloroform-d) δ 8.09 – 8.00 (m, 3H), 7.61 – 7.52 (m, 2H), 7.48 – 7.41 (m, 2H), 7.38 (dd, J = 8.2, 6.7 Hz, 1H), 6.89 – 6.82 (m, 2H), 3.63 (s, 4H), 3.40 (s, 3H), 3.10 (s, 3H), 2.50 (s, 3H).

Ethyl 3-methyl-4-(4-(methyl(tetrahydro-2H-pyran-4-yl)amino)phenyl)-1-phenyl-1H-pyrazolo[3,4-b]pyridine-6-carboxylate (61c).

61 (100 mg, 0.229 mmol, 1 eq) was treated with N-methyltetrahydro-2H-pyran-4-amine according to general procedure D to afford the desired product **61c** (19 mg, 17% yield). LCMS: m/z = 471.5 (M+H)+.

3-methyl-4-(4-(methyl(tetrahydro-2H-pyran-4-yl)amino)phenyl)-1-phenyl-1H-pyrazolo[3,4-b]pyridine-6-carboxylic acid (26).

61c (19 mg, 0.040 mmol, 1.0 eq) in Methanol was treated according to general procedure E to afford the desired compound **26** (4 mg, 20% yield). LCMS: m/z = 443.2 (M+H)+. 1H NMR (400 MHz, Chloroform-d) δ 8.07 – 8.03 (m, 2H), 8.02 (s, 1H), 7.60 – 7.54 (m, 2H), 7.48 – 7.43 (m, 2H), 7.42 – 7.36 (m, 1H), 6.95 – 6.91 (m, 2H), 4.17 – 4.09 (m, 2H), 4.01 – 3.92 (m, 1H), 3.56 (td, J = 11.9, 2.0 Hz, 2H), 2.92 (s, 3H), 2.51 (s, 3H), 1.99 – 1.89 (m, 2H), 1.81 – 1.73 (m, 2H).

Ethyl 4-(4-(4-cyanopiperidin-1-yl)phenyl)-3-methyl-1-phenyl-1H-pyrazolo[3,4-b]pyridine-6-carboxylate (61d).

61 (100 mg, 0.229 mmol, 1 eq) was treated with piperidine-4-carbonitrile according to general procedure D to afford the desired product **61d** (55 mg, 51% yield). LCMS: m/z = 466.6 (M+H)+. 1H NMR (400 MHz, Chloroform-d) δ 8.45 – 8.35 (m, 2H), 7.92 (s, 1H), 7.59 – 7.53 (m, 2H), 7.51 – 7.46 (m, 2H), 7.36 – 7.30 (m, 1H), 7.13 – 7.06 (m, 2H), 4.53 (q, J = 7.1 Hz, 2H), 3.60 (ddd, J = 11.7, 7.2, 3.7 Hz, 2H), 3.30 (ddd, J = 12.4, 7.7, 3.5 Hz, 2H), 2.91 (tt, J = 7.9, 4.3 Hz, 1H), 2.45 (s, 3H), 2.21 – 2.01 (m, 4H), 1.50 (t, J = 7.1 Hz, 3H).

4-(4-(4-cyanopiperidin-1-yl)phenyl)-3-methyl-1-phenyl-1H-pyrazolo[3,4-b]pyridine-6-carboxylic acid (28).

61d (55 mg, 0.012 mmol, 1.0 eq) in DCM/Methanol was treated according to general procedure E to afford the desired compound **28** (31 mg, 61% yield). LCMS: m/z = 438.5 (M+H)+. 1H NMR (400 MHz, DMSO-d6) *δ* 8.38 – 8.31 (m, 2H), 7.78 (s, 1H), 7.64 – 7.56 (m, 2H), 7.56 – 7.50 (m, 2H), 7.41 – 7.32 (m, 1H), 7.15 (d, J = 8.7 Hz, 2H), 3.60 – 3.49 (m, 2H), 3.26 – 3.20 (m, 2H), 3.12 (tt, J = 8.4, 4.1 Hz, 1H), 2.39 (s, 3H), 2.06 – 2.00 (m, 2H), 1.94 – 1.79 (m, 2H).

Scheme S18. Synthesis of **27**

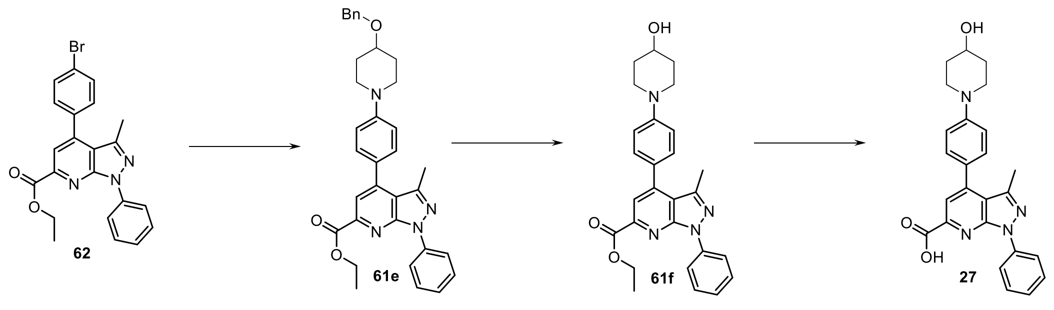

Ethyl 4-(4-(4-(benzyloxy)piperidin-1-yl)phenyl)-3-methyl-1-phenyl-1H-pyrazolo[3,4-

b]pyridine-6-carboxylate (61e)

61 (100 mg, 0.229 mmol, 1 eq) was treated with 4-(benzyloxy)piperidine according to general procedure D to afford the desired product **61e** (27 mg, 21% yield). LCMS: m/z = 547.6 (M+H)+. 1H NMR (400 MHz, Chloroform-d) δ 8.46 – 8.36 (m, 2H), 7.92 (s, 1H), 7.60 – 7.52 (m, 2H), 7.50 – 7.44 (m, 2H), 7.44 – 7.36 (m, 4H), 7.36 – 7.30 (m, 2H), 7.13 – 7.05 (m, 2H), 4.65 (s, 2H), 4.53 (q, J = 7.1 Hz, 2H), 3.70 (tq, J = 8.3, 4.2, 3.7 Hz, 3H), 3.15 (ddd, J = 12.5, 9.1, 3.3 Hz, 2H), 2.46 (s, 3H), 2.18 – 2.04 (m, 2H), 1.88 (dtd, J = 12.7, 8.7, 3.7 Hz, 2H), 1.50 (t, J = 7.1 Hz, 3H).

Ethyl 4-(4-(4-hydroxypiperidin-1-yl)phenyl)-3-methyl-1-phenyl-1H-pyrazolo[3,4-b]pyridine-6-carboxylate (61f)

To a solution of **61e** (26 mg, 0.047 mmol, 1 eq) in methanol (1 ml) under Argon atmosphere was added 10% palladium activated on carbon (8 mg, 30%w/w). The reaction mixture was purged with argon then with Hydrogen and kept under Hydrogen atmosphere. The reaction mixture was stirred at RT overnight. The reaction mixture was then purged with Argon and filtered on a pad of diatomaceous earth. Solids were washed with dichloromethane, and the filtrate was concentrated in vacuo to afford the titled compound **61f** (15 mg, 71% yield). LCMS: m/z = 457.6 (M+H)+.

4-(4-(4-hydroxypiperidin-1-yl)phenyl)-3-methyl-1-phenyl-1H-pyrazolo[3,4-b]pyridine-6-carboxylic acid (27).

61f (15 mg, 0.033 mmol, 1.0 eq) in DCM/Methanol was treated according to general procedure E to afford the desired compound **27** (14 mg, 100% yield). LCMS: m/z = 429.5 (M+H)+. 1H NMR (400 MHz, DMSO-d6) δ 8.38 – 8.31 (m, 2H), 7.78 (s, 1H), 7.60 (t, J = 8.0 Hz, 2H), 7.51 (d, J =

8.6 Hz, 2H), 7.36 (t, J = 7.4 Hz, 1H), 7.12 (d, J = 8.7 Hz, 2H), 4.73 (s, 1H), 3.75 –3.70 (m, 3H), 3.01 (ddd, J = 12.8, 10.0, 3.0 Hz, 2H), 2.40 (s, 3H), 1.88 – 1.83 (m, 2H), 1.56 – 1.43 (m, 2H).

Scheme S19. Synthesis of **15**

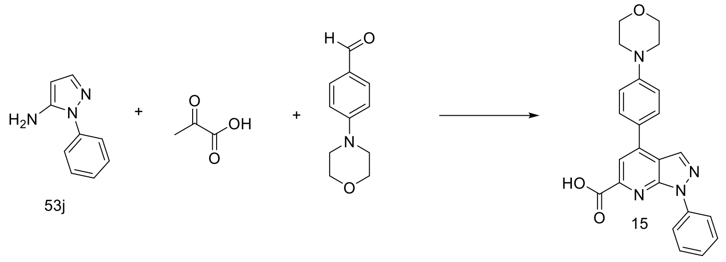

4-(4-morpholinophenyl)-1-phenyl-1H-pyrazolo[3,4-b]pyridine-6-carboxylic acid (15).

The 4-morpholinobenzaldehyde (241 mg, 1.26 mmol, 1 eq), pyruvic acid (111 mg, 1.26 mmol, 1 eq) and 1-phenyl-1H-pyrazol-5-amine **53j** (CAS:826-85-7200 mg, 1.26 mmol, 1 eq) in acetic acid (5 mL) were heated under microwave irradiation at 160°C for 20 minutes. The volatiles were removed in vacuo and the crude was purified by preparative HPLC to afford the desired compound **15** (269 mg, 53 % yield). LCMS: m/z = 401.0 (M+H)+. 1H NMR (400 MHz, DMSO-d6) δ 13.52 (s, 1H), 8.74 (s, 1H), 8.39 – 8.32 (m, 2H), 8.06 (s, 1H), 7.93 – 7.86 (m, 2H), 7.67 – 7.58 (m, 2H), 7.45 – 7.36 (m, 1H), 7.22 – 7.15 (m, 2H), 3.82 – 3.75 (m, 4H), 3.31 – 3.26 (m, 4H).

Scheme S17. Synthesis of 1-phenyl-3-substituted-4-(4-morpholinophenyl)-1H-pyrazolo[3,4-b]pyridine-6-carboxylic acids (**16,17,18)**

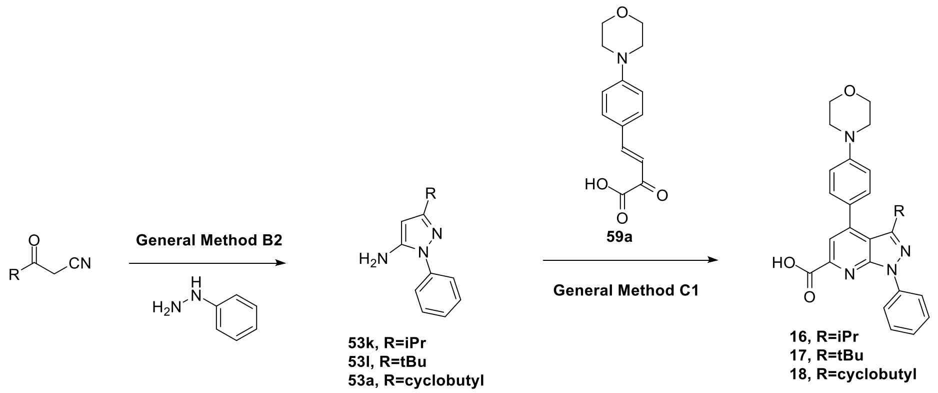

5-Isopropyl-2-phenyl-2H-pyrazol-3-ylamine (53k).

4-methyl-3-oxopentanenitrile (5.6 g, 51 mmol, 1.1 eq) and phenylhydrazine (5.0 g, 46 mmol, 1 eq) in EtOH was treated according to general procedure B2 to afford the desired compound **53k** (7g, 76% yield). LCMS: m/z = 202.1 (M+H)+. 1H NMR (400 MHz, Chloroform-d) δ 7.62 – 7.55 (m, 2H), 7.47 (dd, J = 8.6, 7.1 Hz, 2H), 7.37 – 7.30 (m, 1H), 5.51 (s, 1H), 3.79 (s, 2H), 2.96 (hept, J = 6.9 Hz, 1H), 1.30 (d, J = 6.9 Hz, 6H).

3-isopropyl-4-(4-morpholinophenyl)-1-phenyl-1H-pyrazolo[3,4-b]pyridine-6-carboxylic acid (16).

59a (260 mg, 0.99 mmol, 1.0 eq) and **53k** (200 mg, 0.99 mmol, 1.0 eq) in DMF were treated according to the general procedure C1 to afford the desired compound **16**. LCMS: m/z = 443.0 (M+H)+. 1H NMR (400 MHz, DMSO-d6) δ 8.35 (d, J = 8.1 Hz, 2H), 7.73 (d, J = 1.4 Hz, 1H), 7.60 (t, J = 7.9 Hz, 2H), 7.51 (d, J = 8.5 Hz, 2H), 7.36 (t, J = 7.4 Hz, 1H), 7.18 – 7.10 (m, 2H), 3.79 (dd, J = 6.0, 3.7 Hz, 4H), 3.27 – 3.24 (m, 4H), 2.55 (d, J = 2.0 Hz, 1H), 1.14 (d, J = 6.8 Hz, 6H).

5-tert-Butyl-2-phenyl-pyrazol-3-amine (53l).

4,4-dimethyl-3-oxopentanenitrile (500 mg, 4.0 mmol, 1.0 eq) and phenylhydrazine (433 mg, 432 mmol, 1 eq) in EtOH was treated according to general procedure B2 to afford the desired compound **53l**. LCMS: m/z = 216.1 (M+H)+.

3-(tert-butyl)-4-(4-morpholinophenyl)-1-phenyl-1H-pyrazolo[3,4-b]pyridine-6-carboxylic acid (17).

59a (243 mg, 0.99 mmol, 1.0 eq) and **53l** (200 mg, 0.93 mmol, 1.0 eq) in AcOH were treated according to the general procedure C1 to afford the desired compound **17**. LCMS: m/z = 457.1 (M+H)+. 1H NMR (400 MHz, DMSO-d6) δ 8.33 (d, J = 8.1 Hz, 2H), 7.65 – 7.56 (m, 3H), 7.36 (dd, J = 20.4, 7.8 Hz, 3H), 7.09 (d, J = 8.4 Hz, 2H), 3.79 (t, J = 4.8 Hz, 4H), 3.22 (t, J = 4.9 Hz, 4H), 1.20 (s, 9H).

5-Cyclobutyl-2-phenyl-2H-pyrazol-3-ylamine (53a).

3-cyclobutyl-3-oxopropanenitrile (320 mg, 2.6 mmol, 1.0 eq) and phenylhydrazine (280 mg, 2.6 mmol, 1 eq) in EtOH was treated according to general procedure B2 to afford the desired compound **53a** (500 mg, 90% yield). LCMS: m/z = 214.3 (M+H)+. 1H NMR (400 MHz, Chloroform-d) δ 7.62 – 7.56 (m, 2H), 7.53 – 7.43 (m, 2H), 7.37 – 7.31 (m, 1H), 5.60 (s, 1H), 3.79 (s, 2H), 3.60 – 3.46 (m, 1H), 2.41 – 2.31 (m, 2H), 2.30 – 2.18 (m, 2H), 2.09 – 1.85 (m, 2H).

3-cyclobutyl-4-(4-morpholinophenyl)-1-phenyl-1H-pyrazolo[3,4-b]pyridine-6-carboxylic acid (18).

59a (60 mg, 0.23 mmol, 1.0 eq) and **53a** (49 mg, 0.23 mmol, 1.0 eq) in AcOH were treated according to the general procedure C1 to afford the desired compound **18** (25mg, 24% yield). LCMS: m/z = 455.5 (M+H)+. 1H NMR (400 MHz, Chloroform-d) δ 8.14 – 8.08 (m, 2H), 7.99 (s, 1H), 7.62 – 7.54 (m, 2H), 7.46 – 7.41 (m, 2H), 7.41 – 7.36 (m, 1H), 7.09 – 7.01 (m, 2H), 3.97 – 3.89 (m, 4H), 3.68 (p, J = 8.4 Hz, 1H), 3.36 – 3.27 (m, 4H), 2.48 – 2.34 (m, 2H), 2.11 – 1.98 (m, 2H), 1.95 – 1.83 (m, 2H).

Scheme S20. Synthesis of **19**

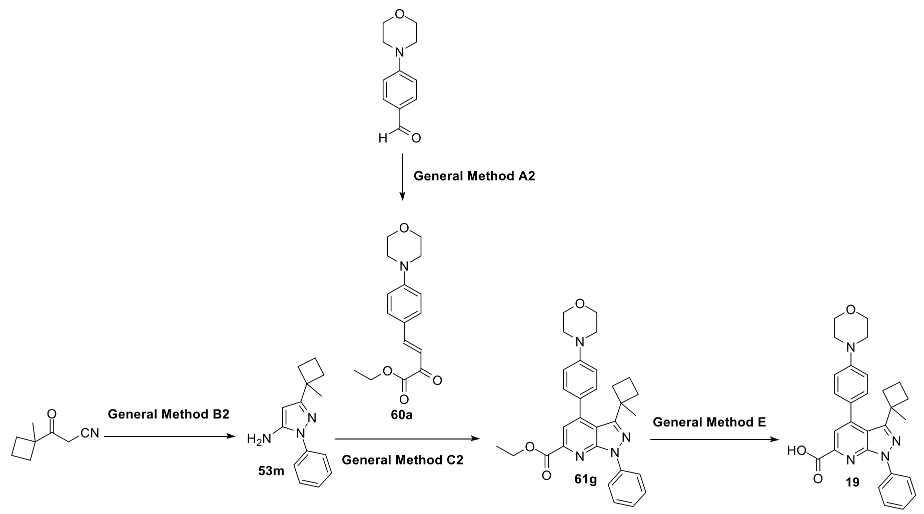

Ethyl (E)-4-(4-morpholinophenyl)-2-oxobut-3-enoate (60a).

4-morpholinobenzaldehyde (10 g, 52.3 mmol, 1.0 eq) was treated according to general procedure A2 to afford the desired product **60a**(11.6g, 76% yield). LCMS: m/z = 290.2 (M+H)+. 1H NMR (400 MHz, Chloroform-d) δ 7.82 (d, J = 15.9 Hz, 1H), 7.60 – 7.52 (m, 2H), 7.21 (d, J = 15.9 Hz, 1H), 6.92 – 6.84 (m, 2H), 4.38 (q, J = 7.1 Hz, 2H), 3.89 – 3.82 (m, 4H), 3.34 – 3.27 (m, 4H), 1.41 (t, J = 7.1 Hz, 3H).

5-(1-Methyl-cyclobutyl)-2-phenyl-2H-pyrazol-3-ylamine (53m).

3-(1-methylcyclobutyl)-3-oxopropanenitrile (CAS 2093977-91-2 230 mg, 1.68 mmol, 1.1 eq) and phenylhydrazine (165 mg, 1.53 mmol, 1.0 eq) in EtOH was treated according to general procedure B1 to afford the desired compound **53m** (173 mg, 78% yield). LCMS: m/z = 228.3 (M+H)+.1H NMR (400 MHz, Chloroform-d) δ 7.60 – 7.55 (m, 2H), 7.48 – 7.42 (m, 2H), 7.34 – 7.28 (m, 1H), 5.52 (s, 1H), 3.74 (s, 2H), 2.54 – 2.43 (m, 2H), 2.11 – 1.85 (m, 4H), 1.51 (s, 3H).

Ethyl 3-(1-methylcyclobutyl)-4-(4-morpholinophenyl)-1-phenyl-1H-pyrazolo[3,4-b]pyridine-6-carboxylate (61g).

60a (341 mg, 1.18 mmol, 1.0 eq) and **53m** (268 mg, 1.18 mmol, 1.0 eq) in AcOH were treated according to the general procedure C2 to afford the desired compound **61g** (100mg, 17% yield). LCMS: m/z = 497.5 (M+H)+. 1H NMR (400 MHz, Chloroform-d) δ 8.44 – 8.38 (m, 2H), 7.78 (s, 1H), 7.57 – 7.49 (m, 2H), 7.34 – 7.27 (m, 3H), 7.03 – 6.95 (m, 2H), 4.48 (q, J = 7.1 Hz, 2H), 3.96 – 3.89 (m, 4H), 3.30 – 3.22 (m, 4H), 2.66 – 2.55 (m, 2H), 2.02 – 1.89 (m, 1H), 1.78 – 1.69 (m, 1H), 1.69 – 1.60 (m, 2H), 1.45 (t, J = 7.1 Hz, 3H), 1.32 (s, 3H).

3-(1-methylcyclobutyl)-4-(4-morpholinophenyl)-1-phenyl-1H-pyrazolo[3,4-b]pyridine-6-carboxylic acid (19).

61g (90 mg, 0.18 mmol, 1.0 eq.) in THF/Methanol was treated according to general procedure E to afford the desired compound **19** (84 mg, 100% yield). LCMS: m/z = 469.5 (M+H)+. 1H NMR (300 MHz, Chloroform-d) δ 8.12 – 7.98 (m, 2H), 7.92 (s, 1H), 7.61 – 7.44 (m, 2H), 7.36 – 7.27 (m, 3H), 7.05 – 6.93 (m, 2H), 3.98 – 3.85 (m, 4H), 3.33 – 3.21 (m, 4H), 2.65 – 2.50 (m, 2H), 2.06 – 1.88 (m, 1H), 1.80 – 1.60 (m, 3H), 1.35 (s, 3H).

Scheme S21. Synthesis of 20

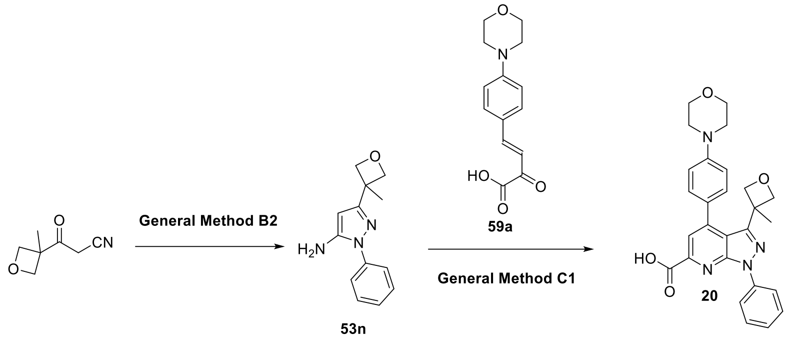

3-(3-methyloxetan-3-yl)-1-phenyl-1H-pyrazol-5-amine (53n).

3-(3-methyloxetan-3-yl)-3-oxopropanenitrile (CAS 1267855-75-3 76 mg, 0.55 mmol, 1.0 eq) and phenylhydrazine (59 mg, 0.55 mmol, 1.0 eq) in toluene was treated according to general procedure B1 to afford the desired compound **53n** (41 mg, 33% yield). LCMS: m/z = 230.2 (M+H)+.

1H NMR (400 MHz, Chloroform-d) δ 7.60 – 7.53 (m, 2H), 7.47 (dd, J = 8.6, 7.1 Hz, 2H), 7.38 – 7.30 (m, 1H), 5.64 (s, 1H), 5.00 (d, J = 5.4 Hz, 2H), 4.57 (d, J = 5.5 Hz, 2H), 3.81 (s, 2H), 1.74 (s, 3H).

3-(3-methyloxetan-3-yl)-4-(4-morpholinophenyl)-1-phenyl-1H-pyrazolo[3,4-b]pyridine-6-carboxylic acid (20).

59a (43 mg, 0.163 mmol, 1.0 eq) and **53n** (38 mg, 0.163 mmol, 1.0 eq) in AcOH were treated according to the general procedure C to afford the desired compound **20** (9mg, 12% yield). LCMS: m/z = 471.3 (M+H)+. 1H NMR (400 MHz, DMSO-d6) δ 13.56 (s, 1H), 8.31 (d, J = 8.0 Hz, 2H), 7.72 (s, 1H), 7.61 (t, J = 7.8 Hz, 2H), 7.39 (t, J = 7.3 Hz, 1H), 7.34 (d, J = 8.2 Hz, 2H), 7.12 (d, J = 8.3 Hz, 2H), 5.03 (d, J = 5.8 Hz, 2H), 4.04 (d, J = 5.8 Hz, 2H), 3.79 (t, J = 4.8 Hz, 4H), 3.24 (t, J = 4.8 Hz, 4H), 1.44 (s, 3H).

Scheme S22. Synthesis of **21**

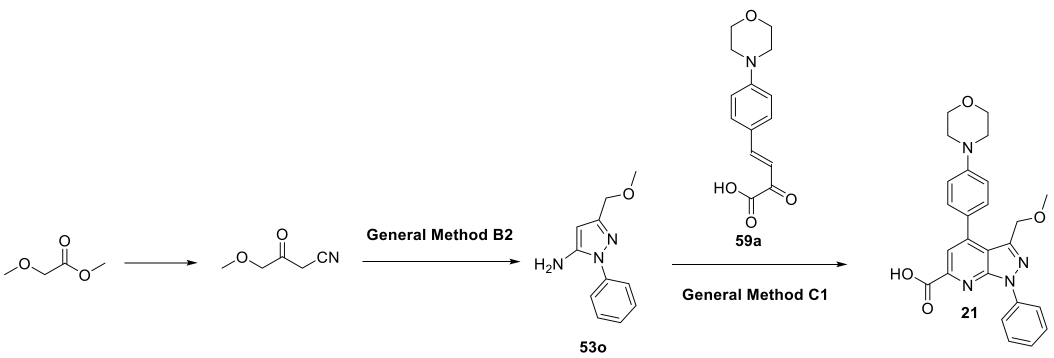

4-methoxy-3-oxobutanenitrile

Dry MeCN (1.1 mL, 21.1 mmol, 1.1 eq) was added to a solution of KOt-Bu (3.23 g, 28.8 mmol, 1.5 eq) in THF (20 mL) at 0°C. Then methyl 2-methoxyacetate (1.9 mL, 19.2 mmol, 1 eq) was added dropwise and the reaction mixture was stirred for 10 min at 0°C then for 18 h at RT. The reaction mixture was quenched by the addition of 2N aqueous HCl and the mixture was extracted with dichloromethane. The combined organic layer was washed with water then brine, dried over MgSO4, filtered and concentrated in vacuo. Purification by flash chromatography on silica gel (elution heptane/EtOAc: 100/0 to 50/50) afforded the titled compound **4-methoxy-3-oxobutanenitrile** (650 mg, 19% yield) which was directly engaged in the next step.

3-(methoxymethyl)-1-phenyl-1H-pyrazol-5-amine (53o).

4-methoxy-3-oxobutanenitrile (650 mg, 5.75 mmol, 1.0 eq) and phenylhydrazine (621 mg, 5.75 mmol, 1.0 eq) in EtOH was treated according to general procedure B2 to afford the desired compound **53o** (339 mg, 53% yield). LCMS: m/z = 204.0 (M+H)+. 1H NMR (400 MHz, Chloroform-d) δ 7.56 (d, J = 7.9 Hz, 2H), 7.47 (t, J = 7.8 Hz, 2H), 7.35 (t, J = 7.4 Hz, 1H), 5.68 (s, 1H), 4.42 (s, 2H), 3.80 (s, 2H), 3.42 (s, 3H).

3-(methoxymethyl)-4-(4-morpholinophenyl)-1-phenyl-1H-pyrazolo[3,4-b]pyridine-6-carboxylic acid (21).

59a (64 mg, 0.246 mmol, 1.0 eq) and **53o** (50 mg, 0.246 mmol, 1.0 eq) in AcOH were treated according to the general procedure C1 to afford the desired compound **21** (8mg, 8% yield). LCMS: m/z = 445.5 (M+H)+. 1H NMR (400 MHz, DMSO-d6) δ 13.55 (s, 1H), 8.35 – 8.27 (m, 2H), 7.84 (s, 1H), 7.68 – 7.58 (m, 4H), 7.41 (tt, J = 7.2, 1.2 Hz, 1H), 7.18 – 7.10 (m, 2H), 4.51 (s, 2H), 3.78 (t, J = 4.8 Hz, 4H), 3.26 (t, J = 4.9 Hz, 4H), 3.15 (s, 3H).

Scheme S23. Synthesis of **22**

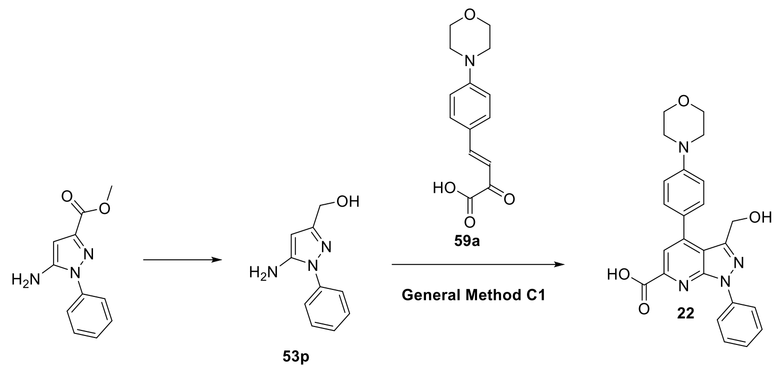

(5-amino-1-phenyl-1H-pyrazol-3-yl)methanol (53p).

A solution of ethyl 5-amino-1-phenyl-1H-pyrazole-3-carboxylate (300 mg, 1.3 mmol, 1.0 eq) in THF (6.5 mL), under nitrogen atmosphere was cooled down to -78°C. Diisobutylaluminum hydride (1 M in toluene, 2.9 mL, 2.9 mmol, 2.2 eq) was added dropwise to the solution, and the stirring at -78°C was continued for 1 hour. Methanol was added, and the reaction mixture was stirred for 30 minutes warming to rt. DCM was added, and the organic phase was washed with water. The organic phase was separated using a phase separator and concentrated under reduced pressure to afford the titled compound **53p** which was used as such without any further purification. LCMS: m/z = 190.1 (M+H)+.

3-(hydroxymethyl)-4-(4-morpholinophenyl)-1-phenyl-1H-pyrazolo[3,4-b]pyridine-6-carboxylic acid (22).

59a (200 mg, 0.765 mmol, 1.0 eq) and **53p** (145 mg, 0.765 mmol, 1.0 eq) in DMF were treated according to the general procedure C1 to afford the desired compound **22** . LCMS: m/z = 431.0 (M+H)+. 1H NMR (400 MHz, DMSO-d6) δ 13.51 (s, 1H), 8.38 – 8.30 (m, 2H), 7.82 (s, 1H), 7.71 – 7.65 (m, 2H), 7.65 – 7.58 (m, 2H), 7.39 (dd, J = 8.1, 6.7 Hz, 1H), 7.16 – 7.09 (m, 2H), 5.22 (t, J = 5.5 Hz, 1H), 4.62 (d, J = 5.3 Hz, 2H), 3.79 (t, J = 4.8 Hz, 4H), 3.30 – 3.24 (m, 3H).

Scheme S24. Synthesis of **33**

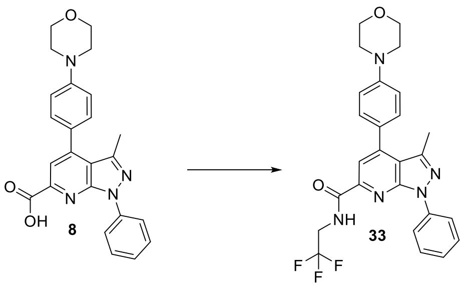

3-methyl-4-(4-morpholinophenyl)-1-phenyl-N-(2,2,2-trifluoroethyl)-1H-pyrazolo[3,4-b]pyridine-6-carboxamide (33).

To a solution of 3-methyl-4-(4-morpholinophenyl)-l-phenylpyrazolo[3,4-b]pyridine-6-carboxylic acid **8** (41 mg, 0.10 mmol, 1.0 eq.) in DCM (1.5 mL) with one drop of DMF was added oxalyl chloride (34 µL, 0.40 mmol, 4 eq.). The reaction mixture was stirred at RT for 30 min. Then a solution of 2,2,2-trifluoroethanamine (15 mg, 0.15 mmol, 1.5 eq) and triethylamine (140 µL, 1 mmol, 10 eq.) in DCM (3 mL) was added dropwise. The resulting solution was stirred overnight at RT. The reaction mixture was diluted with EtOAc with 10% iPrOH and washed with a saturated aqueous solution of NaHCO3. The organic phase was dried over sodium sulfate, filtered and concentrated under vacuo. The residue was recrystallized in a mixture DCM/ pentane/Diethyl

ether, filtered and dried in vacuo to afford the titled compound **33** (25 mg, 51 % yield). LCMS: m/z = 496.5 (M+H)+. 1H NMR (300 MHz, Chloroform-d) δ 8.27 (t, J = 6.7 Hz, 1H), 8.19 – 8.09 (m, 2H), 8.06 (s, 1H), 7.60 (t, J = 7.9 Hz, 2H), 7.55 – 7.45 (m, 2H), 7.45 – 7.34 (m, 1H), 7.12 – 7.03 (m, 2H), 4.21 (qd, J = 9.0, 6.7 Hz, 2H), 4.06 – 3.85 (m, 4H), 3.38 – 3.22 (m, 4H), 2.47 (s, 3H).

Scheme S25. Synthesis of **36**

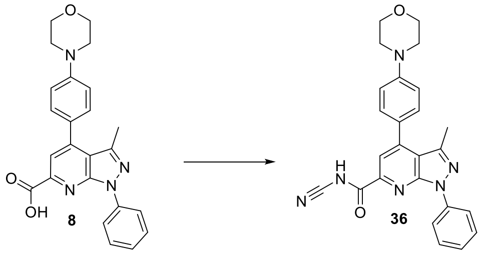

N-cyano-3-methyl-4-(4-morpholinophenyl)-1-phenyl-1H-pyrazolo[3,4-b]pyridine-6-carboxamide (36).

To a solution of 3-methyl-4-(4-morpholinophenyl)-l-phenylpyrazolo[3,4-b]pyridine-6-carboxylic acid **8** (82 mg, 0.20 mmol, 1.0 eq.) in DCM (5 mL) with one drop of DMF was added oxalyl chloride (70 µL, 0.82 mmol, 4.1 eq.). The reaction mixture was stirred at RT for 2h. Then a solution of cyanamide (16 mg, 0.38 mmol, 1.9 eq) and N,N-Diisopropylethylamine (280 µL, 1.6 mmol, 8 eq.) in DCM (3 mL) was added dropwise. The resulting solution was stirred at RT for 4h. The reaction mixture was diluted with DCM and washed with a saturated aqueous solution of NaHCO3. The organic phase was dried over sodium sulfate, filtered and concentrated under vacuo. The residue was purified by flash chromatography on silica gel (elution AcOEt:DCM/MeOH 90/10) to afford the titled compound **36** (35 mg, 40 % yield). LCMS: m/z = 439.2 (M+H)+. 1H NMR (400 MHz, Chloroform-d) δ 9.40 (s, 1H), 7.97 – 7.88 (m, 3H), 7.58 – 7.48 (m, 2H), 7.43 – 7.31 (m, 3H), 7.00 (d, J = 8.8 Hz, 2H), 3.90 – 3.81 (m, 4H), 3.29 – 3.21 (m, 4H), 2.39 (s, 3H).

Scheme S26. Synthesis of **37, 38**

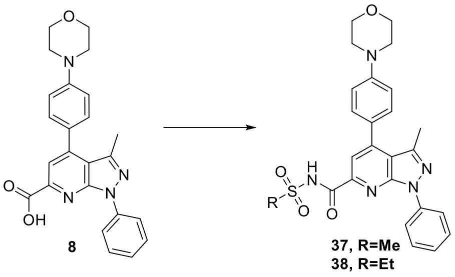

3-methyl-N-(methylsulfonyl)-4-(4-morpholinophenyl)-1-phenyl-1H-pyrazolo[3,4-b]pyridine-6-carboxamide (37).

8 (207 mg, 0.50 mmol, 1.0 eq.) was treated with methanesulfonamide in DCM according to general procedure F to afford the desired compound **37** (21 mg, 9% yield). LCMS: m/z = 492.2 (M+H)+. 1H NMR (400 MHz, DMSO-d6) δ 11.96 (s, 1H), 8.38 – 8.30 (m, 2H), 7.74 (s, 1H), 7.65 – 7.53 (m, 4H), 7.43 – 7.35 (m, 1H), 7.18 – 7.11 (m, 2H), 3.78 (dd, J = 6.2, 3.5 Hz, 4H), 3.42 (s, 3H), 3.27 (dd, J = 5.9, 3.9 Hz, 4H), 2.40 (s, 3H).

N-(ethylsulfonyl)-3-methyl-4-(4-morpholinophenyl)-1-phenyl-1H-pyrazolo[3,4-b]pyridine-6-carboxamide (38).

8 (207 mg, 0.50 mmol, 1.0 eq.) was treated with ethanesulfonamide in DCM according to general procedure F to afford the desired compound **38** (30 mg, 12% yield). LCMS: m/z = 506.3 (M+H)+. 1H NMR (400 MHz, chloroform-d6) δ 9.93 (s, 1H), 8.10 – 8.04 (m, 2H), 8.02 (s, 1H), 7.64 – 7.58 (m, 2H), 7.52 – 7.47 (m, 2H), 7.44 – 7.39 (m, 1H), 7.12 (d, J = 8.4 Hz, 2H), 4.00 – 3.93 (m, 4H), 3.64 (q, J = 7.4 Hz, 2H), 3.38 – 3.31 (m, 4H), 2.46 (s, 3H), 1.49 (t, J = 7.4 Hz, 3H).

Scheme S27. Synthesis of **30**

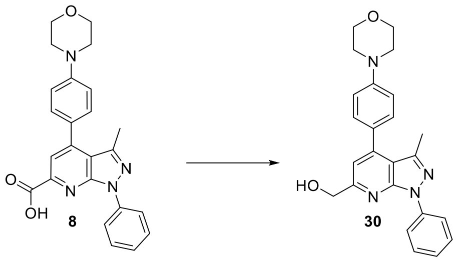

(3-methyl-4-(4-morpholinophenyl)-1-phenyl-1H-pyrazolo[3,4-b]pyridin-6-yl)methanol (30). BH3.SMe2 (112 µL, 1.5 mmol, 3 eq) was added at rt to a solution of 3-methyl-4-(4-morpholinophenyl)-1-phenyl-1H-pyrazolo[3,4-b]pyridine-6-carboxylic acid **8** (207 mg, 0.50 mmol, 1 eq) in THF (2 mL). The reaction mixture was stirred at rt for 16 hours. The volatiles were removed *in vacuo* and the residue was washed with acetonitrile and methanol to give the desired compound **30** (50 mg, 25% yield). LCMS: m/z = 401.0 (M+H)+. 1H NMR (400 MHz, DMSO-d6) δ 8.33 – 8.25 (m, 2H), 7.59 – 7.52 (m, 2H), 7.51 – 7.46 (m, 2H), 7.35 – 7.26 (m, 2H), 7.17 – 7.08 (m, 2H), 5.58 (s, 1H), 4.73 (s, 2H), 3.78 (t, J = 4.9 Hz, 4H), 3.27 – 3.22 (m, 4H), 2.33 (s, 3H).

Scheme S28. Synthesis of **35**

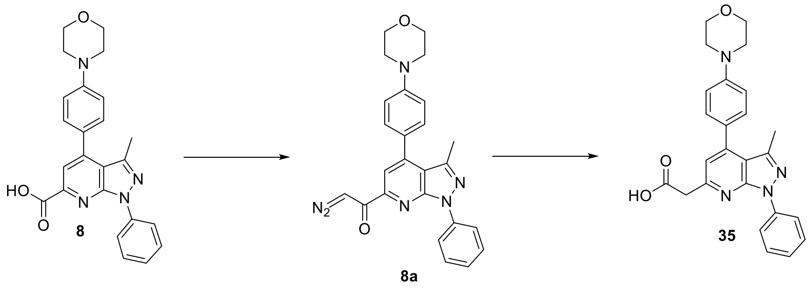

2-diazo-1-(3-methyl-4-(4-morpholinophenyl)-1-phenyl-1H-pyrazolo[3,4-b]pyridin-6-yl)ethan-1-one (8a).

At 0°C, oxalyl chloride (772 µL, 9 mmol, 1.8 eq) was added to a solution of 3-methyl-4-(4-morpholinophenyl)-1-phenyl-1H-pyrazolo[3,4-b]pyridine-6-carboxylic acid **8** (2.07 g, 5 mmol, 1 eq) in DCM (5 mL). One drop of DMF was added. The reaction mixture was stirred at 0°C for 1

hours. The volatiles were evaporated. Acetonitrile (5 mL), THF (20 mL), TMSCHN2 (5 mL, 10 mmol, 2 eq) and triethylamine (1.40 mL, 10 mmol, 2 eq) were added to the residue and the mixture was stirred for 24 hours at 4 °C. The solution was diluted with diethyl ether and water, the organic phase was successively washed with aqueous 1 N citric acid, saturated solution of sodium hydrogencarbonate and brine. The organic phase was dried over sodium sulfate, filtered and concentrated under reduced pressure to give the desired product **8a** which was used directly in the next step.

2-(3-methyl-4-(4-morpholinophenyl)-1-phenyl-1H-pyrazolo[3,4-b]pyridin-6-yl)acetic acid (35).

Silver acetate (322 mg, 2.1 mmol, 1 eq) was added to a solution of 2-diazo-1-(3-methyl-4-(4-morpholinophenyl)-1-phenyl-1H-pyrazolo[3,4-b]pyridin-6-yl)ethan-1-one **8a** (900 mg, 2.1 mmol, 1 eq) in THF (20 mL) and water (10 mL). The reaction mixture was stirred at rt for 16 hours. THF was evaporated. The residue was diluted with ethyl acetate and water, the organic phase was successively washed with aqueous 1 N citric acid, saturated solution of sodium hydrogencarbonate and brine. The organic phase was dried over sodium sulfate, filtered and concentrated under reduced pressure. The crude was purified by flash column chromatography eluting with DCM / MeOH + 0.2% acetic acid, followed by preparative HPLC to give the desired product **35** (2 mg, 0.2% yield). LCMS: m/z = 429.2 (M+H)+. 1H NMR (400 MHz, DMSO-d6) δ 8.33 – 8.28 (m, 2H), 7.61 – 7.50 (m, 2H), 7.50 – 7.44 (m, 2H), 7.35 – 7.26 (m, 1H), 7.17 – 7.07 (m, 3H), 3.85 (s, 2H), 3.81 – 3.76 (m, 4H), 3.26 – 3.23 (m, 4H), 2.33 (s, 3H).

Scheme S29. Synthesis of **29,31 and 32**

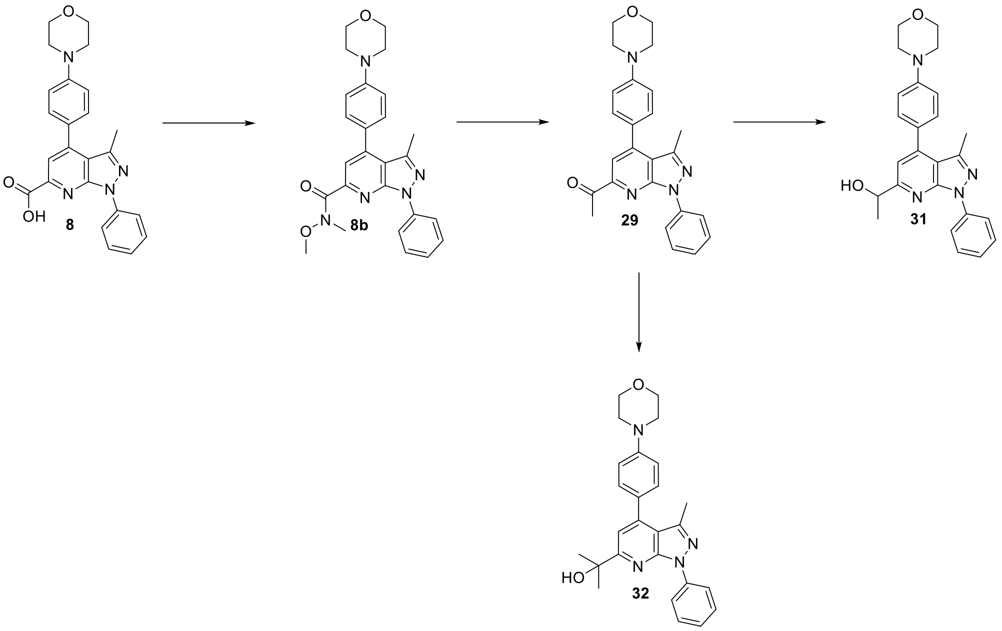

N-methoxy-N,3-dimethyl-4-(4-morpholinophenyl)-1-phenyl-1H-pyrazolo[3,4-b]pyridine-6-carboxamide (8b).

To a solution of 3-methyl-4-(4-morpholinophenyl)-l-phenylpyrazolo[3,4-b]pyridine-6-carboxylic acid **8** (207 mg, 0.5 mmol, 1.0 eq.) in DCM (30 mL) with one drop of DMF was added oxalyl chloride (170 µL, 2.0 mmol, 4.0 eq.). The reaction mixture was stirred at RT for 2h. Then a solution of N,O-dimethylhydroxylamine (122 mg, 2.0 mmol, 4.0 eq) and N,N-Diisopropylethylamine (700 µL, 4.0 mmol, 8.0 eq.) in DCM (5 mL) was added dropwise. The resulting solution was stirred at RT for 2h. The reaction mixture was diluted with AcOEt and water. The 2 phases were separated and the organic phase was washed with a saturated aqueous solution of NaHCO3, dried over sodium sulfate, filtered and concentrated under vacuo. The residue was purified by flash chromatography on silica gel (elution Heptane / AcOEt) to afford the titled compound **8b** (170mg, 74 % yield). LCMS: m/z = 458.5 (M+H)+.

1-(3-methyl-4-(4-morpholinophenyl)-1-phenyl-1H-pyrazolo[3,4-b]pyridin-6-yl)ethan-1-one (29).

To a solution of **8b** (69 mg, 0.15 mmol, 1.0 eq.) in THF (10 mL) at 0°C was added a 3M solution of Methylmagnesium bromide in Et2O (110 µL, 0.33 mmol, 2.2 eq.). The reaction mixture was warmed up to rt and stirred overnight. The reaction mixture was hydrolyzed with a saturated aqueous solution of NH4Cl and diluted with AcOEt. The 2 phases were separated and the aqueous phase was further extracted with AcOEt. The combined organic phases were washed with water, brine, dried over sodium sulfate, filtered and concentrated under vacuo. The residue was purified by flash chromatography on silica gel (elution Heptane/AcOEt) to give the desired product **29** (30 mg, 48% yield). LCMS: m/z = 413.4 (M+H)+. 1H NMR (400 MHz, Chloroform-d) δ 8.44 – 8.37 (m, 2H), 7.89 (s, 1H), 7.63 – 7.54 (m, 2H), 7.52 – 7.44 (m, 2H), 7.36 (tt, J = 7.3, 1.2 Hz, 1H), 7.11 – 7.03 (m, 2H), 3.98 – 3.91 (m, 4H), 3.36 – 3.29 (m, 4H), 2.89 (s, 3H), 2.46 (s, 3H).

1-(3-methyl-4-(4-morpholinophenyl)-1-phenyl-1H-pyrazolo[3,4-b]pyridin-6-yl)ethan-1-ol (31).

To a solution of **29** (22 mg, 0.054 mmol, 1.0 eq.) in MeOH (3 mL) at RT was added Sodium borohydride (15 mg, 0.40 mmol, 7.4 eq.). The reaction mixture was stirred RT for 2h. The reaction mixture was hydrolyzed with a saturated aqueous solution of NH4Cl and diluted with AcOEt. The 2 phases were separated and the aqueous phase was further extracted with AcOEt. The combined organic phases were washed with brine, dried over sodium sulfate, filtered and concentrated under vacuo. The residue was purified by flash chromatography on silica gel (elution Heptane /AcOEt) to give the desired product **31** (10 mg, 45% yield). LCMS: m/z = 415.4 (M+H)+. 1H NMR (400 MHz, Chloroform-d) δ 8.31 – 8.17 (m, 2H), 7.62 – 7.51 (m, 2H), 7.50 – 7.42 (m, 2H), 7.34 (tt, J =

7.3, 1.2 Hz, 1H), 7.10 – 7.05 (m, 2H), 7.04 (s, 1H), 5.08 (q, J = 6.6 Hz, 1H), 4.28 (s, 1H), 4.03 – 3.84 (m, 4H), 3.43 – 3.22 (m, 4H), 2.41 (s, 3H), 1.64 (d, J = 6.6 Hz, 3H).

2-(3-methyl-4-(4-morpholinophenyl)-1-phenyl-1H-pyrazolo[3,4-b]pyridin-6-yl)propan-2-ol (32).

To a solution of **29** (25 mg, 0.060 mmol, 1.0 eq.) in THF (3 mL) at 0°C was added a 3M solution of Methylmagnesium bromide in Et2O (44 µL, 0.12 mmol, 2.0 eq.). The reaction mixture was warmed up to rt and stirred overnight. The reaction mixture was hydrolyzed with a saturated aqueous solution of NH4Cl and diluted with AcOEt. The 2 phases were separated and the aqueous phase was further extracted with AcOEt. The combined organic phases were washed with brine, dried over sodium sulfate, filtered and concentrated under vacuo. The residue was purified by flash chromatography on silica gel (elution Heptane/AcOEt) to give the desired product **32** (7 mg, 30% yield). LCMS: m/z = 429.5 (M+H)+. 1H NMR (400 MHz, Chloroform-d) δ 8.26 – 8.17 (m, 2H), 7.60 – 7.52 (m, 2H), 7.51 – 7.45 (m, 2H), 7.38 – 7.31 (m, 1H), 7.15 (s, 1H), 7.11 – 7.05 (m, 2H), 4.96 (s, 1H), 4.01 – 3.89 (m, 4H), 3.39 – 3.23 (m, 4H), 2.42 (s, 3H), 1.67 (s, 6H).

Scheme S30. Synthesis of **34**

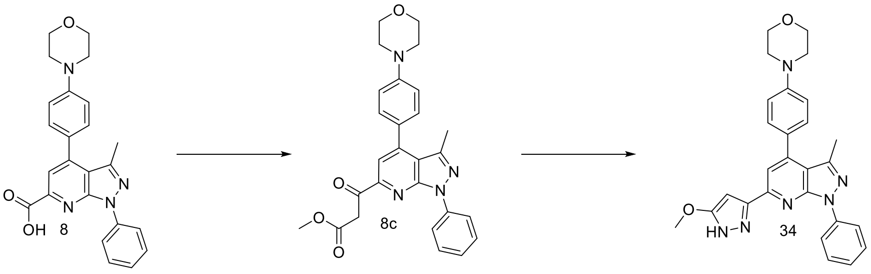

Methyl 3-(3-methyl-4-(4-morpholinophenyl)-1-phenyl-1H-pyrazolo[3,4-b]pyridin-6-yl)-3-oxopropanoate (8c).

To a solution of 3-methyl-4-(4-morpholinophenyl)-l-phenylpyrazolo[3,4-b]pyridine-6-carboxylic acid **8** (207 mg, 0.5 mmol, 1.0 eq.) in THF (20mL) and DCM (10 mL) at RT was added 1,1′-Carbonyldiimidazole (325 mg, 2.0 mmol, 4.0 eq.). The reaction mixture was stirred at RT for 2h. Then to the reaction mixture was added Potassium 3-methoxy-3-oxopropanoate (310 mg, 2.5 mmol, 5.0 eq) and Magnesium chloride (200 mg, 2.0 mmol, 4 eq.). The resulting mixture was stirred at RT overnight. The reaction mixture was diluted with AcOEt and water. The 2 phases were separated and the organic phase was dried over sodium sulfate, filtered and concentrated under vacuo. The residue was purified by flash chromatography on silica gel (elution Heptane / AcOEt) to afford the titled compound **8c** (90mg, 38 % yield). LCMS: m/z = 471.4 (M+H)+.

4-(4-(6-(5-methoxy-1H-pyrazol-3-yl)-3-methyl-1-phenyl-1H-pyrazolo[3,4-b]pyridin-4-yl)phenyl)morpholine (34).

To a solution of **8c** (20 mg, 0.043 mmol, 1.0 eq.) in MeOH (5 mL) at RT was added Hydrazine monohydrate (40 µL, 8.0 mmol, 18.8 eq.) and 3 drops of concentrated HCl. The reaction mixture was heated at reflux for 6h. The reaction mixture was concentrated in vacuo. The residue was solubilized with CHCl3 and water and AcONa was added portionwise until pH=5-6. The 2 phases were separated and the aqueous phase was further extracted with CHCl3. The combined organic phases were washed with brine, dried over sodium sulfate, filtered and concentrated under vacuo. The crude material was purified by flash chromatography on silica gel (elution AcOEt: DCM/MeOH 90/10) to give the titled product **34** (2 mg, 10% yield). LCMS: m/z = 467.2 (M+H)+. 1H NMR (400 MHz, Chloroform-d) δ 10.17 (s, 1H), 8.31 – 8.21 (m, 2H), 7.62 – 7.52 (m, 2H), 7.53 – 7.45 (m, 2H), 7.41 – 7.35 (m, 1H), 7.34 (s, 1H), 7.16 – 7.03 (m, 2H), 6.25 (s, 1H), 4.01 (s, 3H), 3.98 – 3.93 (m, 4H), 3.38 – 3.24 (m, 4H), 2.42 (s, 3H).

Scheme S31. Synthesis of compound **39**

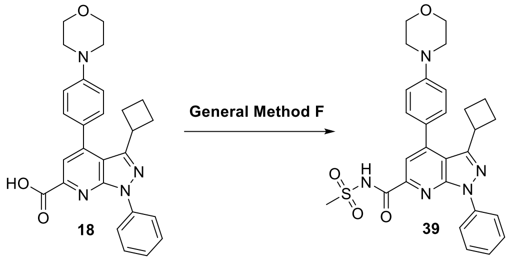

3-cyclobutyl-N-(methylsulfonyl)-4-(4-morpholinophenyl)-1-phenyl-1H-pyrazolo[3,4-b]pyridine-6-carboxamide (39).

18 (30 mg, 0.066 mmol, 1.0 eq) was treated with methanesulfonamide in DCM according to general procedure F to afford the desired compound **39** (22 mg, 63% yield). LCMS: m/z = 532.6 (M+H)+. 1H NMR (400 MHz, Chloroform-d) δ 10.04 (bs, 1H), 8.12 – 8.06 (m, 2H), 7.97 (s, 1H), 7.65 – 7.55 (m, 2H), 7.45 – 7.36 (m, 3H), 7.08 – 7.02 (m, 2H), 3.99 – 3.89 (m, 4H), 3.66 (p, J = 8.3 Hz, 1H), 3.44 (s, 3H), 3.36 – 3.28 (m, 4H), 2.46 – 2.34 (m, 2H), 2.09 – 1.97 (m, 2H), 1.94 – 1.82 (m, 2H).

Scheme S32. Synthesis of **40 and 41**

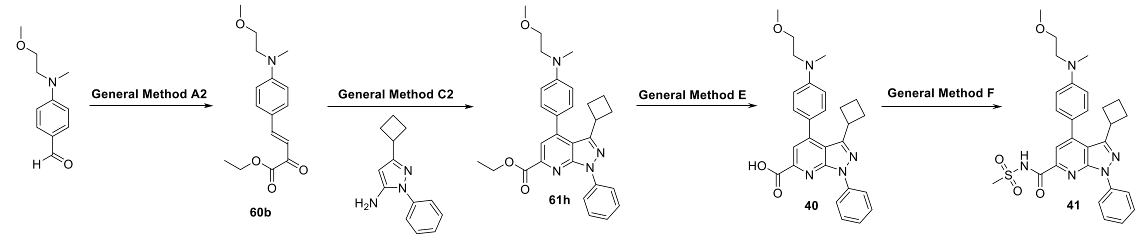

Ethyl (E)-4-(4-((2-methoxyethyl)(methyl)amino)phenyl)-2-oxobut-3-enoate (60b).

4-((2-methoxyethyl)(methyl)amino)benzaldehyde (CAS 27919-82-0; 504 mg, 2.61 mmol, 1.0 eq) was treated according to general procedure A2 to afford the desired product **60b** (501 mg, 66% yield). LCMS: m/z = 290.2 (M+H)+.

Ethyl 3-cyclobutyl-4-(4-((2-methoxyethyl)(methyl)amino)phenyl)-1-phenyl-1H-pyrazolo[3,4-b]pyridine-6-carboxylate (61h).

60b (163 mg, 0.61 mmol, 1.0 eq) and
53a (120 mg, 0.56 mmol, 1.0 eq) in AcOH were treated according to the general procedure C2 to afford the desired compound **61h** (180 mg, 61% yield). LCMS: m/z = 485.7 (M+H)+. 1H NMR (400 MHz, Chloroform-d) δ 8.47 – 8.42 (m, 2H), 7.85 (s, 1H), 7.56 – 7.51 (m, 2H), 7.41 – 7.36 (m, 2H), 7.31 – 7.27 (m, 1H), 6.88 – 6.83 (m, 2H), 4.49 (q, J = 7.1 Hz, 2H), 3.70 (p, J = 8.3 Hz, 1H), 3.64 (s, 4H), 3.41 (s, 3H), 3.10 (s, 3H), 2.48 – 2.36 (m, 2H), 2.10 – 2.01 (m, 2H), 1.93 – 1.83 (m, 2H), 1.47 (t, J = 7.1 Hz, 3H).

3-cyclobutyl-4-(4-((2-methoxyethyl)(methyl)amino)phenyl)-1-phenyl-1H-pyrazolo[3,4-b]pyridine-6-carboxylic acid (40).

61h (180 mg, 0.37 mmol, 1.0 eq.) in THF/Methanol was treated according to general procedure E to afford the desired compound **40** (90 mg, 53% yield). LCMS: m/z = 457.5 (M+H)+. 1H NMR (400 MHz, Chloroform-d) δ 8.14 – 8.07 (m, 2H), 7.99 (s, 1H), 7.62 – 7.53 (m, 2H), 7.43 – 7.34 (m, 3H), 6.89 – 6.82 (m, 2H), 3.74 (p, J = 8.4 Hz, 1H), 3.65 (s, 4H), 3.41 (s, 3H), 3.11 (s, 3H), 2.49 – 2.35 (m, 2H), 2.14 – 2.05 (m, 2H), 1.94 – 1.83 (m, 2H).

3-cyclobutyl-4-(4-((2-methoxyethyl)(methyl)amino)phenyl)-N-(methylsulfonyl)-1-phenyl-1H-pyrazolo[3,4-b]pyridine-6-carboxamide (41).

40 (41 mg, 0.09 mmol, 1.0 eq) was treated with methanesulfonamide in DCM according to general procedure F to afford the desired compound **41** (38 mg, 79% yield). LCMS: m/z = 534.6 (M+H)+. 1H NMR (300 MHz, Chloroform-d) δ 10.06 (s, 1H), 8.13 – 8.07 (m, 2H), 7.97 (s, 1H), 7.62 – 7.55 (m, 2H), 7.41 – 7.34 (m, 3H), 6.89 – 6.82 (m, 2H), 3.73 (p, J = 8.3 Hz, 1H), 3.64 (s, 4H), 3.43 (s, 3H), 3.41 (s, 3H), 3.11 (s, 3H), 2.50 – 2.34 (m, 2H), 2.13 – 2.01 (m, 2H), 1.96 – 1.83 (m, 2H).

Scheme S33. Synthesis of **42** and **43**

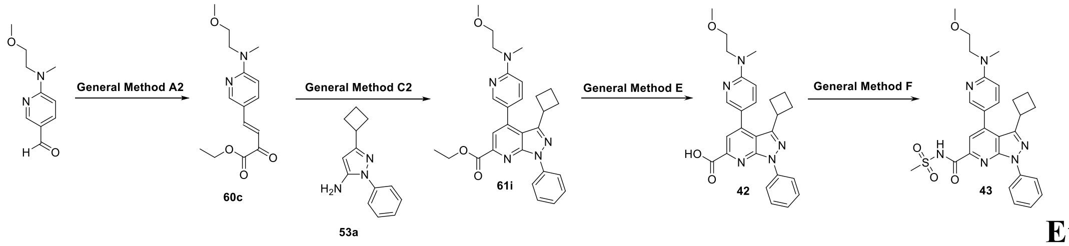

Ethyl (E)-4-(6-((2-methoxyethyl)(methyl)amino)pyridin-3-yl)-2-oxobut-3-enoate (60c).6-((2-methoxyethyl)(methyl)amino)nicotinaldehyde (CAS 1519963-64-4,1.33 g, 6.90 mmol, 1.0 eq) was treated according to general procedure A2 to afford the desired product 60c (1.7 g, 85% yield). LCMS: m/z = 293.3 (M+H)+.

Ethyl-3-cyclobutyl-4-(6-((2-methoxyethyl)(methyl)amino)pyridin-3-yl)-1-phenyl-1H-pyrazolo[3,4-b]pyridine-6-carboxylate (61i).

60c(137 mg, 0.47 mmol, 1.0 eq) and **53a**(100 mg, 0.47 mmol, 1.0 eq) in AcOH were treated according to the general procedure C2 to afford the desired compound **61i** (100 mg, 44% yield). LCMS: m/z = 486.6 (M+H)+. 1H NMR (400 MHz, Chloroform-d) δ 8.51 – 8.42 (m, 2H), 8.36 – 8.27 (m, 1H), 7.87 (s, 1H), 7.68 – 7.44 (m, 4H), 7.37 – 7.28 (m, 1H), 6.70 (dd, J = 8.9, 5.0 Hz,

1H), 4.55 (dq, J = 11.5, 7.2 Hz, 2H), 3.90 (dt, J = 11.1, 5.6 Hz, 2H), 3.73 (tp, J = 11.6, 5.7 Hz, 3H), 3.42 (d, J = 11.4 Hz, 3H), 3.24 (d, J = 6.1 Hz, 3H), 2.47 (dq, J = 11.5, 9.1 Hz, 2H), 2.27 – 2.03 (m, 2H), 2.06 – 1.69 (m, 2H), 1.52 (dt, J = 13.1, 7.1 Hz, 3H).

3-cyclobutyl-4-(6-((2-methoxyethyl)(methyl)amino)pyridin-3-yl)-1-phenyl-1H-pyrazolo[3,4-b]pyridine-6-carboxylic acid (42).

61i (100 mg, 0.20 mmol, 1.0 eq) in THF/Methanol was treated according to general procedure E to afford the desired compound **42** (33mg, 36% yield). LCMS: m/z = 458.6 (M+H)+. 1H NMR (400 MHz, Chloroform-d) δ 8.34 (dd, J = 2.6, 0.8 Hz, 1H), 8.14 (d, J = 8.0 Hz, 2H), 8.01 (s, 1H), 7.69 – 7.57 (m, 3H), 7.42 (t, J = 7.4 Hz, 1H), 6.75 – 6.68 (m, 1H), 3.92 (t, J = 5.6 Hz, 2H), 3.79 (q, J = 8.4 Hz, 1H), 3.71 (t, J = 5.6 Hz, 2H), 3.44 (s, 3H), 3.25 (s, 3H), 2.47 (pd, J = 9.0, 2.4 Hz, 2H), 2.23 – 2.06 (m, 2H), 2.06 – 1.87 (m, 2H).

3-cyclobutyl-4-(6-((2-methoxyethyl)(methyl)amino)pyridin-3-yl)-N-(methylsulfonyl)-1-phenyl-1H-pyrazolo[3,4-b]pyridine-6-carboxamide (43).

42 (20 mg, 0.043 mmol, 1.0 eq) was treated with methanesulfonamide in DCM according to general procedure F to afford the desired compound **43** (10 mg, 43% yield). LCMS: m/z = 535.5 (M+H)+. 1H NMR (400 MHz, Chloroform-d) δ 10.07 (s, 1H), 8.47 – 8.40 (m, 1H), 8.33 (dd, J = 2.6, 0.9 Hz, 1H), 8.13 (dd, J = 8.6, 1.3 Hz, 1H), 7.99 (s, 1H), 7.68 – 7.54 (m, 3H), 7.43 (t, J = 7.5 Hz, 1H), 6.71 (dd, J = 8.8, 4.8 Hz, 1H), 4.07 (s, 1H), 3.95 – 3.88 (m, 2H), 3.71 (t, J = 5.6 Hz, 2H), 3.44 (s, 3H), 3.25 (d, J = 2.4 Hz, 3H), 2.47 (dq, J = 11.2, 9.2 Hz, 2H), 2.16-2.11 (m, 2H), 2.04 – 1.83 (m, 2H), 1.59 (s, 3H).

Scheme S34. Synthesis of **44 and 45**

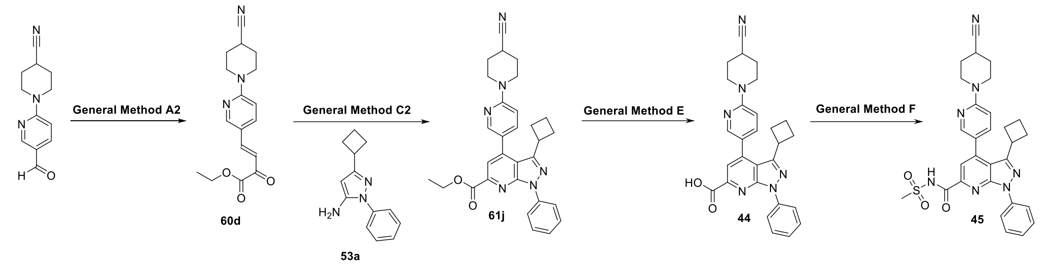

Ethyl (E)-4-(6-(4-cyanopiperidin-1-yl)pyridin-3-yl)-2-oxobut-3-enoate (60d).

1-(5-formylpyridin-2-yl)piperidine-4-carbonitrile (1.43 g, 6.90 mmol, 1.0 eq) was treated according to general procedure A2 to afford the desired product **60d** (1.63 g, 75% yield). LCMS: m/z = 314.3 (M+H)+.

ethyl 4-(6-(4-cyanopiperidin-1-yl)pyridin-3-yl)-3-cyclobutyl-1-phenyl-1H-pyrazolo[3,4-b]pyridine-6-carboxylate (61j).

60d (137 mg, 0.47 mmol, 1.0 eq) and **53a** (100 mg, 0.47 mmol, 1.0 eq) in AcOH were treated according to the general procedure C2 to afford the desired compound **61j** (118 mg, 49% yield). LCMS: m/z = 507.5 (M+H)+. 1H NMR (400 MHz, Chloroform-d) δ 8.50 – 8.41 (m, 3H), 8.39 – 8.34 (m, 1H), 7.87 (d, J = 0.8 Hz, 1H), 7.69 (dd, J = 8.8, 2.5 Hz, 1H), 7.62 – 7.54 (m, 3H), 7.34 (t, J = 7.4 Hz, 2H), 6.85 (d, J = 8.8 Hz, 1H), 4.60 – 4.49 (m, 3H), 4.16 – 3.89 (m, 3H), 3.85 – 3.49 (m, 4H), 3.00 (dt, J = 7.6, 3.5 Hz, 1H), 2.71 – 2.32 (m, 3H), 2.33 – 1.74 (m, 5H), 1.51 (td, J = 7.1, 0.8 Hz, 3H).

4-(6-(4-cyanopiperidin-1-yl)pyridin-3-yl)-3-cyclobutyl-1-phenyl-1H-pyrazolo[3,4-b]pyridine-6-carboxylic acid (44).

61j (118 mg, 0.23 mmol, 1.0 eq) in THF/Methanol was treated according to general procedure E to afford the desired compound **44** (100mg, 90% yield). LCMS: m/z = 479.5 (M+H)+. 1H NMR (400 MHz, Chloroform-d) δ 8.37 (dd, J = 2.5, 0.7 Hz, 1H), 8.17 – 8.11 (m, 1H), 8.15 – 8.06 (m, 1H), 8.00 (s, 1H), 7.70 (dd, J = 8.8, 2.5 Hz, 1H), 7.67 – 7.55 (m, 2H), 7.48 – 7.39 (m, 1H), 6.89 – 6.82 (m, 1H), 4.01 (ddd, J = 13.6, 7.3, 3.7 Hz, 2H), 3.88 – 3.59 (m, 3H), 3.01 (tt, J = 7.9, 4.3 Hz, 1H), 2.47 (dq, J = 11.4, 9.0 Hz, 2H), 2.27 – 1.75 (m, 8H).

4-(6-(4-cyanopiperidin-1-yl)pyridin-3-yl)-3-cyclobutyl-N-(methylsulfonyl)-1-phenyl-1H-pyrazolo[3,4-b]pyridine-6-carboxamide (45).

44 (85 mg, 0.177 mmol, 1.0 eq) was treated with methanesulfonamide in DCM according to general procedure F to afford the desired compound **45** (41 mg, 41% yield). LCMS: m/z = 556.6 (M+H)+. 1H NMR (300 MHz, Chloroform-d) δ 9.91 (s, 1H), 8.22 (dd, J = 2.6, 0.7 Hz, 1H), 8.03 – 7.93 (m, 2H), 7.84 (s, 1H), 7.59 – 7.44 (m, 3H), 7.36 – 7.24 (m, 1H), 6.72 (d, J = 8.8 Hz, 1H), 3.97 – 3.80 (m, 3H), 3.67 – 3.50 (m, 4H), 3.34 (s, 3H), 2.87 (tt, J = 7.9, 4.3 Hz, 1H), 2.42 – 2.23 (m, 2H), 2.06 – 1.72 (m, 6H).

Scheme S35. Synthesis of 56a: methyl 4-chloro-3-cyclobutyl-1-phenyl-pyrazolo[3,4-b]pyridine-6-carboxylate

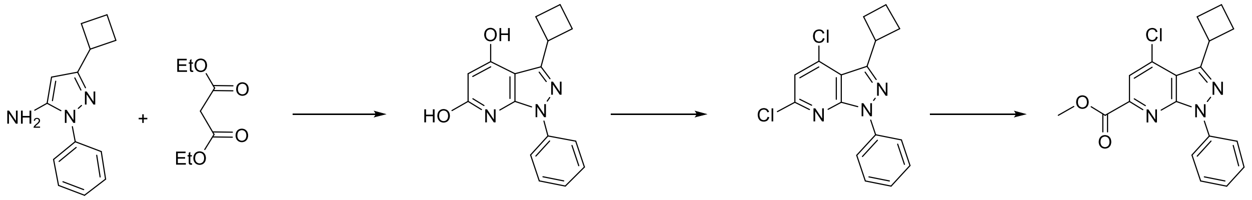

53a 54a 55a 56a

3-cyclobutyl-1-phenyl-pyrazolo[3,4-b]pyridine-4,6-diol 54a

To a mixture of 3-cyclobutyl-1-phenyl-1H-pyrazol-5-amine **53a** (74.0 g, 347 mmol) in oxydibenzene (450.0 g, 2.64 mol) was added diethyl malonate (CAS 105-53-3, 139.0 g, 867 mmol). The system was heated at 130-150 °C for 40 h. By this time solid had precipitated and heating was stopped. Two more reactions were set up as described above. All three reaction mixtures were combined. The combined mixture was cooled to below 40 °C and diluted with about 1.8 L of diethyl ether, and the resulting suspension was stirred for 2 h and then filtered. The collected solids were rinsed with diethyl ether (1 L). The solids were dried on the filter to give the titled compound **54a (65.4 g, 67%)**. LCMS: m/z = 282.4 (M+H)+. 1H NMR (400 MHz, DMSO-d6) δ ppm 11.28 (s, 1H), 8.18 (d, J=7.9 Hz, 2H), 7.58 - 7.39 (m, 2H), 7.21 (t, J=7.1 Hz, 1H), 5.85 (s, 1H), 3.88 (quin, J=8.4 Hz, 1H), 2.44 - 2.22 (m, 4H), 2.07 - 1.78 (m, 2H).

4,6-dichloro-3-cyclobutyl-1-phenyl-pyrazolo[3,4-b]pyridine 55a

A mixture of 3-cyclobutyl-1-phenyl-pyrazolo[3,4-*b*]pyridine-4,6-diol **54a** (60.0 g, 213 mmol) in phenyl dichlorophosphate (CAS 770-12-7,135 g, 640 mmol) was stirred at 170 °C for 15 h. Two more reactions were set up as described above. All three reaction mixtures were combined and poured into ice water (5 L) keeping the internal temperature <10 °C. The mixture was neutralized with concentrated NH_4OH (500 mL) to pH 6~7, then the suspension was stirred for 2 h. As the pH increased and with continued stirring, the semi-solid suspension becomes a flowing solid. The solid was collected by filtration. The wet solid was dissolved in dichloromethane (3 L) and filtered through a short path of silica gel (2 kg), eluting with dichloromethane (15 L). The filtrate was concentrated to a solid which was triturated with acetonitrile (1.5 L) and and collected by filtration to give the titled compound **55a (50.9 g, 75%)**. LCMS: m/z = 319.8 (M+H)+. ^{1}H NMR (400 MHz,DMSO-d_6) δ ppm 8.20 (d, J=7.9 Hz, 2H), 7.51 (t, J=7.5 Hz, 2H), 7.35 - 7.27 (m, 1H), 7.16 (s, 1H), 4.15 (quin, J=8.4 Hz, 1H), 2.63 - 2.41 (m, 4H), 2.20 - 2.07 (m, 1H), 2.00 (s, 1H).

Methyl 4-chloro-3-cyclobutyl-1-phenyl-pyrazolo[3,4-b]pyridine-6-carboxylate 56a

To a mixture of 4,6-dichloro-3-cyclobutyl-1-phenyl-1*H*-pyrazolo[3,4-*b*]pyridine **55a** (50.0 g, 157 mmol) in methanol (700 mL) was added triethylamine (31.8 g, 314 mmol) and Pd(dppf)Cl_2•DCM (CAS 95464-05-4, 6.4 g, 7.86 mmol). The system was heated at 60 °C under CO (30 psi) for 40 h. Two more reactions were set up as described above. All three reaction mixtures were combined and concentrated to give a semisolid which was dissolved in dichloromethane (3 L) and filtered through a 2 kg plug of silica gel. After concentration, about 130 g of solid was obtained. This solid was taken up in 1.3 L of ethyl acetate with heating. This solution was stirred at room temperature. Solids came out over a couple of minutes, and then 1.3 L of hexane was added in a thin stream via addition funnel with stirring for 2 hours. The solids were collected by filtration to give the titled compound **56a** (31.14 g, 58%) LCMS: m/z = 342.0 (M+H)+. ^{1}H NMR (400 MHz, $CDCl_3$) δ ppm 8.38 - 8.27 (m, 2H), 7.95 (s, 1H), 7.53 (t, J=7.9 Hz, 2H), 7.35 - 7.27 (m, 1H), 4.21 (q, J=8.6 Hz, 1H), 4.08 - 4.03 (m, 1H), 2.66 - 2.40 (m, 4H), 2.22 - 1.93 (m, 2H).

Scheme S36. Synthesis of 53b:

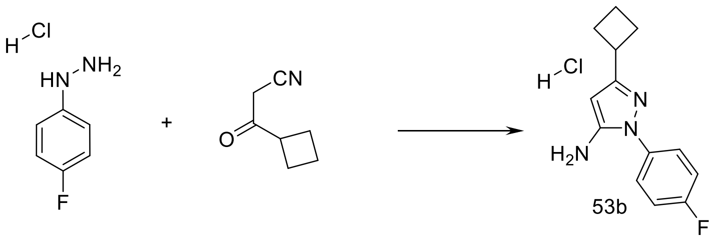

53b

A round bottom flask was charged with 3-cyclobutyl-3-oxo-propionitrile (CAS 118431-89-3, 10 g, 81.4 mmol), 4-fluorophenylhydrazine hydrochloride (CAS 823-85-8, 12 g, 74 mmol) and EtOH (35 mL). The reaction mixture was refluxed for 2 hours and cooled down to RT. Half of the solvent was concentrated *in vacuo*. The mixture was vigorously stirred and diisopropyl ether (350 mL) was added. The stirring was continued for 1 hour and the formed precipitate was filtered, washed with diisopropyl ether and dried at 40°C under reduced pressure to give the titled compound **53b** as HCl salt (19,8 g, 100 %). LCMS: m/z = 332.3 (M+H)+. 1H NMR (400 MHz, DMSO) δ 7.67 – 7.53 (m, 2H), 7.36 – 7.21 (m, 2H), 5.39 (s, 1H), 5.27 (s, 2H), 3.37 – 3.29 (m, 1H), 2.25 – 2.02 (m, 4H), 1.99 – 1.75 (m, 2H).

Scheme S37. Synthesis of 56b : Methyl 4-chloro-3-cyclobutyl-1-(4-fluorophenyl)-1H-pyrazolo[3,4-b]pyridine-6-carboxylate

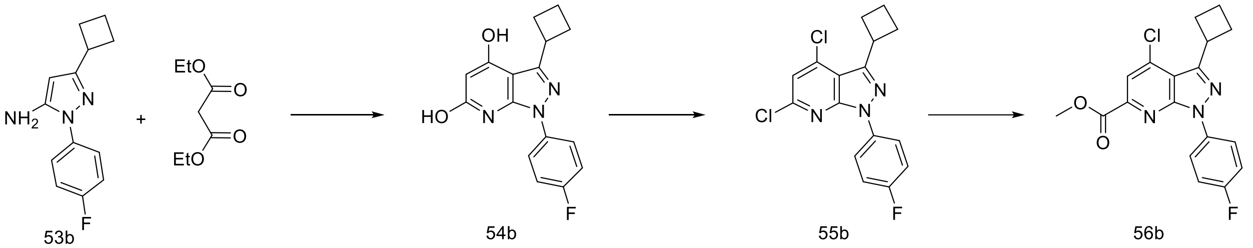

53b 54b 55b 56b

3-Cyclobutyl-1-(4-fluorophenyl)-1H-pyrazolo[3,4-b]pyridine-4,6-diol 54b

A mixture of 3-cyclobutyl-1-(4-fluorophenyl)-1*H*-pyrazol-5-amine **53b** (5 g, 18.6 mmol) and diethyl malonate ([105-53-3], 8.5 mL, 55.8 mmol) was heated at 100 °C for 30 minutes and then at 170 °C for 3 hours. The reaction mixture was cooled down to RT and dissolved in dichloromethane (60 mL). The resultant solution was poured into a stirred solution of *n*-heptane (700 mL). The precipitate was collected by filtration, washed with *n*-heptane and dried at 40 °C under reduced pressure to give the titled compound **54b** (5,29 g, 95 %). LCMS: m/z = 300.3 (M+H)+. 1H NMR (400 MHz, DMSO) δ 11.35 (s, 1H), 8.31 – 8.11 (m, 2H), 7.42 – 7.25 (m, 2H), 5.87 (s, 1H), 3.97 – 3.84 (m, 1H), 2.46 – 2.26 (m, 4H), 2.06 – 1.96 (m, 1H), 1.93 – 1.80 (m, 1H).

4,6-Dichloro-3-cyclobutyl-1-(4-fluorophenyl)-1H-pyrazolo[3,4-b]pyridine 55b

A three-neck round-bottom flask equipped with a Dean-Stark apparatus was charged with phenyl dichlorophosphate ([770-12-7], 854 g, 4.05 mol). 3-Cyclobutyl-1-(4-fluorophenyl)-1*H*-pyrazolo[3,4-*b*]pyridine-4,6-diol **54b** (404 g, 1.35 mol) was added in portions over a period of 5 minutes. The temperature was increased to 170 °C over a period of 1 hour, and the stirring at 170 °C was continued for 21 hours. The reaction mixture was cooled down to 50 °C and added slowly to a stirred aqueous 4 N NaOH (5 L) keeping the temperature below 20 °C. The suspension was stirred for 1 hour at 10-15 °C, and then cold water (3 L) was added. The precipitate was collected by filtration, washed with water and dried at 40 °C under reduced pressure to give the titled compound **55b** (385 g, 85 %). LCMS: m/z = 337.3 (M+H)+. ^{1}H NMR (400 MHz, CDCl3) δ 8.24 – 8.15 (m, 2H), 7.26 – 7.19 (m, 2H), 7.18 (s, 1H), 4.23 – 4.09 (m, 1H), 2.64 – 2.43 (m, 4H), 2.23 – 2.08 (m, 1H), 2.08 – 1.95 (m, 1H).

Methyl 4-chloro-3-cyclobutyl-1-(4-fluorophenyl)-1H-pyrazolo[3,4-b]pyridine-6-carboxylate 56b

A pressured vessel was charged with 4,6-dichloro-3-cyclobutyl-1-(4-fluorophenyl)-1*H*-pyrazolo[3,4-*b*]pyridine **55b** (5 g, 14.9 mmol), Pd(dppf)Cl_2•DCM (CAS 95464-05-4, 218 mg, 0.3 mmol), and sodium acetate (1.8 g, 22.3 mmol) in dioxane/methanol (1:1, 25 mL). The system was loaded with CO (4 bars) and heated at 40 °C for 2 hours. The vessel was cooled to RT, and the conversion was monitored by LCMS. The reaction vessel was charged again with CO (4 bars) and heated at 40 °C. The sequence was repeated until full conversion was observed. The crude mixture was concentrated under reduced pressure and purified by flash column chromatography eluting with a mixture of *n*-heptane/dichloromethane (90/10 to 30/70) to give the titled compound **56b** (3.38 g, 63%). LCMS: m/z = 360.2 (M+H)+. ^{1}H NMR (400 MHz, DMSO) δ 8.31 – 8.17 (m, 2H), 7.94 (s, 1H), 7.53 – 7.40 (m, 2H), 4.25 – 4.10 (m, 1H), 3.95 (s, 3H), 2.49 – 2.41 (m, 4H), 2.19 – 2.04 (m, 1H), 2.01 – 1.87 (m, 1H).

Scheme S38. Synthesis of 3-cyclobutyl-N-(methylsulfonyl)-1-phenyl-4-(piperidin-1-yl)-1H-pyrazolo[3,4-b]pyridine-6-carboxamide (46)

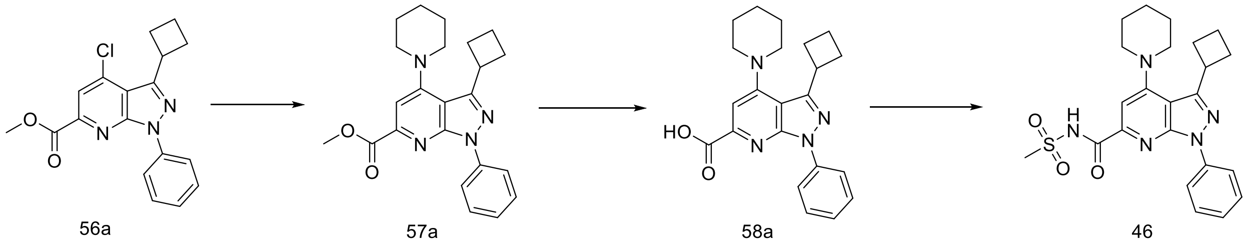

Methyl 3-cyclobutyl-1-phenyl-4-(piperidin-1-yl)-1H-pyrazolo[3,4-b]pyridine-6-carboxylate 57a

Methyl 4-chloro-3-cyclobutyl-1-phenyl-1H-pyrazolo[3,4-b]pyridine-6-carboxylate **56a** (50 mg, 0.15 mmol), piperidine (25 mg, 0.29 mmol) and DIPEA (26 µL, 0.15 mmol) in acetonitrile (1 mL) were treated according to general procedure H to afford the desired product **57a** (43 mg, 74%). LCMS: m/z = 391.4 (M+H)+.

3-Cyclobutyl-1-phenyl-4-(piperidin-1-yl)-1H-pyrazolo[3,4-b]pyridine-6-carboxylic acid 58a

Methyl 3-cyclobutyl-1-phenyl-4-(piperidin-1-yl)-1H-pyrazolo[3,4-b]pyridine-6-carboxylate **57a** (43 mg, 0.11 mmol) and aqueous 1 N sodium hydroxide (440 µL, 0.44 mmol) in a mixture of methanol / THF (1 mL, 1 / 1) were treated according to general procedure **I** to afford the desired product **58a** (35 mg, 85%). LCMS: m/z = 377.3 (M+H)+. ^{1}H NMR (400 MHz, CDCl3) δ 8.13 – 8.05 (m, 2H), 7.63 – 7.53 (m, 2H), 7.50 (s, 1H), 7.47 – 7.35 (m, 1H), 4.11 – 3.98 (m, 1H), 3.37 – 3.23 (m, 4H), 2.67 (m, 2H), 2.53-2.40 (m, 2H), 2.21 – 2.03 (m, 2H), 1.94-1.84 (m, 4H), 1.82-1.72 (m, 2H).

3-Cyclobutyl-N-(methylsulfonyl)-1-phenyl-4-(piperidin-1-yl)-1H-pyrazolo[3,4-b]pyridine-6-carboxamide (46)

3-Cyclobutyl-1-phenyl-4-(piperidin-1-yl)-1H-pyrazolo[3,4-b]pyridine-6-carboxylic acid **58a** (30 mg, 0.08 mmol), methanesulfonamide (15 mg, 0.16 mmol), EDC•HCl (21 mg, 0.11 mmol) and 4-(dimethylamino) pyridine (2 mg, 0.016 mmol) in dichloromethane (1 mL) were treated according to general procedure J to afford the desired product **46** (11 mg, 30%). LCMS: m/z = 454.4 (M+H)+. ^{1}H NMR(400 MHz, methanol-d_4) δ ppm 8.22 (d, J=8.4 Hz, 2 H), 7.59 (t, J=10.3 Hz, 2 H), 7.46 (s, 1 H), 7.40 (t, J=7.3 Hz, 1H), 4.13 (t, J=8.3 Hz, 2 H), 3.37 - 3.37 (m, 2 H), 2.58 - 2.70 (m, 3 H), 2.50 (d, J=8.1 Hz, 2 H), 2.01 - 2.23 (m, 2 H), 1.85 - 1.98 (m, 4 H), 1.79 (d, J=4.0 Hz, 2 H).

Scheme S39. Synthesis of 3-cyclobutyl-4-(4-methoxypiperidin-1-yl)-N-(methylsulfonyl)-1-phenyl-1H-pyrazolo[3,4-b]pyridine-6-carboxamide (47)

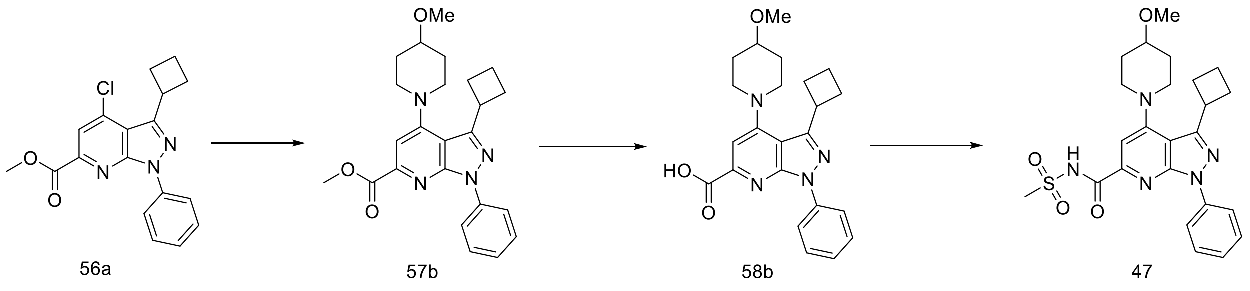

Methyl 3-cyclobutyl-4-(4-methoxypiperidin-1-yl)-1-phenyl-1H-pyrazolo[3,4-b]pyridine-6-carboxylate 57b

Methyl 4-chloro-3-cyclobutyl-1-phenyl-1H-pyrazolo[3,4-b]pyridine-6-carboxylate **56a** (500 mg, 1.46 mmol), 4-methoxypiperidine (544 µL, 4.39 mmol) and DIPEA (764 µL, 4.39 mmol) in acetonitrile (10 mL) were treated according to general procedure **H** to afford the desired product **57b** (499 mg, 81%). LCMS: m/z = 421.6 (M+H)+. ^{1}H NMR (400 MHz, CDCl3) δ 8.37 – 8.30 (m, 2H), 7.56 – 7.47 (m, 2H), 7.41 (s, 1H), 7.32 – 7.23 (m, 1H), 4.09 – 3.96 (m, 2H), 4.00 (s, 3H), 3.50 (m, 4H), 3.44 (s, 3H), 3.06 (m, 2H), 2.64 (m, 2H), 2.48 – 2.36 (m, 1H), 2.14 (s, 2H), 2.20 – 2.01 (m, 1H), 1.89 (m, 2H).

3-Cyclobutyl-4-(4-methoxypiperidin-1-yl)-1-phenyl-1H-pyrazolo[3,4-b]pyridine-6-carboxylic acid 58b

Methyl 3-cyclobutyl-4-(4-methoxypiperidin-1-yl)-1-phenyl-1H-pyrazolo[3,4-b]pyridine-6-carboxylate **57b** (499 mg, 1.19 mmol) and aqueous 1 N sodium hydroxide (2.37 mL, 4.75 mmol) in a mixture of methanol / THF (10 mL, 1 / 1) were treated according to general procedure I to afford the desired product **58b** (478 mg, 99%). LCMS: m/z = 407.5 (M+H)+. 1H NMR (400 MHz, CDCl3) δ 8.08 – 8.00 (m, 2H), 7.59 – 7.50 (m, 2H), 7.47 (s, 1H), 7.36 (m, 1H), 4.00 (m, 1H), 3.55 (m, 3H), 3.44 (s, 3H), 3.14 (m, 2H), 2.63 (m, 2H), 2.49 – 2.36 (m, 1H), 2.20 – 2.01 (m, 2H), 1.89 (m, 2H).

3-Cyclobutyl-4-(4-methoxypiperidin-1-yl)-N-(methylsulfonyl)-1-phenyl-1H-pyrazolo[3,4-b]pyridine-6-carboxamide (47)

3-Cyclobutyl-4-(4-methoxypiperidin-1-yl)-1-phenyl-1H-pyrazolo[3,4-b]pyridine-6-carboxylic acid **58b** (20 mg, 0.05 mmol), methanesulfonamide (9 mg, 0.1 mmol), EDC•HCl (11.3 mg, 0.06 mmol) and 4-(dimethylamino) pyridine (1.2 mg, 0.01 mmol) in dichloromethane (1 mL) were treated according to general procedure J to afford the desired product **47** (17 mg, 71%). LCMS: m/z = 484.6 (M+H)+. ^{1}H NMR (400 MHz, CDCl3) δ 9.83 (s, 1H), 7.87 – 7.79 (m, 2H), 7.40 – 7.28 (m, 2H), 7.20 – 7.11 (m, 1H), 3.86 – 3.73 (m, 1H), 3.33 (m, 3H), 3.22 (d, J = 13.8 Hz, 6H), 2.91 (m, 2H), 2.42 (m, 2H), 2.28 – 2.09 (m, 1H), 1.99 – 1.78 (m, 3H), 1.68 (m, 2H), 1.12 – 1.01 (m, 2H).

Scheme S40. Synthesis of 4-(4-cyanopiperidin-1-yl)-3-cyclobutyl-N-(methylsulfonyl)-1-phenyl-1H-pyrazolo[3,4-b]pyridine-6-carboxamide (48)

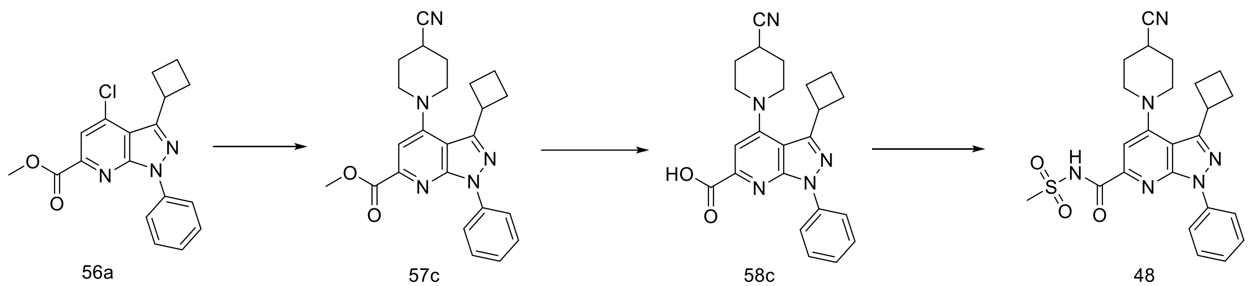

Methyl 4-(4-cyanopiperidin-1-yl)-3-cyclobutyl-1-phenyl-1H-pyrazolo[3,4-b]pyridine-6-carboxylate 57c

Methyl 4-chloro-3-cyclobutyl-1-phenyl-1H-pyrazolo[3,4-b]pyridine-6-carboxylate **56a** (50 mg, 0.15 mmol), 4-cyanopiperidine (32 mg, 0.29 mmol) and DIPEA (26 µL, 0.15 mmol) in acetonitrile (1 mL) were treated according to general procedure H to afford the desired product **57c** (29 mg, 47%). LCMS: m/z = 416.4 (M+H)+.

4-(4-cyanopiperidin-1-yl)-3-cyclobutyl-1-phenyl-1H-pyrazolo[3,4-b]pyridine-6-carboxylic acid 58c

Methyl 4-(4-cyanopiperidin-1-yl)-3-cyclobutyl-1-phenyl-1H-pyrazolo[3,4-b]pyridine-6-carboxylate **57c** (29 mg, 0.07 mmol) and aqueous 1 N sodium hydroxide (280 µL, 0.28 mmol) in a mixture of methanol / THF (1 mL, 1 / 1) were treated according to general procedure I to afford the desired product **58c** (27 mg, 96%). LCMS: m/z = 402.4 (M+H)+. 1H NMR (400 MHz, CDCl3) δ 8.14 – 8.02 (m, 2H), 7.59 (dd, J = 8.6, 7.4 Hz, 2H), 7.53 (s, 1H), 7.44 – 7.38 (m, 1H), 4.06 – 3.92 (m, 1H), 3.66 – 3.52 (m, 2H), 3.34 – 3.21 (m, 2H), 3.05 – 2.94 (m, 1H), 2.76 – 2.61 (m, 2H), 2.54 – 2.40 (m, 2H), 2.34 – 2.06 (m, 6H).

4-(4-cyanopiperidin-1-yl)-3-cyclobutyl-N-(methylsulfonyl)-1-phenyl-1H-pyrazolo[3,4-b]pyridine-6-carboxamide (48)

4-(4-cyanopiperidin-1-yl)-3-cyclobutyl-1-phenyl-1H-pyrazolo[3,4-b]pyridine-6-carboxylic acid **58c** (25 mg, 0.06 mmol), methanesulfonamide (12 mg, 0.12 mmol), EDC•HCl (17 mg, 0.09 mmol) and 4-(dimethylamino) pyridine (1.5 mg, 0.01 mmol) in dichloromethane (1 mL) were treated according to general procedure J to afford the desired product **48** (19 mg, 64%). LCMS: m/z = 479.2 (M+H)+. ^{1}H NMR(400 MHz, methanol-*d₄*) δ ppm 8.23 (d, J=8.2 Hz, 2H), 7.60 (dt, J=2.0, 7.6 Hz, 2H), 7.51 (s, 1H), 7.39 (t, J=7.4 Hz, 1H), 4.15 - 4.06 (m, 1H), 3.58 - 3.50 (m, 2H), 3.39 (s,

3H), 3.31 - 3.23 (m, 3H), 3.18 - 3.14 (m, 1H), 2.67 - 2.61 (m, 2H), 2.56 - 2.48 (m, 2H), 2.32 - 2.26 (m, 2H), 2.20 - 2.07 (m, 4H).

Scheme S41. Synthesis of 3-cyclobutyl-4-(4-morpholinopiperidin-1-yl)-1-phenyl-1H-pyrazolo[3,4-b]pyridine-6-carboxylic acid 58d

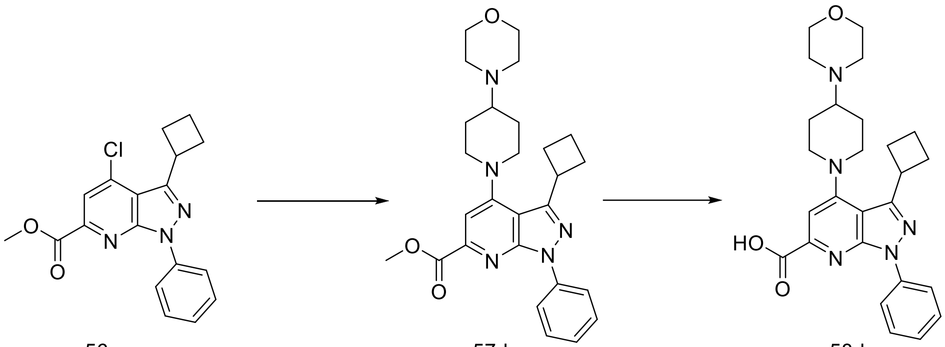

56a 57d 58d

Methyl 3-cyclobutyl-4-(4-morpholinopiperidin-1-yl)-1-phenyl-1H-pyrazolo[3,4-b]pyridine-6-carboxylate 57d

To a solution of Methyl 4-chloro-3-cyclobutyl-1-phenyl-1H-pyrazolo[3,4-b]pyridine-6-carboxylate **56a** (1 g, 2.92 mmol) in acetonitrile (10 mL) was added 4-piperidin-4-ylmorpholine (0.75 g, 4.38 mmol, CAS 53617-35-9) and diisopropylethylamine (2.19 mL, 12.5 mmol). The reaction mixture was refluxed for 20 hours and then cooled down to room temperature. The precipitate was filtered, washed with diethyl ether, and dried at 40°C under reduced pressure to yield 1.23 g (88%) of **57d** as a beige solid. LCMS: m/z = 476.5 (M+H)+.

3-Cyclobutyl-4-(4-morpholinopiperidin-1-yl)-1-phenyl-1H-pyrazolo[3,4-b]pyridine-6-carboxylic acid 58d

To methyl 3-cyclobutyl-4-(4-morpholinopiperidin-1-yl)-1-phenyl-1H-pyrazolo[3,4-b]pyridine-6-carboxylate **57d** (1.23 g, 2.58 mmol) in a mixture of THF/methanol 2/1 (18 mL) was added aqueous 1 N sodium hydroxide (3.1 mL, 3.35 mmol). The reaction mixture was heated at 60 °C for 1 hour and left cooling down to room temperature. The precipitate was filtered and successively washed with water and diethyl ether. The compound was dried at 40°C under reduced pressure to afford 0.98 g of the titled compound **58d** (82%) as a beige solid. LCMS: m/z = 462.4 (M+H)+. ^{1}H

NMR (400 MHz, DMSO) δ 13.31 (s, 1H), 8.34 (d, J = 7.5 Hz, 2H), 7.57 (t, J = 7.4 Hz, 2H), 7.37 – 7.28 (m, 2H), 4.00 (p, J = 8.3 Hz, 1H), 3.67 – 3.53 (m, 6H), 2.93 (t, J = 11.9 Hz, 2H), 2.60 – 2.55 (m, 3H), 2.47 – 2.34 (m, 4H), 2.15 – 1.92 (m, 4H), 1.75 – 1.60 (m, 2H).

Scheme S42. Synthesis of 3-cyclobutyl-N-(methylsulfonyl)-4-(4-morpholinopiperidin-1-yl)-1-phenyl-1H-pyrazolo[3,4-b]pyridine-6-carboxamide (49)

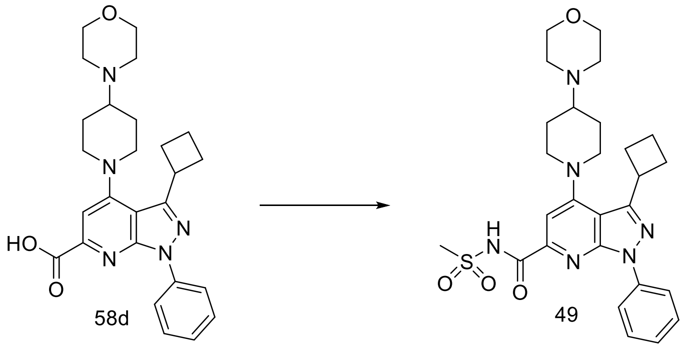

3-cyclobutyl-N-(methylsulfonyl)-4-(4-morpholinopiperidin-1-yl)-1-phenyl-1H-pyrazolo[3,4-b]pyridine-6-carboxamide (49)

A 3-neck round bottom flask (10 L) was charged with 3-Cyclobutyl-4-(4-morpholinopiperidin-1-yl)-1-phenyl-1H-pyrazolo[3,4-b]pyridine-6-carboxylic acid **58d** (431 g, 934 mmol) and dry DMF (3.4 L). 1,1′-Carbonyldiimidazole (374 g, 2054 mmol) was added in one portion and the mixture was heated to 83 °C in 40 minutes. The solution was cooled down to 0-5°C in an ice/water bath and methanesulfonamide (178 g, 1868 mmol) was added in one portion, followed by 1,8-Diazabicyclo[5.4.0]undec-7-ene (307 mL, 2054 mmol) via a dropping funnel over a 10 minutes period. The cooling bath was removed, and the mixture was allowed to warm to room temperature for 45 minutes. The reaction mixture was cooled down to 5 °C in an ice/water bath. Concentrated HCl (646 mL, 7321 mmol) was added drop wise over one hour period. Water (3.4 L) was added drop wise over a one-hour period keeping the temperature below 21°C. The reaction mixture was stirred for one hour and the precipitate was filtered, washed with water (3.4 L) and dried in vacuo at 40 °C for 20 hours to afford the desired compound **49** as a yellow solid (530 g, 100% yield). LCMS: m/z = 539.6 (M+H)+. ^{1}H NMR(500 MHz, DMSO-J6) δ ppm 8.37-8.33 (m, 2H), 7.59-7.54 (m, 2H), 7.37-7.33 (m, 1H), 7.30 (s, 1H), 3.99 (tt, J =8.3, 8.3 Hz, 1H), 3.71-3.65 (m, 4H), 3.65-

3.59 (m, 2H), 3.24 (s, 3H), 3.18-3.16 (m, 1H), 2.98-2.91 (m, 2H), 2.80-2.62 (m, 5H), 2.56-2.39 (m, 3H), 2.13-1.94 (m, 4H), 1.78-1.68 (m, 2H).

Scheme S43. Synthesis of 3-cyclobutyl-1-(4-fluorophenyl)-N-(methylsulfonyl)-4-(4-morpholinopiperidin-1-yl)-1H-pyrazolo[3,4-b]pyridine-6-carboxamide hydrochloride (50).

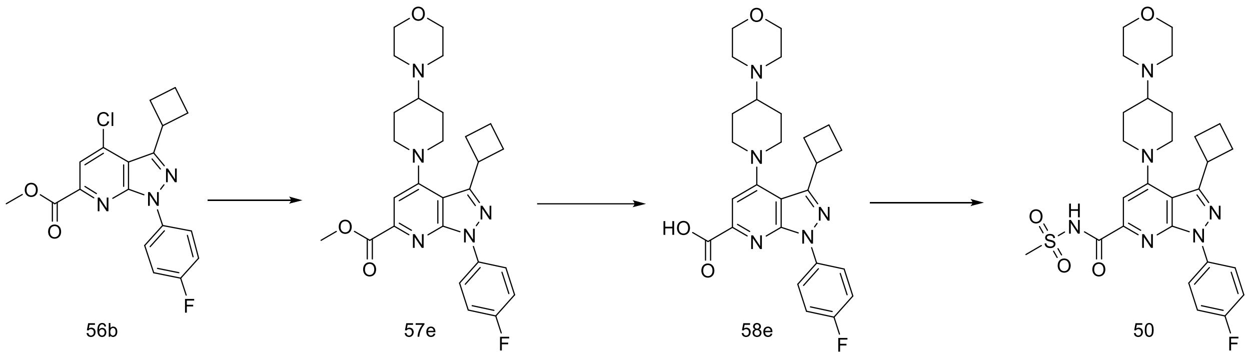

Methyl 3-cyclobutyl-1-(4-fluorophenyl)-4-(4-morpholinopiperidin-1-yl)-1H-pyrazolo[3,4-b]pyridine-6-carboxyate 57e

Methyl 4-chloro-3-cyclobutyl-1-(4-fluorophenyl)-1H-pyrazolo[3,4-b]pyridine-6-carboxylate **56b** (1 g, 2.78 mmol), 4-(piperidin-4-yl)morpholine (945 mg, 5.56 mmol) and DIPEA (1.45 mL, 8.34 mmol) in acetonitrile (10 mL) were treated according to general procedure H to afford the desired product **57e** (1.29 g, 94%). LCMS: m/z = 494.6 (M+H)+. ^{1}H NMR (400 MHz, CDCl3) δ 8.37 – 8.27 (m, 2H), 7.42 (s, 1H), 7.27 – 7.17 (m, 2H), 4.02 (3, 4H), 3.82 (m, 4H), 3.70 (d, J = 12.3 Hz, 2H), 2.93 (m, 2H), 2.69 (t, J = 4.6 Hz, 4H), 2.62 (m, 2H), 2.49 – 2.36 (m, 2H), 2.19 – 2.02 (m, 4H), 1.84 (m, 2H).

3-cyclobutyl-1-(4-fluorophenyl)-4-(4-morpholinopiperidin-1-yl)-1H-pyrazolo[3,4-b]pyridine-6-carboxylic acid 58e

Methyl-3-cyclobutyl-1-(4-fluorophenyl)-4-(4-morpholinopiperidin-1-yl)-1H-pyrazolo[3,4-b]pyridine-6-carboxyate **57e** (5.46 mg, 11.1 mmol) and aqueous 2 N sodium hydroxide (11 mL, 22.1 mmol) in THF (80 mL) were treated according to general procedure I to afford the desired product **58e** (5.46 mg, quantitative). LCMS: m/z = 480.6 (M+H)+. ^{1}H NMR (400 MHz, DMSO) δ 8.40 – 8.30 (m, 2H), 7.47 – 7.36 (m, 2H), 7.31 (s, 1H), 3.98 (m, 1H), 3.65 – 3.54 (m, 7H), 2.91

(t, J = 11.9 Hz, 2H), 2.55 (t, J = 4.7 Hz, 4H), 2.50 – 2.41 (m, 1H), 2.44 – 2.37 (m, 3H), 2.15 – 2.02 (m, 1H), 2.00 (s, 3H), 1.69 (m, 1H), 1.64 (m, 1H).

3-cyclobutyl-1-(4-fluorophenyl)-N-(methylsulfonyl)-4-(4-morpholinopiperidin-1-yl)-1H-pyrazolo[3,4-b]pyridine-6-carboxamide hydrochloride (50).

3-cyclobutyl-1-(4-fluorophenyl)-4-(4-morpholinopiperidin-1-yl)-1H-pyrazolo[3,4-b]pyridine-6-carboxylic acid **58e** (100 mg, 0.21 mmol), methanesulfonamide (79 mg, 0.83 mmol), EDC•HCl (48 mg, 0.25 mmol) and 4-(dimethylamino) pyridine (5 mg, 0.04 mmol) in dichloromethane (2 mL) were treated according to general procedure J to afford the free base (23 mg, 19%). The compound was solubilized in dichloromethane (1 mL) and 0.1N HCl in diethyl ether (0.42 mL, 0.04 mmol) was added to the stirring solution. After 10 minutes, n-pentane was added until a precipitate was formed. The precipitate was filtered and dried under reduced pressure to afford the titled compound **50** as a HCl salt (20 mg, 80%). LCMS: m/z = 557.7 (M+H)+. ^{1}H NMR(400 MHz, CDCl3) (400 MHz, CDCl3) δ ppm 9.94 (br. s, 1 H), 7.96 - 8.00 (m, 2 H), 7.47 (s, 1 H), 7.26 - 7.31 (m, 2 H), 4.45 - 4.51 (t, 2H), 4.06 - 4.10 (m., 2 H), 3.92-3.97 (m, 1 H), 3.83 - 3.87 (m., 2 H), 3.50 - 3.56 (m, 2 H), 3.43 (s, 3 H), 3.22 -3.31 (m, 1 H), 3.00-3.11 (m, 4H), 2.56-2.67 (m, 2 H), 2.45 - 2.52 (m, 4 H), 2.28 - 2.37 (m, 2 H), 2.06 - 2.21 (m, 2 H).

Scheme S44. Synthesis of 3-cyclobutyl-N-(N,N-dimethylsulfamoyl)-1-(4-fluorophenyl)-4-(4-morpholinopiperidin-1-yl)-1H-pyrazolo[3,4-b]pyridine-6-carboxamide (51)

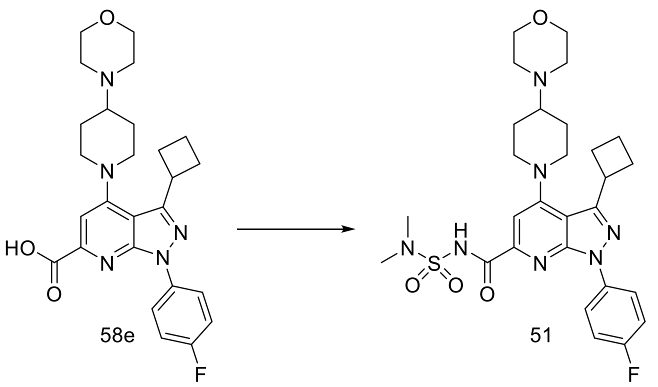

3-cyclobutyl-N-(N,N-dimethylsulfamoyl)-1-(4-fluorophenyl)-4-(4-morpholinopiperidin-1-yl)-1H-pyrazolo[3,4-b]pyridine-6-carboxamide (51)

EDC.HCl (6 g, 31.5 mmol) was added at room temperature to a stirring solution of 3-cyclobutyl-1-(4-fluorophenyl)-4-(4-morpholinopiperidin-1-yl)-1H-pyrazolo[3,4-b]pyridine-6-carboxylic acid **58e** (12.58 g, 26.2 mmol), N,N-dimethylsulfamide (3.15 g, 28.9 mmol) and 4-(dimethylamino) pyridine (6.4 g, 52.5 mmol) in dichloromethane (260 mL) and DMF (10 mL). The reaction mixture was stirred at room temperature for 20 hours. The reaction mixture was diluted with dichloromethane and washed with aqueous 1 M citric acid. The aqueous phase was extracted twice with dichloromethane. The combined organic phases were washed with an aqueous saturated NaCl solution, dried over sodium sulfate and concentrated in vacuo. The residue was purified by flash column chromatography eluting with dichloromethane / methanol to yield the desired compound **51** (6.1 g, 44%). LCMS: m/z = 586.4 (M+H)+. 1H NMR (400 MHz, CDCl3) δ 8.07 – 7.99 (m, 2H), 7.47 (s, 1H), 7.31 – 7.22 (m, 3H), 4.00 (p, J = 8.4 Hz, 1H), 3.82 (s, 4H), 3.73 (d, J = 12.5 Hz, 2H), 3.06 (s, 6H), 3.02 – 2.92 (m, 2H), 2.75 – 2.56 (m, 6H), 2.51 – 2.38 (m, 3H), 2.20 – 2.02 (m, 4H), 1.91 – 1.77 (m, 2H).

Scheme S45. Synthesis of 3-cyclobutyl-N-(N,N-dimethylsulfamoyl)-1-(4-fluorophenyl)-4-(4-methoxy-[1,4'-bipiperidin]-1'-yl)-1H-pyrazolo[3,4-b]pyridine-6-carboxamide (52) or GLPG2737

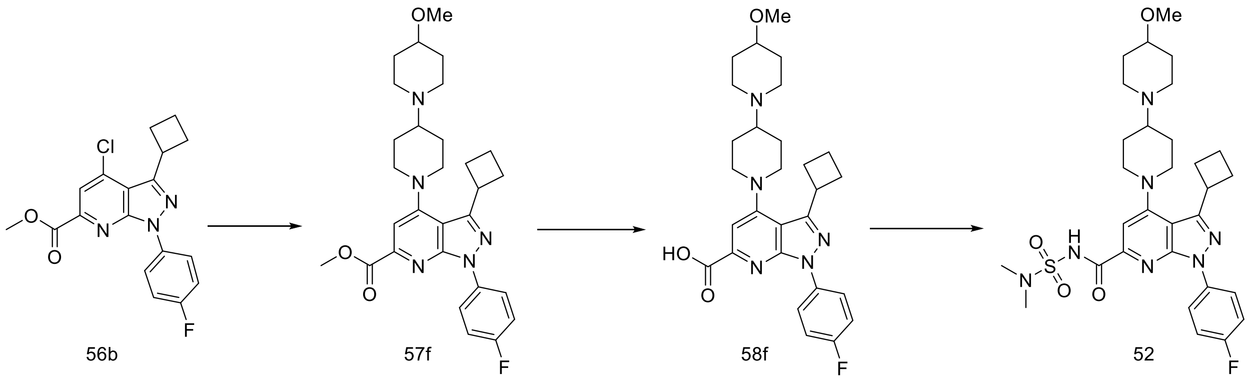

Methyl 3-cyclobutyl-1-(4-fluorophenyl)-4-(4-methoxy-[1,4'-bipiperidin]-1'-yl)-1H-pyrazolo[3,4-b]pyridine-6-carboxylate 57f

To a solution of methyl 4-chloro-3-cyclobutyl-1-(4-fluorophenyl)-1H-pyrazolo[3,4-b]pyridine-6-carboxylate **56b** (1 g, 2.78 mmol) in DMSO (10 mL) was added 4-methoxy-1,4'-bipiperidine (1.5 g, 5.56 mmol) and triethylamine (1.55 g, 11.12 mmol). The reaction mixture was stirred at 100 °C for 24 hours and then cooled down to ambient temperature and diluted with water (100 mL). The suspension was stirred for 20 hours, filtered, and was washed with water. The precipitate

was dried at 40°C under reduced pressure to give the titled compound **57f** (670 mg, 46%). LCMS: m/z = 522.4 (M+H)+. 1H NMR(400 MHz, DMSO-d6) δ 8.34 – 8.25 (m, 2H), 7.42 (t, J = 8.8 Hz, 2H), 7.29 (s, 1H), 4.04 – 3.93 (m, 1H), 3.91 (s, 3H), 3.60 (d, J = 12.1 Hz, 2H), 3.24 (s, 3H), 3.21 – 3.13 (m, 1H), 2.92 (t, J = 11.9 Hz, 2H), 2.87 – 2.77 (m, 2H), 2.46 – 2.36 (m, 4H), 2.35 – 2.22 (m, 3H), 2.14 – 1.81 (m, 6H), 1.78 – 1.61 (m, 2H), 1.49 – 1.34 (m, 2H).

3-Cyclobutyl-1-(4-fluorophenyl)-4-(4-methoxy-[1,4'-bipiperidin]-1'-yl)-1H-pyrazolo[3,4-b]pyridine-6-carboxylic acid 58f

Methyl 3-cyclobutyl-1-(4-fluorophenyl)-4-(4-methoxy-[1,4'-bipiperidin]-1'-yl)-1H-pyrazolo[3,4-b]pyridine-6-carboxylate **57f** (22.58 mmol) and lithium hydroxide monohydrate (1.9 g, 45.16 mmol) in a mixture of dioxane / water (200 mL, 2 / 1) were heated at 50°C for 1 hour. The reaction mixture was cooled down to room temperature and the volatile were removed in vacuo. The residue was diluted with water and acidified to pH = 5 with aqueous 1 M HCl. The precipitate was filtered, washed with water and dried at 40°C under reduced pressure to afford the desired product **58f** (9.02 g, 79%). LCMS: m/z = 508.0 (M+H)+. 1H NMR (400 MHz, CDCl3) δ 12.33 – 12.23 (m, 1H), 7.94 (m, 2H), 7.15 (m, 2H), 3.83 (p, J = 8.2 Hz, 1H), 3.74 (d, J = 12.6 Hz, 2H), 3.59 (s, 1H), 3.34 – 3.22 (m, 6H), 3.13 (q, J = 11.5 Hz, 2H), 2.97 (t, J = 12.3 Hz, 2H), 2.60 – 2.30 (m, 9H), 2.15 – 1.95 (m, 6H).

3-Cyclobutyl-N-(N,N-dimethylsulfamoyl)-1-(4-fluorophenyl)-4-(4-methoxy-[1,4'-bipiperidin]-1'-yl)-1H-pyrazolo[3,4-b]pyridine-6-carboxamide (52) or GLPG2737

A round bottom flask was charged with 3-Cyclobutyl-1-(4-fluorophenyl)-4-(4-methoxy-[1,4'-bipiperidin]-1'-yl)-1H-pyrazolo[3,4-b]pyridine-6-carboxylic acid **58f** (7.1 g, 13.94 mmol) in dry DMF (100 mL). 1,1′-Carbonyldiimidazole (5.42 g, 33.46 mmol) was added in one portion and the mixture was stirred at room temperature for 30 minutes. Methanesulfonamide (3.46 g, 27.88 mmol) was added followed by 1,8-Diazabicyclo[5.4.0]undec-7-ene (4.58 mL, 30.67 mmol). The mixture was stirred for 1 hour and then poured onto water (500 mL). The solution was acidified with 1 M citric acid until a persistent precipitate appear. The precipitate was filtrate, washed with water and dried in vacuo at 40 °C. The crude material was purified by flash column chromatography eluting with dichloromethane / methanol to afford the desired compound **52** as a yellow solid (5.71 g, 67% yield). LCMS: m/z = 614.5 (M+H)+. 1H NMR (400 MHz, CDCl3) δ 8.07 – 7.98 (m, 2H), 7.46 (s, 1H), 7.32 – 7.21 (m, 2H), 4.00 (p, J = 8.4 Hz, 1H), 3.73 (d, J = 12.2

Hz, 2H), 3.39 (s, 3H), 3.34 – 3.24 (m, 1H), 3.06 (s, 6H), 3.02 – 2.87 (m, 4H), 2.62 (dq, J = 11.5, 9.0 Hz, 2H), 2.55 – 2.35 (m, 5H), 2.24 – 2.03 (m, 5H), 1.97 (s, 2H), 1.91 – 1.77 (m, 2H), 1.75 – 1.60 (m, 2H).

III. HPLC traces of compounds 8-52

3-methyl-4-[4-(morpholin-4-yl)phenyl]-1-phenyl-1H-pyrazolo[3,4-b]pyridine-6-carboxylic acid (8)

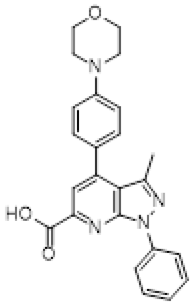

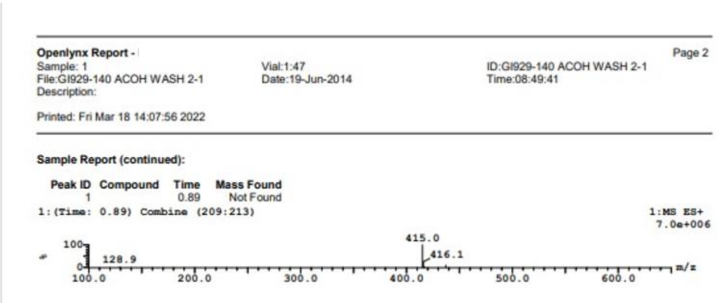
Openlynx Report -

Page 2

Sample: 1
File:GI929-140 ACOH WASH 2-1
Description:

Vial:1:47
Date:19-Jun-2014

ID:GI929-140 ACOH WASH 2-1
Time:08:49:41

Printed: Fri Mar 18 14:07:56 2022

Sample Report (continued):

Peak ID	Compound	Time	Mass Found
1		0.89	Not Found

1:(Time: 0.89) Combine (209:213)

1:MS ES+
7.0e+006

1-(3-fluorophenyl)-3-methyl-4-(4-morpholinophenyl)-1H-pyrazolo[3,4-b]pyridine-6-carboxylic acid (9)

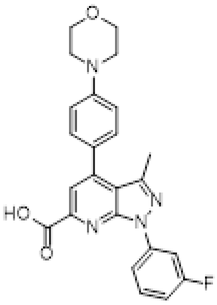

Openlynx Report - Adeline
Sample: 1
File:GI158-096-C-PR-1
Description:

Vial:2:3
Date:09-May-2014

ID:GI158-096-C-PR-1
Time:12:18:38

Page 1

Printed: Wed May 14 16:05:32 2014

Sample Report:

Sample 1 Vial 2:3 ID GI158-096-C-PR-1 File GI158-096-C-PR-1 Date 09-May-2014 Time 12:18:38 Description

1: MS ES+ :TIC Smooth (Mn, 1x1)

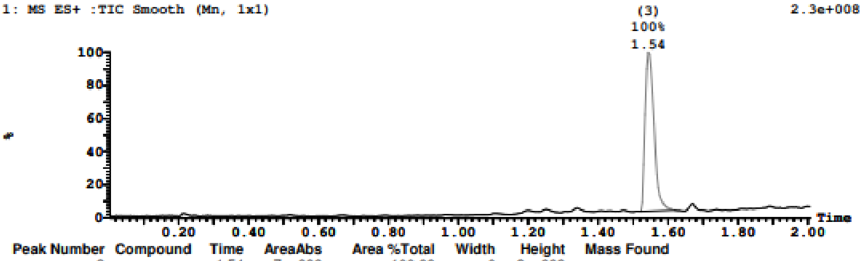

Peak Number	Compound	Time	AreaAbs	Area %Total	Width	Height	Mass Found
3		1.54	7e+006	100.00	0	2e+008	

2: UV Detector: TIC

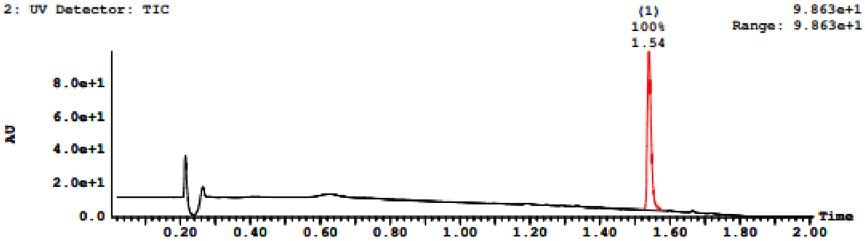

Peak Number	Compound	Time	AreaAbs	Area %Total	Width	Height	Mass Found
1		1.54	1e+006	100.00	0	9e+007	

2: UV Detector: 220

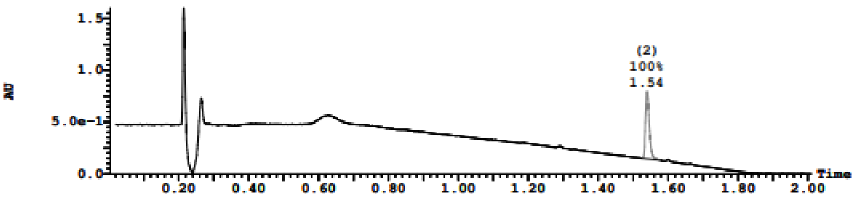

Peak Number	Compound	Time	AreaAbs	Area %Total	Width	Height	Mass Found
2		1.54	8e+003	100.00	0	7e+005	

Openlynx Report - Adeline
Sample: 1
File:GI158-096-C-PR-1
Description:

Vial:2:3
Date:09-May-2014

ID:GI158-096-C-PR-1
Time:12:18:38

Page 2

Printed: Wed May 14 16:05:32 2014

Sample Report (continued):

Peak ID	Compound	Time	Mass Found
1		1.54	

1:(Time: 1.54) Combine (364:369)

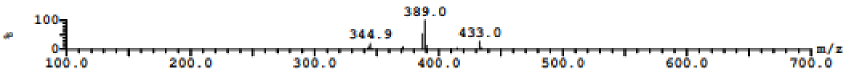

1-(4-fluorophenyl)-3-methyl-4-(4-morpholinophenyl)-1H-pyrazolo[3,4-b]pyridine-6-carboxylic acid (10).

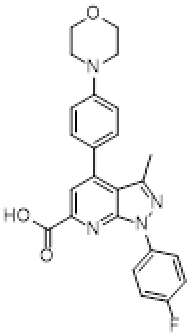

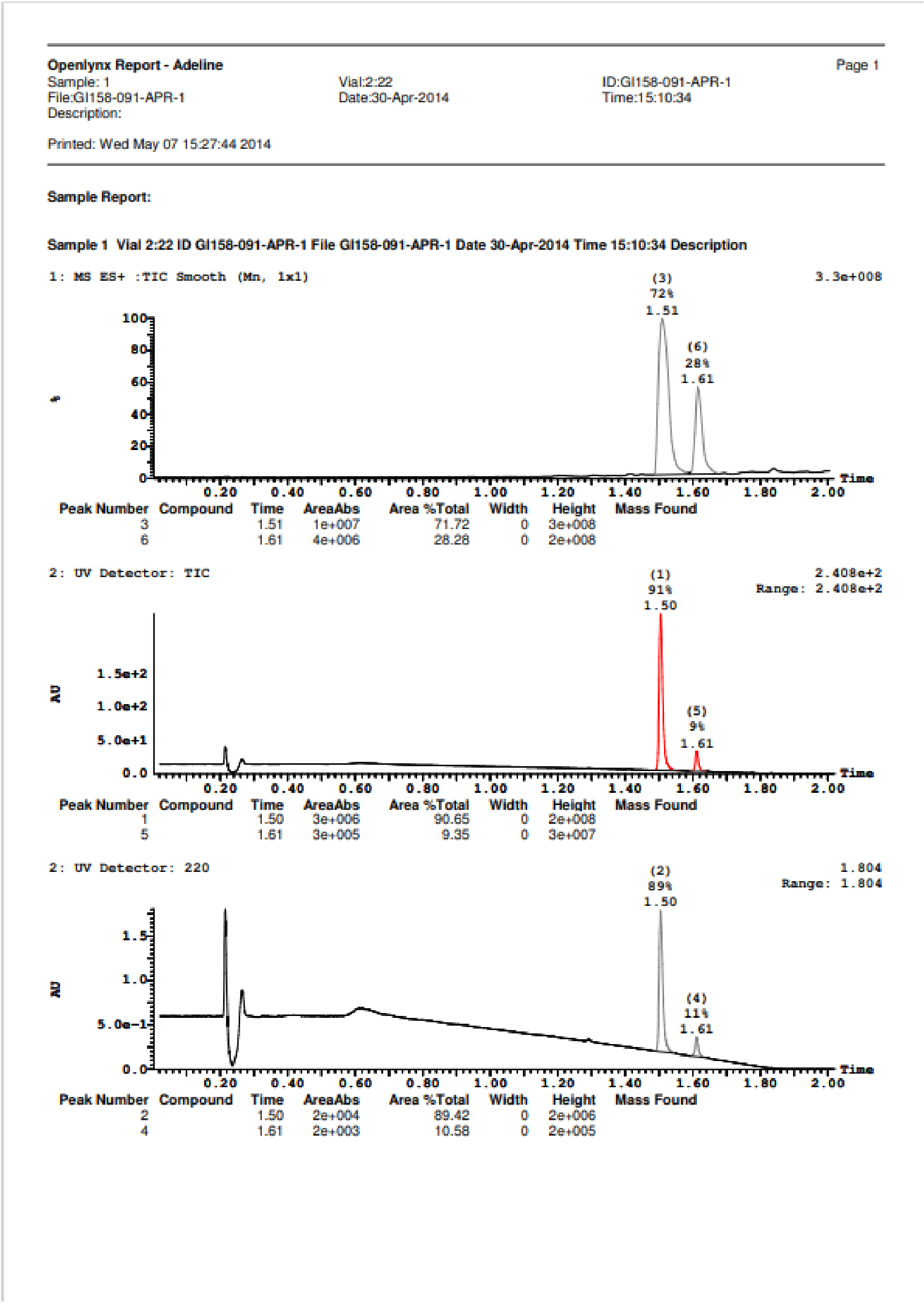

Openlynx Report - Adeline

Sample: 1 Vial:2:22 ID:GI158-091-APR-1 Page 1

File:GI158-091-APR-1 Date:30-Apr-2014 Time:15:10:34

Description:

Printed: Wed May 07 15:27:44 2014

Sample Report:

Sample 1 Vial 2:22 ID GI158-091-APR-1 File GI158-091-APR-1 Date 30-Apr-2014 Time 15:10:34 Description

Peak Number	Compound	Time	AreaAbs	Area %Total	Width	Height	Mass Found
3		1.51	1e+007	71.72	0	3e+008	
6		1.61	4e+006	28.28	0	2e+008	

Peak Number	Compound	Time	AreaAbs	Area %Total	Width	Height	Mass Found
1		1.50	3e+006	90.65	0	2e+008	
5		1.61	3e+005	9.35	0	3e+007	

Peak Number	Compound	Time	AreaAbs	Area %Total	Width	Height	Mass Found
2		1.50	2e+004	89.42	0	2e+006	
4		1.61	2e+003	10.58	0	2e+005	

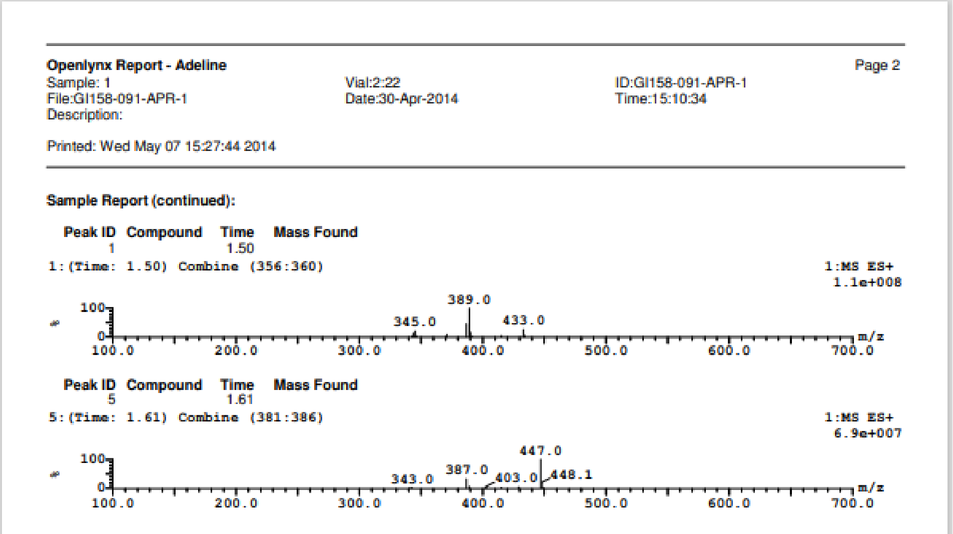

Openlynx Report - Adeline
Page 2
Sample: 1
Vial:2:22
ID:GI158-091-APR-1
File:GI158-091-APR-1
Date:30-Apr-2014
Time:15:10:34
Description:
Printed: Wed May 07 15:27:44 2014
Sample Report (continued):
Peak ID Compound Time Mass Found
1 1.50
1: (Time: 1.50) Combine (356:360)
1:MS ES+
1.1e+008
389.0
345.0
433.0
100.0 200.0 300.0 400.0 500.0 600.0 700.0
m/z
Peak ID Compound Time Mass Found
5 1.61
5: (Time: 1.61) Combine (381:386)
1:MS ES+
6.9e+007
447.0
343.0
387.0
403.0
448.1
100.0 200.0 300.0 400.0 500.0 600.0 700.0
m/z

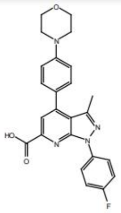

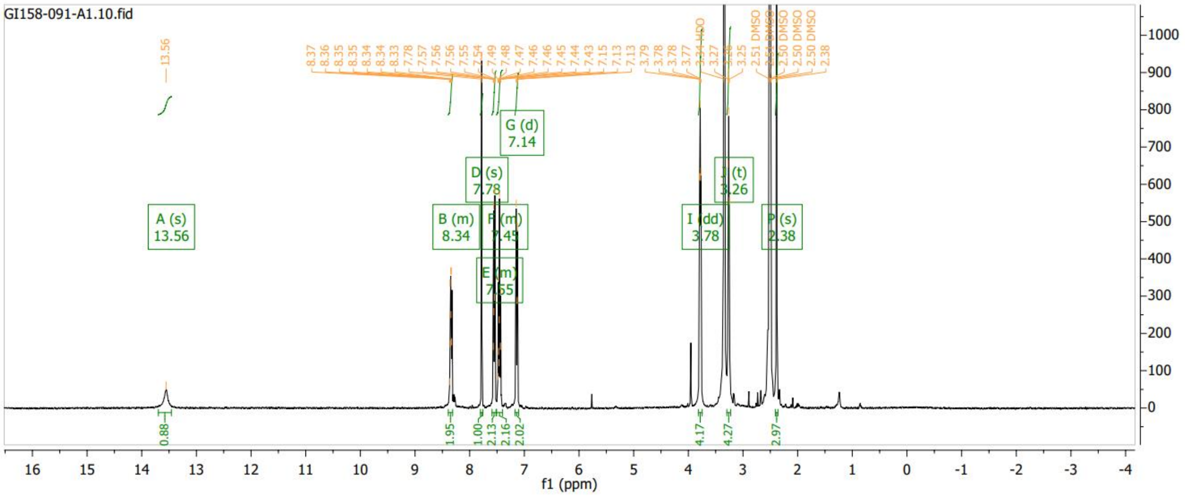

GI158-091-A1.10.fid
A (s)
13.56
B (m)
8.34
D (s)
7.78
E (m)
7.55
F (m)
7.49
G (d)
7.14
I (dd)
3.78
J (t)
3.26
P (s)
2.38
0.88
1.95
1.00
2.13
2.16
2.02
4.17
4.27
2.97
f1 (ppm)

1-cyclopropyl-3-methyl-4-(4-morpholinophenyl)-1H-pyrazolo[3,4-b]pyridine-6-carboxylic acid (11)

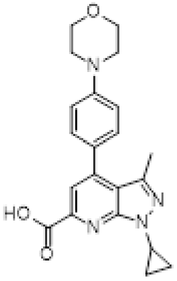

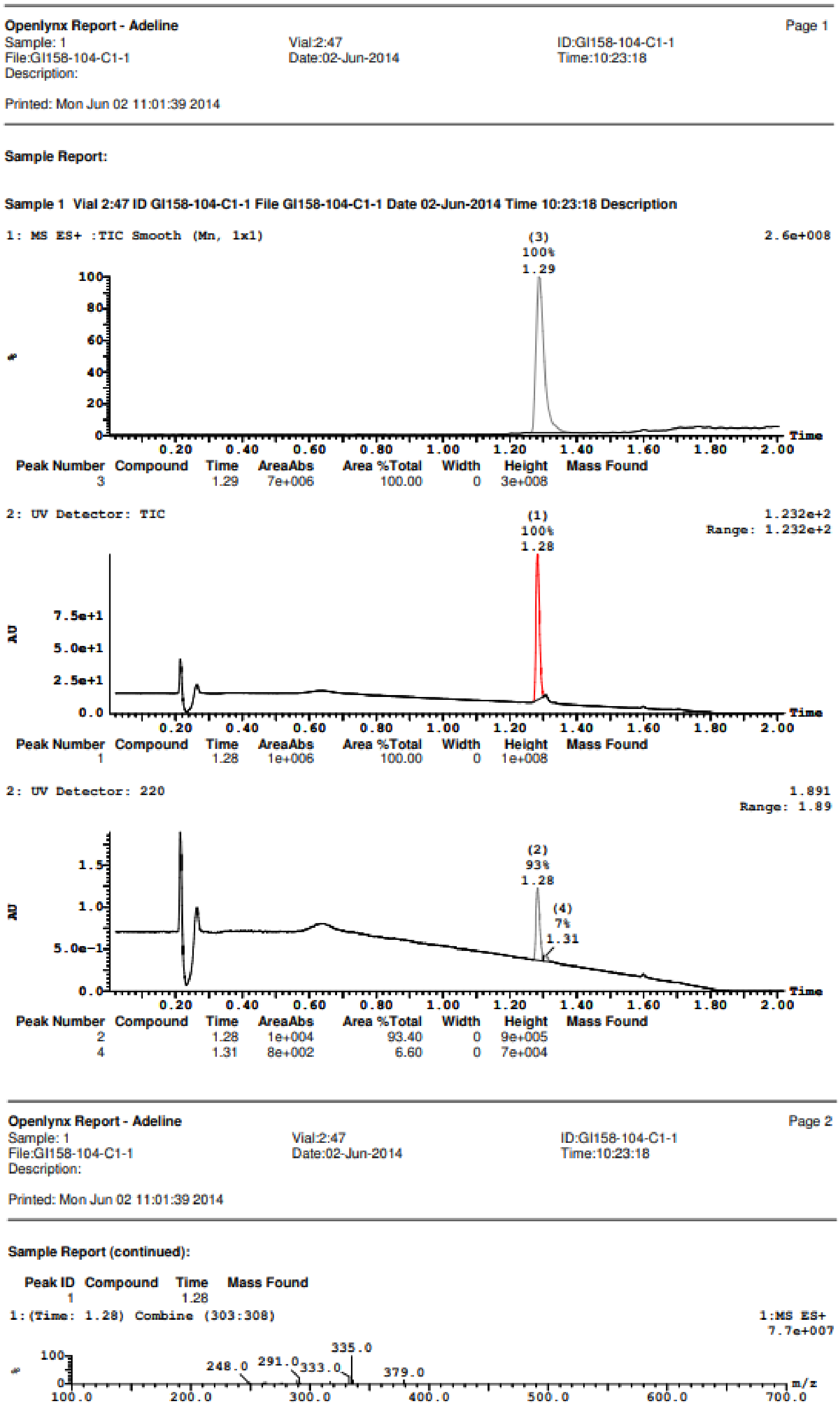

Openlynx Report - Adeline Page 1
Sample: 1 Vial:2:47 ID:GI158-104-C1-1
File:GI158-104-C1-1 Date:02-Jun-2014 Time:10:23:18
Description:

Printed: Mon Jun 02 11:01:39 2014

Sample Report:

Sample 1 Vial 2:47 ID GI158-104-C1-1 File GI158-104-C1-1 Date 02-Jun-2014 Time 10:23:18 Description

Peak Number	Compound	Time	AreaAbs	Area %Total	Width	Height	Mass Found
3		1.29	7e+006	100.00	0	3e+008	

Peak Number	Compound	Time	AreaAbs	Area %Total	Width	Height	Mass Found
1		1.28	1e+006	100.00	0	1e+008	

Peak Number	Compound	Time	AreaAbs	Area %Total	Width	Height	Mass Found
2		1.28	1e+004	93.40	0	9e+005	
4		1.31	8e+002	6.60	0	7e+004	

Openlynx Report - Adeline Page 2
Sample: 1 Vial:2:47 ID:GI158-104-C1-1
File:GI158-104-C1-1 Date:02-Jun-2014 Time:10:23:18
Description:

Printed: Mon Jun 02 11:01:39 2014

Sample Report (continued):

Peak ID	Compound	Time	Mass Found
1		1.28	

1-cyclohexyl-3-methyl-4-(4-morpholinophenyl)-1H-pyrazolo[3,4-b]pyridine-6-carboxylic acid (12)

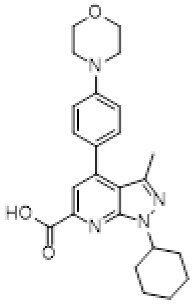

Openlynx Report - Adeline Page 1
Sample: 1 Vial:2:45 ID:GI158-091-CPR3-1
File:GI158-091-CPR3-1 Date:15-May-2014 Time:10:57:38
Description:

Printed: Fri May 16 15:23:09 2014

Sample Report:

Sample 1 Vial 2:45 ID GI158-091-CPR3-1 File GI158-091-CPR3-1 Date 15-May-2014 Time 10:57:38 Description

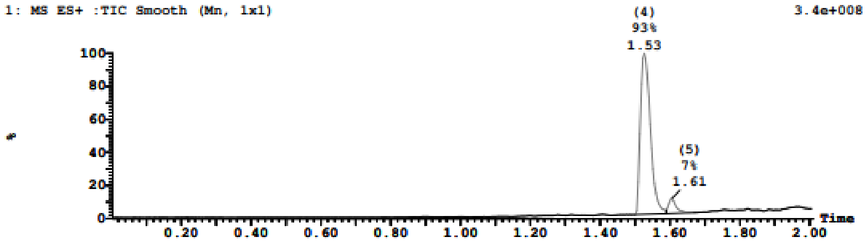

Peak Number	Compound	Time	AreaAbs	Area %Total	Width	Height	Mass Found
4		1.53	1e+007	93.46	0	3e+008	
5		1.61	8e+005	6.54	0	3e+007	

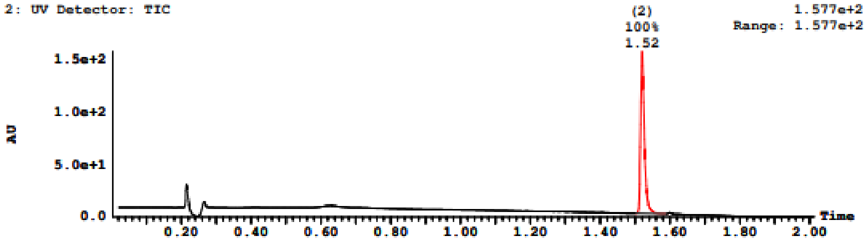

Peak Number	Compound	Time	AreaAbs	Area %Total	Width	Height	Mass Found
2		1.52	2e+006	100.00	0	2e+008	

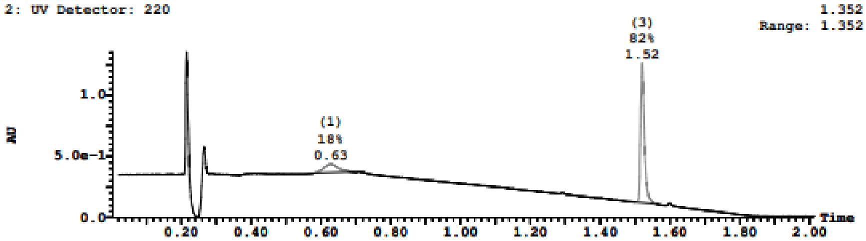

Peak Number	Compound	Time	AreaAbs	Area %Total	Width	Height	Mass Found

Openlynx Report - Adeline Page 2
Sample: 1 Vial:2:45 ID:GI158-091-CPR3-1
File:GI158-091-CPR3-1 Date:15-May-2014 Time:10:57:38
Description:

Printed: Fri May 16 15:23:09 2014

Sample Report (continued):

Peak ID	Compound	Time	Mass Found
2		1.52	

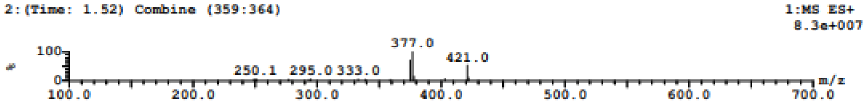

3-methyl-4-(4-morpholinophenyl)-1-(tetrahydro-2H-pyran-4-yl)-1H-pyrazolo[3,4-b]pyridine-6-carboxylic acid (13)

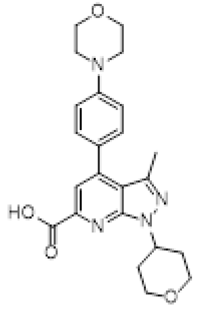

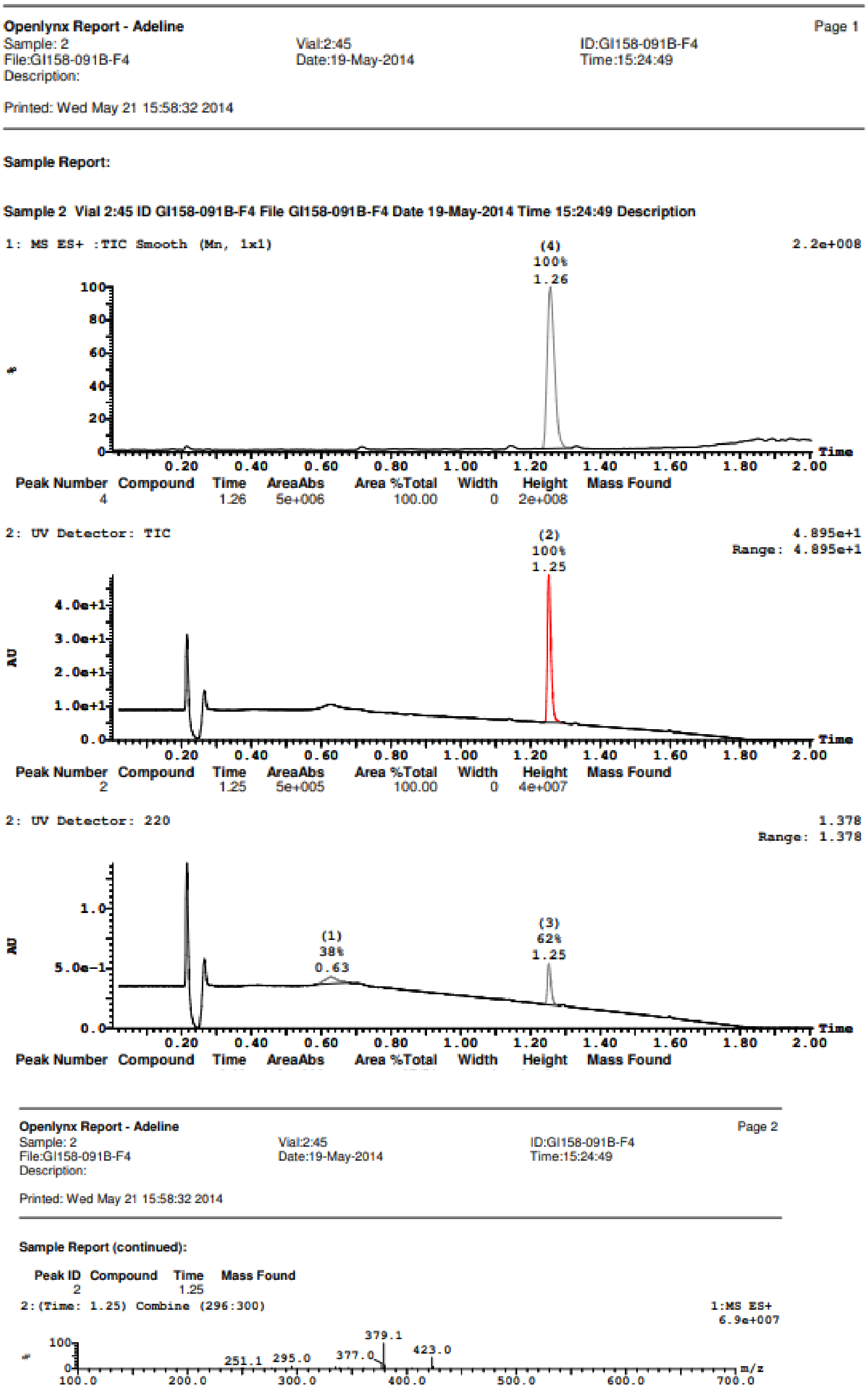

3-methyl-1-(1-methylpiperidin-4-yl)-4-(4-morpholinophenyl)-1H-pyrazolo[3,4-b]pyridine-6-carboxylic acid (14).

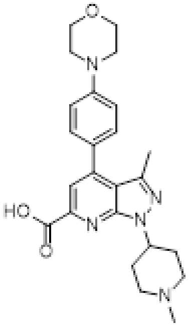

Openlynx Report - Adeline Page 1
Sample: 1 Vial:2:48 ID:GI158-104-D1-1
File:GI158-104-D1-1 Date:02-Jun-2014 Time:10:27:09
Description:

Printed: Mon Jun 02 11:02:39 2014

Sample Report:

Sample 1 Vial 2:48 ID GI158-104-D1-1 File GI158-104-D1-1 Date 02-Jun-2014 Time 10:27:09 Description

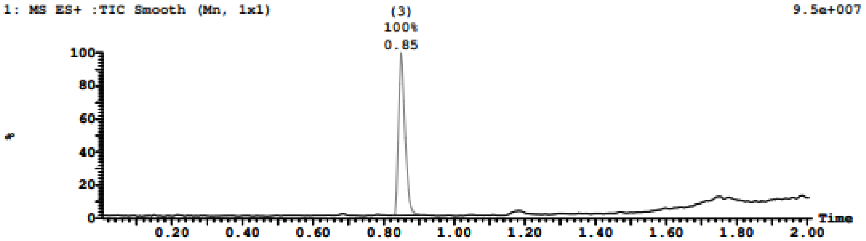

Peak Number	Compound	Time	AreaAbs	Area %Total	Width	Height	Mass Found
3		0.85	2e+006	100.00	0	9e+007	

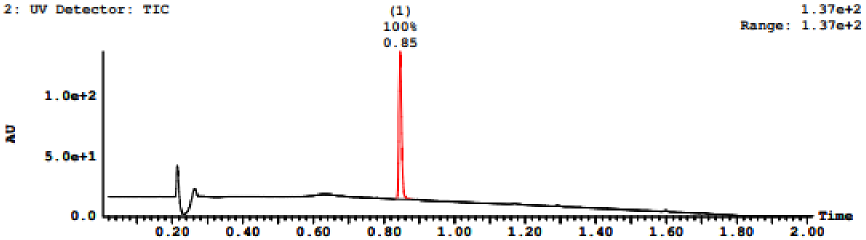

Peak Number	Compound	Time	AreaAbs	Area %Total	Width	Height	Mass Found
1		0.85	1e+006	100.00	0	1e+008	

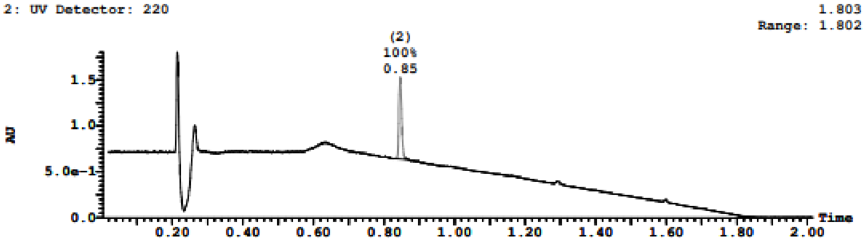

Peak Number	Compound	Time	AreaAbs	Area %Total	Width	Height	Mass Found
2		0.85	8e+003	100.00	0	9e+005	

Openlynx Report - Adeline Page 2
Sample: 1 Vial:2:48 ID:GI158-104-D1-1
File:GI158-104-D1-1 Date:02-Jun-2014 Time:10:27:09
Description:

Printed: Mon Jun 02 11:02:39 2014

Sample Report (continued):

Peak ID	Compound	Time	Mass Found
1		0.85	

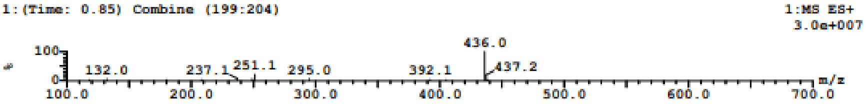

4-(4-morpholinophenyl)-1-phenyl-1H-pyrazolo[3,4-b]pyridine-6-carboxylic acid (15).

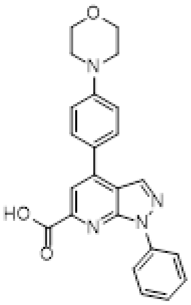

Openlynx Report ·
Sample: 1
File:GI158-072-A1-1
Description:

Vial:2:7
Date:28-Apr-2014

ID:GI158-072-A1-1
Time:09:36:47

Page 1

Printed: Mon Apr 28 12:01:33 2014

Sample Report:

Sample 1 Vial 2:7 ID GI158-072-A1-1 File GI158-072-A1-1 Date 28-Apr-2014 Time 09:36:47 Description

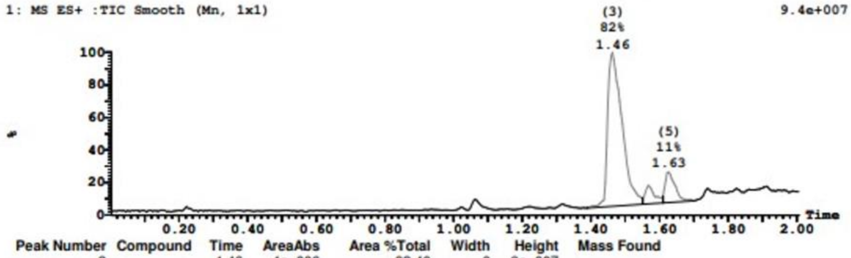

Peak Number	Compound	Time	AreaAbs	Area %Total	Width	Height	Mass Found
3		1.46	4e+006	82.40	0	9e+007	
4		1.57	3e+005	6.16	0	1e+007	
5		1.63	6e+005	11.44	0	2e+007	

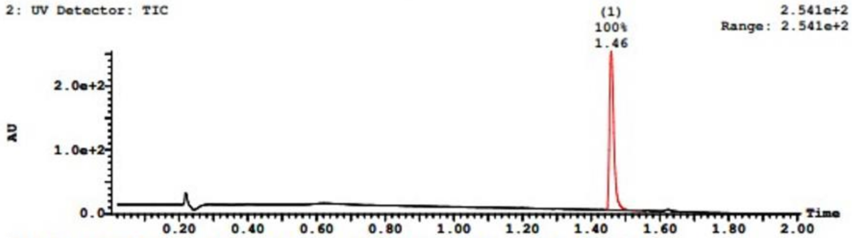

Peak Number	Compound	Time	AreaAbs	Area %Total	Width	Height	Mass Found
1		1.46	4e+006	100.00	0	2e+008	

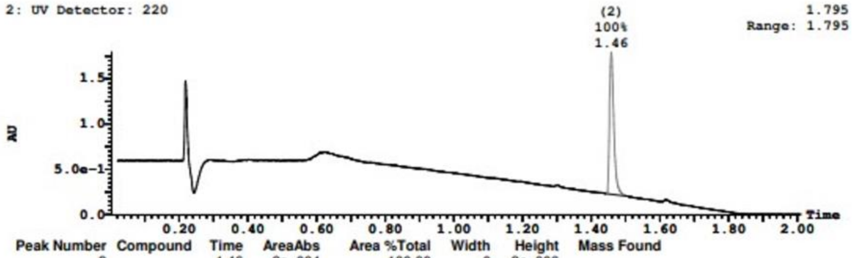

Peak Number	Compound	Time	AreaAbs	Area %Total	Width	Height	Mass Found
2		1.46	2e+004	100.00	0	2e+006	

Openlynx Report - Adeline
Sample: 1
File:GI158-072-A1-1
Description:

Vial:2:7
Date:28-Apr-2014

ID:GI158-072-A1-1
Time:09:36:47

Page 2

Printed: Mon Apr 28 12:01:33 2014

Sample Report (continued):

Peak ID	Compound	Time	Mass Found
1		1.46	

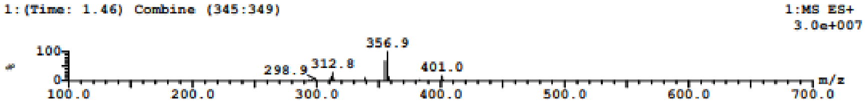

3-isopropyl-4-(4-morpholinophenyl)-1-phenyl-1H-pyrazolo[3,4-b]pyridine-6-carboxylic acid (16).

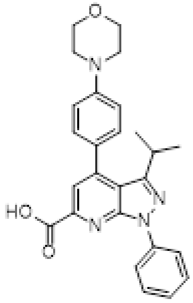

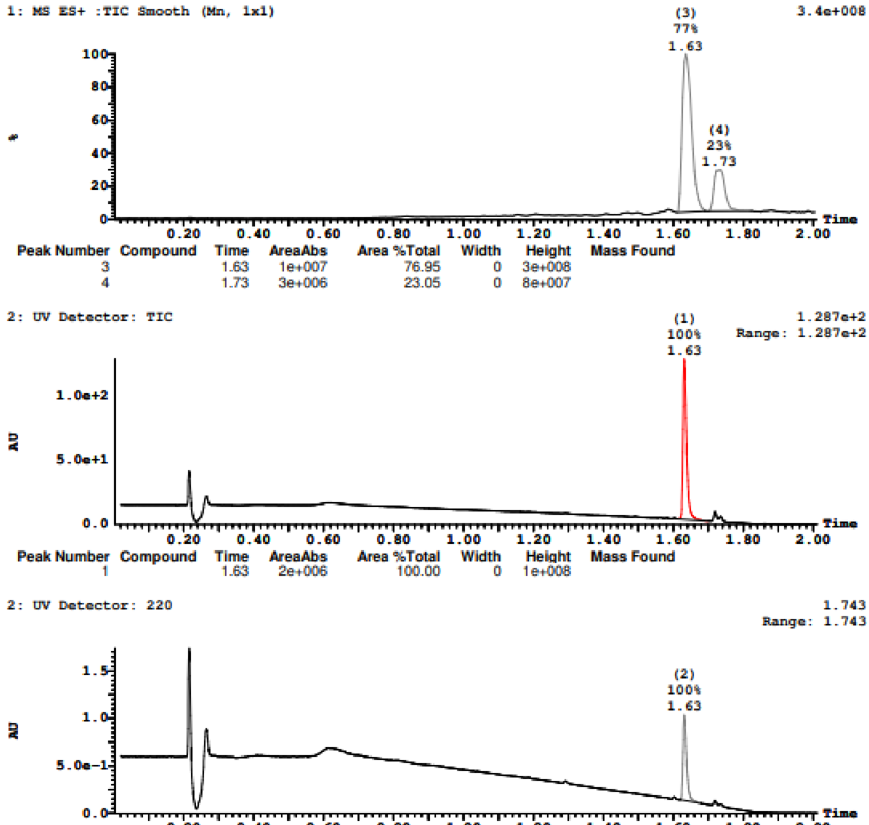

Openlynx Report - Adeline Page 1

Sample: 1 Vial:2:20 ID:GI158-088-PR-1

File:GI158-088-PR-1 Date:30-Apr-2014 Time:15:02:49

Description:

Printed: Wed Apr 30 15:44:16 2014

Sample Report:

Sample 1 Vial 2:20 ID GI158-088-PR-1 File GI158-088-PR-1 Date 30-Apr-2014 Time 15:02:49 Description

1: MS ES+ :TIC Smooth (Mn, 1x1) 3.4e+008

Peak Number	Compound	Time	AreaAbs	Area %Total	Width	Height	Mass Found
3		1.63	1e+007	76.95	0	3e+008	
4		1.73	3e+006	23.05	0	8e+007	

2: UV Detector: TIC 1.287e+2 Range: 1.287e+2

Peak Number	Compound	Time	AreaAbs	Area %Total	Width	Height	Mass Found
1		1.63	2e+006	100.00	0	1e+008	

2: UV Detector: 220 1.743 Range: 1.743

Peak Number	Compound	Time	AreaAbs	Area %Total	Width	Height	Mass Found
2		1.63	1e+004	100.00	0	9e+005	

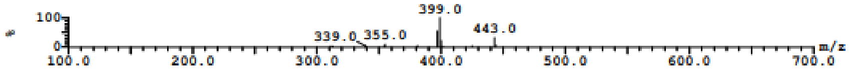

Openlynx Report - Adeline Page 2

Sample: 1 Vial:2:20 ID:GI158-088-PR-1

File:GI158-088-PR-1 Date:30-Apr-2014 Time:15:02:49

Description:

Printed: Wed Apr 30 15:44:16 2014

Sample Report (continued):

Peak ID	Compound	Time	Mass Found
1		1.63	

1: (Time: 1.63) Combine (386:390) 1:MS ES+ 1.1e+008

3-(tert-butyl)-4-(4-morpholinophenyl)-1-phenyl-1H-pyrazolo[3,4-b]pyridine-6-carboxylic acid (17).

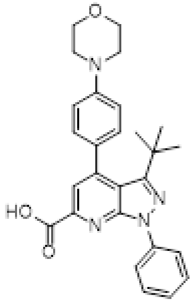

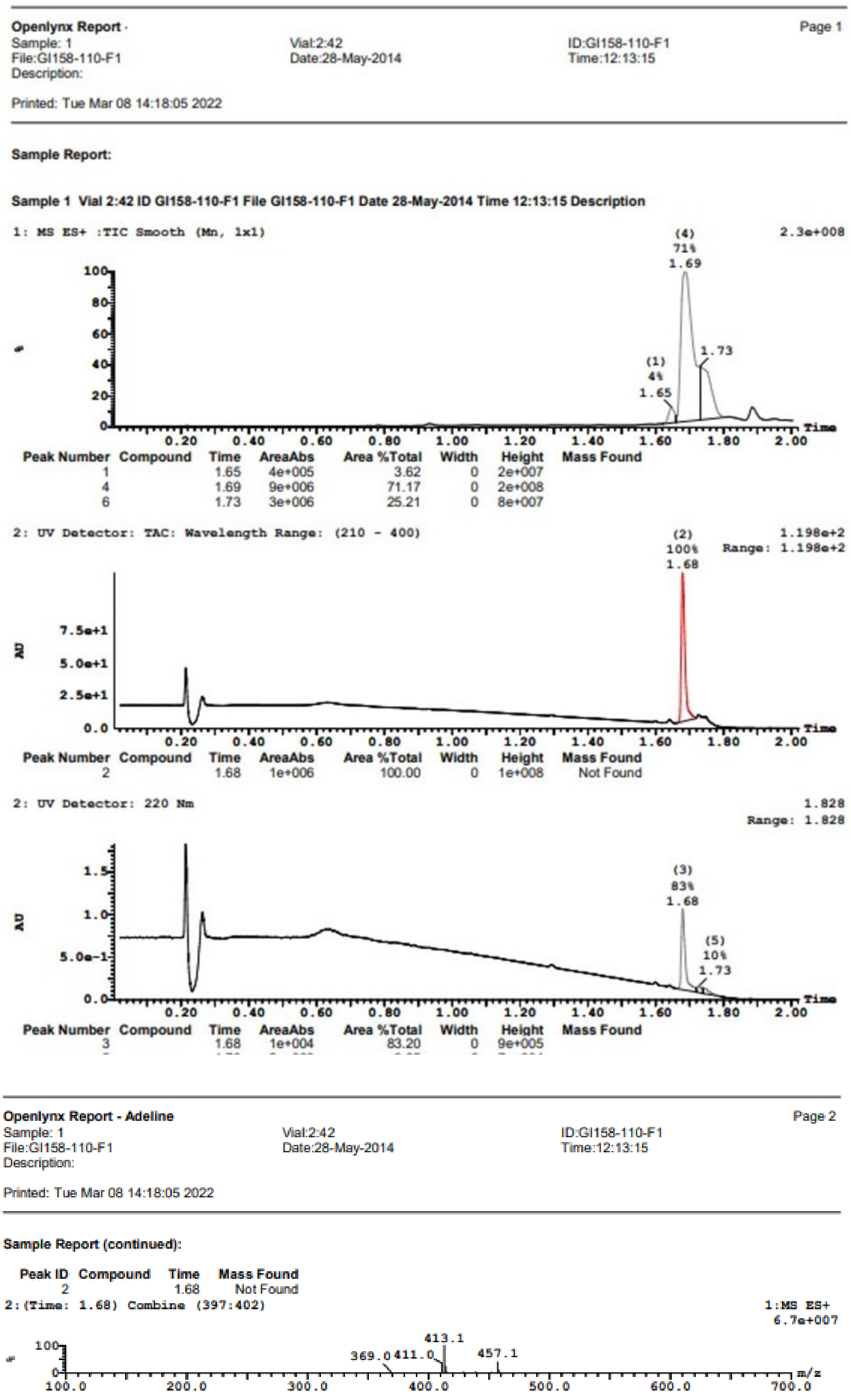

Openlynx Report · Page 1
Sample: 1 · Vial:2:42 · ID:GI158-110-F1
File:GI158-110-F1 · Date:28-May-2014 · Time:12:13:15
Description:

Printed: Tue Mar 08 14:18:05 2022

Sample Report:

Sample 1 Vial 2:42 ID GI158-110-F1 File GI158-110-F1 Date 28-May-2014 Time 12:13:15 Description

1: MS ES+ :TIC Smooth (Mn, 1x1) 2.3e+008

Peak Number	Compound	Time	AreaAbs	Area %Total	Width	Height	Mass Found
1		1.65	4e+005	3.62	0	2e+007	
4		1.69	9e+006	71.17	0	2e+008	
6		1.73	3e+006	25.21	0	8e+007	

2: UV Detector: TAC: Wavelength Range: (210 - 400) 1.198e+2 Range: 1.198e+2

Peak Number	Compound	Time	AreaAbs	Area %Total	Width	Height	Mass Found
2		1.68	1e+006	100.00	0	1e+008	Not Found

2: UV Detector: 220 Nm 1.828 Range: 1.828

Peak Number	Compound	Time	AreaAbs	Area %Total	Width	Height	Mass Found
3		1.68	1e+004	83.20	0	9e+005	

Openlynx Report - Adeline · Page 2
Sample: 1 · Vial:2:42 · ID:GI158-110-F1
File:GI158-110-F1 · Date:28-May-2014 · Time:12:13:15
Description:

Printed: Tue Mar 08 14:18:05 2022

Sample Report (continued):

Peak ID	Compound	Time	Mass Found
2		1.68	Not Found

2:(Time: 1.68) Combine (397:402) 1:MS ES+ 6.7e+007

3-cyclobutyl-4-(4-morpholinophenyl)-1-phenyl-1H-pyrazolo[3,4-b]pyridine-6-carboxylic acid (18).

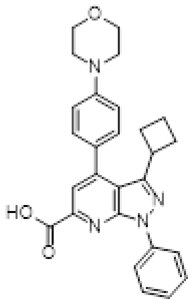

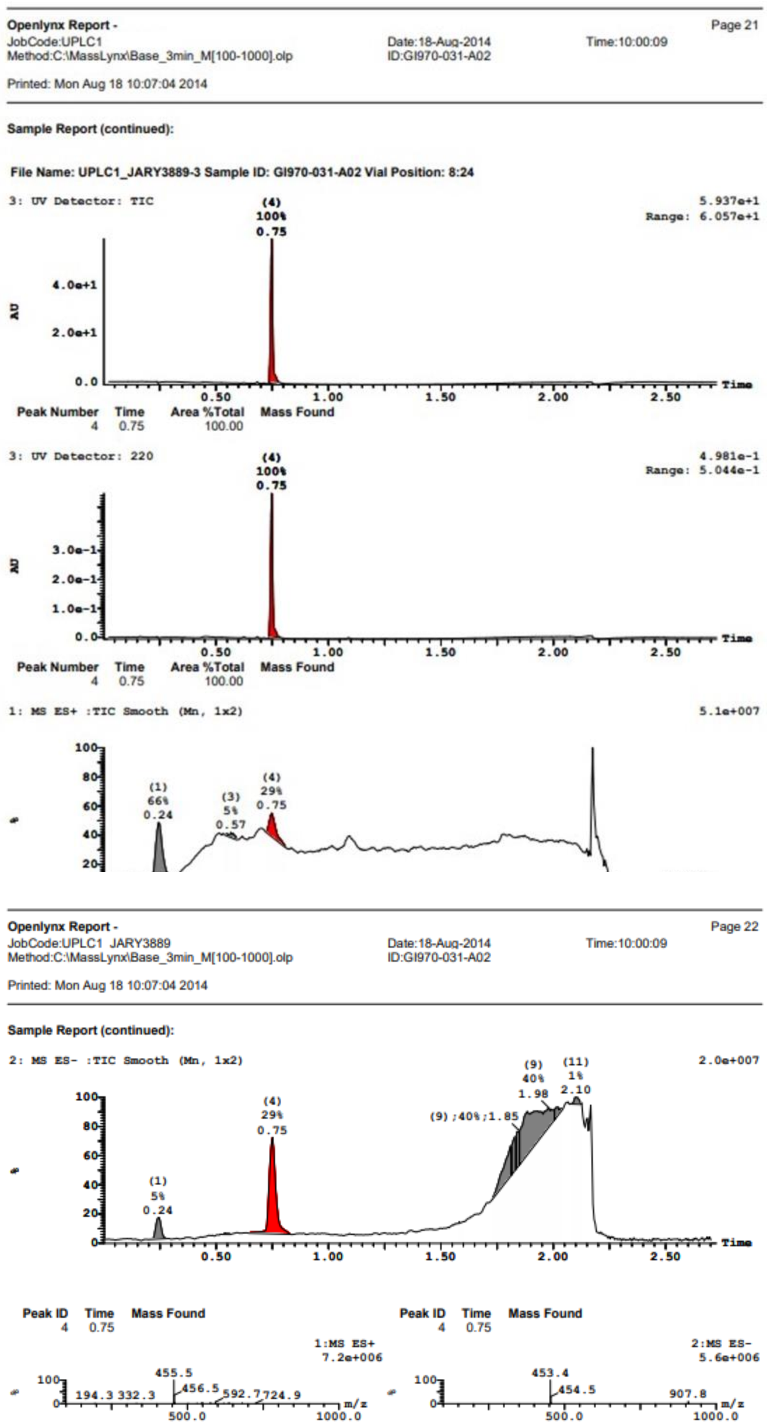

3-cyclobutyl-4-(4-morpholinophenyl)-1-phenyl-1H-pyrazolo[3,4-b]pyridine-6-carboxylic acid (18).

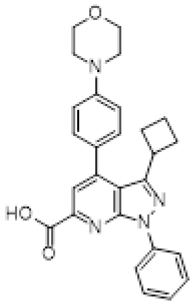

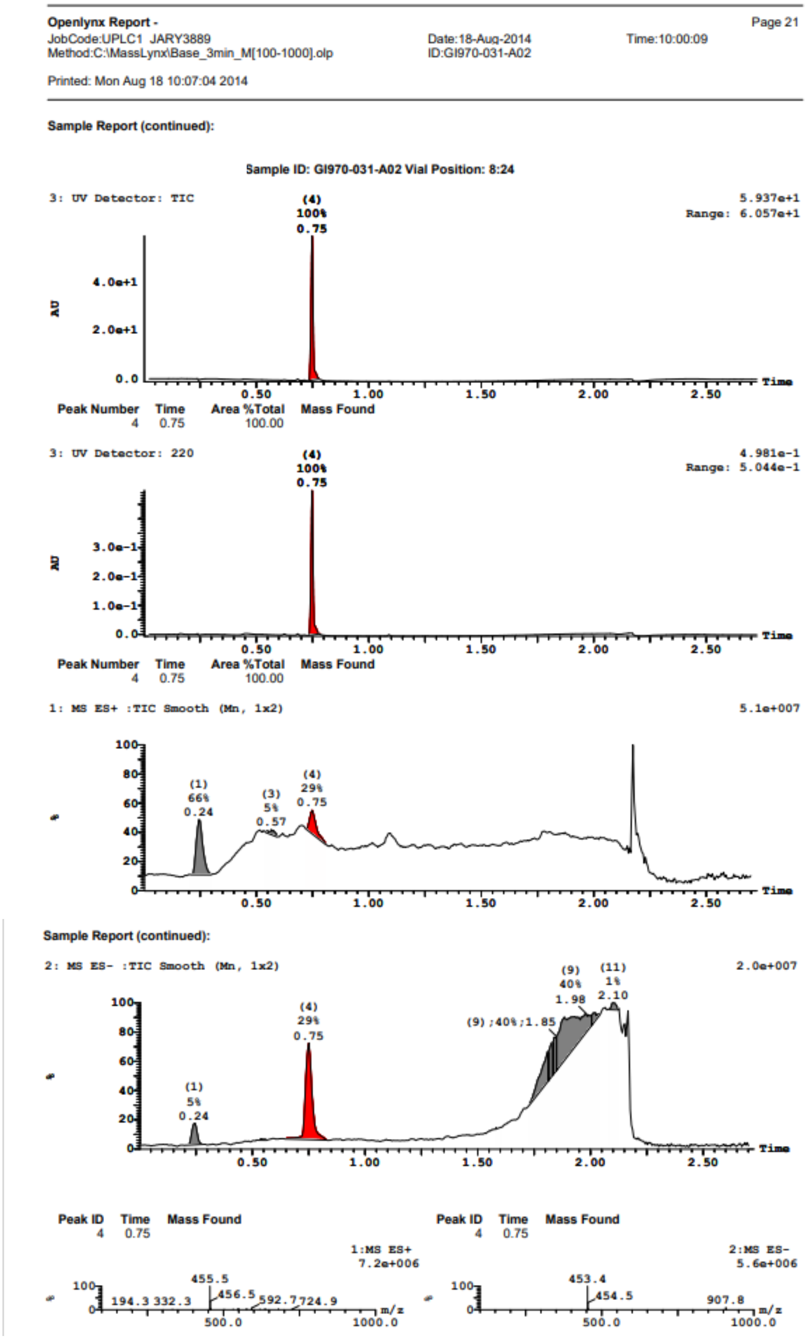

3-(1-methylcyclobutyl)-4-(4-morpholinophenyl)-1-phenyl-1H-pyrazolo[3,4-b]pyridine-6-carboxylic acid (19).

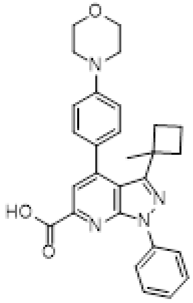

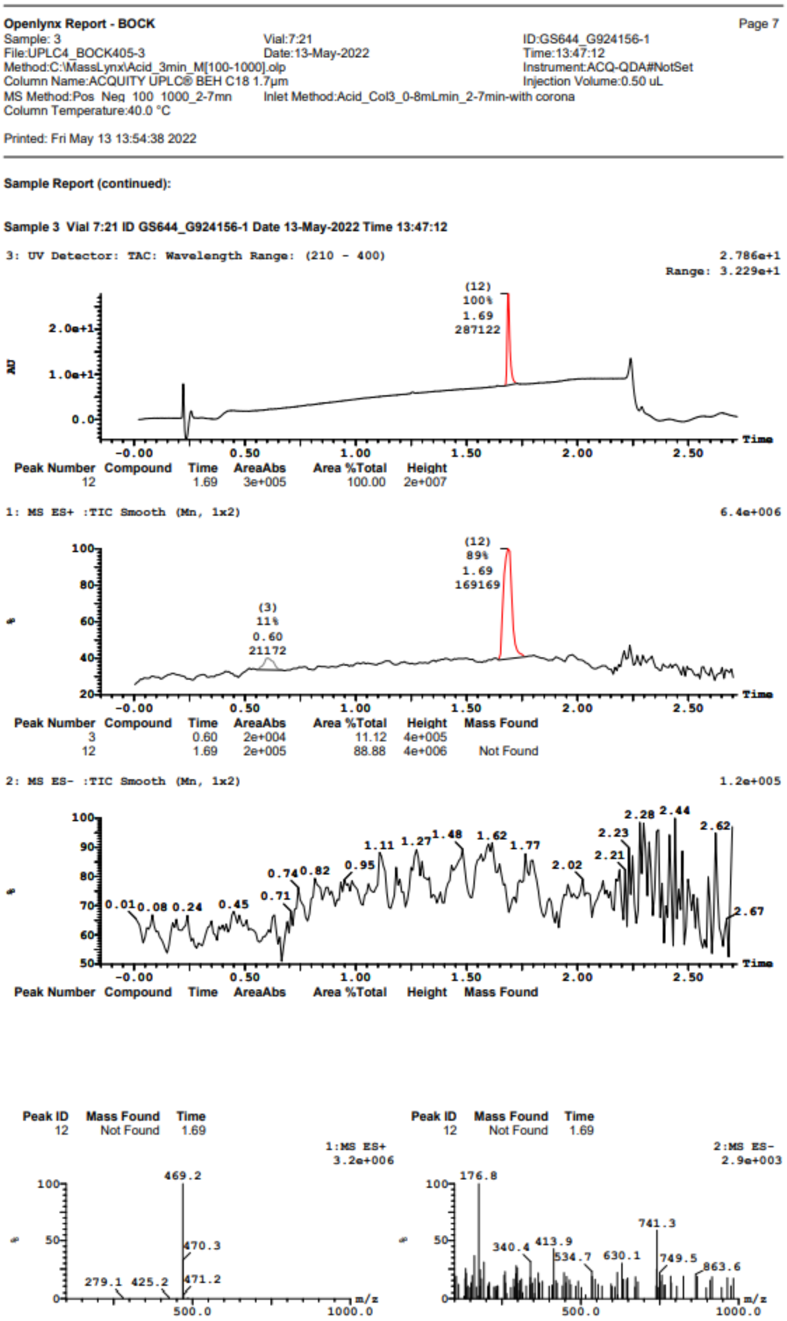

3-(3-methyloxetan-3-yl)-4-(4-morpholinophenyl)-1-phenyl-1H-pyrazolo[3,4-b]pyridine-6-carboxylic acid (20).

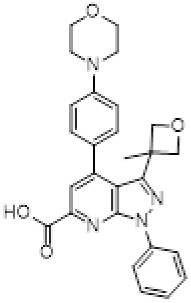

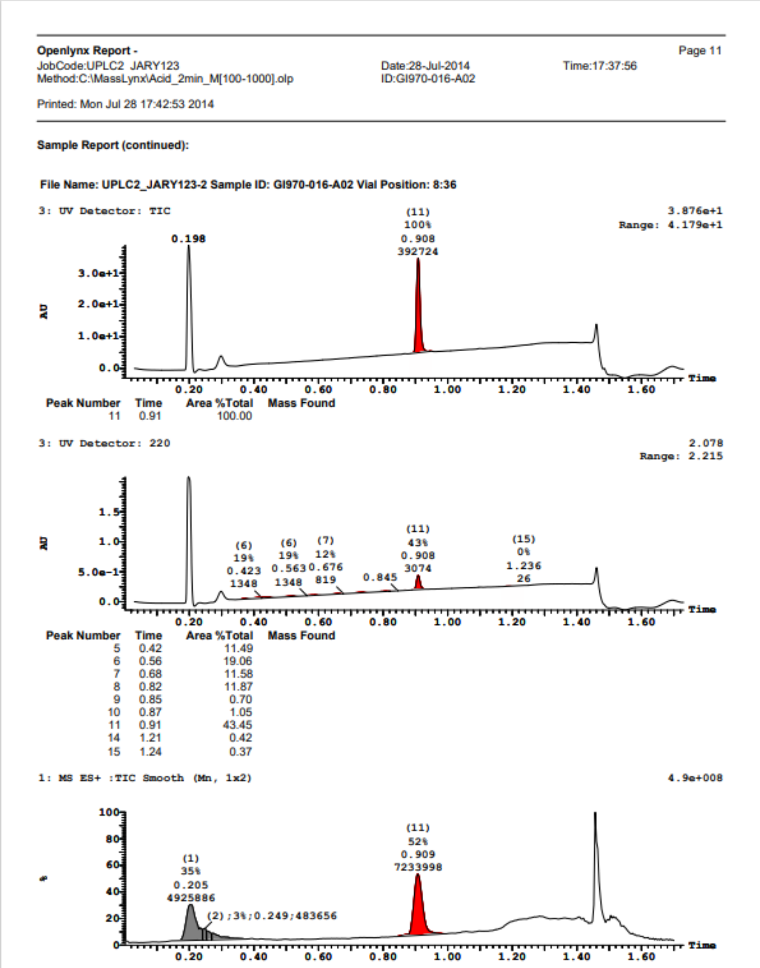

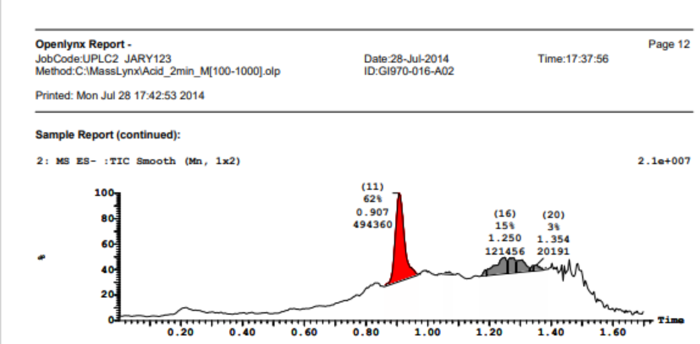

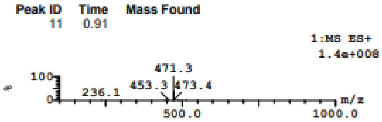

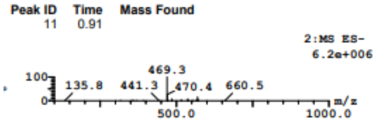

3-(methoxymethyl)-4-(4-morpholinophenyl)-1-phenyl-1H-pyrazolo[3,4-b]pyridine-6-carboxylic acid (21).

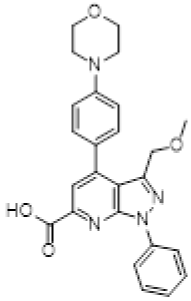

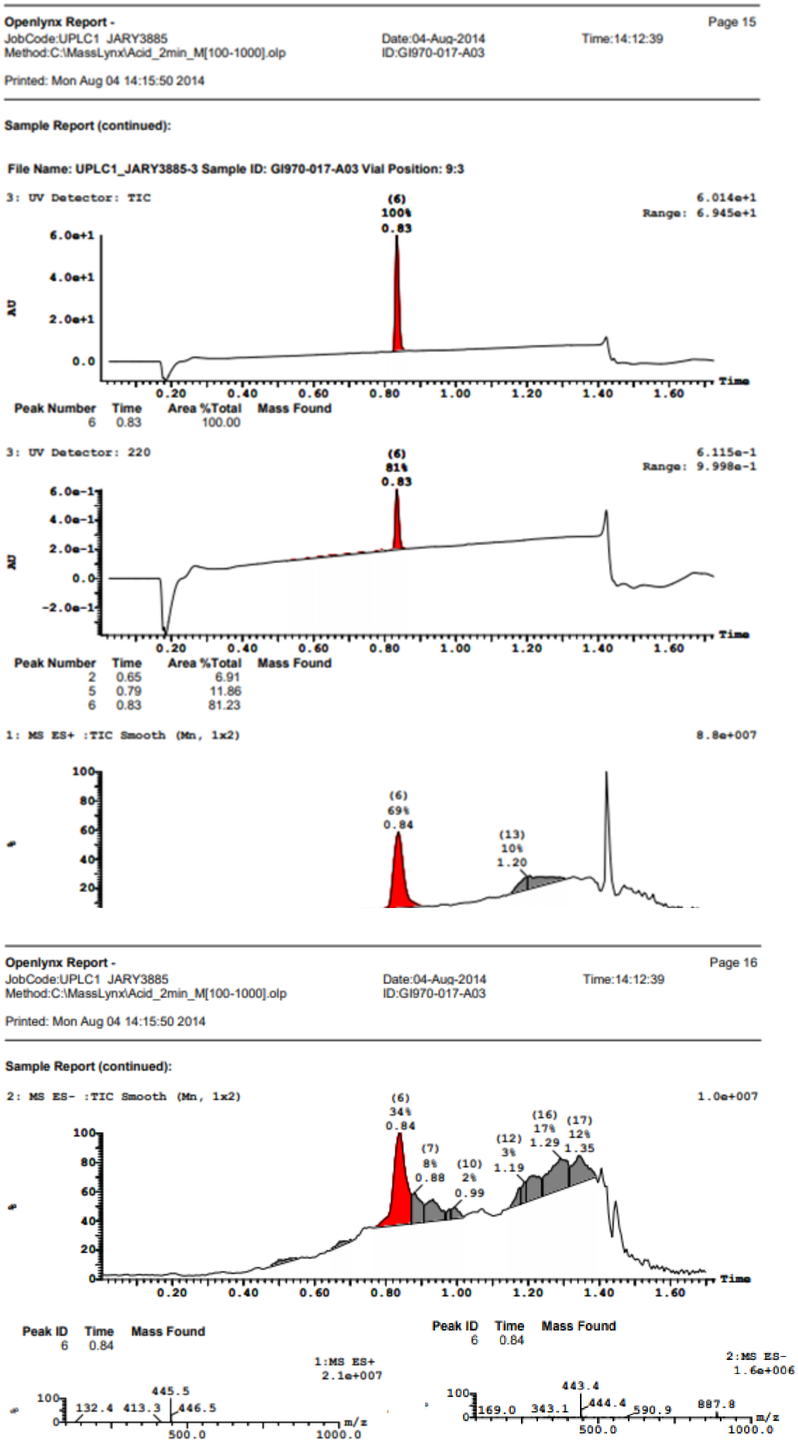

3-(hydroxymethyl)-4-(4-morpholinophenyl)-1-phenyl-1H-pyrazolo[3,4-b]pyridine-6-carboxylic acid (22).

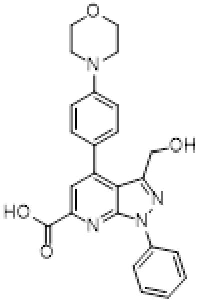

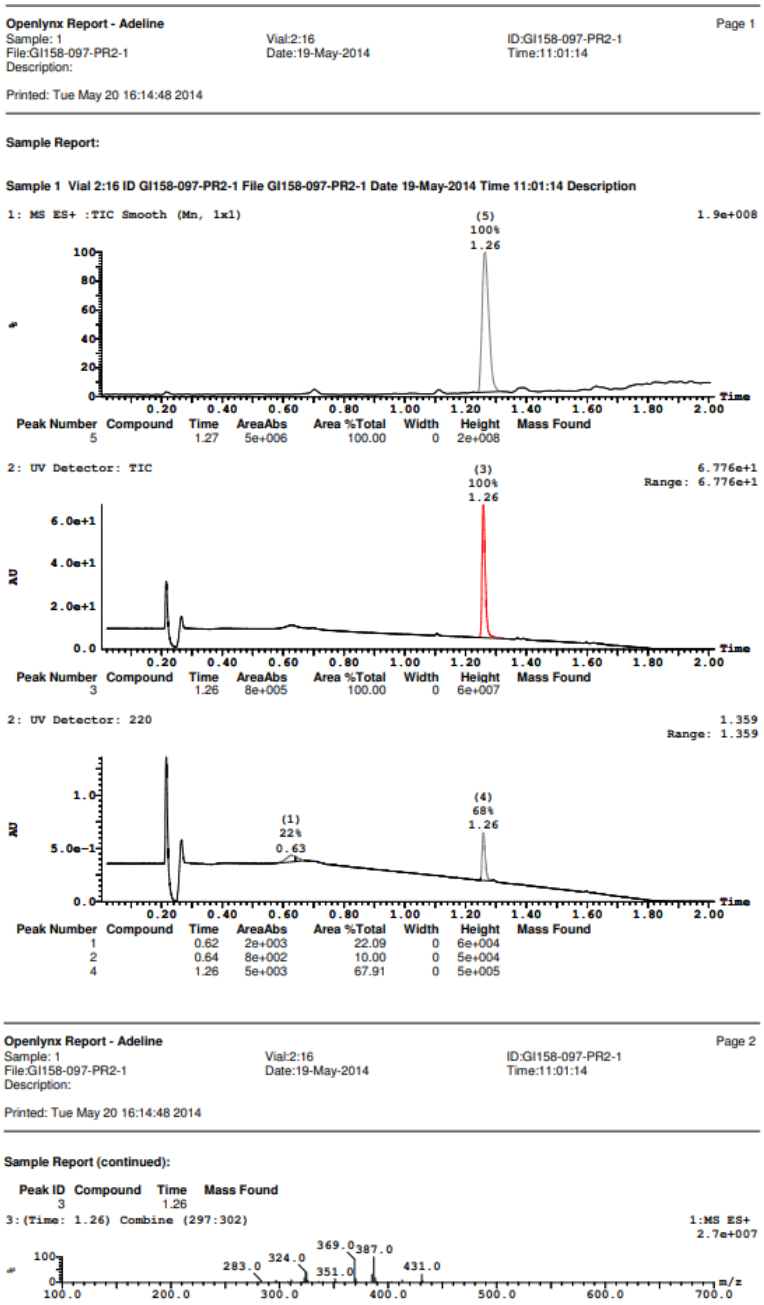

Openlynx Report - Adeline Page 1
Sample: 1 Vial:2:16 ID:GI158-097-PR2-1
File:GI158-097-PR2-1 Date:19-May-2014 Time:11:01:14
Description:

Printed: Tue May 20 16:14:48 2014

Sample Report:

Sample 1 Vial 2:16 ID GI158-097-PR2-1 File GI158-097-PR2-1 Date 19-May-2014 Time 11:01:14 Description

1: MS ES+ :TIC Smooth (Mn, 1x1) 1.9e+008

Peak Number	Compound	Time	AreaAbs	Area %Total	Width	Height	Mass Found
5		1.27	5e+006	100.00	0	2e+008	

2: UV Detector: TIC 6.776e+1 Range: 6.776e+1

Peak Number	Compound	Time	AreaAbs	Area %Total	Width	Height	Mass Found
3		1.26	8e+005	100.00	0	6e+007	

2: UV Detector: 220 1.359 Range: 1.359

Peak Number	Compound	Time	AreaAbs	Area %Total	Width	Height	Mass Found
1		0.62	2e+003	22.09	0	6e+004	
2		0.64	8e+002	10.00	0	5e+004	
4		1.26	5e+003	67.91	0	5e+005	

Openlynx Report - Adeline Page 2
Sample: 1 Vial:2:16 ID:GI158-097-PR2-1
File:GI158-097-PR2-1 Date:19-May-2014 Time:11:01:14
Description:

Printed: Tue May 20 16:14:48 2014

Sample Report (continued):

Peak ID	Compound	Time	Mass Found
3		1.26	

3: (Time: 1.26) Combine (297:302) 1:MS ES+ 2.7e+007

3-methyl-4-(6-morpholinopyridin-3-yl)-1-phenyl-1H-pyrazolo[3,4-b]pyridine-6-carboxylic acid (23).

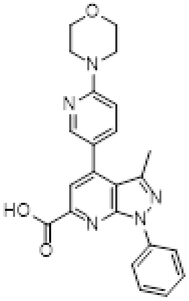

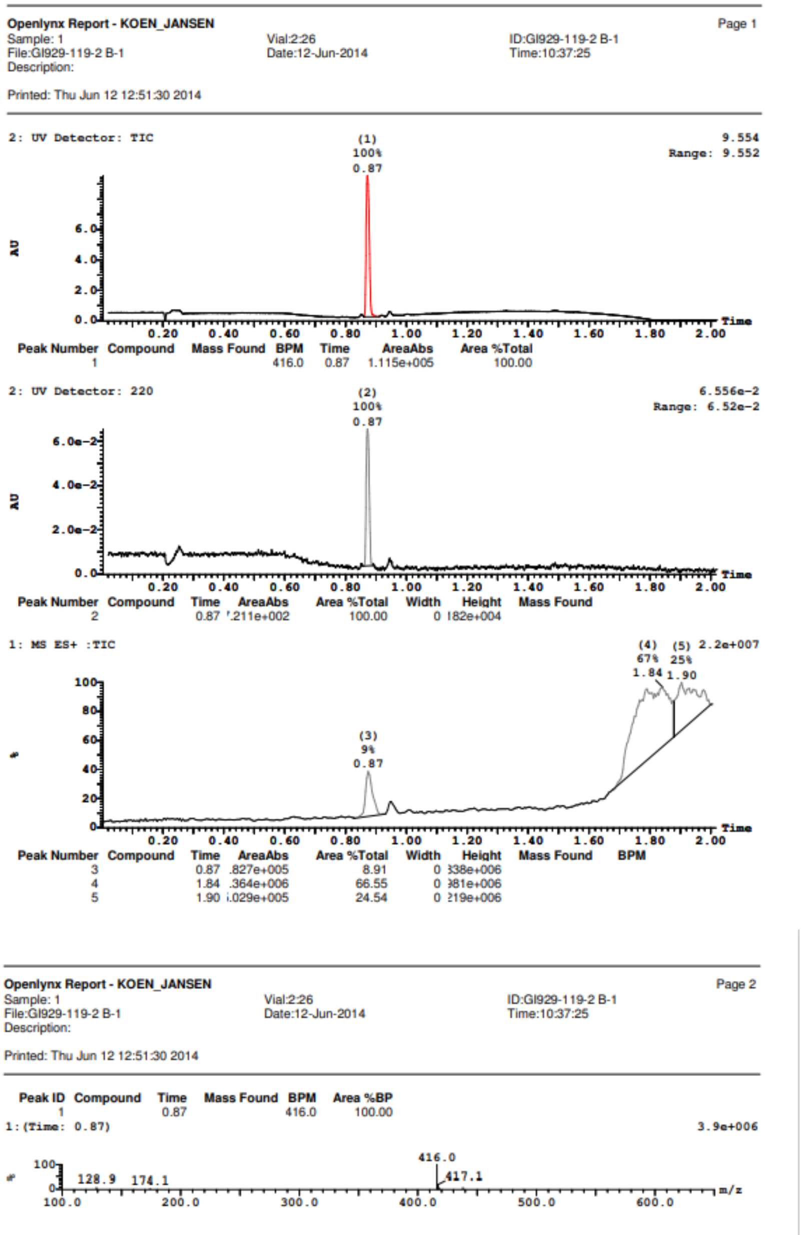

4-(4-((2-methoxyethyl)amino)phenyl)-3-methyl-1-phenyl-1H-pyrazolo[3,4-b]pyridine-6-carboxylic acid (24).

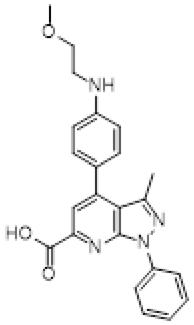

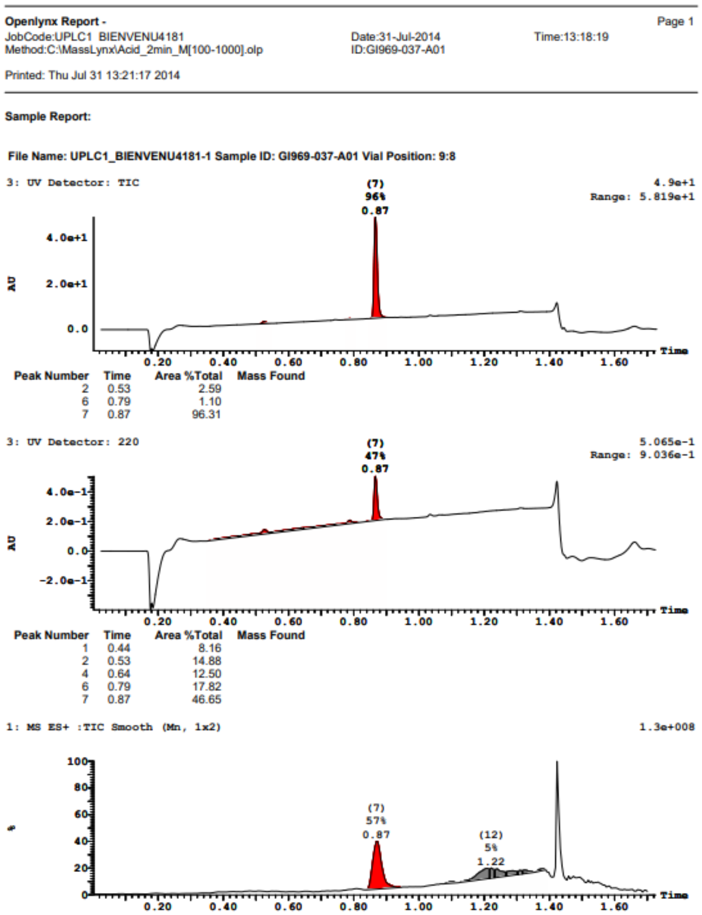

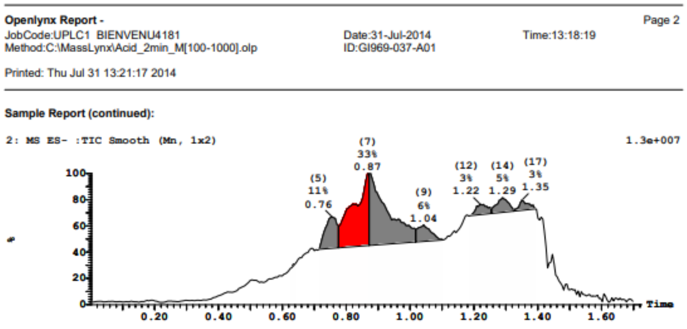

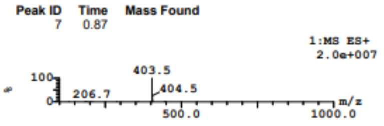

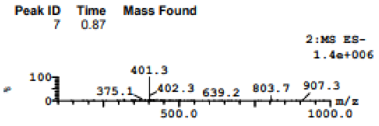

4-(4-((2-methoxyethyl)(methyl)amino)phenyl)-3-methyl-1-phenyl-1H-pyrazolo[3,4-b]pyridine-6-carboxylic acid (25).

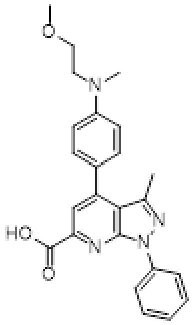

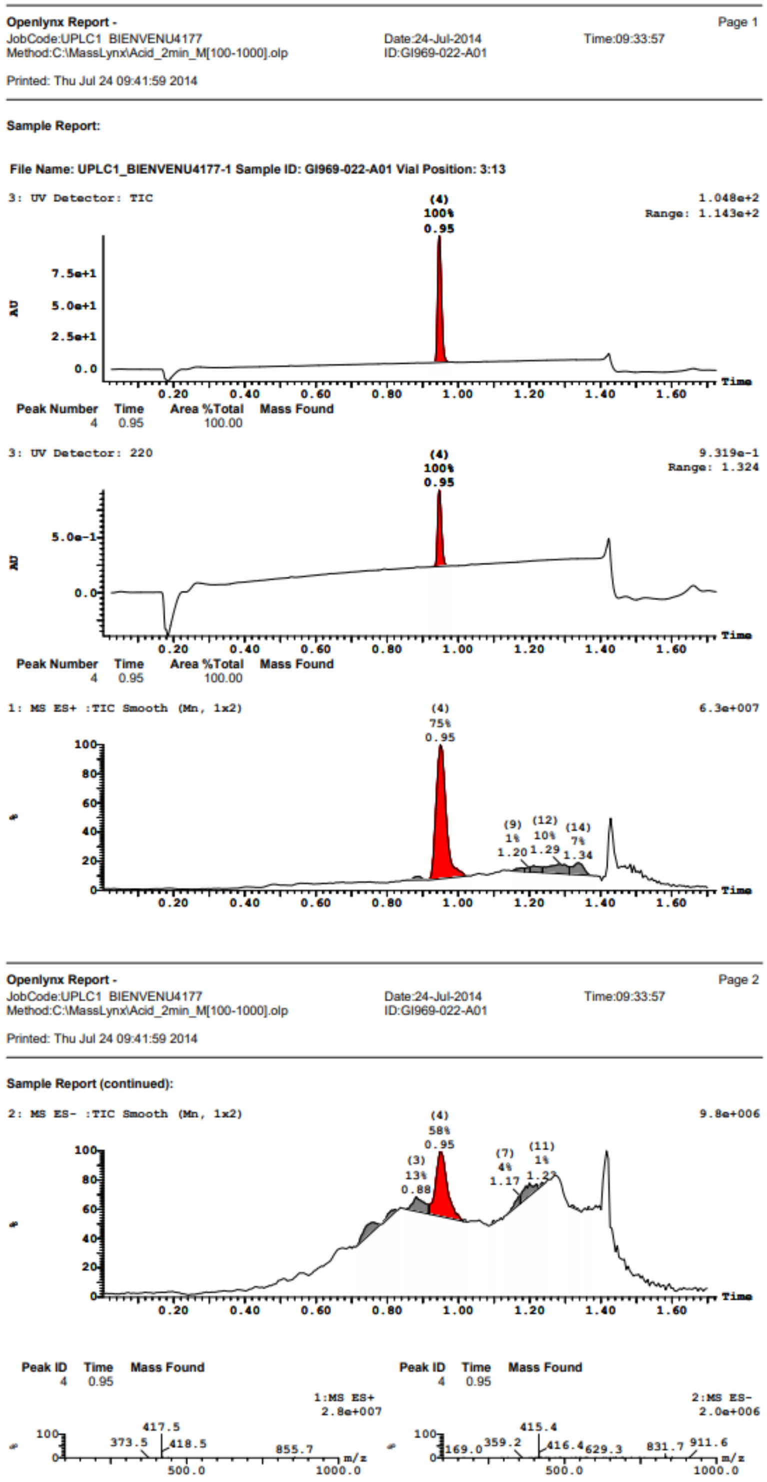

3-methyl-4-(4-(methyl(tetrahydro-2H-pyran-4-yl)amino)phenyl)-1-phenyl-1H-pyrazolo[3,4-b]pyridine-6-carboxylic acid (26).

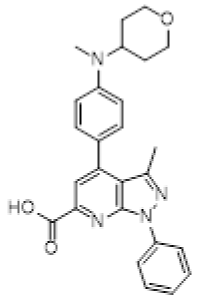

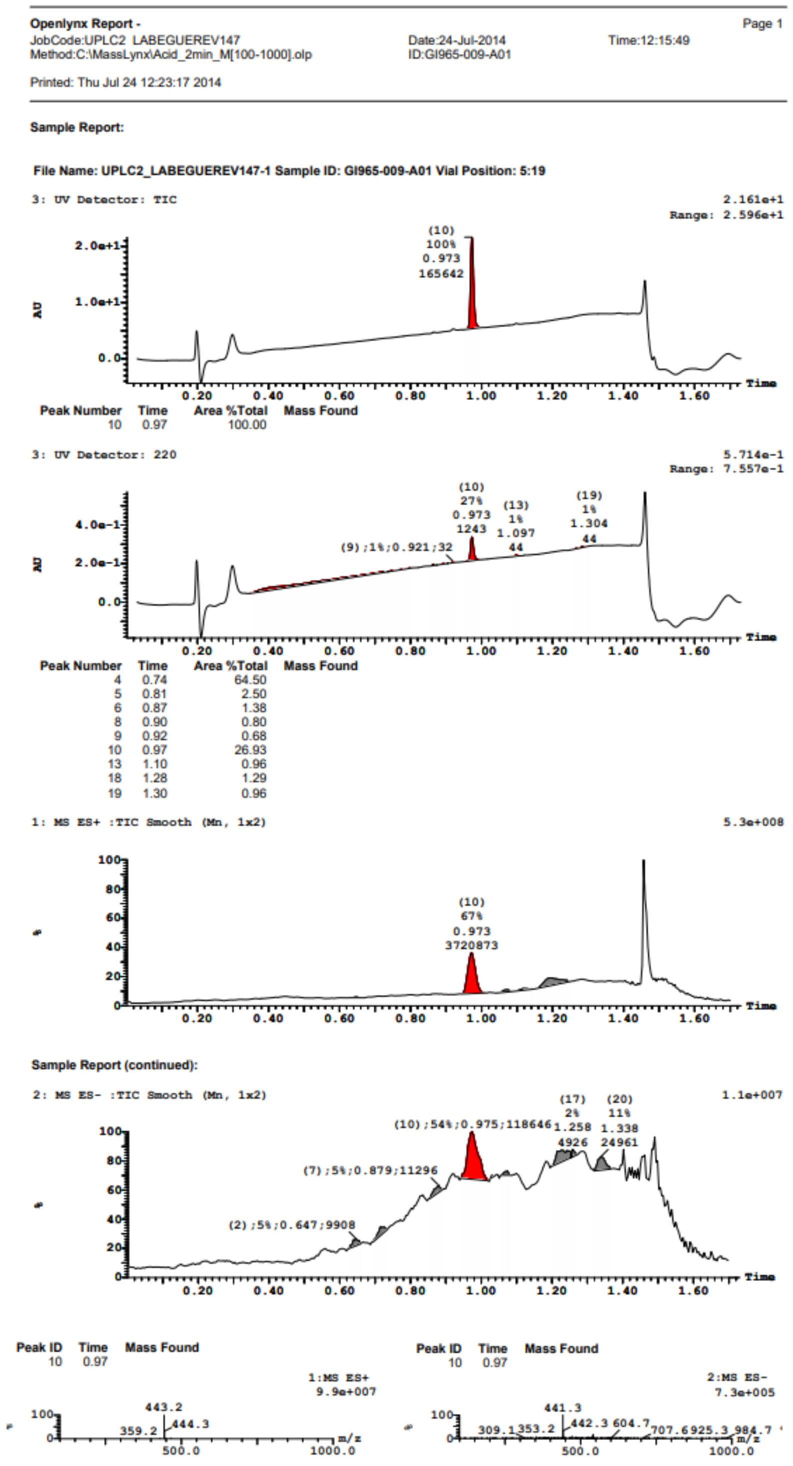

4-(4-(4-hydroxypiperidin-1-yl)phenyl)-3-methyl-1-phenyl-1H-pyrazolo[3,4-b]pyridine-6-carboxylic acid (27)

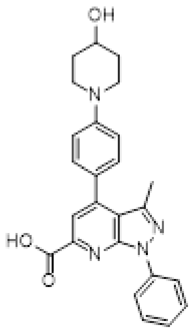

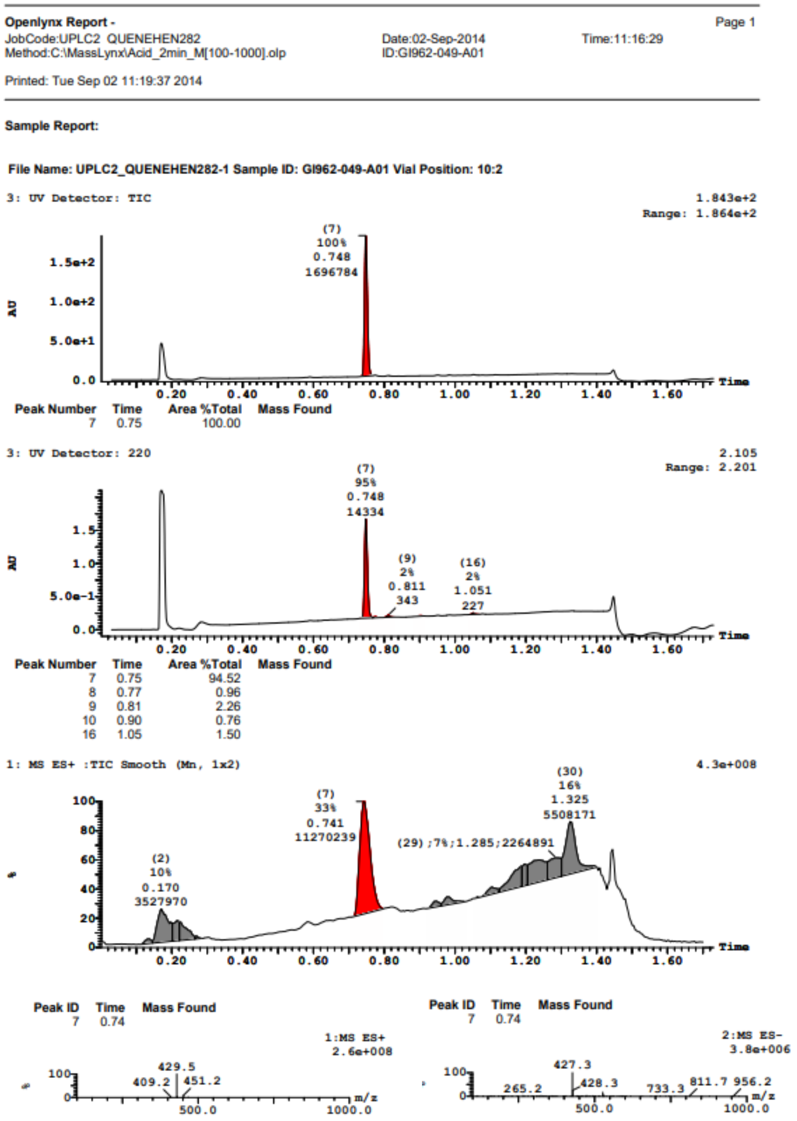

4-(4-(4-cyanopiperidin-1-yl)phenyl)-3-methyl-1-phenyl-1H-pyrazolo[3,4-b]pyridine-6-carboxylic acid (28).

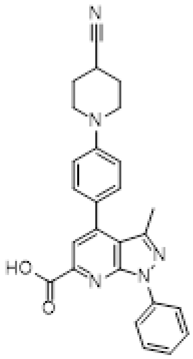

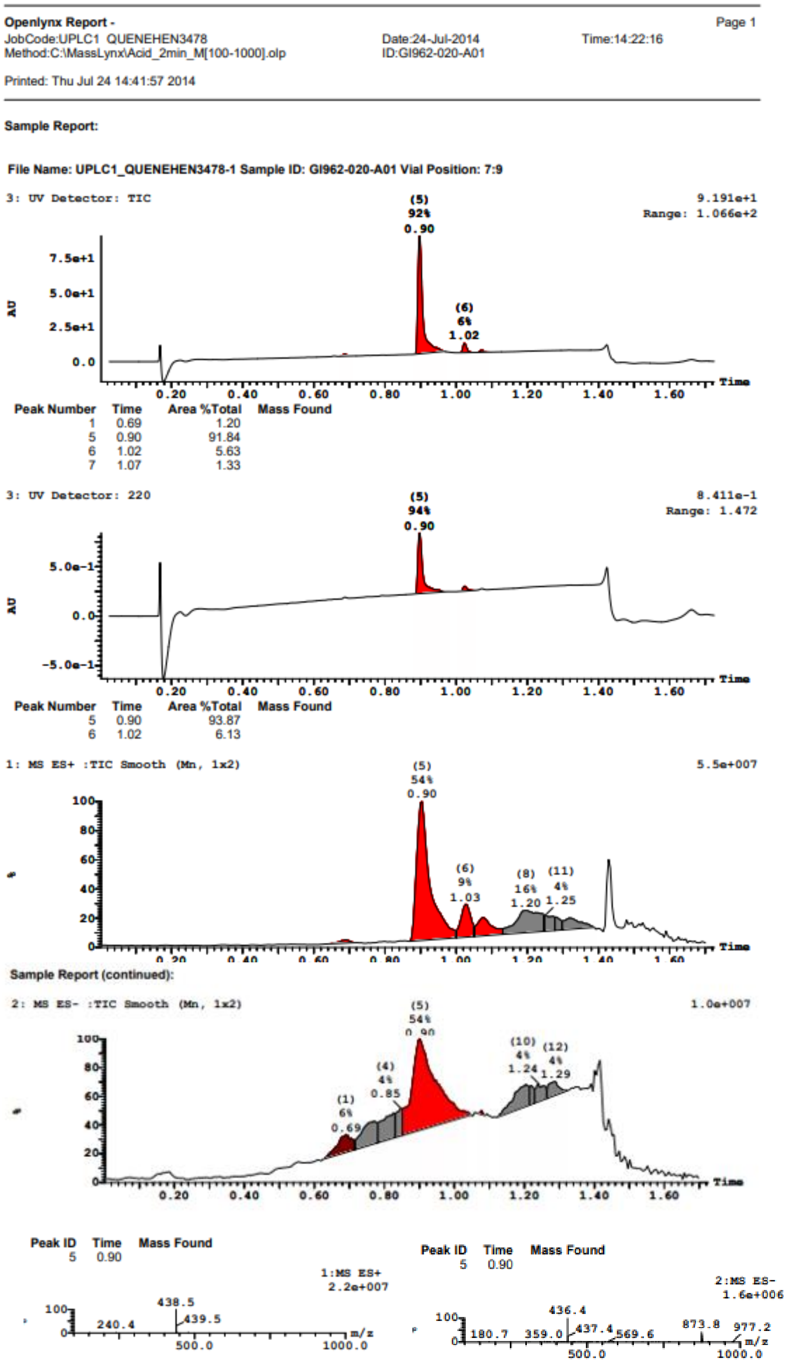

^{1}H NMR (400 MHz, DMSO) δ 8.38 – 8.31 (m, 2H), 7.78 (s, 1H), 7.64 – 7.56 (m, 2H), 7.56 – 7.50 (m, 2H), 7.36 (t, *J* = 7.4 Hz, 1H), 7.15 (d, *J* = 8.7 Hz, 2H), 3.59 – 3.49 (m, 2H), 3.28 – 3.17 (m, 2H), 3.12 (tt, *J* = 8.4, 4.1 Hz, 1H), 2.39 (s, 3H), 2.08 – 1.97 (m, 2H), 1.94 – 1.79 (m, 2H).

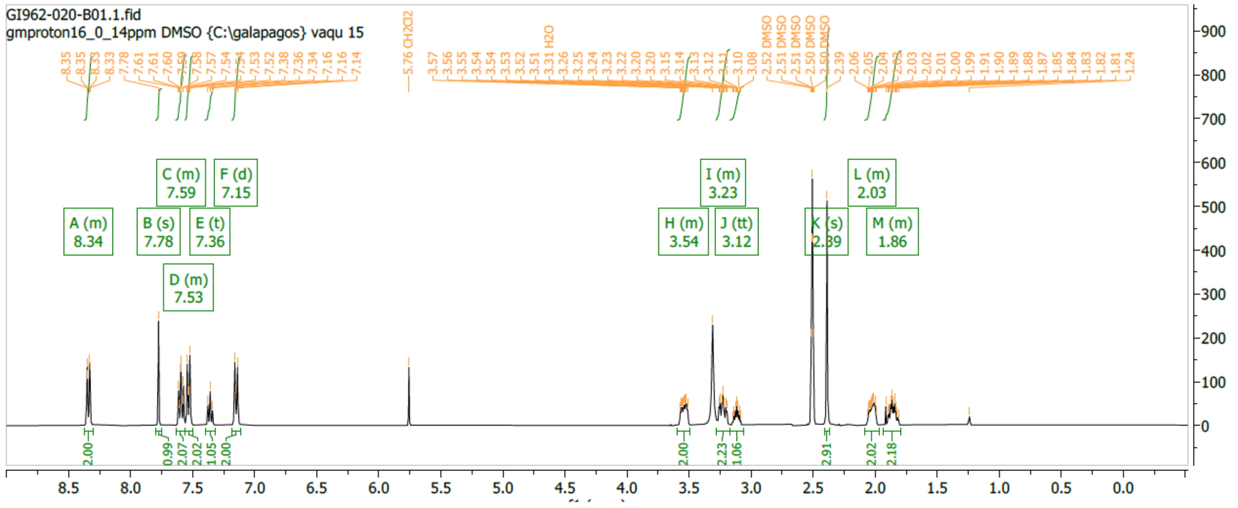

1-(3-methyl-4-(4-morpholinophenyl)-1-phenyl-1H-pyrazolo[3,4-b]pyridin-6-yl)ethan-1-one (29).

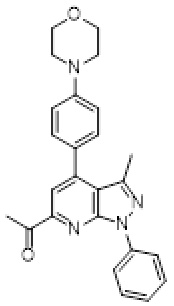

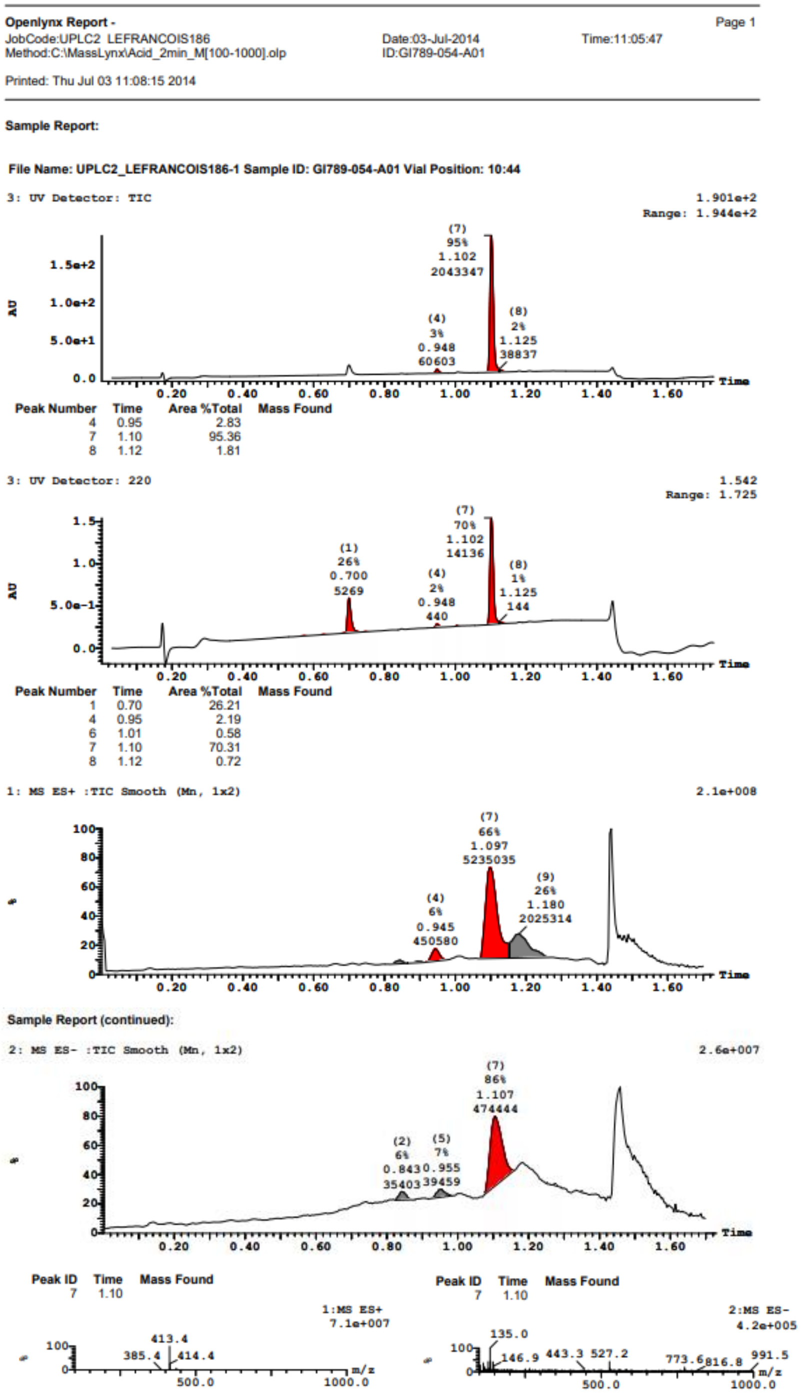

(3-methyl-4-(4-morpholinophenyl)-1-phenyl-1H-pyrazolo[3,4-b]pyridin-6-yl)methanol (30).

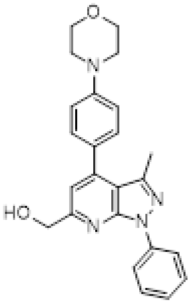

Openlynx Report - KOEN_JANSEN Page 1
Sample: 1 Vial:1:25 ID:GI929-096 MECN WASH-1
File:GI929-096 MECN WASH-1 Date:16-May-2014 Time:16:34:25
Description:

Printed: Tue Mar 08 14:08:15 2022

Sample Report:

Sample 1 Vial 1:25 ID GI929-096 MECN WASH-1 File GI929-096 MECN WASH-1 Date 16-May-2014 Time 16:34:25 Description

1: MS ES+ :TIC Smooth (Mn, 1x1) 5.3e+007

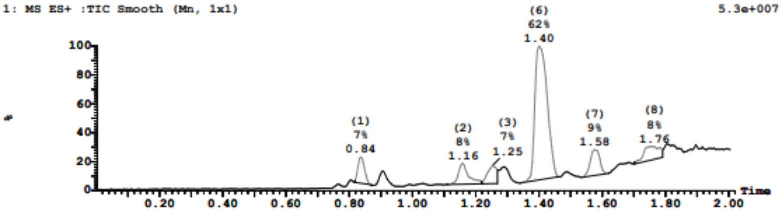

Peak Number	Compound	Time	AreaAbs	Area %Total	Width	Height	Mass Found
1		0.84	2e+005	6.56	0	1e+007	
2		1.16	3e+005	7.96	0	8e+006	
3		1.25	2e+005	6.63	0	7e+006	
6		1.40	2e+006	61.63	0	5e+007	
7		1.58	3e+005	9.20	0	9e+006	
8		1.77	3e+005	8.02	0	5e+006	

2: UV Detector: TAC: Wavelength Range: (210 - 400) 1.093e+2
Range: 1.093e+2

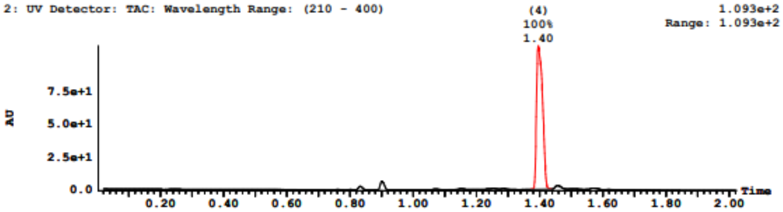

Peak Number	Compound	Time	AreaAbs	Area %Total	Width	Height	Mass Found
4		1.40	2e+006	100.00	0	1e+008	Not Found

2: UV Detector: 220 Nm 7.917e-1
Range: 7.916e-1

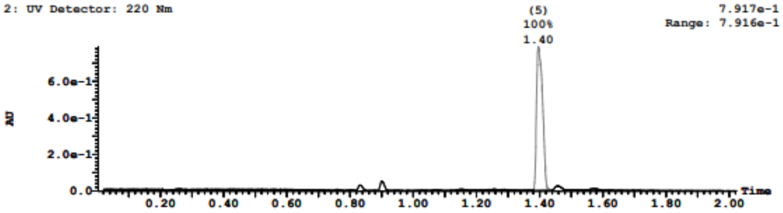

Openlynx Report - KOEN_JANSEN Page 2
Sample: 1 Vial:1:25 ID:GI929-096 MECN WASH-1
File:GI929-096 MECN WASH-1 Date:16-May-2014 Time:16:34:25
Description:

Printed: Tue Mar 08 14:08:15 2022

Sample Report (continued):

Peak Number	Compound	Time	AreaAbs	Area %Total	Width	Height	Mass Found
5		1.40	2e+004	100.00	0	8e+005	

Peak ID	Compound	Time	Mass Found
4		1.40	Not Found

4: (Time: 1.40) Combine (330:334) 1:MS ES+
3.7e+007

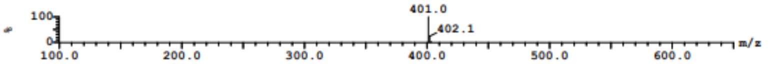

1-(3-methyl-4-(4-morpholinophenyl)-1-phenyl-1H-pyrazolo[3,4-b]pyridin-6-yl)ethan-1-ol (31).

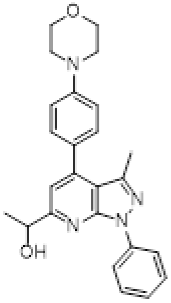

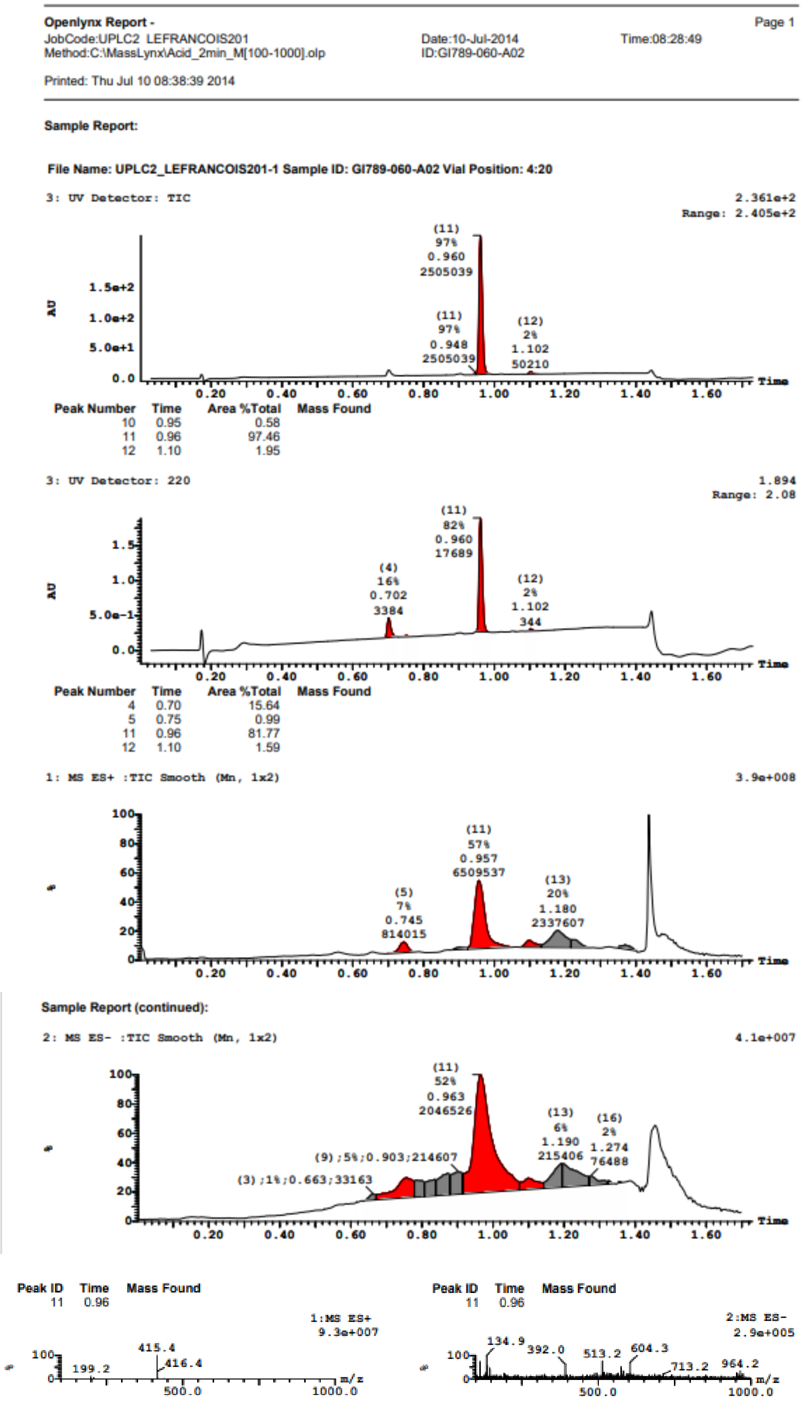

Openlynx Report - JobCode:UPLC2 LEFRANCOIS201 Method:C:\MassLynx\Acid_2min_M[100-1000].olp Date:10-Jul-2014 ID:GI789-060-A02 Time:08:28:49 Page 1

Printed: Thu Jul 10 08:38:39 2014

Sample Report:

File Name: UPLC2_LEFRANCOIS201-1 Sample ID: GI789-060-A02 Vial Position: 4:20

3: UV Detector: TIC

Peak Number	Time	Area %Total	Mass Found
10	0.95	0.58	
11	0.96	97.46	
12	1.10	1.95	

3: UV Detector: 220

Peak Number	Time	Area %Total	Mass Found
4	0.70	15.64	
5	0.75	0.99	
11	0.96	81.77	
12	1.10	1.59	

1: MS ES+ :TIC Smooth (Mn, 1x2)

Sample Report (continued):

2: MS ES- :TIC Smooth (Mn, 1x2)

Peak ID	Time	Mass Found
11	0.96	

Peak ID	Time	Mass Found
11	0.96	

2-(3-methyl-4-(4-morpholinophenyl)-1-phenyl-1H-pyrazolo[3,4-b]pyridin-6-yl)propan-2-ol (32).

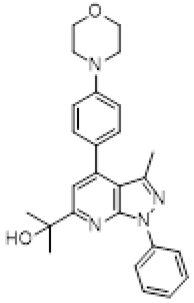

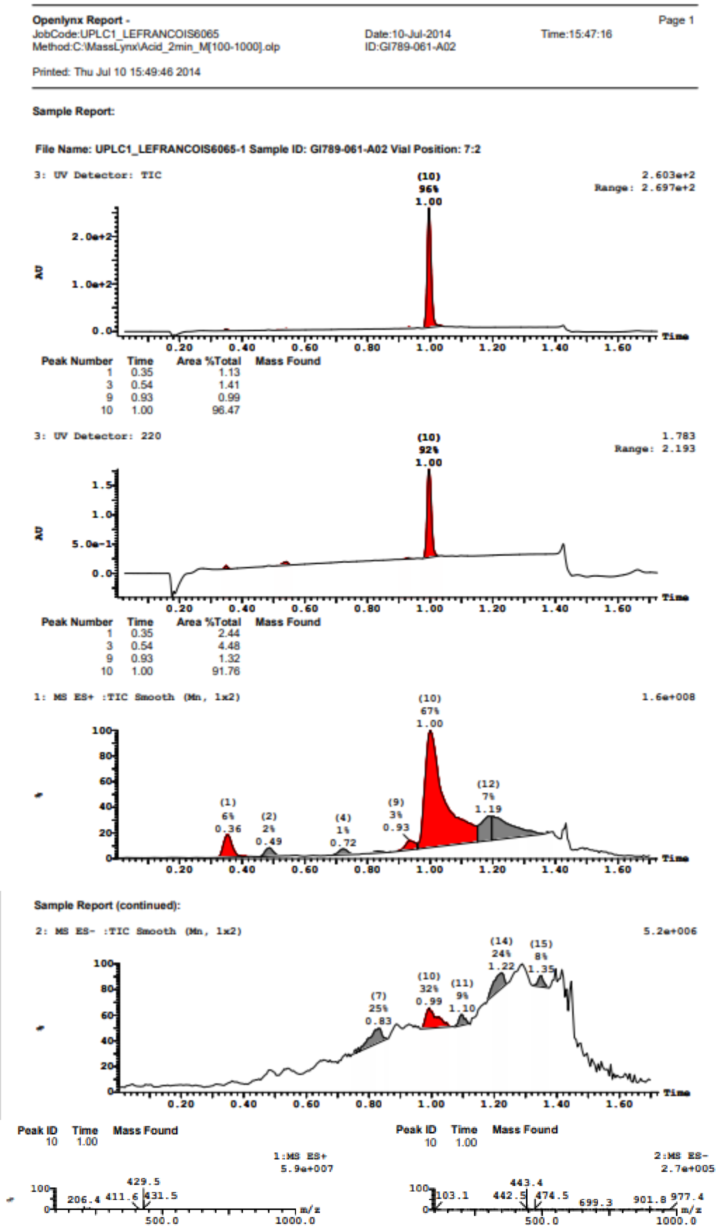

3-methyl-4-(4-morpholinophenyl)-1-phenyl-N-(2,2,2-trifluoroethyl)-1H-pyrazolo[3,4-b]pyridine-6-carboxamide (33).

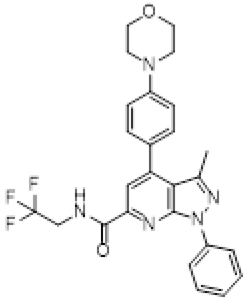

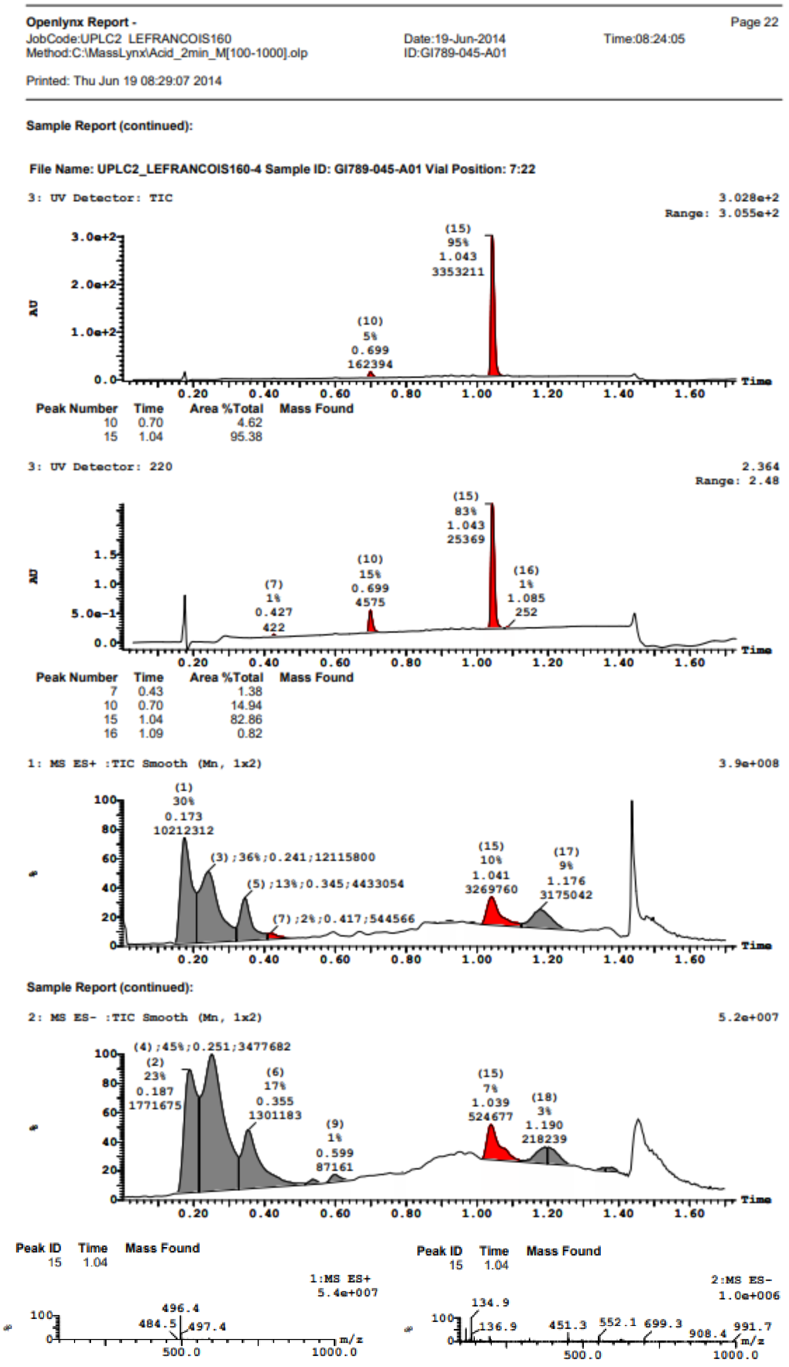

4-(4-(6-(5-methoxy-1H-pyrazol-3-yl)-3-methyl-1-phenyl-1H-pyrazolo[3,4-b]pyridin-4-yl)phenyl)morpholine (34).

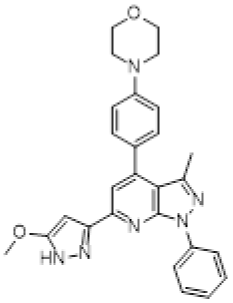

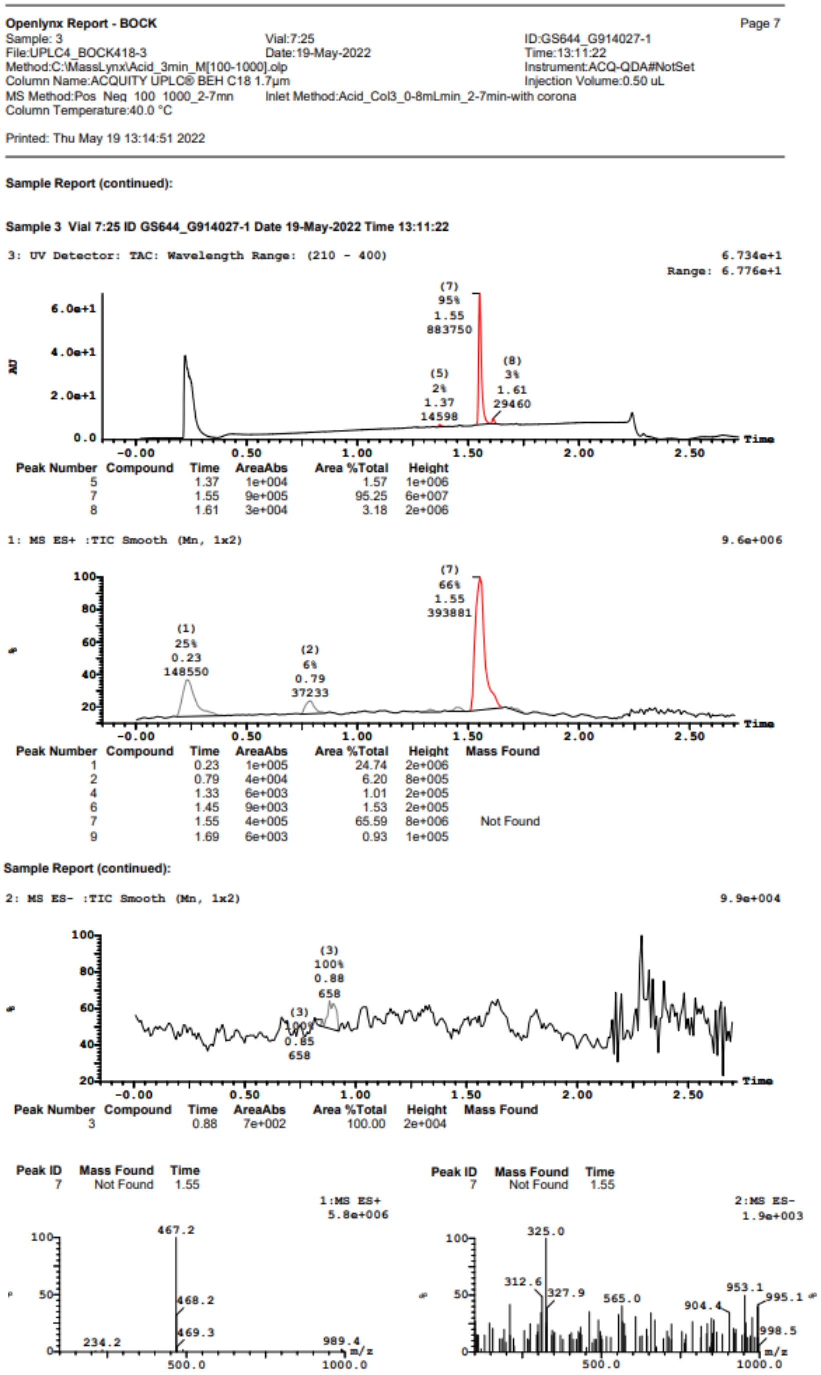

Openlynx Report - BOCK
Sample: 3 Vial:7:25 ID:GS644_G914027-1
File:UPLC4_BOCK418-3 Date:19-May-2022 Time:13:11:22
Method:C:\MassLynx\Acid_3min_M[100-1000].olp Instrument:ACQ-QDA#NotSet
Column Name:ACQUITY UPLC® BEH C18 1.7µm Injection Volume:0.50 uL
MS Method:Pos_Neg_100_1000_2-7mn Inlet Method:Acid_Col3_0-8mLmin_2-7min-with corona
Column Temperature:40.0 °C

Page 7

Printed: Thu May 19 13:14:51 2022

Sample Report (continued):

Sample 3 Vial 7:25 ID GS644_G914027-1 Date 19-May-2022 Time 13:11:22

3: UV Detector: TAC: Wavelength Range: (210 - 400)

Peak Number	Compound	Time	AreaAbs	Area %Total	Height
5		1.37	1e+004	1.57	1e+006
7		1.55	9e+005	95.25	6e+007
8		1.61	3e+004	3.18	2e+006

1: MS ES+ :TIC Smooth (Mn, 1x2)

Peak Number	Compound	Time	AreaAbs	Area %Total	Height	Mass Found
1		0.23	1e+005	24.74	2e+006	
2		0.79	4e+004	6.20	8e+005	
4		1.33	6e+003	1.01	2e+005	
6		1.45	9e+003	1.53	2e+005	
7		1.55	4e+005	65.59	8e+006	Not Found
9		1.69	6e+003	0.93	1e+005	

Sample Report (continued):

2: MS ES- :TIC Smooth (Mn, 1x2)

Peak Number	Compound	Time	AreaAbs	Area %Total	Height	Mass Found
3		0.88	7e+002	100.00	2e+004	

Peak ID	Mass Found	Time
7	Not Found	1.55

Peak ID	Mass Found	Time
7	Not Found	1.55

2-(3-methyl-4-(4-morpholinophenyl)-1-phenyl-1H-pyrazolo[3,4-b]pyridin-6-yl)acetic acid (35).

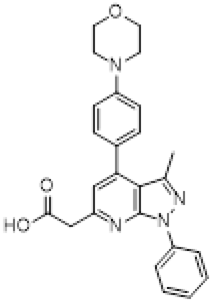

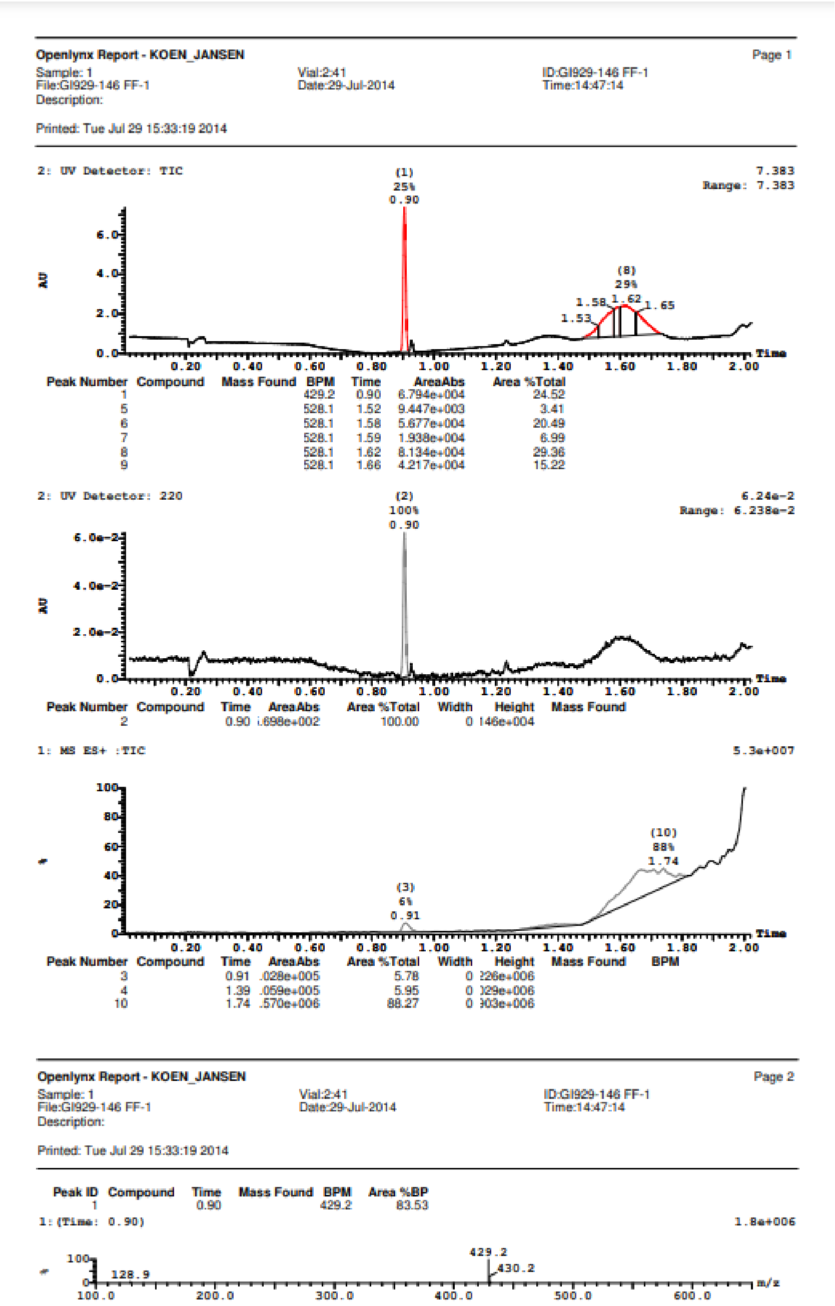

N-cyano-3-methyl-4-(4-morpholinophenyl)-1-phenyl-1H-pyrazolo[3,4-b]pyridine-6-carboxamide (36).

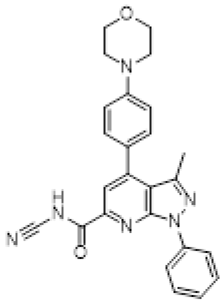

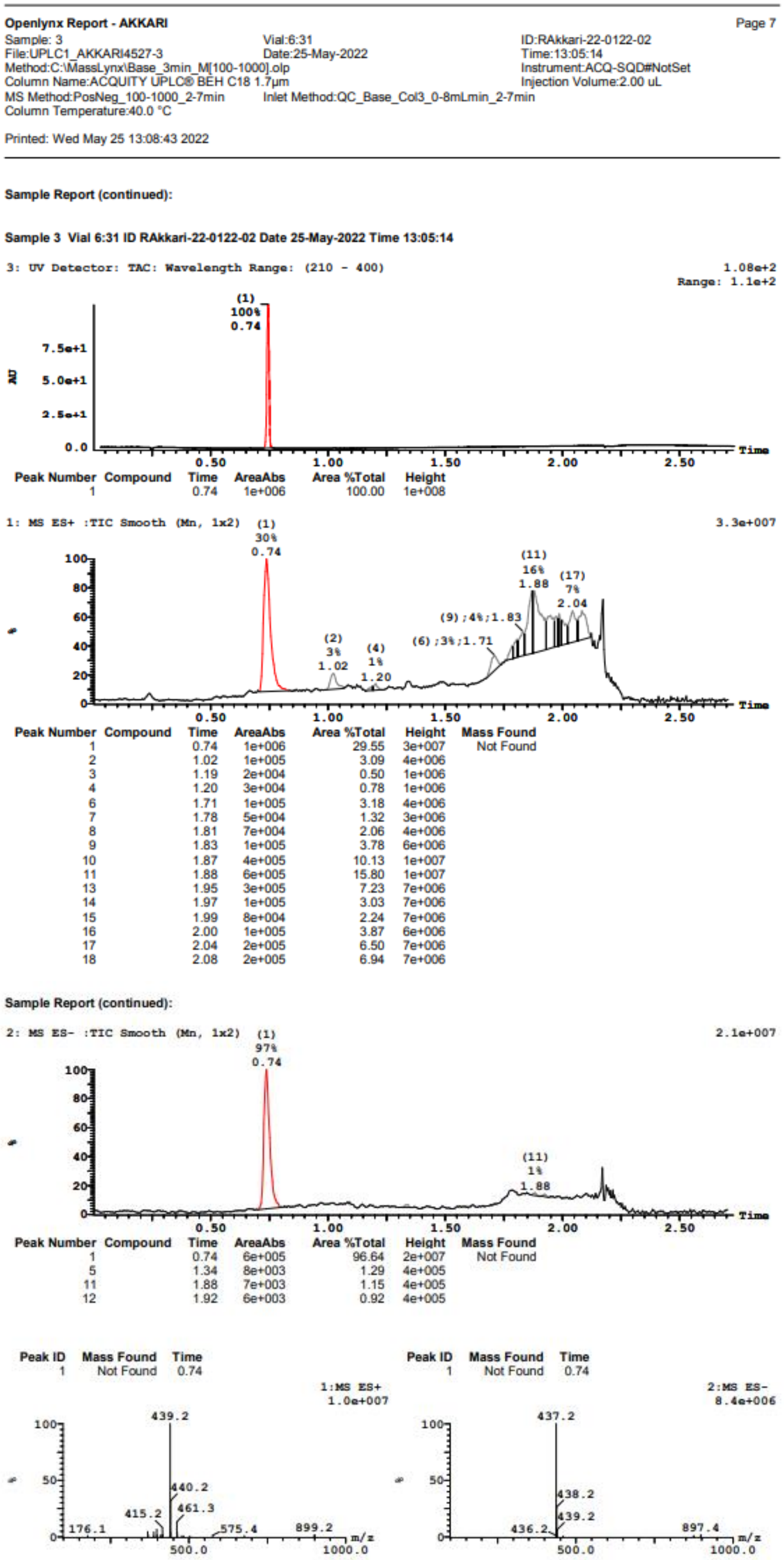

Openlynx Report - AKKARI   Page 7

Sample: 3   Vial:6:31   ID:RAkkari-22-0122-02
File:UPLC1_AKKARI4527-3   Date:25-May-2022   Time:13:05:14
Method:C:\MassLynx\Base_3min_M[100-1000].olp   Instrument:ACQ-SQD#NotSet
Column Name:ACQUITY UPLC® BEH C18 1.7µm   Injection Volume:2.00 uL
MS Method:PosNeg_100-1000_2-7min   Inlet Method:QC_Base_Col3_0-8mLmin_2-7min
Column Temperature:40.0 °C

Printed: Wed May 25 13:08:43 2022

Sample Report (continued):

Sample 3 Vial 6:31 ID RAkkari-22-0122-02 Date 25-May-2022 Time 13:05:14

3: UV Detector: TAC: Wavelength Range: (210 - 400)   1.08e+2
Range: 1.1e+2

Peak Number	Compound	Time	AreaAbs	Area %Total	Height
1		0.74	1e+006	100.00	1e+008

1: MS ES+ :TIC Smooth (Mn, 1x2)   3.3e+007

Peak Number	Compound	Time	AreaAbs	Area %Total	Height	Mass Found
1		0.74	1e+006	29.55	3e+007	Not Found
2		1.02	1e+005	3.09	4e+006	
3		1.19	2e+004	0.50	1e+006	
4		1.20	3e+004	0.78	1e+006	
6		1.71	1e+005	3.18	4e+006	
7		1.78	5e+004	1.32	3e+006	
8		1.81	7e+004	2.06	4e+006	
9		1.83	1e+005	3.78	6e+006	
10		1.87	4e+005	10.13	1e+007	
11		1.88	6e+005	15.80	1e+007	
13		1.95	3e+005	7.23	7e+006	
14		1.97	1e+005	3.03	7e+006	
15		1.99	8e+004	2.24	7e+006	
16		2.00	1e+005	3.87	6e+006	
17		2.04	2e+005	6.50	7e+006	
18		2.08	2e+005	6.94	7e+006	

Sample Report (continued):

2: MS ES- :TIC Smooth (Mn, 1x2)   2.1e+007

Peak Number	Compound	Time	AreaAbs	Area %Total	Height	Mass Found
1		0.74	6e+005	96.64	2e+007	Not Found
5		1.34	8e+003	1.29	4e+005	
11		1.88	7e+003	1.15	4e+005	
12		1.92	6e+003	0.92	4e+005	

Peak ID	Mass Found	Time
1	Not Found	0.74

1:MS ES+ 1.0e+007

Peak ID	Mass Found	Time
1	Not Found	0.74

2:MS ES- 8.4e+006

3-methyl-N-(methylsulfonyl)-4-(4-morpholinophenyl)-1-phenyl-1H-pyrazolo[3,4-b]pyridine-6-carboxamide (37).

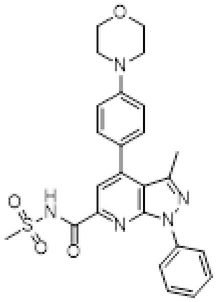

Openlynx Report - KOEN_JANSEN Page 1
Sample: 1 Vial:1:47 ID:GI929-093 F-1
File:GI929-093 F-1 Date:15-May-2014 Time:16:07:56
Description:

Printed: Tue Mar 08 14:07:12 2022

Sample Report:

Sample 1 Vial 1:47 ID GI929-093 F-1 File GI929-093 F-1 Date 15-May-2014 Time 16:07:56 Description

1: MS ES+ :TIC Smooth (Mn, 1x1) 2.6e+007

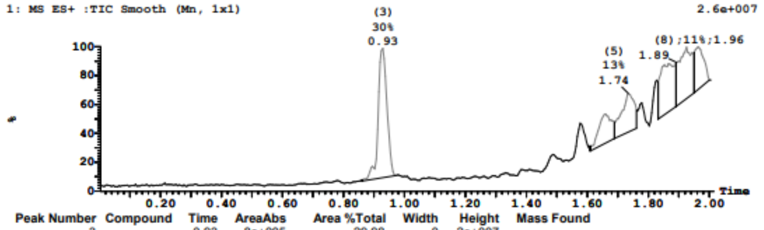

Peak Number	Compound	Time	AreaAbs	Area %Total	Width	Height	Mass Found
3		0.93	8e+005	29.98	0	2e+007	
4		1.66	3e+005	10.78	0	5e+006	
5		1.74	4e+005	13.45	0	7e+006	
6		1.87	4e+005	17.05	0	9e+006	
7		1.92	5e+005	17.71	0	9e+006	
8		1.96	3e+005	11.03	0	8e+006	

2: UV Detector: TAC: Wavelength Range: (210 - 400) 8.679e+1
Range: 8.677e+1

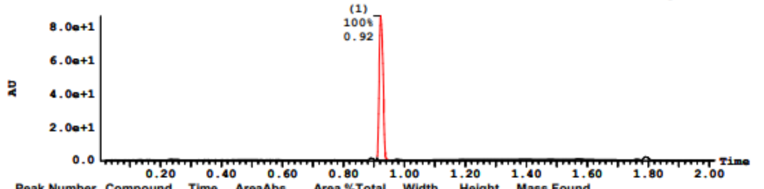

Peak Number	Compound	Time	AreaAbs	Area %Total	Width	Height	Mass Found
1		0.92	1e+006	100.00	0	9e+007	Not Found

2: UV Detector: 220 Nm 6.417e-1
Range: 6.417e-1

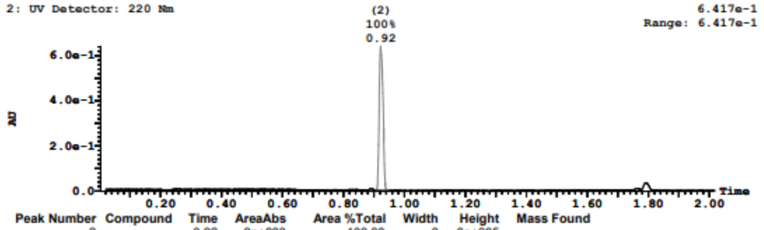

Peak Number	Compound	Time	AreaAbs	Area %Total	Width	Height	Mass Found
2		0.92	9e+003	100.00	0	6e+005	

Sample Report (continued):

Peak ID	Compound	Time	Mass Found
1		0.92	Not Found

1:(Time: 0.92) Combine (217:222) 1:MS ES+ 1.6e+007

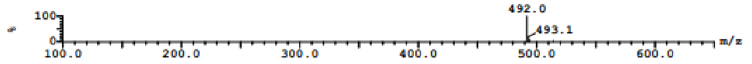

N-(ethylsulfonyl)-3-methyl-4-(4-morpholinophenyl)-1-phenyl-1H-pyrazolo[3,4-b]pyridine-6-carboxamide (38).

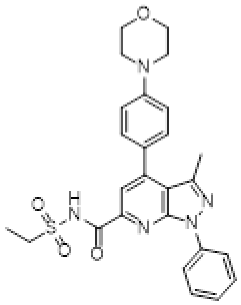

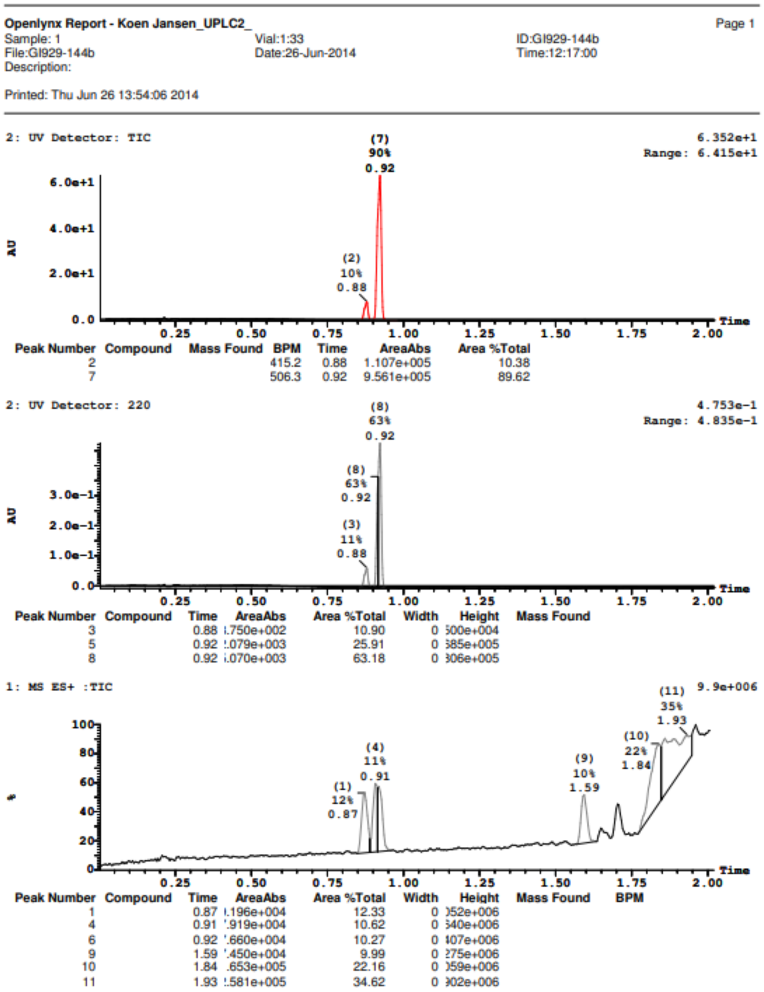

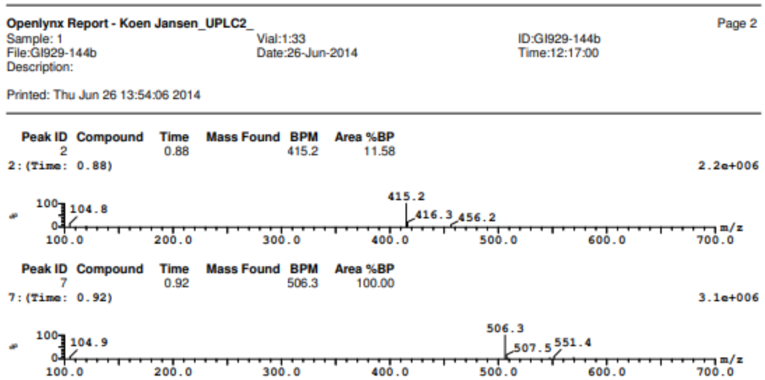

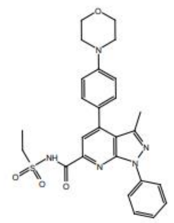

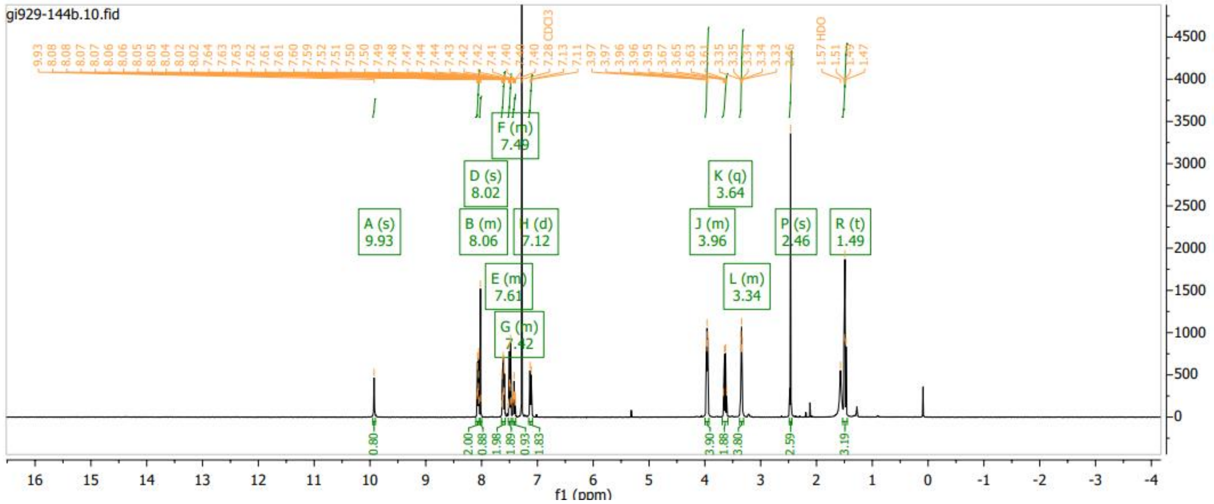

gi929-144b.10.fid
A (s)
9.93
B (m)
8.06
D (s)
8.02
E (m)
7.61
F (m)
7.49
G (m)
7.42
H (d)
7.12
J (m)
3.96
K (q)
3.64
L (m)
3.34
P (s)
2.46
R (t)
1.49
f1 (ppm)

3-cyclobutyl-N-(methylsulfonyl)-4-(4-morpholinophenyl)-1-phenyl-1H-pyrazolo[3,4-b]pyridine-6-carboxamide (39).

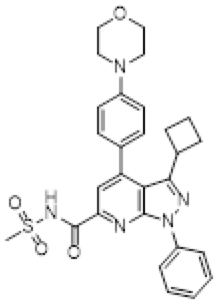

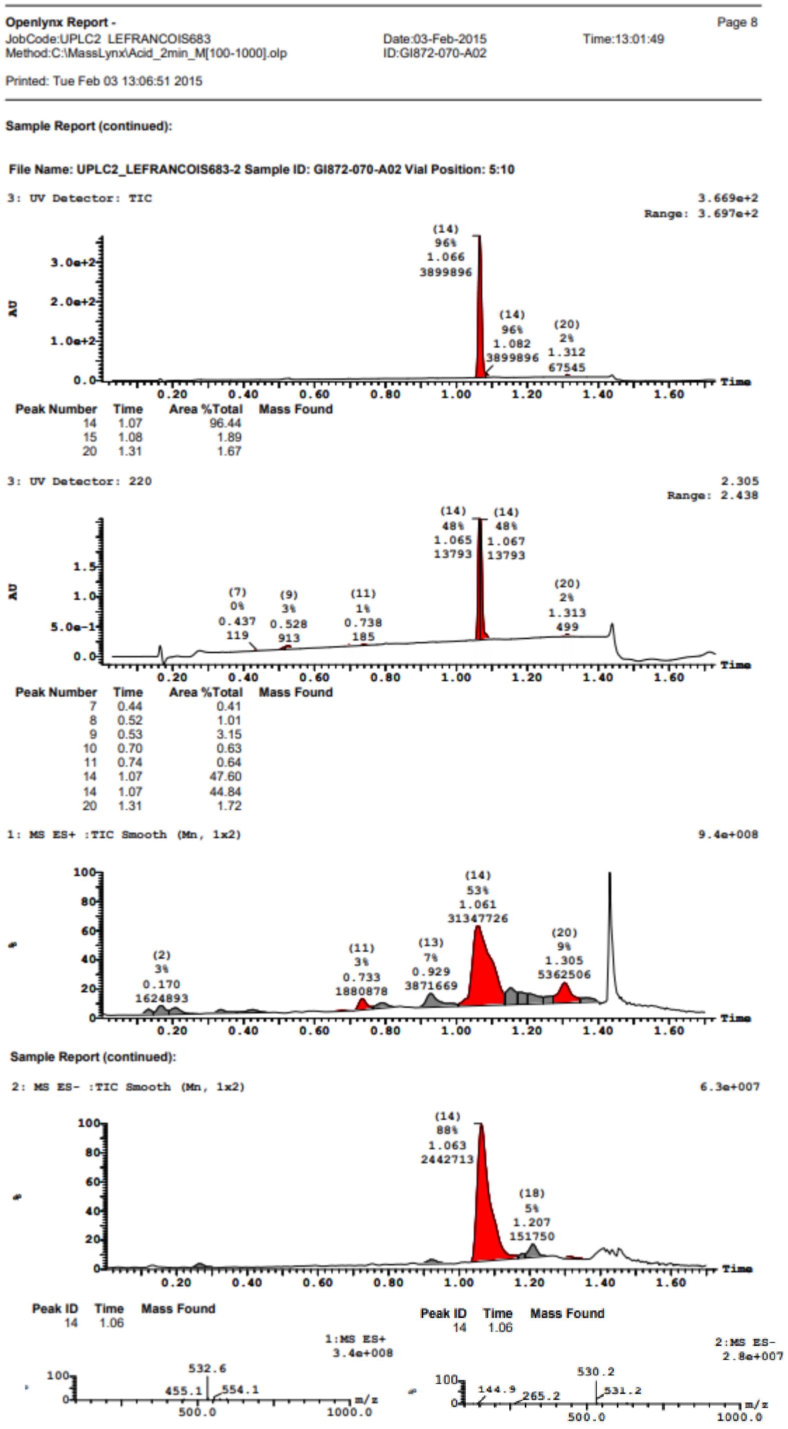

Openlynx Report - Page 8
JobCode:UPLC2 LEFRANCOIS683 Date:03-Feb-2015 Time:13:01:49
Method:C:\MassLynx\Acid_2min_M[100-1000].olp ID:GI872-070-A02
Printed: Tue Feb 03 13:06:51 2015

Sample Report (continued):

File Name: UPLC2_LEFRANCOIS683-2 Sample ID: GI872-070-A02 Vial Position: 5:10

3: UV Detector: TIC

Peak Number	Time	Area %Total	Mass Found
14	1.07	96.44	
15	1.08	1.89	
20	1.31	1.67	

3: UV Detector: 220

Peak Number	Time	Area %Total	Mass Found
7	0.44	0.41	
8	0.52	1.01	
9	0.53	3.15	
10	0.70	0.63	
11	0.74	0.64	
14	1.07	47.60	
14	1.07	44.84	
20	1.31	1.72	

1: MS ES+ :TIC Smooth (Mn, 1x2)

Sample Report (continued):

2: MS ES- :TIC Smooth (Mn, 1x2)

Peak ID	Time	Mass Found
14	1.06	

Peak ID	Time	Mass Found
14	1.06	

3-cyclobutyl-4-(4-((2-methoxyethyl)(methyl)amino)phenyl)-1-phenyl-1H-pyrazolo[3,4-b]pyridine-6-carboxylic acid (40).

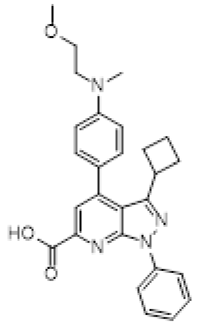

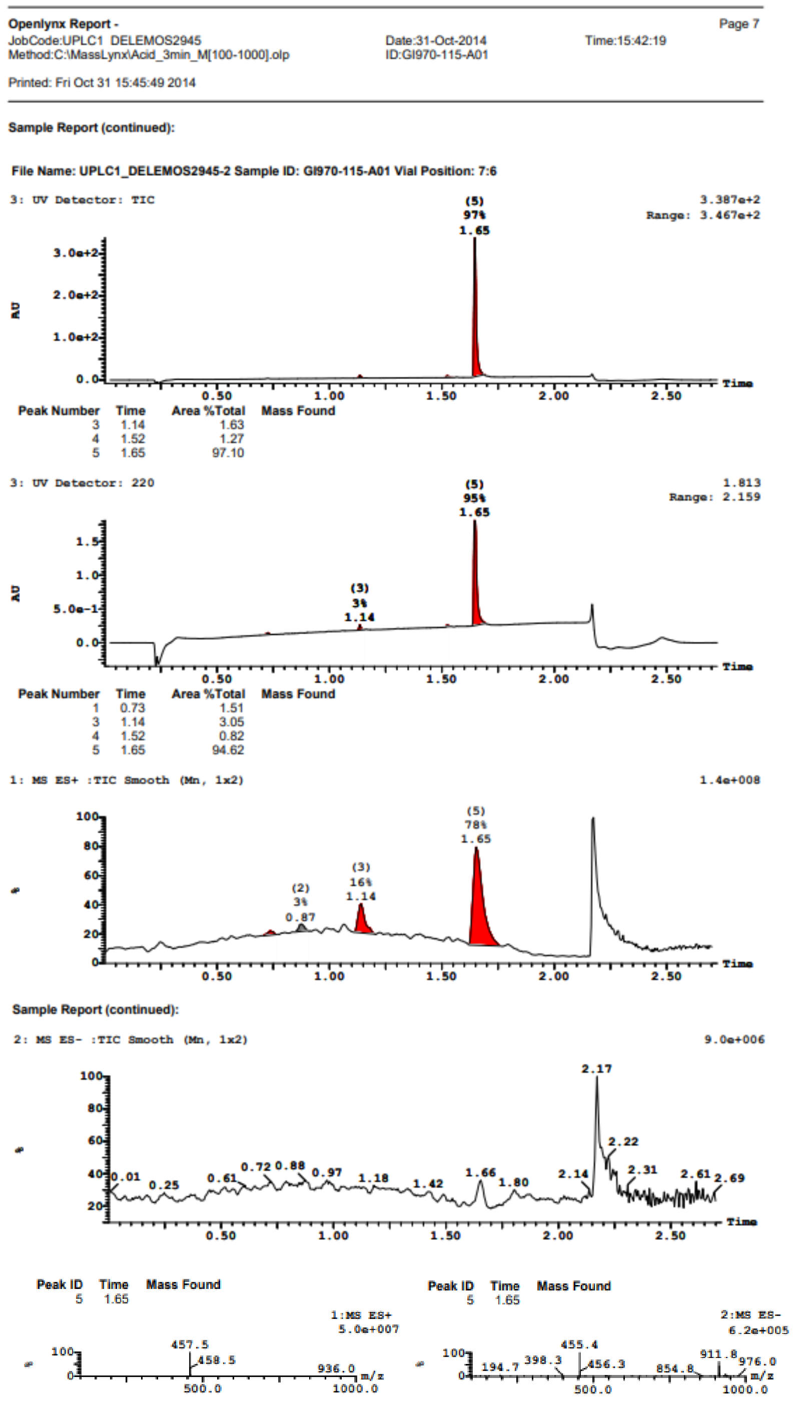

3-cyclobutyl-4-(4-((2-methoxyethyl)(methyl)amino)phenyl)-N-(methylsulfonyl)-1-phenyl-1H-pyrazolo[3,4-b]pyridine-6-carboxamide (41).

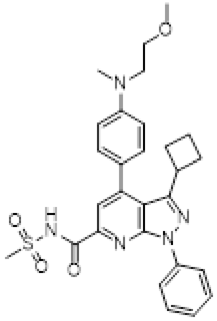

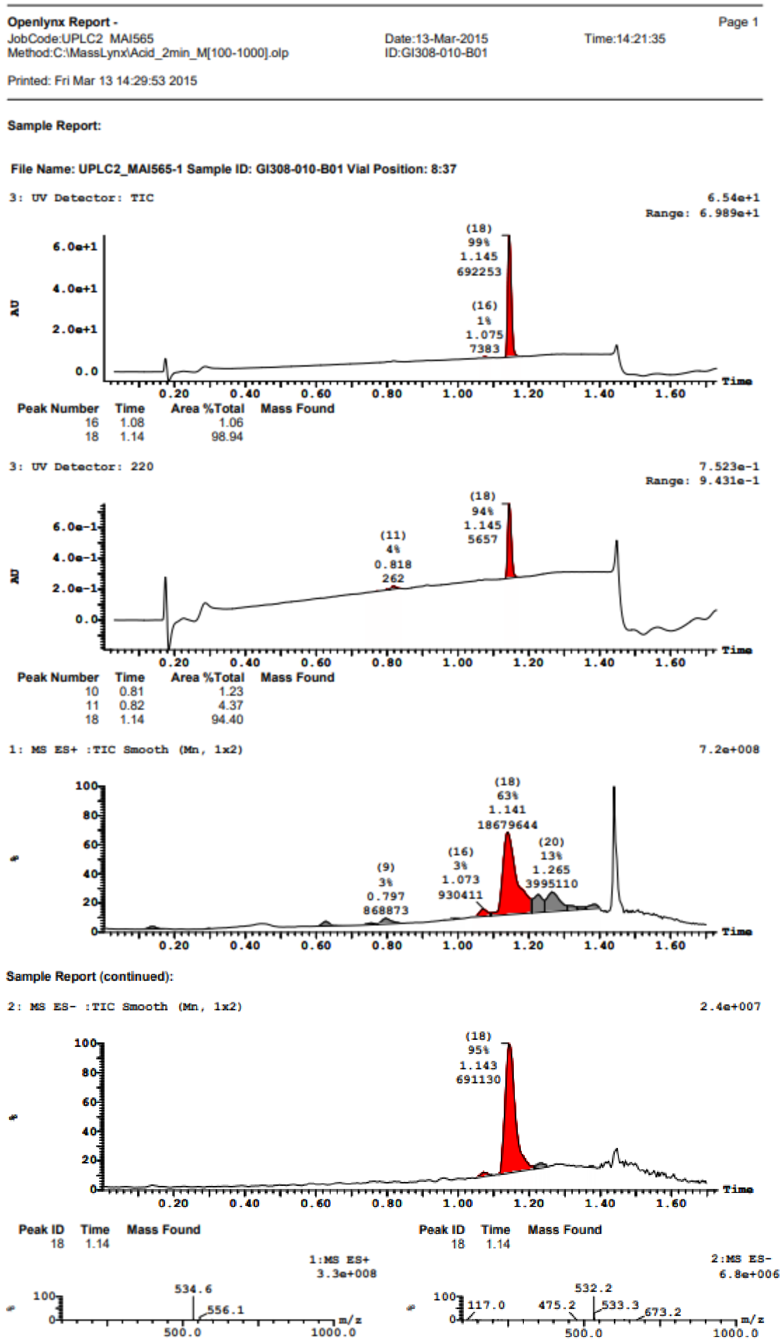

3-cyclobutyl-4-(6-((2-methoxyethyl)(methyl)amino)pyridin-3-yl)-1-phenyl-1H-pyrazolo[3,4-b]pyridine-6-carboxylic acid (42).

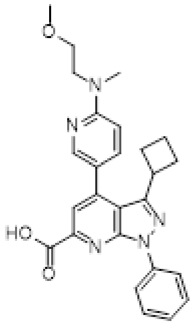

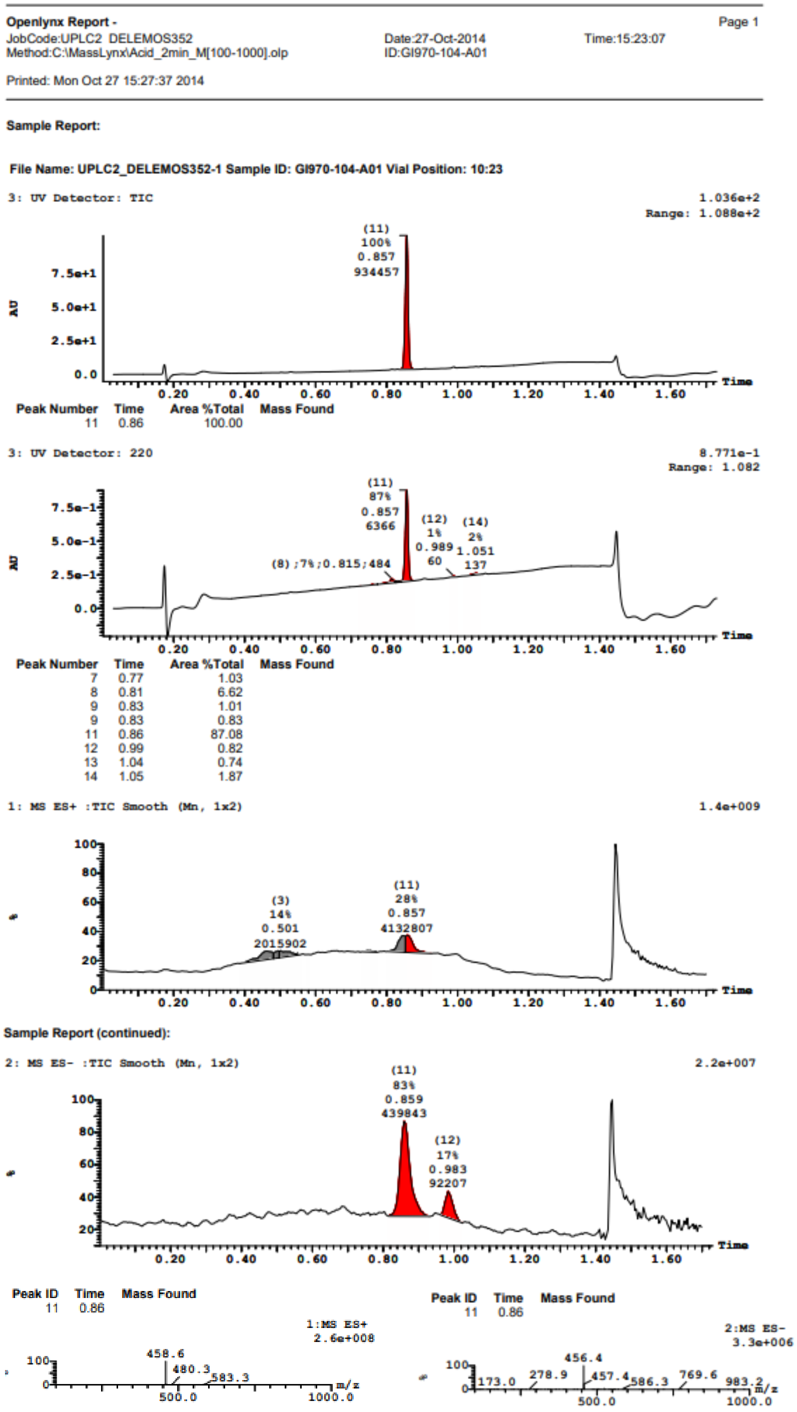

3-cyclobutyl-4-(6-((2-methoxyethyl)(methyl)amino)pyridin-3-yl)-N-(methylsulfonyl)-1-phenyl-1H-pyrazolo[3,4-b]pyridine-6-carboxamide (43).

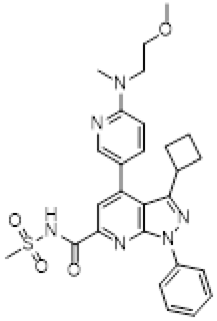

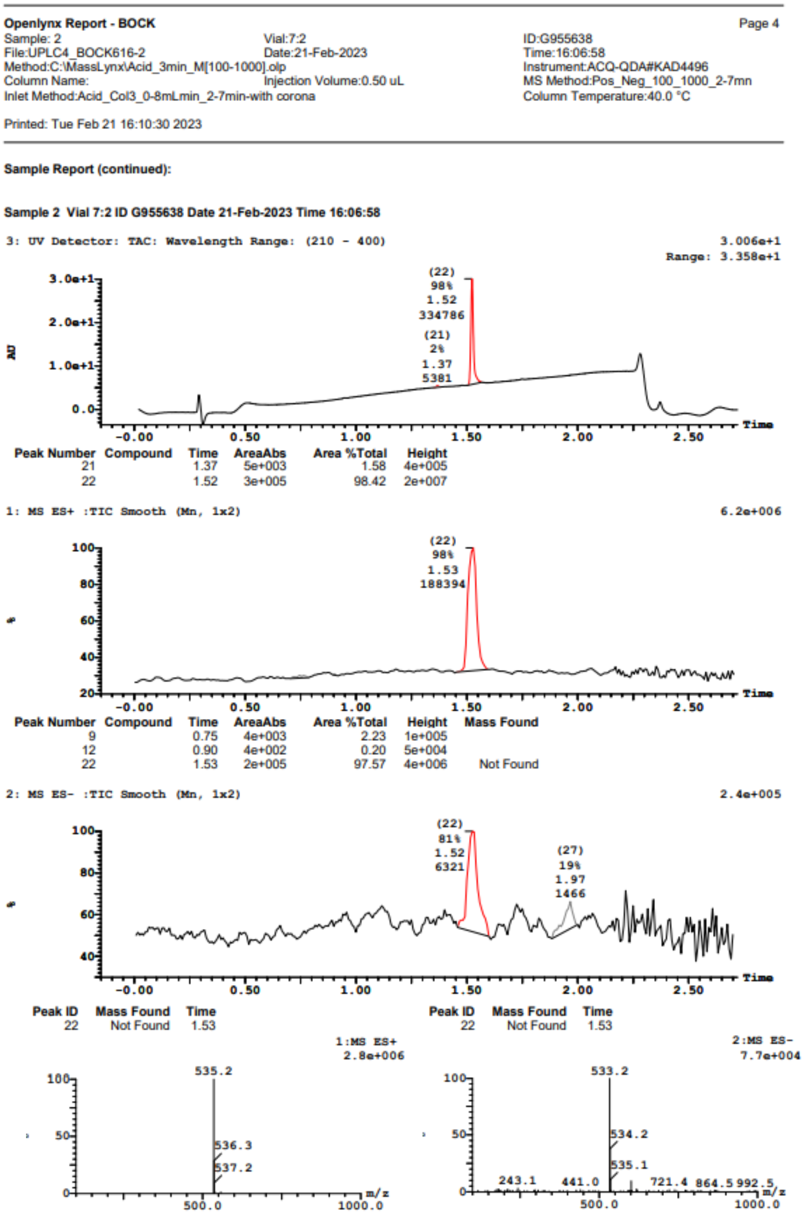

Openlynx Report - BOCK Page 4
Sample: 2 Vial:7:2 ID:G955638
File:UPLC4_BOCK616-2 Date:21-Feb-2023 Time:16:06:58
Method:C:\MassLynx\Acid_3min_M[100-1000].olp Instrument:ACQ-QDA#KAD4496
Column Name: Injection Volume:0.50 uL MS Method:Pos_Neg_100_1000_2-7mn
Inlet Method:Acid_Col3_0-8mLmin_2-7min-with corona Column Temperature:40.0 °C

Printed: Tue Feb 21 16:10:30 2023

Sample Report (continued):

Sample 2 Vial 7:2 ID G955638 Date 21-Feb-2023 Time 16:06:58

3: UV Detector: TAC: Wavelength Range: (210 - 400) 3.006e+1 Range: 3.358e+1

Peak Number	Compound	Time	AreaAbs	Area %Total	Height
21		1.37	5e+003	1.58	4e+005
22		1.52	3e+005	98.42	2e+007

1: MS ES+ :TIC Smooth (Mn, 1x2) 6.2e+006

Peak Number	Compound	Time	AreaAbs	Area %Total	Height	Mass Found
9		0.75	4e+003	2.23	1e+005	
12		0.90	4e+002	0.20	5e+004	
22		1.53	2e+005	97.57	4e+006	Not Found

2: MS ES- :TIC Smooth (Mn, 1x2) 2.4e+005

Peak ID	Mass Found	Time
22	Not Found	1.53

Peak ID	Mass Found	Time
22	Not Found	1.53

1:MS ES+ 2.8e+006

2:MS ES- 7.7e+004

4-(6-(4-cyanopiperidin-1-yl)pyridin-3-yl)-3-cyclobutyl-1-phenyl-1H-pyrazolo[3,4-b]pyridine-6-carboxylic acid (44).

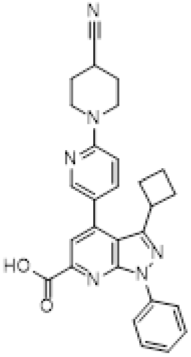

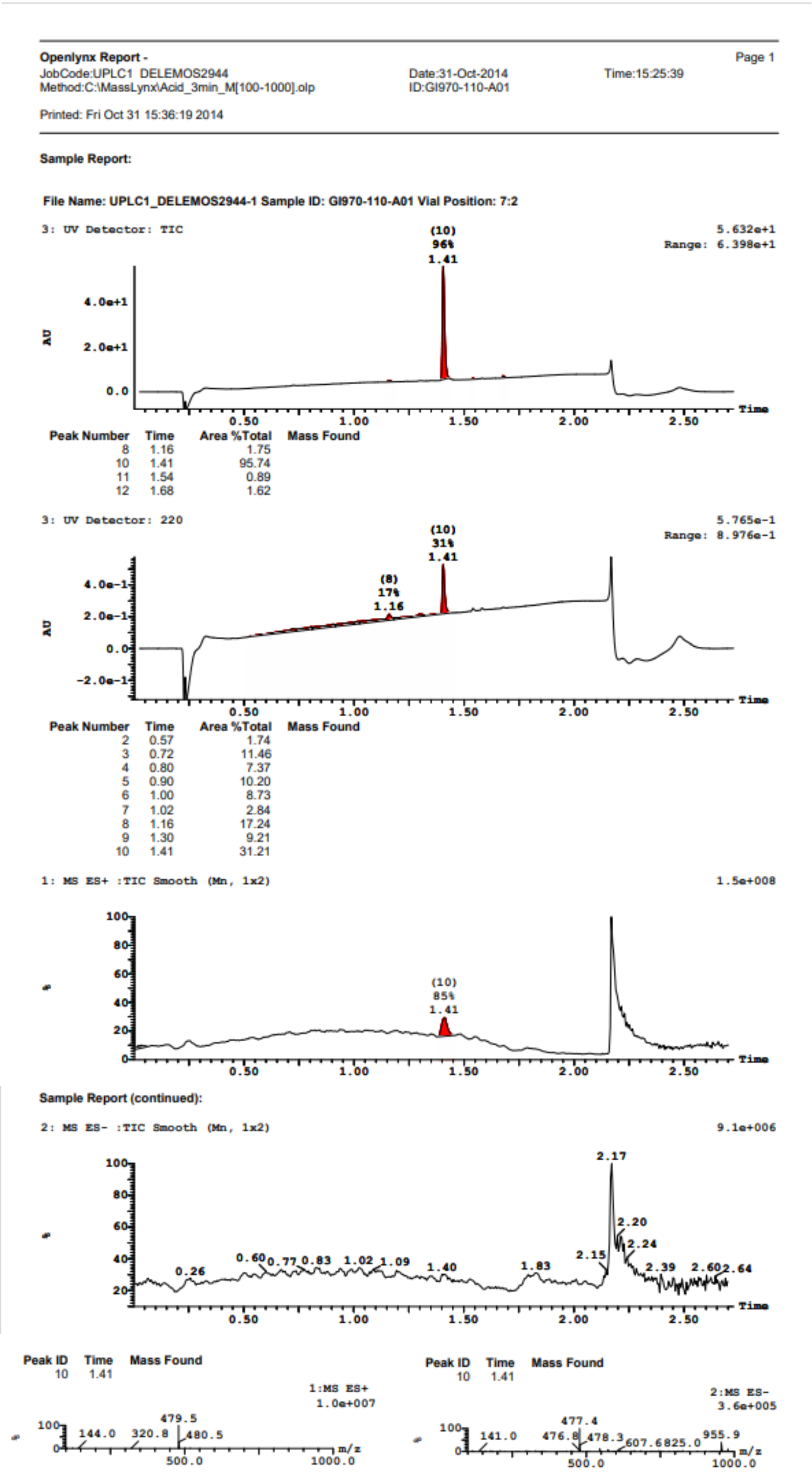

4-(6-(4-cyanopiperidin-1-yl)pyridin-3-yl)-3-cyclobutyl-N-(methylsulfonyl)-1-phenyl-1H-pyrazolo[3,4-b]pyridine-6-carboxamide (45).

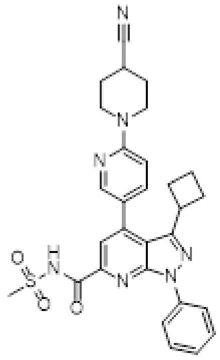

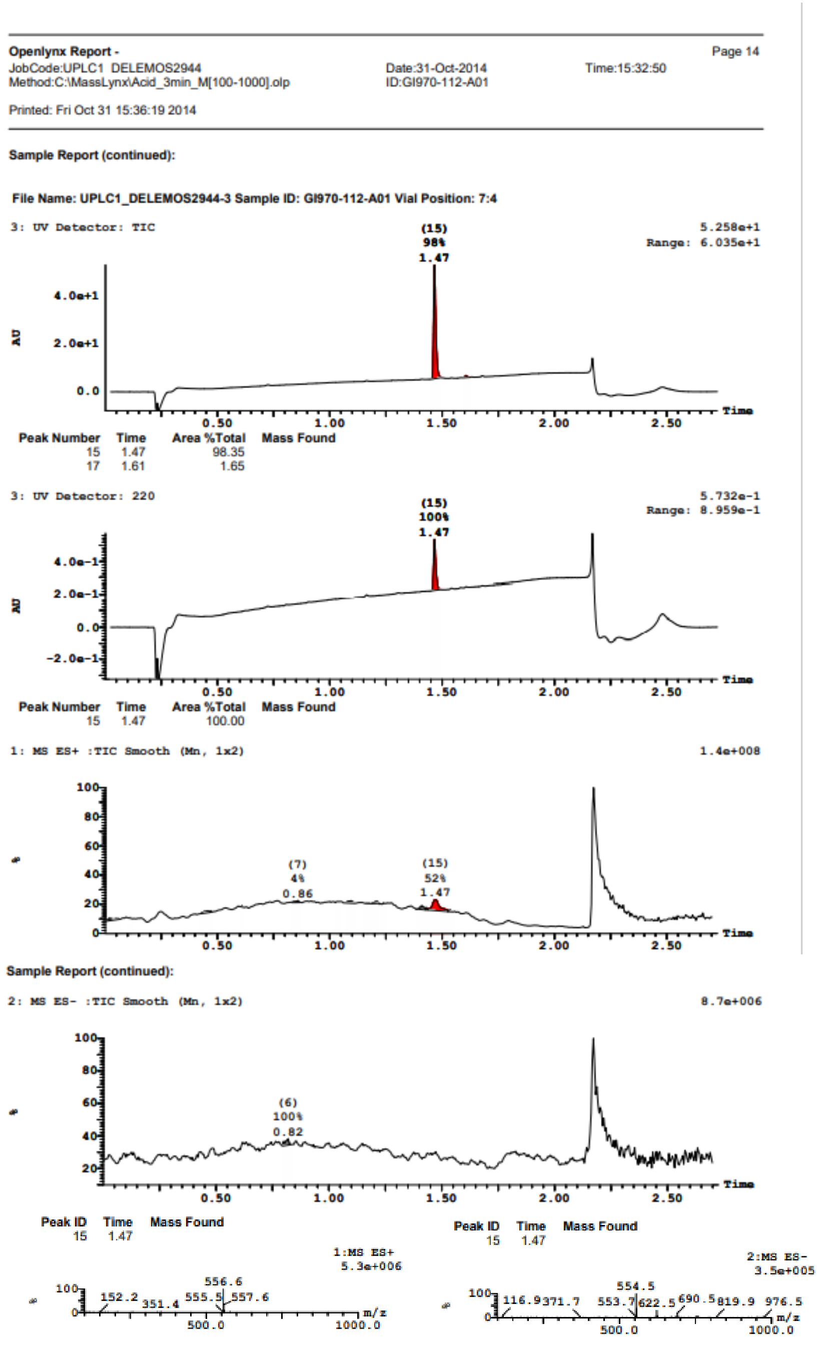

Synthesis of 3-cyclobutyl-N-(methylsulfonyl)-1-phenyl-4-(piperidin-1-yl)-1H-pyrazolo[3,4-b]pyridine-6-carboxamide (46)

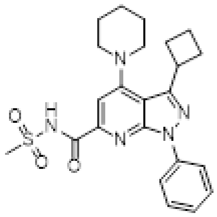

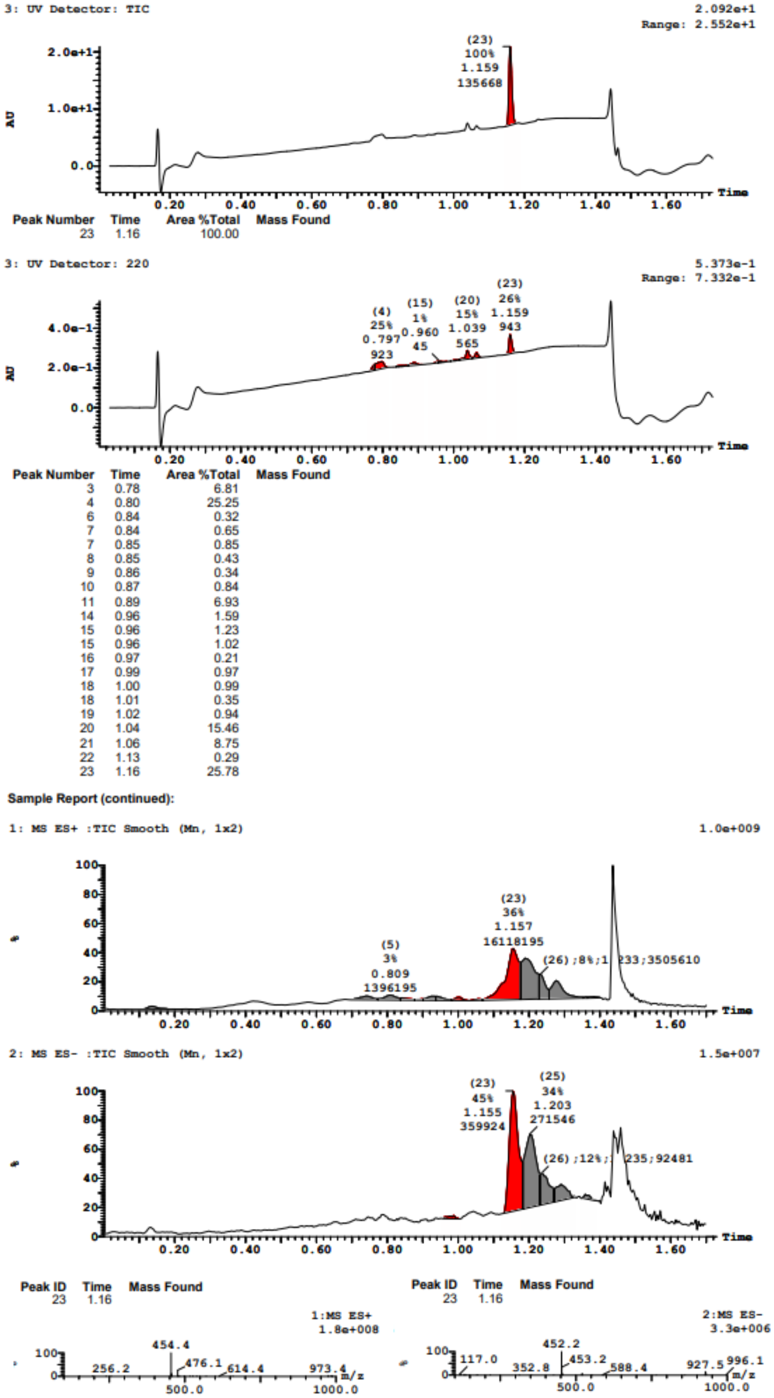

Openlynx Report - Page 1
JobCode:UPLC2 PELTIER382 Date:10-Mar-2015 Time:17:47:10
Method:C:\MassLynx\Acid_2min_M[100-1000].olp ID:GI261-071-A01
Printed: Tue Mar 10 17:55:34 2015

Sample Report:

File Name: UPLC2_PELTIER382-1 Sample ID: GI261-071-A01 Vial Position: 4:27

3: UV Detector: TIC 2.092e+1 Range: 2.552e+1

Peak Number	Time	Area %Total	Mass Found
23	1.16	100.00	

3: UV Detector: 220 5.373e-1 Range: 7.332e-1

Peak Number	Time	Area %Total	Mass Found
3	0.78	6.81	
4	0.80	25.25	
6	0.84	0.32	
7	0.84	0.65	
7	0.85	0.85	
8	0.85	0.43	
9	0.86	0.34	
10	0.87	0.84	
11	0.89	6.93	
14	0.96	1.59	
15	0.96	1.23	
15	0.96	1.02	
16	0.97	0.21	
17	0.99	0.97	
18	1.00	0.99	
18	1.01	0.35	
19	1.02	0.94	
20	1.04	15.46	
21	1.06	8.75	
22	1.13	0.29	
23	1.16	25.78	

Sample Report (continued):

1: MS ES+ :TIC Smooth (Mn, 1x2) 1.0e+009

2: MS ES- :TIC Smooth (Mn, 1x2) 1.5e+007

Peak ID	Time	Mass Found
23	1.16	

1:MS ES+ 1.8e+008

Peak ID	Time	Mass Found
23	1.16	

2:MS ES- 3.3e+006

3-Cyclobutyl-4-(4-methoxypiperidin-1-yl)-N-(methylsulfonyl)-1-phenyl-1H-pyrazolo[3,4-b]pyridine-6-carboxamide (47)

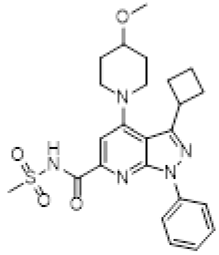

Openlynx Report - Page 1
JobCode:UPLC2_NEWSOME422 Date:28-Jan-2015 Time:08:46:25
Method:C:\MassLynx\Acid_2min_M[100-1000].olp ID:GI953-145-B01

Printed: Wed Jan 28 08:49:37 2015

Sample Report:

File Name: UPLC2_NEWSOME422-1 Sample ID: GI953-145-B01 Vial Position: 7:30

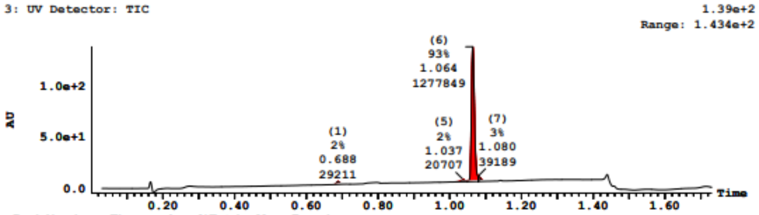

Peak Number	Time	Area %Total	Mass Found
1	0.69	2.14	
5	1.04	1.51	
6	1.06	93.48	
7	1.08	2.87	

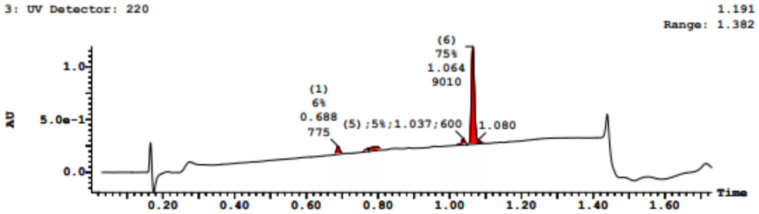

Peak Number	Time	Area %Total	Mass Found
1	0.69	6.48	
2	0.77	2.14	
3	0.80	8.39	
4	1.03	0.63	
5	1.04	5.02	
6	1.06	75.33	
7	1.08	2.02	

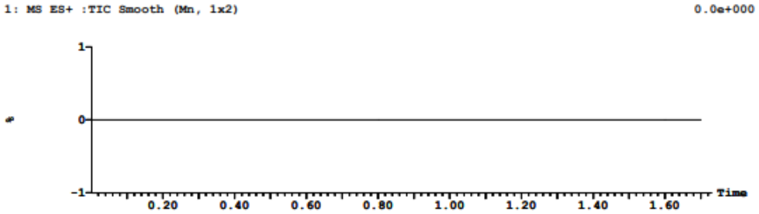

Sample Report (continued):

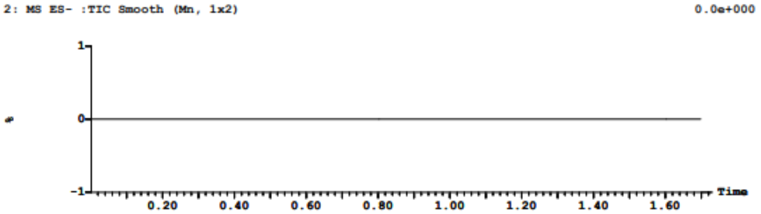

47: ^{1}H NMR (400 MHz, $CDCl_3$) δ 10.06 (s, 1H), 8.11 – 8.03 (m, 2H), 7.64 – 7.54 (m, 2H), 7.50 (s, 1H), 7.43 – 7.34 (m, 1H), 4.03 (p, J = 8.5 Hz, 1H), 3.57 (dtd, J = 16.1, 7.3, 3.9 Hz, 3H), 3.47 (s, 3H), 3.43 (s, 3H), 3.15 (ddd, J = 12.3, 8.9, 3.1 Hz, 2H), 2.66 (pd, J = 9.2, 2.5 Hz, 2H), 2.45 (dddd, J = 14.0, 11.7, 7.4, 2.6 Hz, 2H), 2.23 – 2.14 (m, 2H), 2.16 – 2.01 (m, 2H), 1.92 (dtd, J = 12.4, 8.5, 3.5 Hz, 2H).

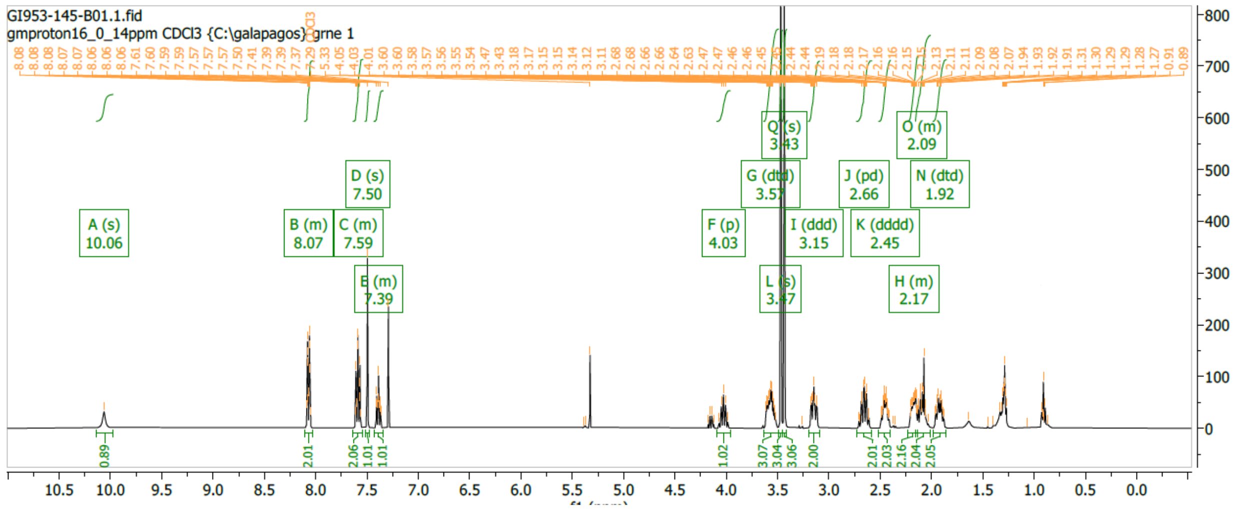

4-(4-cyanopiperidin-1-yl)-3-cyclobutyl-N-(methylsulfonyl)-1-phenyl-1H-pyrazolo[3,4-b]pyridine-6-carboxamide (48)

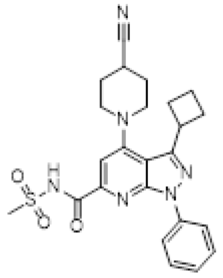

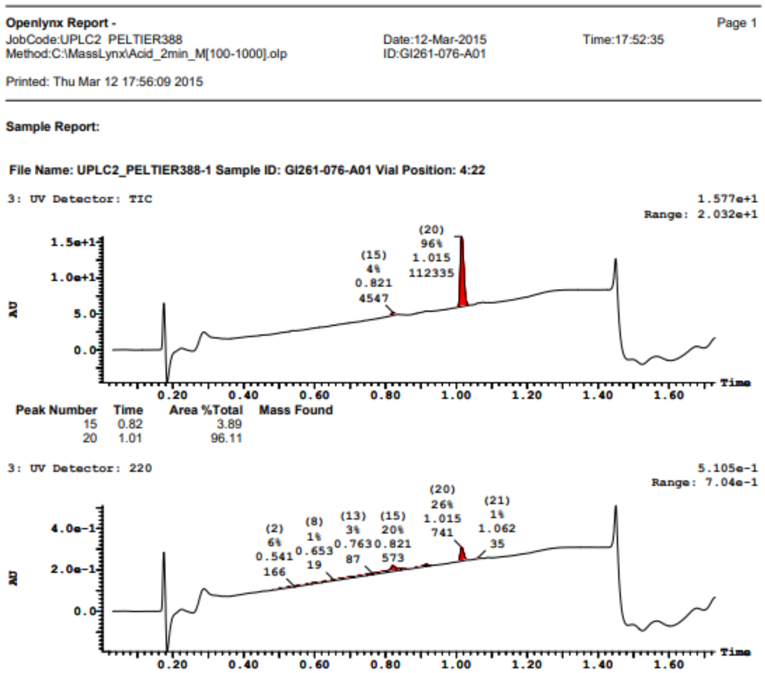

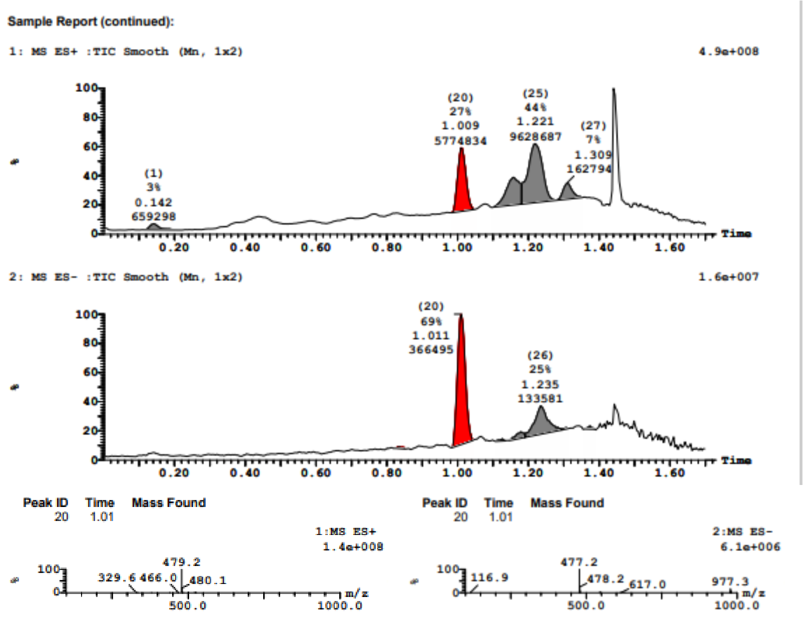

3-cyclobutyl-N-(methylsulfonyl)-4-(4-morpholinopiperidin-1-yl)-1-phenyl-1H-pyrazolo[3,4-b]pyridine-6-carboxamide (49)

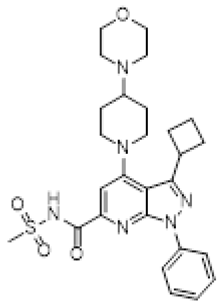

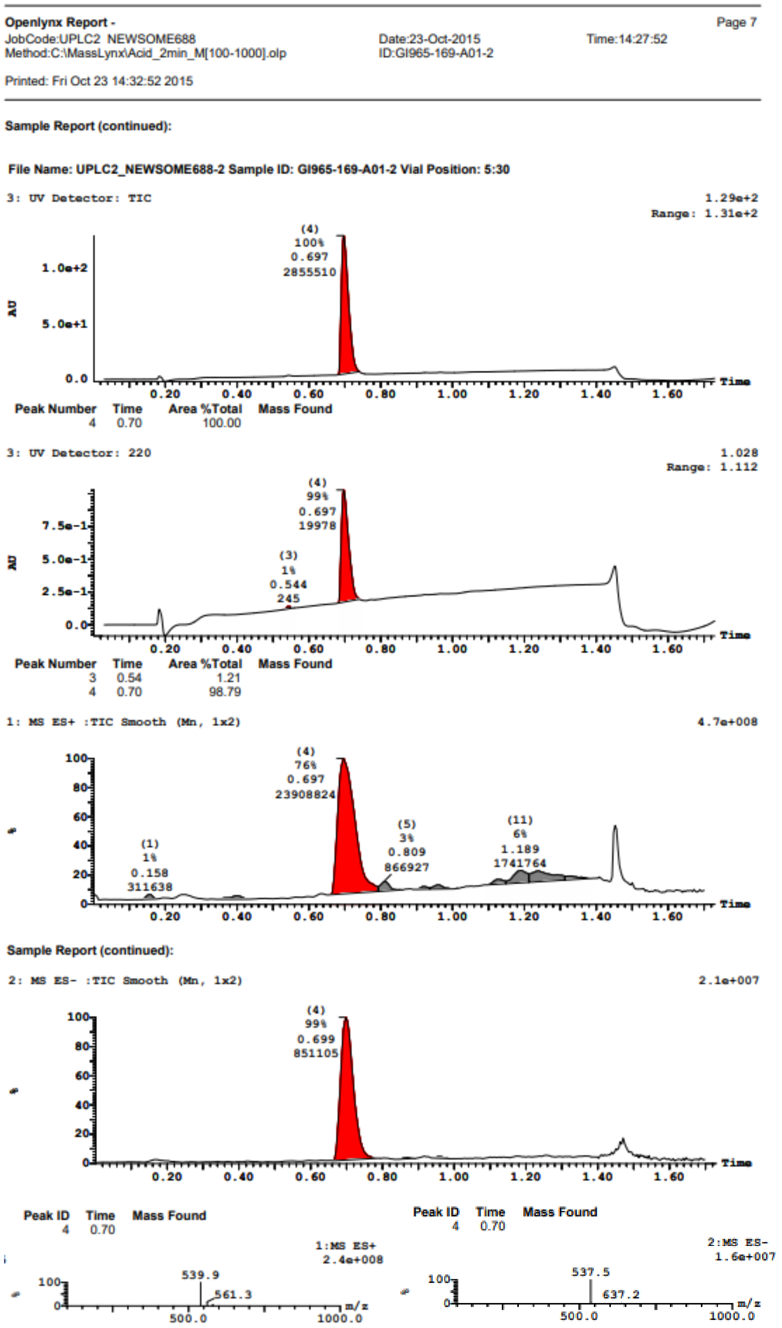

3-cyclobutyl-1-(4-fluorophenyl)-N-(methylsulfonyl)-4-(4-morpholinopiperidin-1-yl)-1H-pyrazolo[3,4-b]pyridine-6-carboxamide hydrochloride (50).

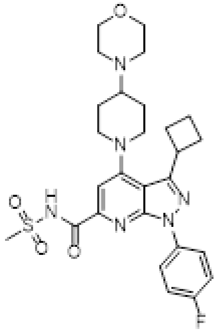

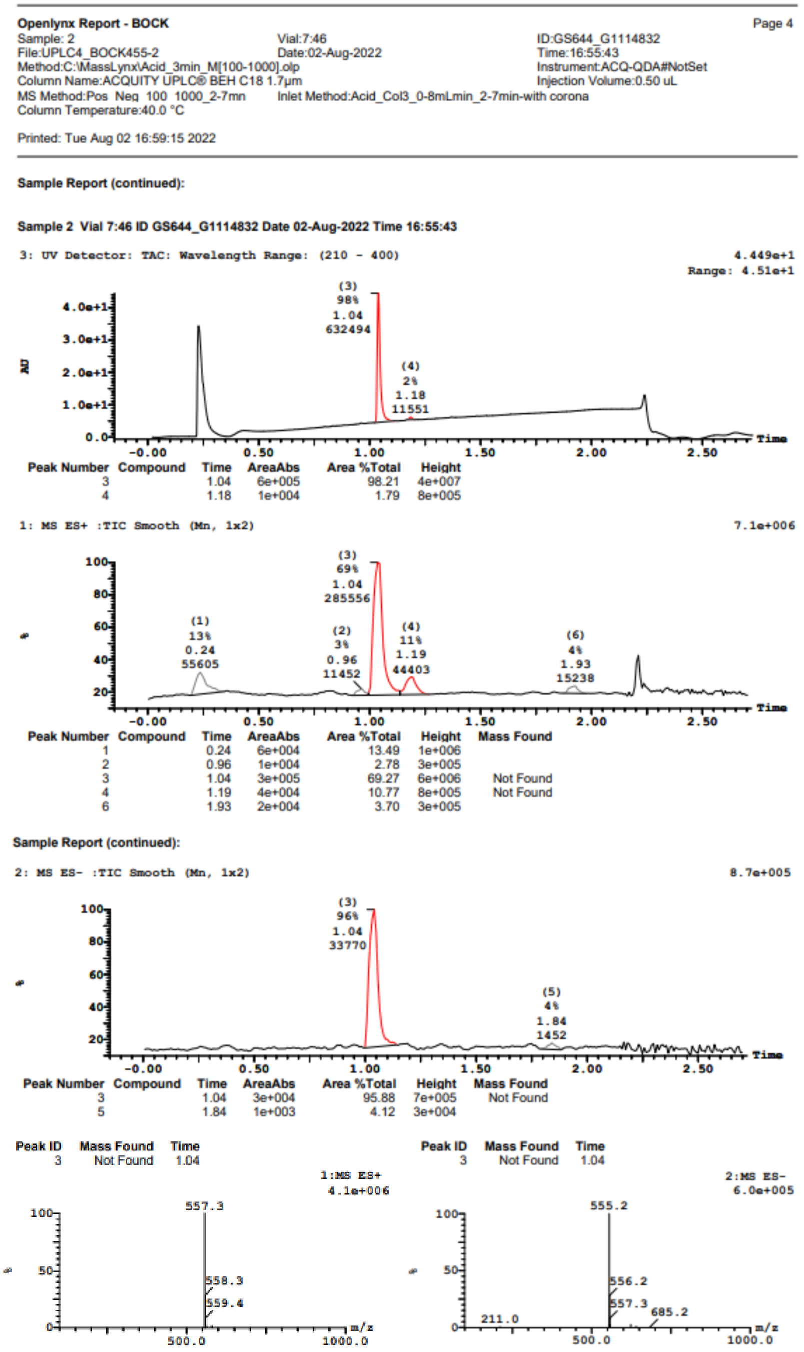

Openlynx Report - BOCK | Page 4

Sample: 2 | Vial:7:46 | ID:GS644_G1114832
File:UPLC4_BOCK455-2 | Date:02-Aug-2022 | Time:16:55:43
Method:C:\MassLynx\Acid_3min_M[100-1000].olp | Instrument:ACQ-QDA#NotSet
Column Name:ACQUITY UPLC® BEH C18 1.7µm | Injection Volume:0.50 uL
MS Method:Pos_Neg_100_1000_2-7mn | Inlet Method:Acid_Col3_0-8mLmin_2-7min-with corona
Column Temperature:40.0 °C

Printed: Tue Aug 02 16:59:15 2022

Sample Report (continued):

Sample 2 Vial 7:46 ID GS644_G1114832 Date 02-Aug-2022 Time 16:55:43

Peak Number	Compound	Time	AreaAbs	Area %Total	Height
3		1.04	6e+005	98.21	4e+007
4		1.18	1e+004	1.79	8e+005

Peak Number	Compound	Time	AreaAbs	Area %Total	Height	Mass Found
1		0.24	6e+004	13.49	1e+006	
2		0.96	1e+004	2.78	3e+005	
3		1.04	3e+005	69.27	6e+006	Not Found
4		1.19	4e+004	10.77	8e+005	Not Found
6		1.93	2e+004	3.70	3e+005	

Sample Report (continued):

Peak Number	Compound	Time	AreaAbs	Area %Total	Height	Mass Found
3		1.04	3e+004	95.88	7e+005	Not Found
5		1.84	1e+003	4.12	3e+004	

Peak ID	Mass Found	Time
3	Not Found	1.04

Peak ID	Mass Found	Time
3	Not Found	1.04

3-cyclobutyl-N-(N,N-dimethylsulfamoyl)-1-(4-fluorophenyl)-4-(4-morpholinopiperidin-1-yl)-1H-pyrazolo[3,4-b]pyridine-6-carboxamide (51)

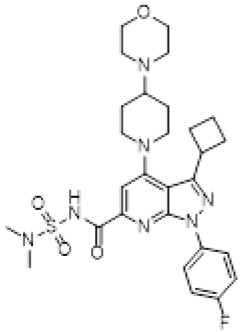

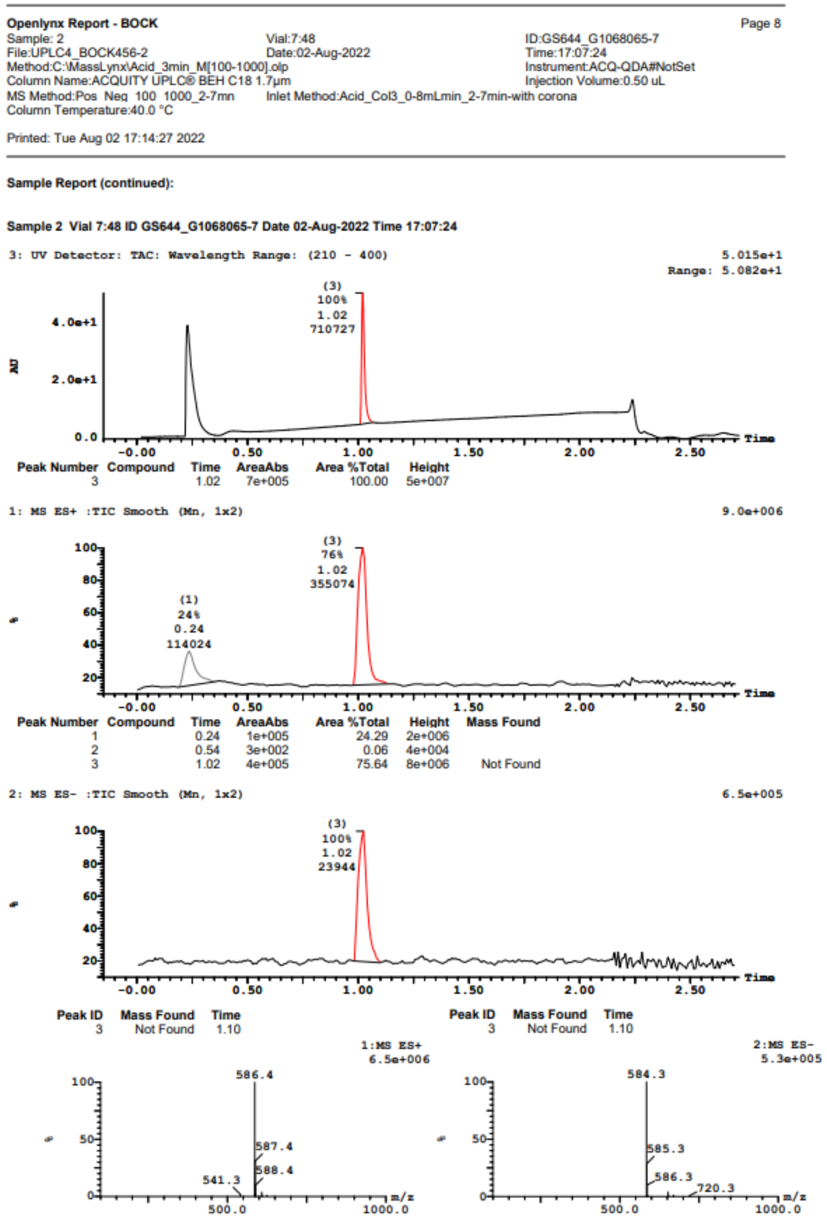

3-Cyclobutyl-N-(N,N-dimethylsulfamoyl)-1-(4-fluorophenyl)-4-(4-methoxy-[1,4'-bipiperidin]-1'-yl)-1H-pyrazolo[3,4-b]pyridine-6-carboxamide (52)

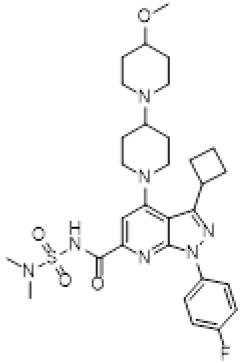

Openlynx Report - Page 16
JobCode:UPLC1 BREBION1693 Date:08-Mar-2016 Time:11:31:22
Method:C:\MassLynx\Acid_3min_M[100-1000].olp ID:GI358-082-N01

Printed: Tue Mar 08 11:34:52 2016

Sample Report (continued):

File Name: UPLC1_BREBION1693-2 Sample ID: GI358-082-N01 Vial Position: 2:2

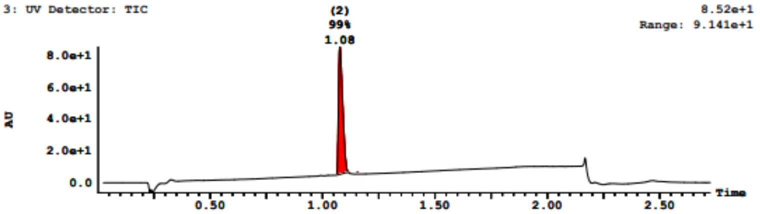

Peak Number	Time	Area %Total	Mass Found
2	1.08	99.37	
5	1.16	0.63	

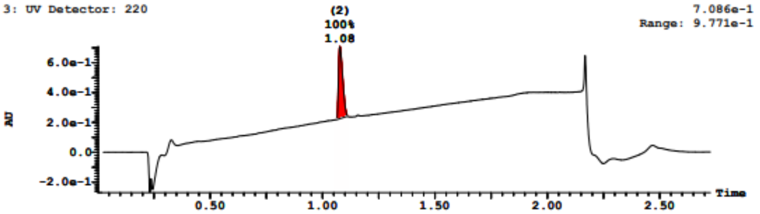

Peak Number	Time	Area %Total	Mass Found
2	1.08	100.00	

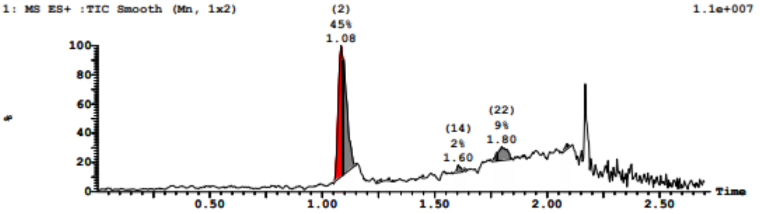

Sample Report (continued):

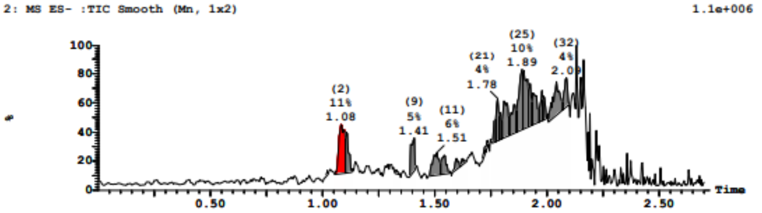

Peak ID	Time	Mass Found
2	1.08	

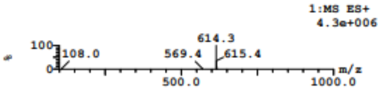

Peak ID	Time	Mass Found
2	1.08	

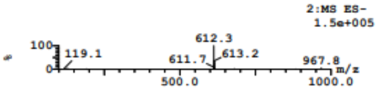

III. Formula Strings

Example Nbr	SMILES	InChIKey
6	COc1ccc(cc1)-c1cc(nc2n(nc(c12)C)-c1ccc(cc1)C)C(=O)O	DBBCECCXYHJOBP-UHFFFAOYSA-N
7	COc1ccc(cc1)-c1cc(nc2n(nc(c12)C)-c1ccccc1)C(=O)O	HLSXELDJNMQUEP-UHFFFAOYSA-N
8	Cc1nn(c2nc(cc(c12)-c1ccc(cc1)N1CCOCC1)C(=O)O)-c1ccccc1	CCRPWWJBCABNNK-UHFFFAOYSA-N
9	Cc1nn(c2nc(cc(c12)-c1ccc(cc1)N1CCOCC1)C(=O)O)-c1cccc(c1)F	UISFEXGUNPBLQS-UHFFFAOYSA-N
10	Cc1nn(c2nc(cc(c12)-c1ccc(cc1)N1CCOCC1)C(=O)O)-c1ccc(cc1)F	IZGZOZXCVXYDRH-UHFFFAOYSA-N
11	Cc1nn(c2nc(cc(c12)-c1ccc(cc1)N1CCOCC1)C(=O)O)C1CC1	UPEMQDNHVSBYSD-UHFFFAOYSA-N
12	Cc1nn(c2nc(cc(c12)-c1ccc(cc1)N1CCOCC1)C(=O)O)C1CCCCC1	IBKMPJNHNWCVTO-UHFFFAOYSA-N
13	Cc1nn(c2nc(cc(c12)-c1ccc(cc1)N1CCOCC1)C(=O)O)C1CCOCC1	COJUPUOIVRWQGQ-UHFFFAOYSA-N
14	CN1CCC(CC1)n1nc(c2c(cc(nc12)C(=O)O)-c1ccc(cc1)N1CCOCC1)C	XDYVNPQRNBECCI-UHFFFAOYSA-N
15	OC(=O)c1cc(c2cnn(c2n1)-c1ccccc1)-c1ccc(cc1)N1CCOCC1	XWAJDKWIIPPQMV-UHFFFAOYSA-N
16	CC(C)c1nn(c2nc(cc(c12)-c1ccc(cc1)N1CCOCC1)C(=O)O)-c1ccccc1	IBJJELHEQPSGNE-UHFFFAOYSA-N
17	CC(C)(C)c1nn(c2nc(cc(c12)-c1ccc(cc1)N1CCOCC1)C(=O)O)-c1ccccc1	ZHRKULVUAGUPSV-UHFFFAOYSA-N
18	OC(=O)c1cc(c2c(nn(c2n1)-c1ccccc1)C1CCC1)-c1ccc(cc1)N1CCOCC1	MOGNFYGRFDBRMA-UHFFFAOYSA-N
19	CC1(CCC1)c1nn(c2nc(cc(c12)-c1ccc(cc1)N1CCOCC1)C(=O)O)-c1ccccc1	HSDFIZXKXKHMLV-UHFFFAOYSA-N
20	CC1(COC1)c1nn(c2nc(cc(c12)-c1ccc(cc1)N1CCOCC1)C(=O)O)-c1ccccc1	LTNUTMRLNACBEF-UHFFFAOYSA-N
21	COCc1nn(c2nc(cc(c12)-c1ccc(cc1)N1CCOCC1)C(=O)O)-c1ccccc1	NKJCHXLBQWLBHP-UHFFFAOYSA-N
22	OCc1nn(c2nc(cc(c12)-c1ccc(cc1)N1CCOCC1)C(=O)O)-c1ccccc1	HKVJPJPPGZBSPU-UHFFFAOYSA-N
23	Cc1nn(c2nc(cc(c12)-c1ccc(nc1)N1CCOCC1)C(=O)O)-c1ccccc1	AXUUHLIQPZMKEH-UHFFFAOYSA-N
24	COCCNc1ccc(cc1)-c1cc(nc2n(nc(c12)C)-c1ccccc1)C(=O)O	RTFYHOUUOMCFPT-UHFFFAOYSA-N
25	COCCN(C)c1ccc(cc1)-c1cc(nc2n(nc(c12)C)-c1ccccc1)C(=O)O	BPDGAFMGZSZPMC-UHFFFAOYSA-N
26	CN(C1CCOCC1)c1ccc(cc1)-c1cc(nc2n(nc(c12)C)-c1ccccc1)C(=O)O	UJZFDFLPALSEBP-UHFFFAOYSA-N
27	Cc1nn(c2nc(cc(c12)-c1ccc(cc1)N1CCC(O)CC1)C(=O)O)-c1ccccc1	JROGWPHFMNZVSM-UHFFFAOYSA-N
28	Cc1nn(c2nc(cc(c12)-c1ccc(cc1)N1CCC(CC1)C#N)C(=O)O)-c1ccccc1	PJORYNYPDCOPKO-UHFFFAOYSA-N
29	CC(=O)c1cc(c2c(nn(c2n1)-c1ccccc1)C)-c1ccc(cc1)N1CCOCC1	SLIHESPSIUDMQF-UHFFFAOYSA-N
30	Cc1nn(c2nc(cc(c12)-c1ccc(cc1)N1CCOCC1)CO)-c1ccccc1	NXZBCBSREQCJFM-UHFFFAOYSA-N
31	CC(O)c1cc(c2c(nn(c2n1)-c1ccccc1)C)-c1ccc(cc1)N1CCOCC1	URUYVMCTFZDDCW-UHFFFAOYSA-N

32	Cc1nn(c2nc(cc(c12)-c1ccc(cc1)N1CCOCC1)C(C)(C)O)-c1ccccc1	HIDXKGPBCQRICV-UHFFFAOYSA-N
33	Cc1nn(c2nc(cc(c12)-c1ccc(cc1)N1CCOCC1)C(=O)NCC(F)(F)F)-c1ccccc1	CHZADCVQVRFTIP-UHFFFAOYSA-N
34	COc1cc(n[nH]1)-c1cc(c2c(nn(c2n1)-c1ccccc1)C)-c1ccc(cc1)N1CCOCC1	ITISUWFJYYWJOY-UHFFFAOYSA-N
35	Cc1nn(c2nc(cc(c12)-c1ccc(cc1)N1CCOCC1)CC(=O)O)-c1ccccc1	QNMSKGWOKFTNEV-UHFFFAOYSA-N
36	Cc1nn(c2nc(cc(c12)-c1ccc(cc1)N1CCOCC1)C(=O)NC#N)-c1ccccc1	WKOKPOKDYFJFDO-UHFFFAOYSA-N
37	Cc1nn(c2nc(cc(c12)-c1ccc(cc1)N1CCOCC1)C(=O)NS(=O)(=O)C)-c1ccccc1	BDJNPORJVYSUKM-UHFFFAOYSA-N
38	CCS(=O)(=O)NC(=O)c1cc(c2c(nn(c2n1)-c1ccccc1)C)-c1ccc(cc1)N1CCOCC1	NHXXXFPAEPFCEJ-UHFFFAOYSA-N
39	CS(=O)(=O)NC(=O)c1cc(c2c(nn(c2n1)-c1ccccc1)C1CCC1)-c1ccc(cc1)N1CCOCC1	WFNQIZJEXRBAGM-UHFFFAOYSA-N
40	COCCN(C)c1ccc(cc1)-c1cc(nc2n(nc(c12)C1CCC1)-c1ccccc1)C(=O)O	URRKQSFCFTURGQ-UHFFFAOYSA-N
41	COCCN(C)c1ccc(cc1)-c1cc(nc2n(nc(c12)C1CCC1)-c1ccccc1)C(=O)NS(=O)(=O)C	FWMMHSLVEULOGY-UHFFFAOYSA-N
42	COCCN(C)c1ccc(cn1)-c1cc(nc2n(nc(c12)C1CCC1)-c1ccccc1)C(=O)O	LWCJLJUCPQVMJV-UHFFFAOYSA-N
43	COCCN(C)c1ccc(cn1)-c1cc(nc2n(nc(c12)C1CCC1)-c1ccccc1)C(=O)NS(=O)(=O)C	MDRFUQSZEQUSKS-UHFFFAOYSA-N
44	OC(=O)c1cc(c2c(nn(c2n1)-c1ccccc1)C1CCC1)-c1ccc(nc1)N1CCC(CC1)C#N	SGQFMCAELKGFNT-UHFFFAOYSA-N
45	CS(=O)(=O)NC(=O)c1cc(c2c(nn(c2n1)-c1ccccc1)C1CCC1)-c1ccc(nc1)N1CCC(CC1)C#N	JXHOIIUOMWWZOE-UHFFFAOYSA-N
46	CS(=O)(=O)NC(=O)c1cc(c2c(nn(c2n1)-c1ccccc1)C1CCC1)N1CCCCC1	HYXARSPXEMGVIU-UHFFFAOYSA-N
47	COC1CCN(CC1)c1cc(nc2n(nc(c12)C1CCC1)-c1ccccc1)C(=O)NS(=O)(=O)C	NRQGRQPZOTWRKQ-UHFFFAOYSA-N
48	CS(=O)(=O)NC(=O)c1cc(c2c(nn(c2n1)-c1ccccc1)C1CCC1)N1CCC(CC1)C#N	VFXUCFYREUJPRG-UHFFFAOYSA-N
49	CS(=O)(=O)NC(=O)c1cc(c2c(nn(c2n1)-c1ccccc1)C1CCC1)N1CCC(CC1)N1CCOCC1	XYBQSRRFUUGKSB-UHFFFAOYSA-N
50	CS(=O)(=O)NC(=O)c1cc(c2c(nn(c2n1)-c1ccc(cc1)F)C1CCC1)N1CCC(CC1)N1CCOCC1	VELJFUONOZUMGS-UHFFFAOYSA-N
51	CN(C)S(=O)(=O)NC(=O)c1cc(c2c(nn(c2n1)-c1ccc(cc1)F)C1CCC1)N1CCC(CC1)N1CCOCC1	HFDDYMAKGOBYDX-UHFFFAOYSA-N
52	COC1CCN(CC1)C1CCN(CC1)c1cc(nc2n(nc(c12)C1CCC1)-c1ccc(cc1)F)C(=O)NS(=O)(=O)N(C)C	JPIAJLPRTOWAEC-UHFFFAOYSA-N
